# Supplementary material for: Earliest modern human genomes constrain timing of Neanderthal admixture
Source: Nature. 2024 Dec 12;638(8051):711–7. doi: 10.1038/s41586-024-08420-x (PMC11839475; doi:10.1038/s41586-024-08420-x)
Supplement: Supplementary file 1 — Pdf file containing Supplementary Information chapters from 1 to 20. [file 41586_2024_8420_MOESM1_ESM.pdf]

nature portfolio

https://doi.org/10.1038/s41586-024-08420-x

Supplementary information

Earliest modern human genomes constrain timing of Neanderthal admixture

In the format provided by the
authors and unedited

Supplementary Information

Table of Contents:

Supplementary Information 1 **4**
Specimens Examined in This Study 4
Zlatý kůň specimens 4
Ilsenhöhle in Ranis specimens 6
1932-1938 excavations: 6
2016-2022 excavations: 9
Supplementary Information 2 **10**
Bone Pre-Screening and Radiocarbon Dating 10
Bone preservation screening and sampling 10
Collagen extraction and dating 11
Radiocarbon dating of related individuals 13
Supplementary Information 3 **15**
Data Processing and Quality Controls for Low Coverage Genomes 15
Initial processing 15
Coverage and contamination estimates in the capture data 16
1240k captures 16
Archaic Admixture captures 19
Y chromosome capture 20
Supplementary Information 4 **21**
Data Processing & General Filters for High Coverage Genomes 21
Initial processing 21
Spurious alignments, length and quality filters 22
Contamination and coverage 23
Supplementary Information 5 **27**
Genotyping of the High Coverage Genomes 27
Supplementary Information 6 **30**
Biological Kinship 30
Supplementary Information 7 **38**
Heterozygosity and Consanguinity 38
Heterozygosity estimates for high coverage genomes from the genotype calls 38
Inference of consanguinity 40
Detection of HBD from genotype calls for two high coverage genomes 40
ROH for low coverage capture data 44
Supplementary Information 8 **48**

IBD Analysis 48
Downsampling experiment on the two high-coverage genomes 48
Applying ancIBD to other samples 50
IBD-based dating 53
Supplementary Information 9 **57**
Population Genomic Analysis 57
Analysis using the 1240k capture sites 57
Genetic clustering analysis of the Ranis individuals 57
Testing the genetic relationship of Ranis to other hunter-gatherer (HG) individuals 58
Exploration of the genetic contribution of Ranis/Zlatý kůň to later populations 61
Ranis, African populations and Basal Eurasian ancestry 63
Testing the genetic relationship of Ranis with Late Neandertals and Denisovans 66
Phylogenetic position of Ranis (qpGraph modeling) 68
Analysis using the high coverage shotgun data 71
Comparison of high-coverage shotgun data with Buran Kaya III 73
Supplementary Information 10 **77**
Molecular Dating by Branch Shortening 77
Supplementary Information 11 **81**
Population Size, Age and Split Time Estimates Using PSMC 81
Inference of population size changes over time 81
Inference of population size changes over time corrected for the effect of background selection 86
Age estimates using demographic histories 87
Split time estimates 90
Supplementary Information 12 **94**
Demographic History Inference 94
Supplementary Information 13 **105**
Neandertal Ancestry in the High Coverage Genomes 105
Calling of the Neandertal segments 105
Testing the number of admixture pulses and dating Neandertal introgression 106
Extended pulse model 114
Supplementary Information 14 **116**
Neandertal Ancestry in the Low Coverage Genomes 116
Detecting archaic segments from the Archaic Admixture capture data 117
Testing the number of admixture pulses and dating Neandertal introgression 120
Estimates of generations since the introgression event 122
Estimates of Neandertal ancestry proportion using the segments 122
Calculation of Neandertal ancestry using f4-ratio on 1240k capture data 123
Supplementary Information 15 **126**
Neandertal Ancestry Correlation 126
Calling of Neandertal segments 126
Calculating the correlation of Neandertal segments 126

Supplementary Information 16 **130**
Sharing of Breakpoints in Neandertal Segments **130**
Segments of Neandertal ancestry 130
Neandertal fragments and breakpoints 133
Sharing of breakpoints 134
Supplementary Information 17 **141**
Neandertal Deserts 141
Supplementary Information 18 **144**
Y Chromosome Analysis 144
Haplogroup detection 145
Supplementary Information 19 **147**
The Human Leukocyte Antigen (HLA) Analysis 147
HLA haplotypes of Ranis13 and Zlatý kůň 147
Homozygosity at HLA loci in worldwide human populations 149
Supplementary Information 20 **155**
Phenotypic Inference 155
References **161**

Supplementary Information 1

Specimens Examined in This Study

Hélène Rougier*, Arev Pelin Sümer, Elena Essel, Jörg Orschiedt, Matthias Meyer, Johannes Krause

Zlatý kůň specimens

ZKU001 (SP.C.5428, P7AP 379, P7d1/2006): Left humerus[1]. There are no curatorial records that this bone was conserved with any consolidants since its discovery, and was thus thought to be untreated. The humerus was previously sampled for DNA and dating, however, these attempts were unsuccessful. Thus, we removed a total of twelve micro-samples varying in weight between 4.2 mg and 24.8 mg, and screened six of them for DNA preservation. In addition, we collected a sample of sediment that accumulated inside the shaft of the bone. To assess the fauna which might have been inhabiting the area at the time or after the death of the individual the humerus stems from, the library from the sediment sample was subsequently enriched for mammalian mitochondrial DNA using a capture array[2].

We did not find any evidence of ancient human DNA preservation in the six micro-samples. However, we found traces of ancient, and mostly present day, bovid DNA in all samples. We investigated this further by blasting all recovered DNA fragments, and a second set containing only deaminated fragments, in order to assess whether the human percentage would increase in proportion when looking at only deaminated fragments. We repeated the same process after mapping the raw sequencing data to the bovid reference genome, *bosTau8*, and found more alignments in general but no evidence of ancient DNA damage, i.e. no evidence of ancient human or bovid DNA. These results indicate that the specimen might have been treated with animal glue, in the same way as the Zlatý kůň skull[3]. Due to the absence of any detectable ancient human DNA in the humerus, we did not proceed with any further genetic investigation of this specimen.

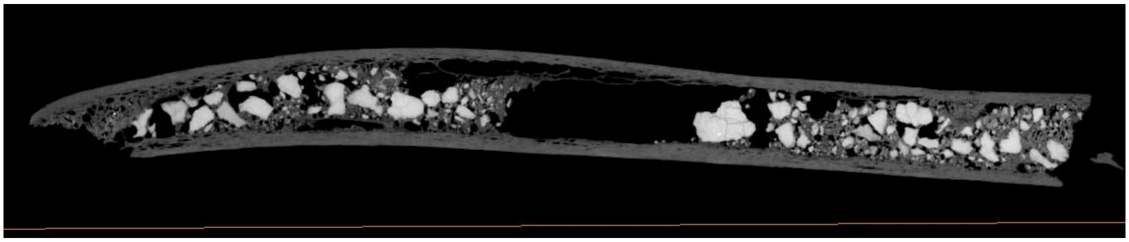

Supplementary Figure 1.1: Snapshot from the microCT scan of the ZKU001 humerus. The scans were done by Heiko Temming, MPI-EVA, and used to determine the best sampling strategies combined with the NIR collagen prediction results.

Supplementary Table 1.1: Top blast results for one of the libraries obtained from the ZKU001 humerus, Lib.L.8205, mapped to the reference genome *hg19* and *bosTau8*.

bosTau8				***hg19***			
**Deaminated**		**All**		**Deaminated**		**All**	
Count	Species	Count	Species	Count	Species	Count	Species
2558	*Bos taurus*	14665	*Bos taurus*	208	*Homo sapiens*	4887	*Homo sapiens*
428	*Homo sapiens*	2677	*Homo sapiens*	79	*Bos taurus*	1248	*Bos taurus*
318	*Ovis ammon aries*	1867	*Ovis ammon aries*	37	*Ovis ammon aries*	440	*Sus scrofa*
281	*Sus scrofa*	1511	*Sus scrofa*	26	LK3 transgenic mice	417	*Ovis ammon aries*

In addition, we analyzed the mitochondrial DNA sequences captured with probes spanning 242 mammalian mitochondrial genomes, including human mtDNA, and analyzed these following Slon et al., 2017[4] using MEGAN[5]. We detected 78.4% of the sequences (n = 7,791) to be assigned to Bovidae, particularly to *Bos taurus.* Terminal C to T substitutions detected in these sequences were low, 4.1% (CI 3.0% - 5.4%) and 3.6% (CI 2.6% - 4.9%) for 5' and 3' ends, respectively, and supported our observations suggesting that the specimen was treated with cow glue. Nevertheless, we were able to detect traces of ancient reindeer (*Rangifer tarandus*) DNA and Przewalski's horse (*Equus przewalskii*) DNA with a few sequences, dominated by the large amount of present day bovid DNA (Table S1.6). However, these results should be considered with caution as the terminal C to T substitutions for both ancient taxa are below 10% on the 3' end, which is the threshold in this analysis. We did not detect any ancient human DNA in the sediment sample.

ZKU002 (SP.B.6627): Right petrous of the Zlatý kůň skull. Previously sampled and published in Prüfer et al., 2021[3], therefore no further sampling was performed. An additional library (ZKU002.A0103) was created from the existing lysate and sequenced together with one of the previous libraries (ZKU002.A0102) to obtain a high coverage genome. The ZKU002.A0101 library created in the previous study was treated with *E.coli* UDG and Endonuclease VIII ('an UDG-half'). Despite being sequenced to a very low coverage, it was included in the genotyping step to account for possible errors in the first two libraries. Libraries ZKU002.A0102 and ZKU002.A0103 were shotgun sequenced to high coverage at the SciLifeLab in Stockholm (see Supplementary Information 4 for details). Existing data includes captures for the double stranded and a half-UDG treated library ZKU002.A0101. No additional captures were performed in this study.

Ilsenhöhle in Ranis specimens

Thirteen specimens associated with the Lincombian-Ranisian-Jerzmanowician technocomplex (LRJ) were described and DNA was extracted from eleven of these for mtDNA analyses in Mylopotamitaki et al., 2024[6]. In this study, we generated data from additional sub-samples from all 13 specimens as well as from the libraries generated previously.

1932-1938 excavations:

Morphologically identified specimens:

RNI004 (SP57770, SP.C.4556; Spec. ID: R10875): Hand phalanx. Nine samples varying between 5 mg and 27.9 mg in weight were removed using a sterile dentistry drill and seven of them were converted into libraries for screening. Among those, three libraries were selected for additional 1240k capture[7], and two for the Archaic Admixture array capture[8] (Tables S1.1-S1.4).

RNI006 (SP57771, SP.C.4557; Spec. ID: R10876): Juvenile clavicle. Nine samples varying between 9 mg and 20.1 mg in weight were collected, and seven of them were turned into libraries for screening. After ancient DNA preservation assessment and a final selection, three libraries were captured with the 1240k SNP array and two with the Archaic Admixture array (Tables S1.1-S1.4).

RNI010 (SP57772, SP.C.4558; Spec. ID: R10874): Juvenile clavicle. Nine samples varying between 9.3 mg and 37.1 mg in weight were collected, and seven of them were turned into libraries for screening. Among them, three libraries were subsequently captured with the 1240k array and two with the Archaic Admixture array. After the initial screening, we determined that this specimen originates from a male individual. Therefore, we captured two of the best libraries for the Y chromosome using both the YMCA, an array designed specifically to increase the number of Y-haplogroup informative SNPs captured[9,10], and a whole Y chromosome capture array[9] (Tables S1.1-S1.5).

RNI012 (SP57773, SP.C.4559; Spec. ID: R10879): Femur fragment. Eleven samples varying between 9.3 mg and 63.9 mg in weight were collected, and eight of them were turned into libraries for screening. All libraries were relatively rich in endogenous DNA (2-5%), although moderately contaminated (6-15%). However, as we found that three other specimens belonged to the same individual as RNI012, we captured only one library from this specimen with the 1240k capture array, and none with the Archaic Admixture array (Tables S1.1-S1.4).

RNI013 (SP57774, SP.C.4560; Spec. ID: R10873): Metacarpal distal extremity. A single sample of 42.1 mg was collected from this specimen and four libraries were constructed from the same extract. All four libraries were sent to SciLifeLab in Stockholm for deeper shotgun sequencing to obtain a high coverage genome (see Supplementary Information 4 for details). A single library was captured with the 1240k SNP array.

Specimens identified by palaeoproteomics:

RNI080 (SP11373, SP.B.2217; Spec. ID: R10396; Proteomic ID: Ra-0353): Long bone fragment. Five samples varying between 7.7 mg and 17.5 mg in weight were collected and a library was constructed from each subsample for screening. Four of these libraries were captured with the 1240k array, and two with the Archaic Admixture array. One of the libraries (Lib.H.3516) was highly contaminated with present day human DNA, hence it was not included in the final dataset for analysis. While analyzing the generated data, we found that this specimen belonged to the same individual as RNI012, RNI081 and RNI085 (Tables S1.1-S1.2).

RNI081 (SP11374, SP.B.2218; Spec. ID: R10355; Proteomic ID: Ra-0087): Rib fragment. Three samples between 7 mg and 10.5 mg in weight were collected and turned into libraries. All

three of them were screened for endogenous DNA, and two were captured with the 1240k array. One of these libraries (Lib.H.3517) was highly contaminated, hence it was removed from our dataset. The remaining library with low contamination and ancient DNA preservation was captured with the Archaic Admixture array (Tables S1.1-S1.4). This specimen is one of the four specimens coming from a single individual, along with RNI012, RNI080 and RNI085.

RNI082 (SP.C.5031; Spec. ID: R10400; Proteomic ID: Ra-2310): Long bone fragment. This specimen was not sampled for DNA or ^{14}C dating in Mylopotamitaki et al., 2024[6]. For DNA extraction, we collected four microsamples varying between 6.2 mg and 9.5 mg in weight. Three of these microsamples were used to create libraries and screened for DNA. Contamination levels for these libraries varied between 71% and 91%, hence they were not selected for subsequent DNA captures and downstream analysis (Tables S1.1-S1.2).

RNI083 (SP.C.5032; Spec. ID: R10318; Proteomic ID: Ra-2326): Rib fragment. This specimen was not sampled for DNA or ^{14}C dating in Mylopotamitaki et al., 2024[6] either. We collected six microsamples varying between 3 mg and 11.5 mg in weight. Four of them were screened for aDNA preservation, and contamination estimates for all were very high, similar to RNI082. Only one library contained less than 50% contamination (44%), however, we decided not to proceed with it since the number of deaminated sequences was very low, which would have been the only reliable portion of the data if we were to proceed with generating SNP capture data (Tables S1.1-S1.2).

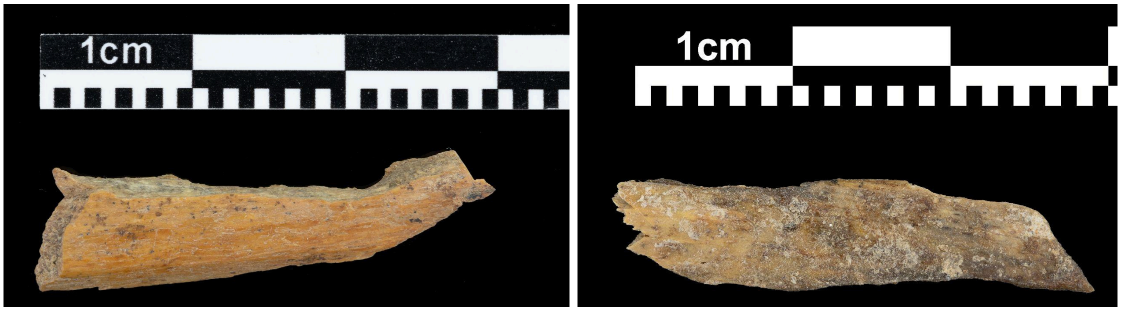

Supplementary Figure 1.2: RNI082 on the left and RNI083 on the right. Libraries from both specimens were excluded from the dataset after initial screening, and not used in the analysis. Photos taken by Sylvio Tüpke, MPI-EVA.

2016-2022 excavations:

Specimens identified by palaeoproteomics:

RNI084 (SP12587, SP.C.4594; Spec. ID: 16/116-159416; Proteomic ID: Ra-2510): Long bone fragment. A single sample of 6.1 mg was collected from the specimen and a single library was made. It was captured with both arrays already mentioned above, but excluded from most of our downstream analyses due to high levels of contamination (Tables S1.1-S1.4).

RNI085 (SP12588, SP.C.4595; Spec. ID: 16/116-159327; Proteomic ID: Ra-1939): Long bone fragment. A single sample of 14 mg was collected, and three libraries were made from the lysate. All libraries were captured with both the 1240k and Archaic Admixture arrays (Tables S1.1-S1.4). This specimen originates from the same individual as RNI012, RNI080 and RNI081.

RNI086 (SP12589, SP.C.4596; Spec. ID: 16/116-159253; Proteomic ID: Ra-1930): Long bone fragment. A single sample of 9.9 mg was collected from the specimen and a single library was made, which was then captured with both arrays as detailed above. However, this library contained around 25% contamination, hence it was excluded from the majority of our downstream analyses (Tables S1.1-S1.4).

RNI087 (SP12590, SP.C.4597; Spec. ID: 16/116-159199; Proteomic ID: Ra-2005): Long bone fragment. A single sample of 10 mg was collected, and three libraries were made from the resulting lysate. All libraries were captured with both the 1240k and Archaic Admixture arrays. Furthermore, as we detected this specimen to belong to a genetically male individual, we captured one library with the complete Y chromosome capture array which enriches the libraries for ~6.9 Mb of the Y chromosome[9], and two libraries with the YMCA[10] (Tables S1.1-S1.5).

Supplementary Information 2

Bone Pre-Screening and Radiocarbon Dating

Helen Fewlass*, Elena Essel, Arev Pelin Sümer

Bone preservation screening and sampling

Numerous attempts at radiocarbon dating of the Zlatý kůň cranium have produced inconsistent results that under-estimate the true age of the specimen due to contamination with bone collagen-based glue[3,11]. We therefore attempted radiocarbon dating of the Zlatý kůň humerus (ZKU001), which is believed to originate from the same individual[1]. This bone was thought to be untreated with glue so it was selected for ^{14}C dating, but subsequent DNA analysis revealed a high proportion of modern cow DNA, consistent with the presence of collagen-based glue. In addition, we aimed to date the two additional bone fragments from Ranis, RNI082 and RNI083, which were identified as *Homo sp.* through proteomics[6] but have not been previously sampled for aDNA analysis or radiocarbon dating (see Supplementary Information 1).

To minimize destruction of the samples and determine the likelihood of obtaining enough collagen for radiocarbon dating prior to sampling, we non-destructively pre-screened the collagen content of the bone fragments (ZKU001, RNI082 and RNI083) using a portable LabSpec4 Hi-res near infrared spectrometer (NIR) with a small-diameter reflectance probe (Malvern Panalytical, Germany), following the method reported in Sponheimer et al., 2019[12] and supplemented by an additional in-house dataset of NIR scans of bones with quantified collagen yields. The bone surfaces of the specimens were scanned in multiple locations with at least two NIR spectra collected from each location in order to check the replicability of our estimates and determine the optimum sampling location. NIR screening indicated collagen was sufficiently preserved for radiocarbon dating (~1% minimum requirement) in RNI082 and ZKU001, with very good preservation indicated for RNI082 (mean 9.9 ± 1.1 % (1SD), n = 8, root mean squared error (RMSE) of the model is ± 3 %) and a lower level for ZKU001 (mean 5.2 ± 1.7 % (1SD), n = 42, RMSE ± 3 %). The locations with the highest predicted collagen content were targeted for sampling for radiocarbon dating. Sampling was performed in the cleanroom facilities of Max Planck Institute for Evolutionary Anthropology in Leipzig, using a sterile diamond saw

(Superflex Diamond Disc). A piece of 225 mg from the ZKU001 specimen and a piece of 144 mg from the RNI082 specimen were sampled and sent to the Ancient Genomics Lab of The Francis Crick Institute for collagen extraction. NIR screening indicated contamination was present on the surface of RNI083 which prevented reliable collagen predictions. This is in keeping with the presence of glue detected on the surface of other human bone fragments from the 1932-1936 collection from Ranis[6], so this bone was not sampled for radiocarbon dating.

Collagen extraction and dating

The collagen extraction was done following the acid-base-acid plus ultrafiltration protocol for small samples outlined in Fewlass et al., 2019[13] in the Ancient Genomics Lab at the Francis Crick Institute, UK. The collagen yields from both bones were well above the 1% minimum requirement for dating and agreed well with the NIR model predictions. The two collagen extracts were measured on a vario PYRO elemental-analyser (EA) coupled to an Elementar Isoprime precisION isotope-mass-spectrometer (IRMS) in the School of Geographical Sciences at the University of Bristol, UK to determine their elemental and stable isotopic values. The values from RNI082 fell within ranges of well-preserved collagen, consistent with published data from other Ranis individuals[6] indicating the extract was suitable for dating (C: 47.5%, N: 17.0%, C/N: 3.3, δ^{13}C: -18.8‰, δ^{15}N: 10.5‰). In contrast, the extract from ZKU001 had a C:N value at the limit of the range accepted for well-preserved collagen, potentially indicating the presence of a low level of external carbon (C: 48%, N: 15.3%, C/N: 3.6, δ^{13}C: -20.3‰, δ^{15}N: 10.5‰).

The extracted collagen was graphitised on an AGE 3 system[14] and dated on a MICADAS accelerator mass spectrometer (AMS)[15,16] at the Laboratory for Ion Beam Physics at ETH Zurich, Switzerland. Dates were then calibrated using the IntCal20 calibration curve[17] in OxCal 4.4[18].

Supplementary Table 2.1: Summary of the radiocarbon dating of ZKU001 and RNI082 produced in this study and the 10 dates from Ranis human bones published in Mylopotamitaki et al., 2024[6]. Dates are rounded to the nearest 10 years and calibrated using the IntCal20 calibration curve[17] in OxCal 4.4[18].

Individual	Specimen ID	Arch ID	Bone weight (mg)	Collagen yield %	AMS lab no	C14 age (BP)	error	95% cal BP	Ref.
Ranis87	RNI087	16/116-159199	83.0	15.1	ETH-127621	44,330	1090	49,540-44,850	6
Ranis84	RNI084	16/116-159416	103.2	14.4	ETH-127622	43,890	1040	48,690-44,640	6
Ranis86	RNI086	16/116-159253	120.4	13	ETH-127619	42,730	910	47,460-44,100	6
Ranis10	RNI010	R10874	61.2	10.8	ETH-127627	42,410	860	46,950-43,450	6
Ranis13	RNI013	R10873	79.9	11.6	ETH-127629	42,220	840	46,580-43,400	6
Ranis12	RNI012	R10879	87.4	15.0	ETH-127628	41,430	770	45,580-43,030	6
	RNI080	R10396	160.0	15.5	ETH-115246	41,570	420	45,180-43,420	6
	RNI085	16/116-159327	99.1	14.9	ETH-127620	41,800	800	45,970-43,200	6
Ranis4	RNI004	R10875	70.9	11.0	ETH-127625	40,320	670	44,530-42,690	6
Ranis6	RNI006	R10876	54.8	13.0	ETH-127626	39,090	580	43,860-42,200	6
Ranis82	RNI082	R10400	144	10.3	ETH-140140	44,140	580	47,900-45,420	This study
	ZKU001		225	5.7	ETH-140139	34,690	200	40,400-39,400	This study

The AMS date from RNI082 (R10400; ETH-140140: 44,140 ± 580 BP) of 47,900 - 45,420 cal BP is in the same range as 10 dates previously obtained from *Homo sapiens* bone fragments from the LRJ contexts at Ranis (Supplementary Figure 2.1) and the 47,500 - 43,300 cal BP modeled range of the LRJ occupation of the site based on recently excavated material[6]. The AMS date from ZKU001 (ETH-140139: 34,690 ± 200 BP; 40,400 - 39,400 cal BP) is older than the ^{14}C

dates previously produced for the Zlatý kůň cranium, the most recent of which was performed using a robust compound-specific method that produced an age of ~34,000 cal BP (OxA-38022: 29,650 ± 650 BP)[3]. The high level of *Bos taurus* DNA in the cranium sample led the authors to consider the date as a minimum age due to the presence of collagen-based glue applied to the surface of the bone, which cannot be detected or reliably separated from the endogenous collagen content during pretreatment. Considering the similarly high levels of modern cow DNA detected in the genetic analysis of the ZKU001 humerus and the elevated C:N value of 3.6, the new ^{14}C determination is also suspected to be affected by collagen-based glue contamination and is therefore also considered to be a minimum age.

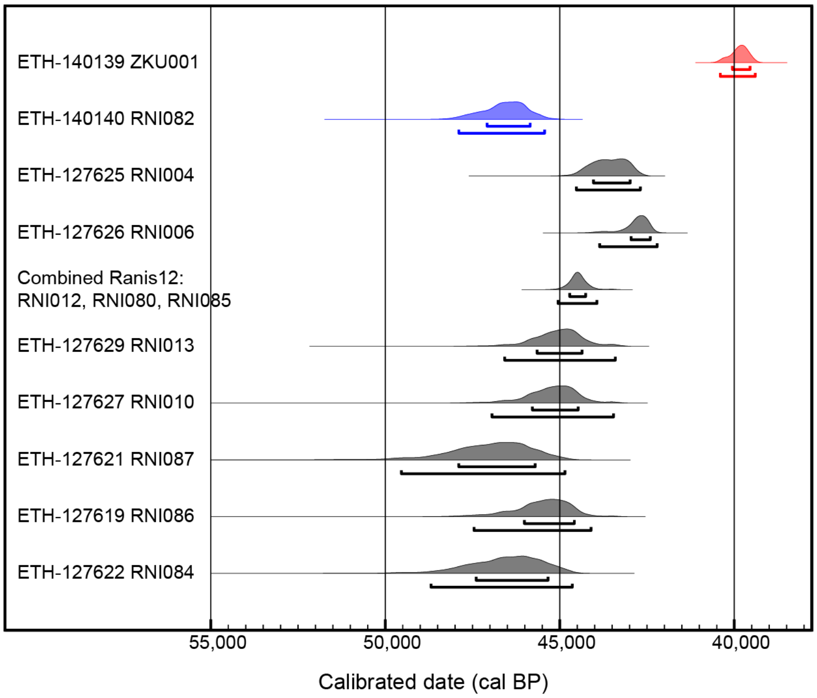

Supplementary Figure 2.1: Calibrated ranges of radiocarbon dates (95% probability) from the ZKU001 humerus (this study, red), RNI082 (this study, blue) and published dates from other Ranis individuals[6] (grey) shown in Supplementary Table 2.1. The date for ZKU001 is considered a minimum age.

Radiocarbon dating of related individuals

Analyses performed with KIN to test for biological relatedness between the Ranis bone fragments are detailed in Supplementary Information 6. The results indicate that bone fragments RNI012, RNI080, RNI081 and RNI085 all originate from one individual, called Ranis12. We combined the dates from the three of these fragments which have been directly ^{14}C dated using

the Combine function in OxCal. The X^2 test shows the three dates to be statistically indistinguishable (X^2 test: T = 0.1 (5% 6.0), df = 2) with a weighted mean age of 45,050 - 43,930 cal BP at 95% probability. The KIN analysis indicates a mother-daughter relationship between Ranis6 (RNI006; daughter) and Ranis4 (RNI004; mother). The direct dates of these two individuals are also statistically indistinguishable (X^2 test: T = 1.820 (5% 3.841), df = 2). A second degree relationship between Ranis12 (RNI012) and Ranis4 (RNI004) is also indicated through KIN. The three dates from Ranis12 and the date from Ranis4 are likewise statistically indistinguishable (X^2 test: T = 1.142 (5% 3.841), df = 1). The calibrated ranges from Ranis12, Ranis6 and Ranis4 all overlap at the 95% probability range, consistent with the findings from the genetics analyses.

Supplementary Information 3

Data Processing and Quality Controls for Low Coverage Genomes

Arev Pelin Sümer*, Vanessa Villalba-Mouco, Elena Essel

Initial processing

In order to obtain nuclear genomes from thirteen early modern human specimens from Ranis, which were described in Mylopotamitaki et al., 2024[6] and Supplementary Information 1, we generated nuclear DNA data for all of the existing libraries previously described in Mylopotamitaki et al., 2024[6], and generated 40 additional libraries for these thirteen specimens (Table S1.1).

After removing a thin layer of surface material with a sterile dentistry drill under clean room conditions, between 3 mg and 63.9 mg of powder was collected from the specimens. DNA extracts were prepared following the protocol described in Rohland et al., 2018[19], on an automated handling platform and using binding buffer "D". Subsequently, purified DNA molecules were recovered in 30 µl of elution buffer. In order to enhance the recovery of endogenous DNA from the samples and to preserve deamination signals, we did not treat extracts with uracil DNA glycosylase (UDG).

We used an automated version of the single-stranded DNA library preparation protocol[20], and produced shallow shotgun sequencing data (depth of 3-5 million reads) from all libraries after their amplification by using a 75 bp single-read configuration at the former Max Planck Institute for the Science of Human History in Jena, Germany, and at the Max Planck Institute for Evolutionary Anthropology (MPI-EVA) in Leipzig, Germany, on HiSeq4000 and NextSeq500 platforms, respectively. We demultiplexed the resulting sequences based on perfect matching of the expected index combinations, mapped them to the *hg19* reference genome with BWA (version 0.5.10-evan.9-1-g44db244, https://github.com/mpieva/network-aware-bwa) with the ancient parameters ("-n 0.01 –o 2 –l 16500")[21] and filtered for mapping quality of 25 and minimum length cutoff of 30 base pairs. We then estimated levels of present-day DNA contamination in each library using AuthentiCT[22], and investigated the terminal ancient DNA

damage in each library. After this initial check of ancient DNA (aDNA) preservation and contamination in the libraries (Table S1.2), we found the four libraries from RNI013 to be extremely rich in endogenous DNA (~30%) with low present-day human DNA contamination estimates, and decided to deeply shotgun sequence these in order to obtain a high coverage genome from this individual (see Supplementary Information 4). We also observed that the libraries from two specimens, RNI082 and RNI083, were highly contaminated with estimates of present-day human DNA contamination ranging between 45% and 90%, hence we excluded them from further data production.

Based on the endogenous DNA preservation and contamination estimates, we selected 25 libraries from the remaining 11 specimens for further SNP enrichment capture using the 1240k array[7] for population genetics analyses, and 17 libraries for capture with the Archaic Admixture array, specifically Panel 4[8], to investigate Neandertal and Denisovan ancestry. Libraries were captured following the capture protocol presented in Slon et al., 2017[4] and Zavala et al., 2022[23]. The libraries captured with the 1240k array were sequenced on a HiSeq4000 or a NextSeq500 platform and mapped to the hs37d5 reference genome with the ancient parameters, and the ones captured with the Archaic Admixture array were sequenced on either a NextSeq500 or a HiSeq4000 platform in the Ancient DNA Core Unit facility at the MPI-EVA in Leipzig, and mapped to the *hg19* reference genome as detailed in the previous paragraph.

Coverage and contamination estimates in the capture data

1240k captures

We performed demultiplexing and mapping as detailed in the previous section, filtered all data for mapping quality of 25 and minimum length cutoff of 30 base pairs, and kept the sequences overlapping the positions on the 1240k SNP array. We obtained between 0.02x and 0.96x coverage for the 25 libraries we captured using the 1240k SNP array. For two of those libraries, Lib.H.3516 and Lib.H.3517, belonging to specimens RNI080 and RNI081, respectively, we found the present-day human contamination to be greater than 50% using AuthentiCT[22], hence we removed those from our dataset for downstream analyses (Table S1.3).

After merging the data from all libraries per specimen, we obtained between 0.02x and 1.86x average coverage for ten specimens (Supplementary Table 3.1). We included two high coverage shotgun genomes we sequenced for this study after subsetting them to the 1240k SNP sites in the dataset. For this dataset, in addition to AuthentiCT[22], we were able to use two other contamination estimation methods, hapCon[24] and hapCon_ROH[25], on the merged data per specimen. This was not possible for the shallow shotgun sequencing, i.e. the screening data, or even on the library level since these contamination estimate methods require a minimum of at least 0.1x coverage[24]. After observing a very low number of SNPs and relatively high contamination levels yielded by RNI084 and RNI086, we decided to exclude these two genomes from our downstream analyses (Supplementary Tables 3.1 and 3.2).

Supplementary Table 3.1: Obtained average coverage per specimen for data produced with the 1240k capture set for 10 specimens, and two high coverage shotgun genomes subsetted to 1240k capture array sites on autosomes. Coverages per chromosome can be found in Table S1.7.

Specimen ID	Archaeological ID	Type of data	Number of SNPs	Proportion Covered	Average depth	Number of SNPs (deam)	Genetic Sex
ZKU002	Zlatý kůň skull	Shotgun	1,142,037	0.99	20.04	982,083	F
RNI013	R10873	Shotgun	1,140,912	0.99	23.14	1,041,626	M
		1240k	181,469	0.16	0.34	49,620	
RNI012	R10879	1240k	430,612	0.37	0.96	103,397	F
RNI010	R10874	1240k	184,085	0.16	0.34	61,987	M
RNI006	R10876	1240k	169,719	0.15	0.30	46,823	F
RNI004	R10875	1240k	660,264	0.57	1.86	220,011	F
RNI080	R10396	1240k	391,045	0.34	0.80	114,037	F
RNI081	R10355	1240k	239,366	0.21	0.43	68,547	F
RNI084	16/116-159416	1240k	27,388	0.02	0.04	6,641	F
RNI085	16/116-159327	1240k	659,112	0.57	1.72	240,002	F
RNI086	16/116-159253	1240k	15,407	0.01	0.02	3,168	M
RNI087	16/116-159199	1240k	544,676	0.47	1.31	229,054	M

Supplementary Table 3.2: Contamination estimates per specimen for data produced with 1240k capture set for 10 specimens, and two high coverage shotgun genomes subsetted to 1240k capture array sites. Column names indicate the method used for estimating the percentage of present-day contamination. CI (confidence intervals) are indicated in parentheses for the point estimates. HapCon can only be used for males as it requires a haploid X chromosome, females are denoted with an “F”.

Specimen ID	AuthentiCT all sequences	hapCon_ROH all sequences	hapCon (all sequences, for males)	hapCon_ROH deaminated sequences
ZKU002	0.10 (-0.11056, 1.3056)	1.7114 (1.6269 - 1.7959)	F	0.9383 (0.7225 - 1.154)
RNI013	1.09 (1.0856 - 2.1072)	0.9636 (0.8818 - 1.0455)	1.6992 (1.5121 - 1.8864)	0.8145 (0.6174 - 1.0116)
RNI012	4.44 (3.1171 - 5.7639)	3.7145 (3.0323 - 4.3968)	F	0 (-0.8559 - 0.8559)
RNI010	7.41 (6.8418 - 7.9718)	13.5487 (11.1363 - 15.9611)	19.3284 (16.8233 - 21.8335)	0 (-4.5505 - 4.5505)
RNI006	15.13 (14.5095 - 15.1339)	16.9299 (14.3337 - 19.5262)	F	4.9258 (-5.6462 - 15.4977)
RNI004	4.6677 (3.7803 - 5.5551)	1.6431 (1.4195 - 1.8667)	F	0.31195 (-0.2413 - 0.8652)
RNI080	12.23 (11.4305 - 13.0365)	9.265 (8.2728 - 10.2572)	F	1.76275 (-0.6333 - 4.1588)
RNI081	7.07 (6.2575 - 7.8735)	0.9877 (-0.0232 - 1.9986)	F	0 (-1.6494 - 1.6494)
RNI084	17.90 (16.7683 - 19.0279)	too low coverage	F	too low coverage
RNI085	7.38 (6.5674 - 8.187)	3.2731 (2.7221 - 3.8242)	F	1.0857 (-0.0664 - 2.2378)
RNI086	25.35 (24.0611 - 26.6407)	too low coverage	too low coverage	too low coverage
RNI087	0.10 (-0.05686 - 0.7686)	2.6351 (2.0092 - 3.2609)	2.6351 (2.0092 - 3.2609)	1.5423 (0.2845 - 2.8001)

We found that hapCon_ROH yielded lower contamination estimates in general, with the exception of RNI010 and RNI087. Furthermore, we detected minimal contamination in the deaminated sequences of RNI006 (albeit with very large uncertainty on the point estimate), RNI080, RNI085, and RNI087 using hapCon_ROH, which is not possible to detect with any other contamination estimation method to date. As hapCon_ROH uses the runs of homozygosity in the genomes to be able to estimate the overall contamination, it is not suitable for individuals with less than 25 cM total ROH[25].

Archaic Admixture captures

We enriched 17 libraries from nine specimens for the ~1.7 million SNPs on the Archaic Admixture capture array[8], which are the positions where at least one archaic genome differs from all or nearly all Yoruba individuals in the 1000 Genomes Project dataset, in order to get more resolution on the Neandertal segments in our low coverage genomes. We did not capture any library belonging to one of the specimens, RNI012, as we discovered that there are other specimens in our study that represent the individual that RNI012 comes from (see Supplementary Information 6). We followed the same demultiplexing, mapping (albeit to *hg19* reference genome here) and filtering methodology explained above for the 1240k captures, and subsetted the sequences to the targeted positions on the Archaic Admixture array. We obtained an average coverage of between 0.02x and 1.79x from nine specimens, and similar to the 1240k capture data, we had to remove two of the specimens, RNI084 and RNI086, from the analysis due to present-day contamination levels larger than 20% along with low genome coverage (Supplementary Table 3.3, Table S1.4). As we only use the data obtained from the Archaic Admixture captures for the inference of the archaic ancestry, and do not restrict the sequences to the ones with ancient DNA damage (i.e. C to T substitutions), we did not use ROH based contamination estimation methods for these data, and estimated the amount of present-day human contamination by using AuthentiCT only.

Supplementary Table 3.3: Average depth estimation in all sequences, and the ones with terminal C to T substitutions, in the data produced with the Archaic Admixture captures from each specimen. Contamination is estimated using AuthentiCT[22], using all sequences, and intervals represent minimum and maximum estimates using 1.96 standard errors of the mean.

Specimen ID	Archaeological ID	Average depth (all)	Average depth (deam)	Contamination % (AuthentiCT)
RNI010	R10874	0.14	0.04	13.3 (12.8-13.9)
RNI006	R10876	0.18	0.05	19.2 (18.6 - 19.8)
RNI004	R10875	1.08	0.26	8.4 (7.6 - 9.1)
RNI080	R10396	0.45	0.11	13 (12.1 - 13.9)
RNI081	R10355	0.32	0.09	9.3 (8.5 - 10.1)
RNI084	16/116-159416	0.04	0.01	23.4 (22.3 - 24.3)
RNI085	16/116-159327	1.79	0.49	10.9 (10.1 - 11.7)

RNI086	16/116-159253	0.02	0.00	31.2 (30.0 - 32.5)
RNI087	16/116-159199	1.38	0.48	0.7 (0.5 - 1.5)

Y chromosome capture

We determined that four of the Ranis specimens, RNI010, RNI013, RNI086 and RNI087, originate from genetically-male individuals based on the ratio of sequences aligning to the X chromosome versus the autosomes (Supplementary Table 3.1, Table S1.7). As we had already obtained a high quality shotgun genome for RNI013 (Supplementary Information 4), we did not need to perform additional SNP capture for the Y chromosome of this individual. The library from RNI086 was highly contaminated and had very low aDNA yield, hence we did not capture the Y chromosome of this specimen either. Libraries belonging to the two remaining specimens, RNI010 and RNI087, were suitable for further capture, and we performed both complete Y chromosome capture[9] and YMCA capture as described in Rohrlach et al., 2021[10] (Supplementary Table 3.4).

Supplementary Table 3.4: Average depth of coverage on the mappable region of the Y chromosome, and the number of SNPs on the mappable region and ISOGG positions for each specimen.

Specimen ID	Archaeological ID	Coverage on Mappable Y Region	Number of SNPs on Mappable Y Region	Number of SNPs on ISOGG
RNI010	R10874	0.454824	2892113	20328
RNI013	R10873	10.0081	9646971	72191
RNI087	16/116-159199	1.86514	6637846	48212

Supplementary Information 4

Data Processing & General Filters for High Coverage Genomes

Arev Pelin Sümer*, Kay Prüfer

Initial processing

Three libraries from ZKU002 and four libraries from RNI013 were used to obtain high coverage genomes for Zlatý kůň and Ranis13 respectively.

Sequences from two libraries, ZKU002.A0101 (double-stranded, half-UDG treated) and ZKU002.A0102 (single-stranded, no UDG treatment), were generated for the previous study to obtain a low-coverage genome of Zlatý kůň[3]. In this study, ZKU002.A0102 was further sequenced on two full HiSeq 4000 runs, using 76bp single-read sequencing and on two lanes of a Novaseq 6000 S4-200 flow-cell using 2x75bp paired-end sequencing (the latter carried out at SciLifeLab in Stockholm, Sweden). A second library, ZKU002.A0103, was produced in this study from the same DNA extract as the two previous libraries. The ZKU002.A0103 library was sequenced at SciLifeLab on a full Novaseq 6000 S4-200 flow-cell using 2x75bp paired-end sequencing.

For Ranis13, four single stranded libraries (RNI013.A0101, RNI013.A0102, RNI013.A0103, and RNI013.A0104) were prepared from the same lysate and pooled together for deeper sequencing. Pooled libraries were sequenced at SciLifeLab following the same set-up as for the ZKU002.A0103 library described above.

We removed the adaptors and merged forward and reverse reads using leeHom[26]. Sequences were assigned to the libraries based on the index sequences, allowing for up to one mismatch in each index. When single libraries were sequenced on a full lane without pooling, all sequences were assigned to a given library, as long as no evidence for extraneous index combinations were found. Assigned sequences were mapped to the human reference genome (hs37d5) with BWA[27] (parameters: -n 0.01 -o 2 -l 16500), and duplicates were removed using bam-rmdup (https://github.com/mpieva/biohazard-tools/) separately for each library.

Spurious alignments, length and quality filters

Due to degradation of DNA after an organism's death, endogenous aDNA molecules are often short. However, at short molecule lengths their alignment to the reference genome becomes difficult, as random microbial DNA sequences present in the data may align to the human reference genome just by chance. These spurious alignments particularly increase for short DNA fragments, and thus a minimum length of 35 base pairs is frequently used for downstream analyses. Here, we used SpAl version 2 (https://figshare.com/articles/spal/7140026[28]) to estimate optimal length cutoffs that maintain a low fraction of spurious alignments.

The program SpAl (https://bioinf.eva.mpg.de/SpAl/) works by mapping all DNA sequences to a modified reference genome, which is created by introducing around 18 million mutations that are not expected to be found in modern humans or archaic humans (Neandertals and Denisovans)[28]. We randomly sampled 1M sequences from each library and mapped them to this modified reference genome and ran SpAl to determine spurious alignment rates. A maximum of 1% of spurious alignments were inferred at length cutoffs of 26, 27 and 27 bp for ZKU002.A0101, ZKU002.A0102 and ZKU002.A0103, respectively. For all four libraries of Ranis13, the length cutoff for at most 1% spurious alignments was 28 bp. We filtered each library accordingly for minimum length, and mapping quality of 25, using samtools[29] (version 1.3.1). Following de Filippo et al., 2018[28] we also removed alignments with indels for sequences shorter than 35 base pairs.

In addition to determining spurious alignment rates, we applied a genome mappability filter. This included the genome mappability filter map35_100% as described in Prüfer et al., 2014, Supplementary Information 5b, page 35[30] and MapL[28] to ensure mappability of sequences shorter than 35 base pairs. As expected for ancient DNA, we observed elevated C to T substitution patterns at the ends of the sequences for all libraries (Supplementary Figure 4.1).

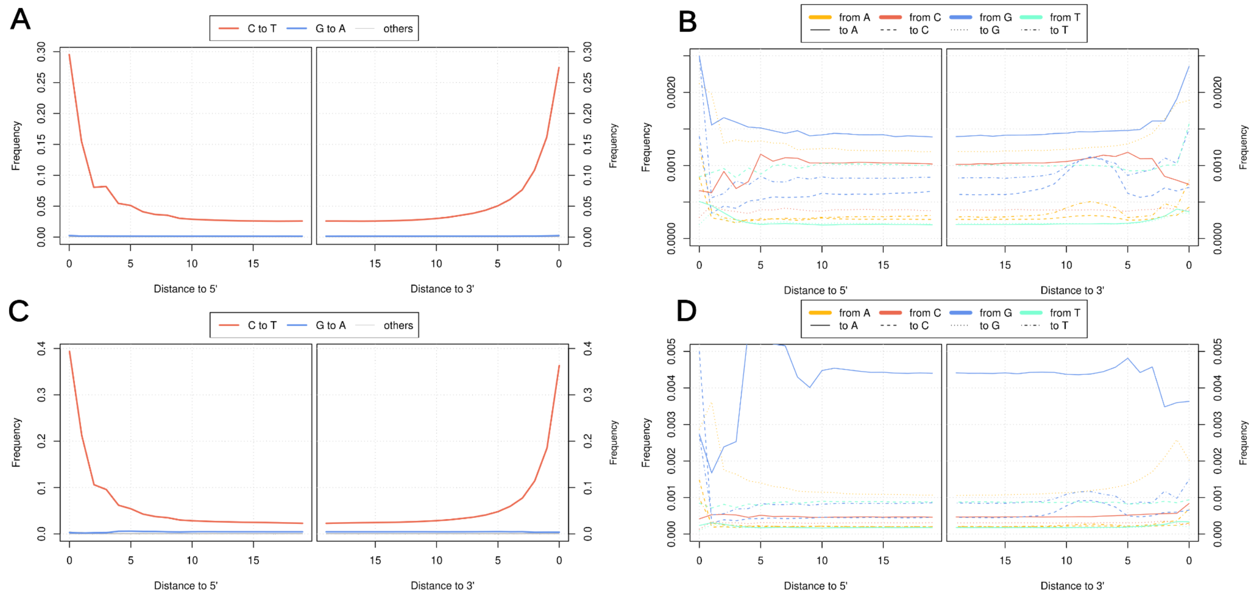

Supplementary Figure 4.1: A. C to T and G to A substitution patterns and **B.** other, less common substitutions for library ZKU002.A0103. The pattern is similar in the other non-UDG treated library of Zlatý kůň (ZKU002.A0102). **C.** C to T and G to A substitution patterns and **D.** other substitutions for the RNI013.A0103 library of Ranis13. Damage patterns are similar in all four libraries of Ranis13 (RNI013.A0101, RNI013.A0102, RNI013.A0103, and RNI013.A0104). Frequency of substitutions is shown on the y-axis.

Contamination and coverage

Genomic coverage was determined from the sequence data of each library within the 35-mer mappability regions by filtering for fragments of length 35 or larger. We found the average depth of coverage on the autosomes to be approximately 22x for Zlatý kůň and 25.5x for Ranis13 (Supplementary Table 4.1). For Zlatý kůň, we find the average coverage on the X chromosome to be similar to the average coverage on the autosomes, indicating (as reported previously), that this individual was genetically female. The X chromosome of Ranis13 has around half the coverage of that of the autosomes, indicating Ranis13 was a genetically male individual (Supplementary Table 4.2).

Supplementary Table 4.1: Average depth of coverage and contamination proportion estimates for each library of Zlatý kůň and Ranis13, after applying all the filters described above, resulting in around 22x and 25.5x genomic coverage. Confidence intervals for AuthentiCT and hapCon_ROH correspond to 1.96 standard errors.

Specimen	Library	Coverage	Contamination (AuthentiCT)	Contamination (hapCon_ROH)

ZKU002	A0101	0.5x	method not applicable to double stranded UDG libraries	0.017985 (0.014342 - 0.021628)
ZKU002	A0201	9.7721x	0.028652 (0.017156, 0.040148)	0.016662 (0.015434 - 0.017889)
ZKU002	A0301	12.3506x	0.028582 (0.013242, 0.043922)	0.01605 (0.014961 - 0.017139)
RNI013	A0101	5.5215x	0.040225 (0.027693, 0.052757)	0.00637 (0.004625 - 0.008114)
RNI013	A0201	7.2769x	0.029698 (0.01688, 0.042516)	0.004434 (0.002867 - 0.006002)
RNI013	A0301	6.3088x	0.046025 (0.033413, 0.058637)	0.005466 (0.003945 - 0.006986)
RNI013	A0401	6.3985x	0.023653 (0.010017, 0.037289)	0.004557 (0.003067 - 0.006046)

We estimated present-day human contamination for all single-stranded libraries using AuthentiCT[22]. We were not able to calculate the contamination proportion in library A0101 of Zlatý kůň using this method. This is due to the half-UDG treatment used while preparing this library that alters the C to T substitution patterns, which is the primary source of information for the HMM underlying AuthentiCT. Mean estimates of contamination obtained using AuthentiCT for all libraries varied between 1% and 5.9%. In addition, we used a contamination estimation method based on ROH, using hapCon_ROH (Supplementary Information 2)[25]. Overall, this method yielded lower estimates, which varied between 0.3% and 2.2%.

Supplementary Table 4.2: Number of base pairs aligned to each chromosome of the high coverage Zlatý kůň and Ranis13 genomes, after filtering for the minimum length reported by SpAl, mapping quality, alignability filter, and short indels. The size of each chromosome and ratio of the genome that aligned to a specific chromosome are also reported.

**Chromosome**		**Ranis13**		**Zlatý kůň**	
**Name**	**Size**	**Bases**	**Ratio**	**Bases**	**Ratio**
Chr1	225,280,621	4,719,179,986	20.95	4,276,709,625	18.98
Chr2	238,204,518	5,296,004,102	22.23	4,652,075,784	19.53
Chr3	194,797,135	4,393,157,394	22.55	3,864,957,722	19.84
Chr4	187,661,676	4,286,266,191	22.84	3,677,461,611	19.60
Chr5	177,695,260	3,988,668,905	22.45	3,465,205,290	19.50
Chr6	167,395,066	3,668,221,711	21.91	3,217,228,781	19.22
Chr7	155,353,663	3,243,058,719	20.88	2,903,591,726	18.69
Chr8	142,888,922	3,246,935,898	22.72	2,815,895,285	19.71
Chr9	120,143,431	2,418,908,636	20.13	2,136,969,176	17.79
Chr10	131,314,738	2,841,824,289	21.64	2,517,181,304	19.17
Chr11	131,129,516	2,855,835,372	21.78	2,527,904,324	19.28

Chr12	130,481,393	2,818,048,937	21.60	2,526,764,024	19.36
Chr13	95,589,878	2,205,639,327	23.07	1,919,430,397	20.08
Chr14	88,289,540	1,930,795,365	21.87	1,717,416,664	19.45
Chr15	81,694,766	1,682,782,041	20.60	1,518,408,418	18.59
Chr16	78,884,753	1,608,067,654	20.39	1,427,666,532	18.10
Chr17	77,795,210	1,540,137,239	19.80	1,396,979,196	17.96
Chr18	74,657,229	1,737,021,046	23.27	1,497,298,696	20.06
Chr19	55,808,983	1,065,372,608	19.09	952,126,141	17.06
Chr20	59,505,520	1,308,578,623	21.99	1,178,640,751	19.81
Chr21	35,106,642	764,952,799	21.79	670,147,899	19.09
Chr22	34,894,545	677,320,795	19.41	614,788,630	17.62
ChrX	151,100,560	1,620,588,369	10.73	2,707,197,505	17.92
ChrY	22,984,529	131,668,464	5.73	2,980,509	0.13

A previous metagenomic analysis of shotgun sequences from Zlatý kůň indicated an unusually high rate of mapping to cattle (*Bos taurus*) that may be explained by a preservation treatment of the Zlatý kůň skull with bone glue. Some of these cattle sequences may mismap to the human genome. To investigate how high the proportion of such mismapping sequences is, we mapped 226 million sequences from library ZKU002.A0102 to the cow genome (*bosTau8*) using BWA[27] with the same parameters as before. Of these, 702,011 sequences map to the cow genome with a mapping quality of 25 and a minimum length of 35, whereas 11,066,479 mapped to the human genome with identical cutoffs. We used a minimum length of 35 bp here as the majority of the analyses are done using this cutoff, as detailed in the next chapters. Given the evolutionary similarity between the two reference genomes, many of the matches to the cow genome may fall in conserved regions and do not represent true cattle sequences. To further differentiate potentially true cattle sequences from matches to conserved regions, we further required that sequences mapped with a smaller number of mismatches to the cow genome compared to the human genome. A total of 5,732 sequences matched this criteria, leading to an estimated proportion of 0.00052 (exact binomial confidence interval 0.00050-0.00053) of misaligned cow sequences among human aligning sequences. If, conservatively, all sequences with an equal or better alignment to the cow genome are considered cow sequences, then the estimated proportion

is 0.00261 (0.00258-0.00264). Note that the latter estimate would include *bona fide* human sequences mapping to highly conserved regions in the cow genome and is thus an overestimate.

Supplementary Information 5

Genotyping of the High Coverage Genomes

Arev Pelin Sümer*, Kay Prüfer

We used the software snpAD[31,32] for genotype calling of the Zlatý kůň and Ranis13 high coverage genomes to account for false heterozygous calls which occur due to ancient DNA damage, sequencing or alignment errors. Prior to genotype calling, we filtered the sequences on all autosomes as described in the previous chapter, and, in addition, filtered for a base quality of 30, merged the bam files from different libraries per specimen, and re-aligned indels using GATK[33] (version 1.3-14). We produced the genotypes for a sequence length cutoff of 35 similar to previous studies. Error profiles (probabilities of base exchanges) and genotype frequencies were estimated and used to call the most likely genotype at each site, independently for each chromosome. As a quality control, we estimated the transition transversion ratios (TS/TV) from the called genotypes, which are around 2 in ancient and present-day modern humans.

Additional filters on genotypes

Following previous approaches[30,31,34], we applied additional filters to remove sites that fall within the 2.5% extremes of the GC-corrected coverage distribution, removed tandem repeats[35] and removed sites called as indels. We also filtered for sites with a minimum depth of 10x, and maximum depth of 50x.

Supplementary Table 5.1: Transition to transversion ratios and coverage estimates for each chromosome of Zlatý kůň, filtered for minimum sequence length of 35 base pairs.

Chromosome	TS/TV	Number of sites	Mean depth
Chr1	2.11	142,107,646	18.8425
Chr2	2.02	156,705,831	18.9461
Chr3	2	129,495,278	18.9533
Chr4	1.98	122,126,659	19.0037
Chr5	1.99	116,011,726	18.9693
Chr6	2.05	107,252,997	19.035
Chr7	2.01	94,664,866	18.9105
Chr8	1.86	94,670,239	18.9351

Chr9	1.92	71,604,782	18.8578
Chr10	2.12	83,699,666	18.9015
Chr11	2.01	84,366,292	18.7431
Chr12	2.06	83,348,240	18.8683
Chr13	2.03	64,454,148	19.0233
Chr14	2.05	56,927,150	18.9074
Chr15	2.01	49,913,048	18.9966
Chr16	1.8	46,578,353	18.784
Chr17	2.26	45,037,264	18.7377
Chr18	2.09	51,143,949	18.9413
Chr19	2.15	27,193,645	18.4302
Chr20	2.19	40,022,598	18.7599
Chr21	2.07	22,106,456	18.9243
Chr22	2.31	20,287,318	18.4273

Supplementary Table 5.2: Transition to transversion ratios and coverage estimates for each chromosome of Ranis13, filtered for minimum sequence length of 35 base pairs.

Chromosome	TS/TV	Number of sites	Mean depth
Chr1	2.08	139,970,403	21.0857
Chr2	1.99	155,601,656	21.7905
Chr3	1.96	128,842,571	21.7435
Chr4	1.96	122,329,064	22.3181
Chr5	1.94	115.770,653	22.0167
Chr6	2.03	106,864,805	21.8807
Chr7	1.97	93,551,496	21.4165
Chr8	1.83	94,308,645	22.0459
Chr9	1.9	70,747,160	21.6374
Chr10	2.07	82,729,404	21.655
Chr11	1.97	83,266,188	21.4348
Chr12	2.03	82,516,643	21.32
Chr13	2.01	64,330,721	22.0318
Chr14	2.01	56,269,714	21.5453
Chr15	1.99	49,055,807	21.3146
Chr16	1.75	45,642,370	21.6279

Chr17	2.21	43,678,995	21.2004
Chr18	2.07	51,013,668	22.1704
Chr19	2.16	26,056,471	21.2523
Chr20	2.19	39,300,711	21.1635
Chr21	2.04	21,886,528	21.8775
Chr22	2.21	19,648,699	20.819

Supplementary Information 6

Biological Kinship

Alba Bossoms Mesa*, Arev Pelin Sümer

Here, we discern the minimum number of distinct individuals present in the Ranis collection and search for biological kinship among them. Our results establish genetic connections between specimens from the 1932-1938 and 2016-2022 excavations.

We use the software KIN[36] to infer biological relatedness among low-coverage and high-coverage samples. The method was developed for ancient DNA and takes consanguinity and contamination into account. For this, estimates of contamination need to be provided and we use estimates from AuthentiCT[22] and hapCon_ROH[25] as described in Supplementary Information 3 (see Supplementary Table 3.2). We also inferred biological kinship using only deaminated sequences to gain further confidence in the inferences. Since contamination cannot be estimated solely from deaminated sequences with AuthentiCT, we run KIN with an estimate of 0% contamination in addition to the hapCon_ROH estimate when estimating kinship from those data.

As required by KIN, we restricted our analyses to on-target sites from the 1240K capture array[7]. Two specimens, RNI084 and RNI086, were excluded from our main kinship analysis since they fall below the recommended coverage of 0.05x average depth for KIN. In addition, contamination could not be estimated using hapCon_ROH on neither all sequences nor deaminated sequences for both samples. However, see below for an attempt at detecting identity with other samples.

We first ran KIN on the best samples available, both in terms of higher coverage and lower contamination, i.e. Zlatý kůň, RNI004, RNI012, RNI013, and RNI087. This allowed us to define “noisy” windows of the genome, as well as to infer the expected proportion of pairwise differences in the population (p0 = 0.26 for all sequences, and p0 = 0.28 for deaminated-sequences only). Following previous approaches[36], the latter proportions of pairwise mismatches are used when later including samples with higher contamination and lower coverage. KIN consistently estimates no kinship for all pairs except for RNI004 and RNI012.

The latter two samples show a second degree relationship (e.g. grandparent and grandchild, half siblings or aunt/uncle and niece/nephew) in three of four analyses (Supplementary Figure 6.1). The last analysis using only deaminated sequences and the hapCon_ROH contamination estimate also detected kinship between both samples but was unable to distinguish between second and third degree relatedness. We conclude that RNI004 and RNI012 are likely second degree related. A close relationship of up to 5th degree is also supported by IBD sharing as described in Supplementary Information 8.

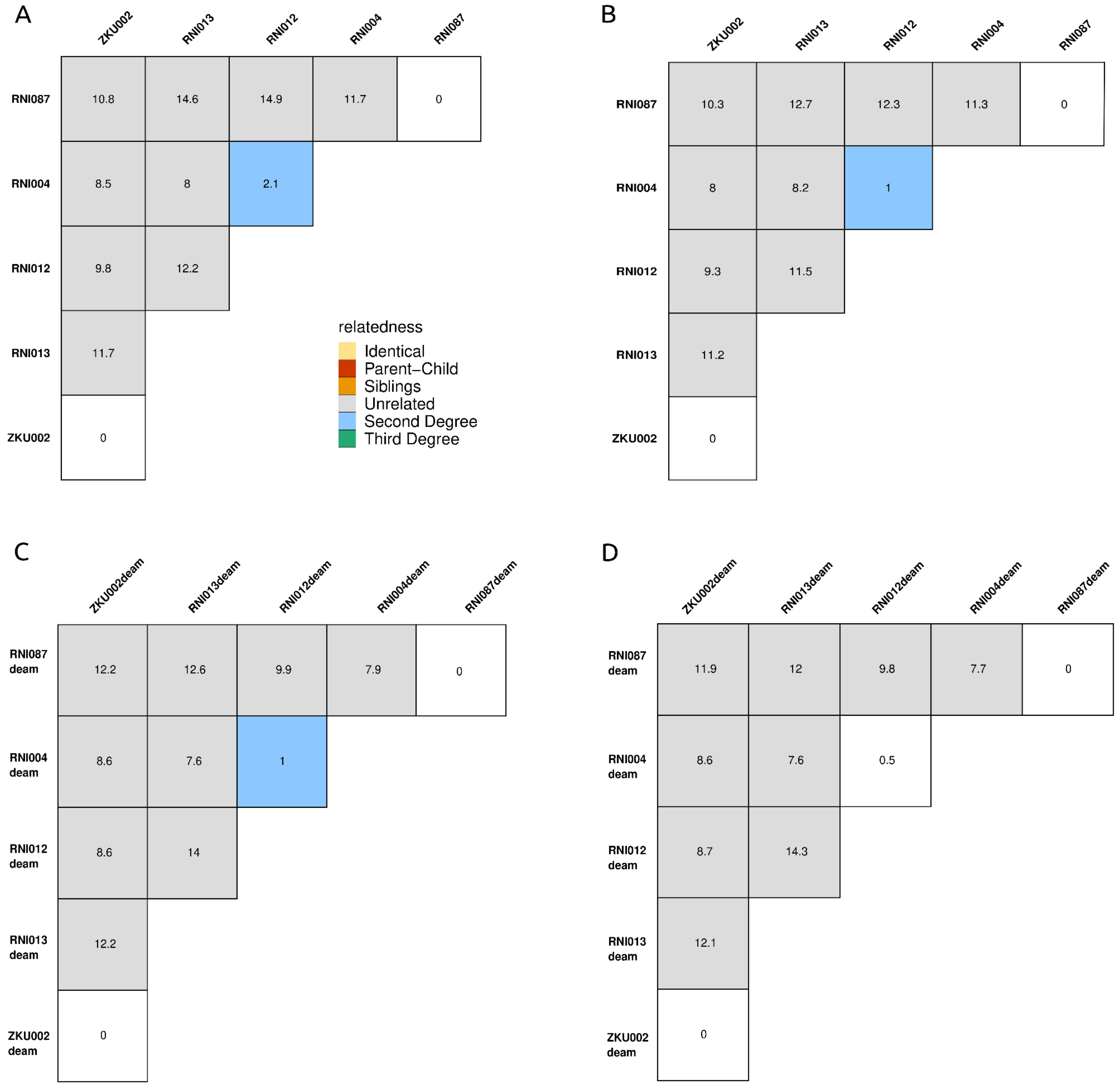

Supplementary Figure 6.1: KIN results for the best set of samples. **A)** All sequences with AuthentiCT estimates, **B)** all sequences with hapCon_ROH estimates, **C)** deaminated-sequences only assuming no contamination, and **D)** deaminated-sequences only with hapCon_ROH estimates. Relatedness is colour-coded. Numbers represent log-likelihoods, and the cells that are not coloured are with a log-likelihood value less than the threshold we use to trust an inference, which is one.

Next, we included low-coverage samples with higher contamination rates in our analyses, using the pairwise difference parameter (p0) estimated above. In addition to the second degree relationship between RNI004 and RNI012, the extended dataset revealed a consistent parent-offspring relationship between RNI004 and RNI006 (Supplementary Figure 6.2). Both individuals are female and RNI006 is a juvenile less than five years old based on its size. This indicates that sample RNI004 belonged to the mother and RNI006 to her daughter.

A cluster of close pairwise relationships links samples RNI012, RNI080, RNI081, and RNI085, although the type of inferred kinship differs between the four types of filters and contamination estimates (Supplementary Figure 6.2 and 6.3). To further clarify the relationship of these four samples, we consult the inferred Neandertal ancestry segments in all four specimens (see Supplementary Information 14 and find that all four samples produce matching Neandertal ancestry patterns. We conclude that RNI012, RNI080, RNI081 and RNI085 belonged to the same individual that we will refer to as Ranis12. We will analogously refer to the individuals represented by a single sample with a designator RanisN where N is the sample number (Supplementary Table 6.1). Three of these specimens, RNI012, RNI080, and RNI081 were discovered during the old excavations (in 1934 and 1935), and the last one, RNI085, in the 2020 excavation season. All four specimens were excavated from different squares, scattered across the site (Extended Figure 1).

Supplementary Table 6.1: Average depth and present-day contamination estimates for individuals for which we obtained low coverage genomes using the 1240k capture array.

Individual ID	Specimen ID(s)	Archaeological ID(s)	Average depth of coverage
Ranis4	RNI004	R10875	1.86
Ranis6	RNI006	R10876	0.30
Ranis10	RNI010	R10874	0.34
Ranis12	RNI012, RNI080, RNI081, RNI085	R10879,R10396, R10355,16/116-159327	3.91

Ranis84	RNI084	16/116-159416	0.04
Ranis86	RNI086	16/116-159253	0.02
Ranis87	RNI087	16/116-159199	1.31

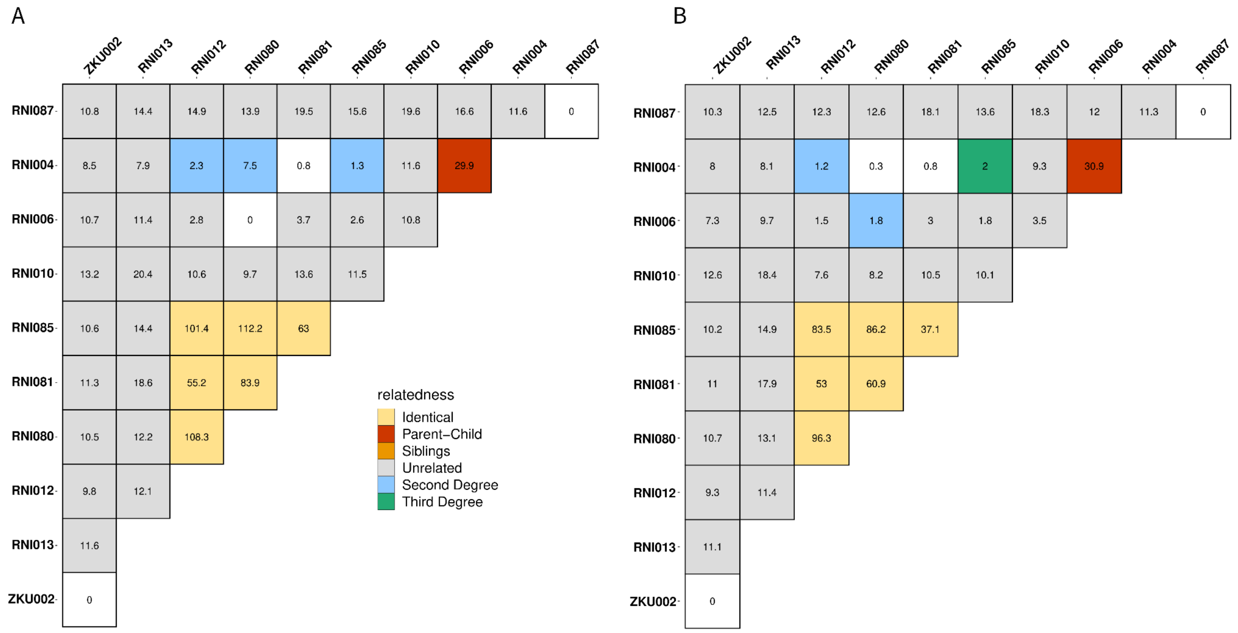

Supplementary Figure 6.2: KIN results for all specimens (unmerged data) using all sequences and **A.** AuthentiCT contamination estimates or **B.** hapCon_ROH contamination estimates.

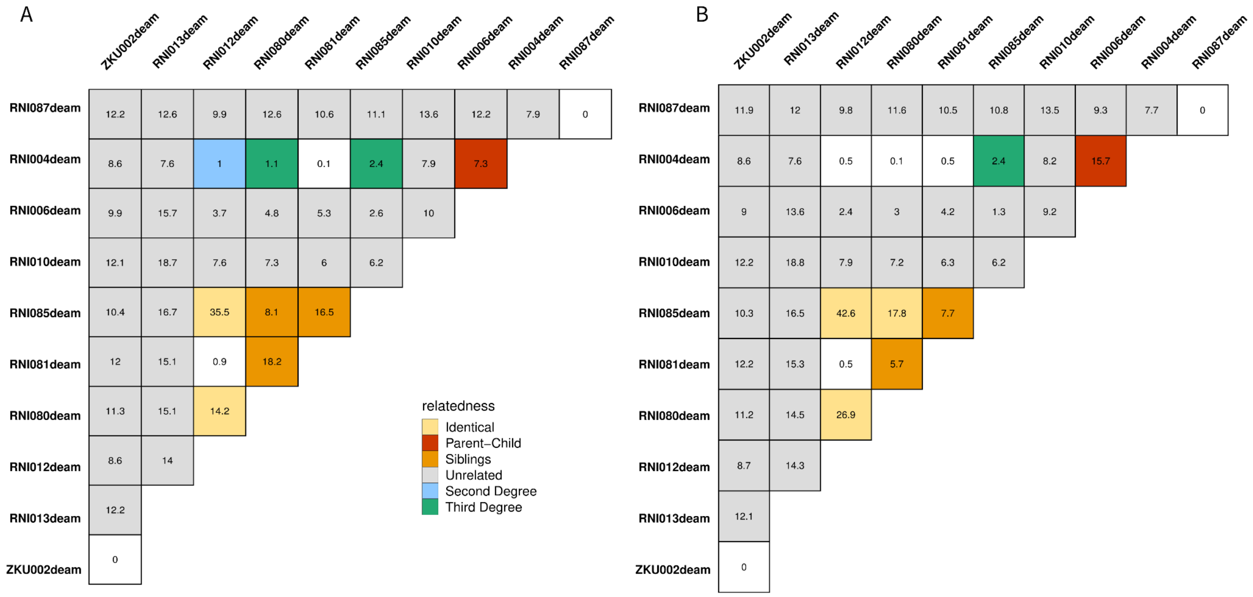

Supplementary Figure 6.3: KIN results for all specimens (unmerged data) using deaminated sequences only and **A.** assuming no contamination or **B.** using hapCon_ROH contamination estimates.

After merging the data for RNI012, RNI080, RNI081, and RNI085 to produce Ranis12, we estimated contamination for the merged data and rerun KIN again using the contamination estimates per individual (Supplementary Figures 6.4, 6.5 and 6.6). The inferred relationship between Ranis12 and Ranis4 varies between second and third degree, as before.

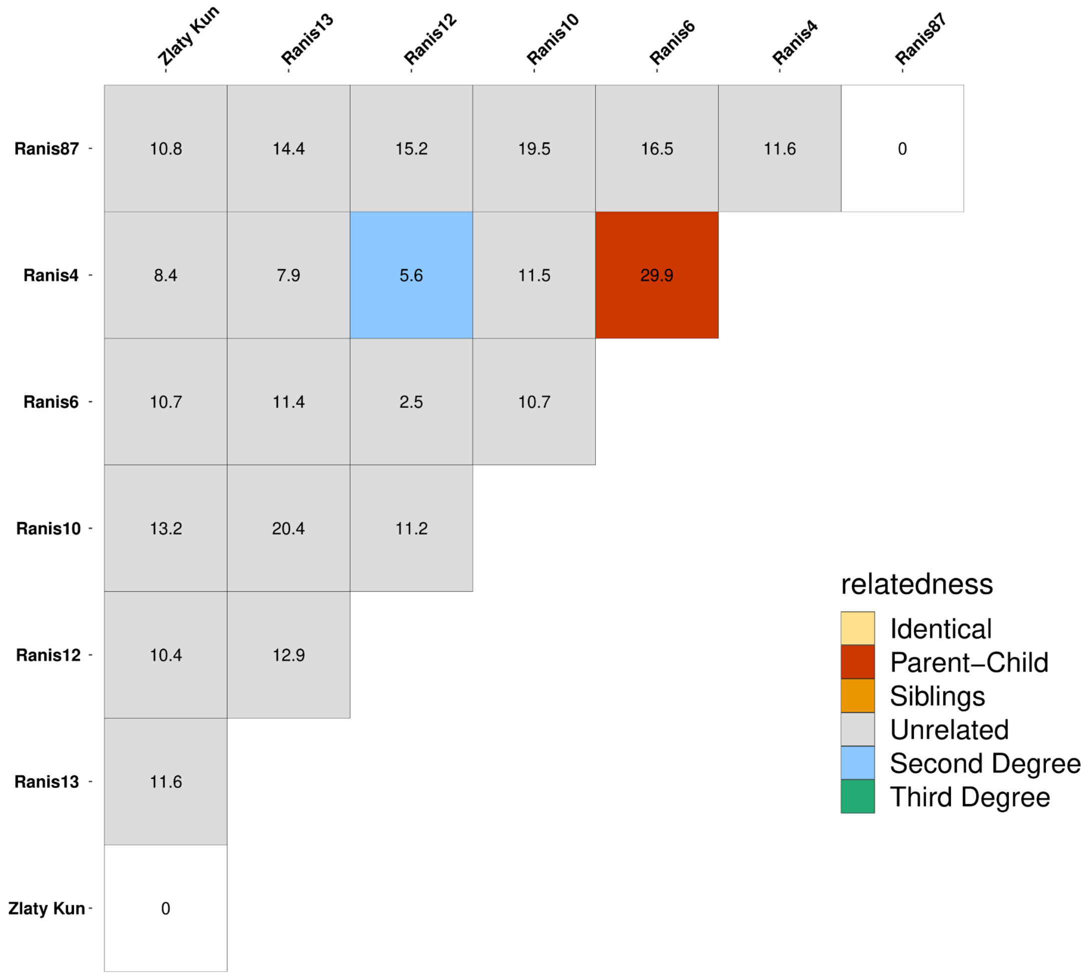

Supplementary Figure 6.4: KIN results for all specimens (merged data) using all sequences and AuthentiCT contamination estimates.

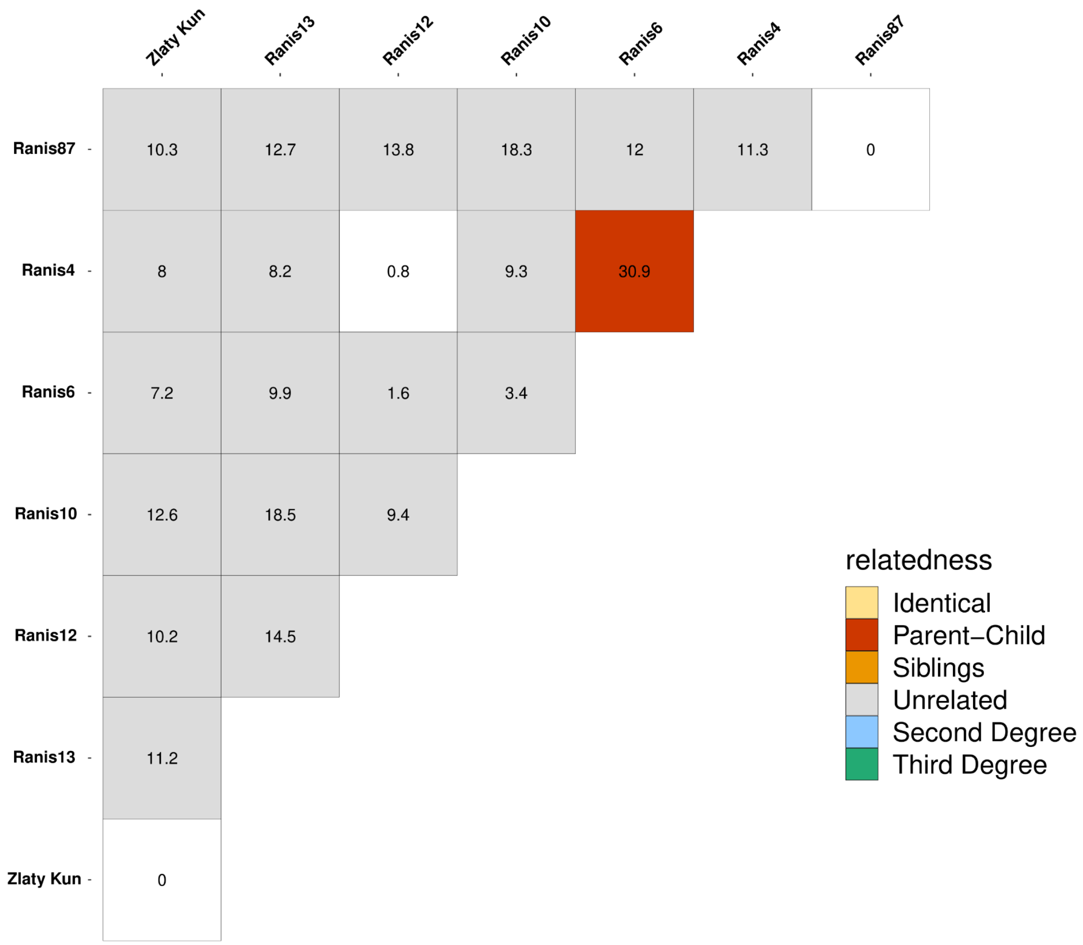

Supplementary Figure 6.5: KIN results for all specimens (merged data) using all sequences and hapCon_ROH contamination estimates.

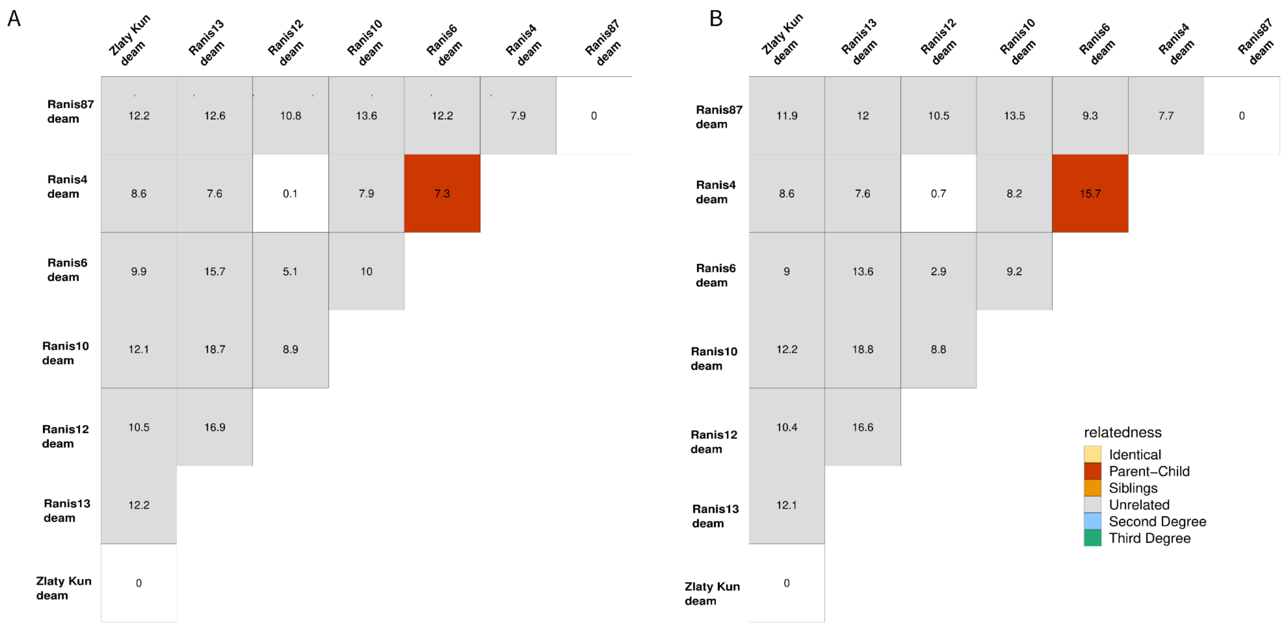

Supplementary Figure 6.6: KIN results for all specimens (merged data) using deaminated sequences only and **A.** assuming no contamination or **B.** using hapCon_ROH contamination estimates.

Mylopotamitaki et al., 2024[6] showed that the mitochondrial genomes reconstructed from specimens RNI080 (R10396), RNI012 (R10879), RNI010 (R10874), and RNI085 (16/116-159327) carried at most five missing positions and were identical to each other, which led to the suggestion that these specimens either come from the same individual, or individuals maternally related to each other. Furthermore, the mtDNA genome of RNI081 (R10355) also showed no differences with these four, albeit having more missing positions. Here, we are able to infer that RNI080, RNI012, RNI085, and RNI081 are indeed originating from the same individual, Ranis12. However, RNI010 showed no close relationship (i.e. more distant than third degree) with Ranis12. These individuals can still be maternally related to each other, but more distantly than third degree, which is the detection limit of KIN. Due to low quality of the Ranis10 genome, we were not able to investigate the IBD sharing with other Ranis individuals, which could have shed additional light on more distant biological kinship relations (Supplementary Information 8).

The mother-daughter pair, represented with Ranis4 and Ranis6, were found to have identical mitochondrial genomes as well, however this link was tentative due to the high number of missing positions in these two genomes (40 and 26, respectively). In addition Ranis84 did not

have any differences to these two individuals on the mitochondrial sequences as well, and carried four missing positions[6].

Although the genomes from specimens RNI084 and RNI086 are too low coverage for confident inference of relatedness, we included them in the tests using all sequences in an attempt to infer identity to other samples. We found no evidence for identity with other specimens to the limits of our resolution (Tables S2.1-S2.4) and provisionally assign the specimens to independent individuals (Ranis84 and Ranis86). We note that some results suggested that Ranis84 was related to Ranis4 and Ranis6, however due to the low coverage, these results could not be further corroborated (Tables S2.1-S2.4). In conclusion, we were able to assign the 13 specimens from the Ranis humans to a total of eight individuals.

Supplementary Information 7

Heterozygosity and Consanguinity

Cesare de Filippo, Vanessa Villalba-Mouco, Arev Pelin Sümer

Heterozygosity estimates for high coverage genomes from the genotype calls

We estimated heterozygosity on the autosomes from the genotype calls for Ranis13 and Zlatý kůň, filtered as described in Supplementary Information 5. We did this by estimating the proportion of the heterozygous sites for each chromosome and included the high coverage ancient modern human genomes of Ust'-Ishim[37], Loschbour[38], Stuttgart (LBK)[38], and four archaic genomes: Vindija Neandertal (Vindija33.19)[31], Chagyrskaya Neandertal (Chagyrskaya8)[34], Altai Neandertal (Denisova5)[30] and Denisova (Denisova3)[21] for comparison. These genomes were processed, genotyped and filtered in a comparable way to the Ranis13 and Zlatý kůň data[31].

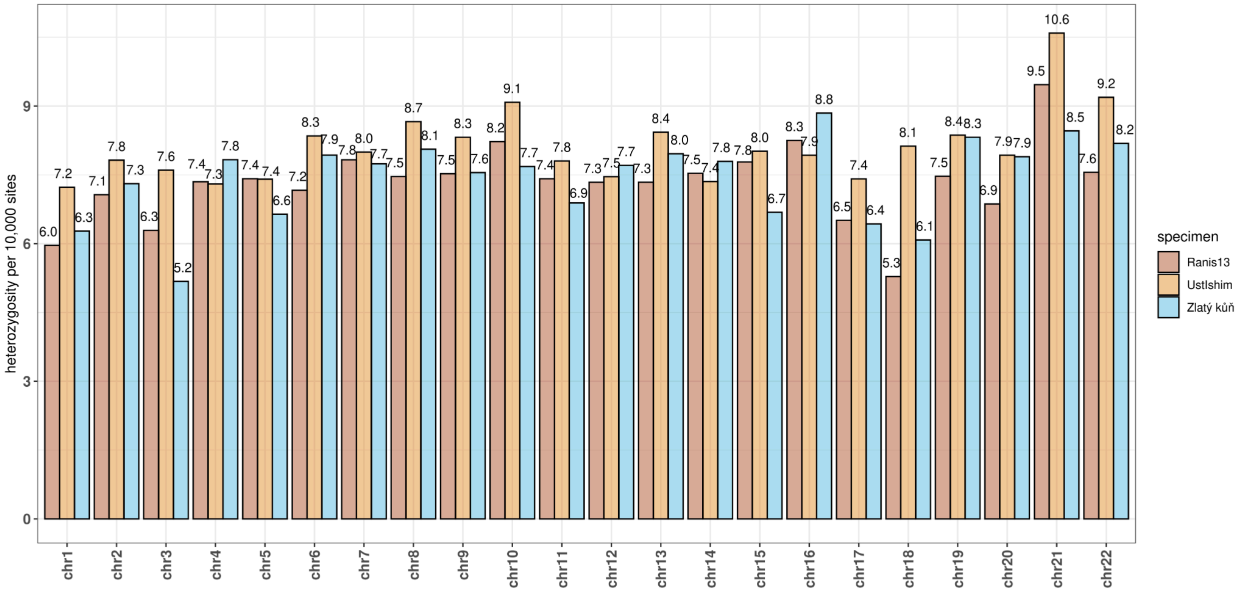

Supplementary Figure 7.1: Heterozygosity estimates per 10,000 sites for three high coverage early modern human genomes per chromosome.

We also calculated heterozygosity in the autosomes of 21 modern individuals from 10 non African and five African populations from the SGDP[39] (Table S3.1). The high coverage genomes of Ranis13 and Zlatý kůň yield similar heterozygosity estimates that are lower than that of Ust'-Ishim[37] (Supplementary Figure 7.2). The heterozygosity estimates for the genomes of

Ranis13 and Zlatý kůň are lower than estimates of present-day Africans, higher than for archaic humans, but within the variation of estimates of present-day Eurasians (Table S3.1), as expected from an individual sampled from the Out of Africa bottleneck. Ranis13 and Zlatý kůň yield similar estimates that are lower than that of Ust'-Ishim[37]. In a more detailed per-chromosome comparison we observe that Ust'-Ishim[37] has higher heterozygosity in most cases (Supplementary Figure 7.1 and Supplementary Table 7.1).

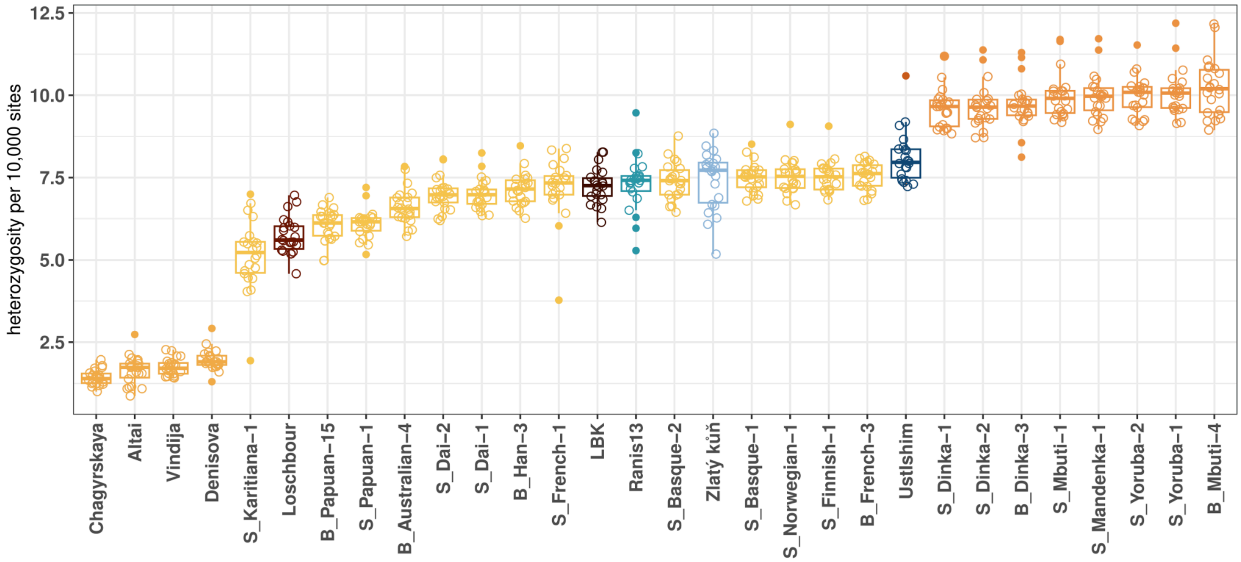

Supplementary Figure 7.2: Heterozygosity estimates (number of heterozygous positions in 10,000 sites) on the autosomes (n=22) for some African and non-African genomes from the SGDP, three high coverage Neandertals, and the high coverage Denisovan genome. Each point represents the heterozygosity estimate on a single chromosome. Archaics are in light and Africans are in dark orange, while non-Africans are in yellow. Five ancient modern humans are in unique colours different from the present-day humans.

Supplementary Table 7.1: Proportion of heterozygous positions for each chromosome of Zlatý kůň and Ranis13, for the files filtered with manifesto and length cutoff of 35 base pairs. Estimates are visualized in Supplementary Figure 7.1 above.

Chromosome	Zlatý kůň	Ranis13
Chr1	0.000627361	0.000596167
Chr2	0.000730783	0.000706676
Chr3	0.00051764	0.000629218
Chr4	0.000783088	0.000735121
Chr5	0.0006642	0.000741646
Chr6	0.00079325	0.000716302
Chr7	0.000774146	0.000782872

Chr8	0.000805991	0.00074632
Chr9	0.000755038	0.000752577
Chr10	0.000768169	0.000822495
Chr11	0.000688769	0.000741547
Chr12	0.000770615	0.000733889
Chr13	0.000796167	0.000733982
Chr14	0.00077948	0.000753335
Chr15	0.000668465	0.000778048
Chr16	0.000884671	0.000825174
Chr17	0.000643019	0.000650744
Chr18	0.000608165	0.000528247
Chr19	0.000832025	0.000746909
Chr20	0.000789609	0.000686601
Chr21	0.00084582	0.000946623
Chr22	0.000818592	0.000755568

Inference of consanguinity

Detection of HBD from genotype calls for two high coverage genomes

We used a previously described method[30,31] to identify homozygous by descent (HBD) regions in ancient genomes. Briefly, the method uses genotype data to detect uninterrupted runs of homozygosity (ROH) and then joins these tracts into larger regions by allowing for a pre-defined proportion of bases in tracts that can contain heterozygous sites. This second step is necessary to accommodate genotyping errors but also to allow for rare *bona fide* cases of *de novo* mutations that may have accumulated in HBD regions. The pre-defined parameter π specifies the proportion of the tract that is not allowed to contain any heterozygous sites for a HBD region to be called.

As before, we analyzed the high-coverage genomes of Ranis13 and Zlatý kůň together with comparison genomes of three ancient modern humans (Ust'-Ishim, Loschbour[38], and Stuttgart (LBK)[38]), three Neandertals and one Denisovan. The parameter π can differ between these genomes since heterozygosity and error rates differ. To choose adequate cutoffs for each genome

we tested a set of different values, $\pi = \{0.8, 0.825, \dots, 0.975, 0.99\}$, and plotted the total length of regions with a length greater than 12 cM assuming a uniform recombination rate of 1.3 cM/Mb. As expected, the total length of 12 cM regions increases with lower values for π as neighboring shorter regions can be collapsed into larger regions. To choose a cutoff on π that allows for comparisons between genomes, we reason that the joining of short regions will plateau as soon as most errors are overcome. A cutoff is chosen by using the value for π with the highest proportion of long HBD segments over the total amount of consanguinity in intermediate and long segments (see later). This maximizes the detection of long segments and minimizes that of shorter ones, as by decreasing π there could be more false positive segments of shorter length (see Supplementary Figure 7.3).

We calculated the amount of consanguinity as the total number sites in HBD segments divided by the total number of sites retained after filtering (see Supplementary Table 8.2). In addition to a constant recombination rate of 1.3 cM/Mb, we also tested the effect of using an African and a European (deCode) recombination map to scale region length. Using these approaches to determine genetic length, we classified regions into long (> 12 cM) and intermediate lengths (4 cM - 12 cM) (Supplementary Tables 7.2 and 7.3). (Note that these length cutoffs match those for the hapROH analysis below, and that different cutoffs were used in the past (2.5 and 10 cM)[30,31,34].)

Zlatý kůň shows a consistently higher fraction of the genome in both intermediate and long HBD segments than Ranis13 does (Supplementary Table 7.2 and Supplementary Figure 7.3) independent of the map of recombination rates used for scaling (Supplementary Table 7.3). Specifically, the amount of consanguinity in intermediate and long HBD segments in Zlatý kůň are ~60% and ~20% higher than Ranis13. Furthermore, Zlatý kůň's fractions of HBD are nearly twice as high as the hunter-gatherer Ust'-Ishim's values for both long and intermediate segments, more than twice as high than the ~8,000-year-old Loschbour's values (which carries only one long region), and more than eight times higher than the ~7,000-year-old farmer Stuttgart (LBK) (with only one long HBD and no intermediate regions) (Tables S3.2-S3.6).

The total amount of consanguinity in Zlatý kůň (7.2%) and Ranis13 (5.2%) is comparable to that found in the ~45,000-year-old Vindija Neandertal (7%). However, a smaller fraction of the

Vindija Neandertal's genome resides in long HBD segments (1.5%). Other Neandertals show a substantially higher HBD fraction while Denisova shows lower fractions (Tables S3.7-S3.10).

Supplementary Table 7.2: Amount of consanguinity in different ancient high-coverage genomes. The table shows the amount of HBD in percentage and their total length in cM, with lengths higher than 4 cM (all) and 12 cM (long). These values are equivalent to those in Supplementary Figure 7.4.

Samples	Amount of HBD [%]		Total length HBD [cM]		Genome used [Mb]
	long	all	long	all	
Denisova	0.5	2.8	31.6	130.9	1649
Altai	12.2	16.7	439.7	600.3	1657
Chagyrskaya	6.1	16.2	219.7	581.8	1636
Vindija	1.5	7.0	50.3	254.4	1654
Ust'-Ishim	1.8	3.7	58.2	133.4	1692
Zlatý kůň	3.1	7.2	109.5	265.1	1638
Ranis13	2.6	5.2	87.8	189.7	1633
Loschbour	0.5	3.1	16.8	133.6	1609
LBK	0.7	0.8	25.5	25.5	1578

Supplementary Table 7.3: Amount of consanguinity as in Supplementary Table 7.2 but using different recombination maps. See also Supplementary Figure 7.4.

Samples	African American map				European (deCode) map			
	Amount of HBD [%]		Total length HBD [cM]		Amount of HBD [%]		Total length HBD [cM]	
	long	all	long	all	long	all	long	all
Denisova	0	2.5	0	103.0	0	2.6	0	111.9
Altai	2.7	15.8	461.0	594.0	12.3	15.4	425.4	554.0
Chagyrskaya	5.2	13.6	253.1	568.6	3.8	12.0	174.0	463.3
Vindija	1.6	7.6	64.7	364.5	0.9	4.1	25.6	104.4
Ust'-Ishim	1.0	2.9	25.1	91.2	1.1	3.1	29.6	95.8
Zlatý kůň	3.4	6.1	107.6	221.6	3.1	6.0	89.1	210.9
Ranis13	1.9	3.5	61.3	149.4	1.7	3.7	39.1	140.8
Loschbour	0.6	3.8	51.3	195.2	0.2	2.2	12.9	84.7
LBK	0.7	0.8	13.0	13.0	0.8	0.8	12.7	12.7

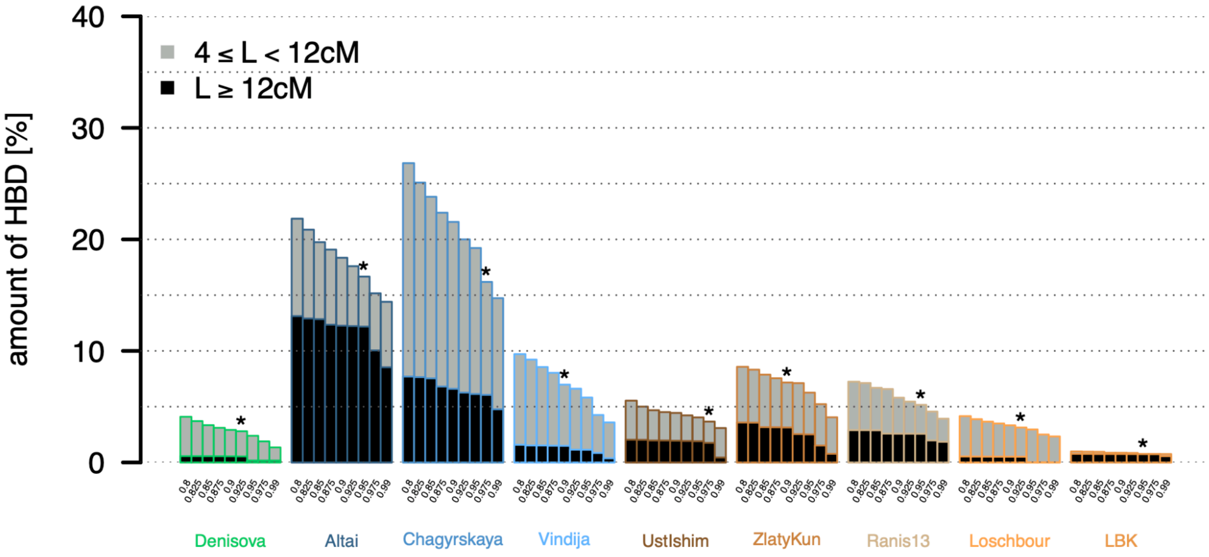

Supplementary Figure 7.3: Amount of consanguinity. The height (y-axis) of the bars is the percentage of HBD over the total and the different bars on the x-axis the different values of π for the high coverage ancient genomes. Filled black and gray bars correspond to the sum HBD segments with long and intermediate lengths (L), respectively, as shown in the legend. The asterisks on top of the bars represent the optimal values of π for each genome (see text).

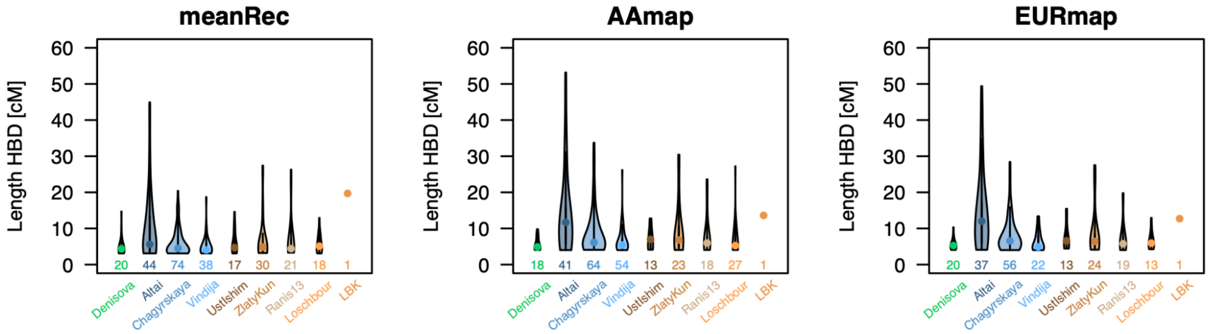

Supplementary Figure 7.4: Length distributions of HBD segments using different recombination rates. The width of the violins is proportional to the number of HBD segments (n) reported under the violins and the points represent the median of each distribution.

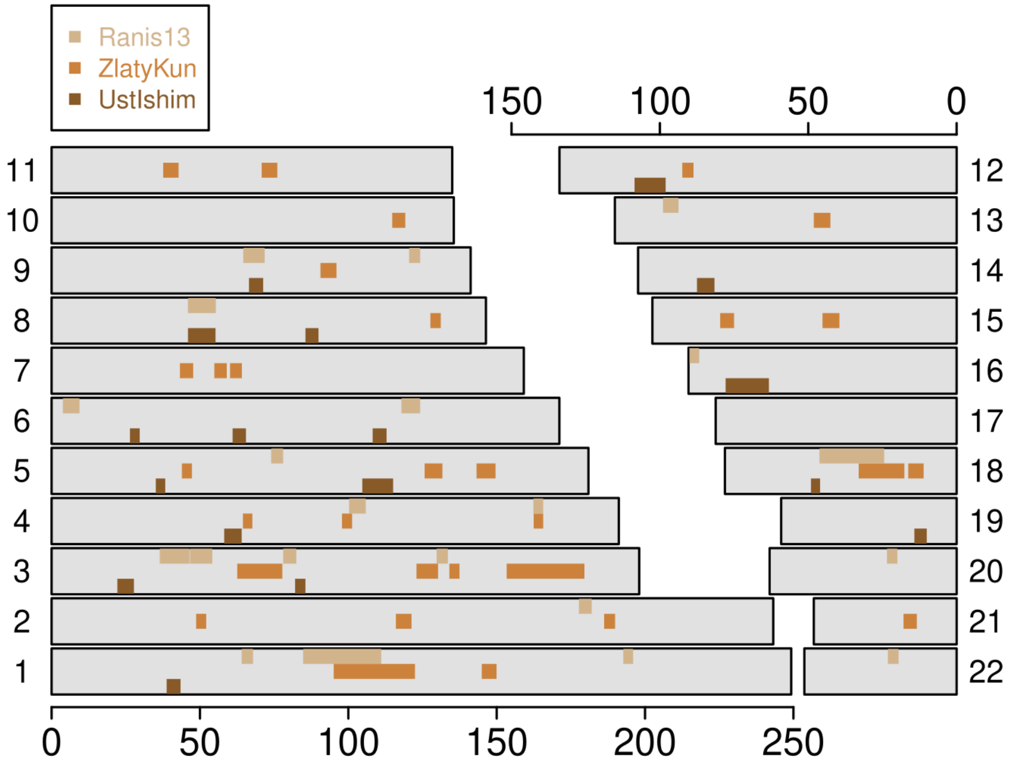

Supplementary Figure 7.5: Locations of the HBD segments in autosomes of the three high coverage Early Modern Human genomes. Each gray box indicates a chromosome with the physical position on the x-axis in Mb and the bars represent HBD regions in different genomes in colours as in Supplementary Figure 7.3.

ROH for low coverage capture data

We used hapROH[40] to detect and classify runs of homozygosity (ROH) (we use ROH and HBD interchangeably) in the genotyped data produced with pileupcaller (https://github.com/stschiff/sequenceTools) and 1240k bedfile of our high coverage and low coverage genomes, produce either via deep sequencing (high coverage data) or using 1240k capture (low coverage data). The 1240k capture data from four specimens (RNI012, RNI080, RNI081, and RNI085) were merged to form Ranis12, since these samples originate from the same individual, as described earlier (Supplementary Information 6). The coverage for Ranis6 and Ranis10 was insufficient for our analysis after capture (<300,000 sites covered) and these individuals were excluded from further analysis. The sites overlapping the 1240k positions were extracted from the bamfiles of the high-coverage shotgun data for Zlatý kůň and Ranis13. We also include the previously genotyped 1240k bam files published for Zlatý kůň[3] as an additional control and genotyped 1240k bam files from Bacho Kiro and Oase1 as published in Hajdinjak et

al., 2021[41]. We used the genotyped version of capture data from Tianyuan and Ust'-Ishim from the ADDR dataset as published in Posth et al., 2023[25]. Concretely for Ust'-Ishim, the snpAD genotyped version from Prüfer et al., 2017[31] available from ADDR was used.

The accumulation of short ROH (< 12 cM) in all individuals is indicative of background relatedness resulting from a small population size. Ranis13 exhibited the highest cumulative values amongst those compared, but we caution that the absence of short ROH (< 12 cM) in some individuals may be attributed to lower coverage. For long regions (> 12 cM), Ranis and Zlatý kůň individuals show the highest fractions of ROH in their genomes, followed by Bacho Kiro F6-620. We note that F6-620 is the highest quality sample among the published Bacho Kiro samples. Further high-quality samples will be needed to establish whether Bacho Kiro individuals show a consistent similarity in their fraction ROH to Ranis and Zlatý kůň individuals.

The detection of long ROHs are more dependent on data quality because longer fractions of the genome need to be covered without gaps. For this reason, we have plotted the location of ROH longer than 4 cM on the chromosomes to search for regions of the genome that could be cases of long ROH, but were erroneously split into several shorter ROH (Supplementary Figure 7.6). In Supplementary Figure 7.6, we observe adjacent ROH shorter than 4 cM that could potentially indicate the presence of longer ROH segments fragmented due to data quality issues (e.g. Chromosome 20 of Ranis87, Chromosome 22 in Tianyuan, Chromosome 15 in Bacho_Kiro_IUP_BB7_240deam, etc.). However, these potential long ROH cases do not change our overall results, that the genome under ROH in Ranis4, Zlatý kůň and Ranis13 is higher compared to the other individuals, except Bacho Kiro F6-620 which has comparable values.

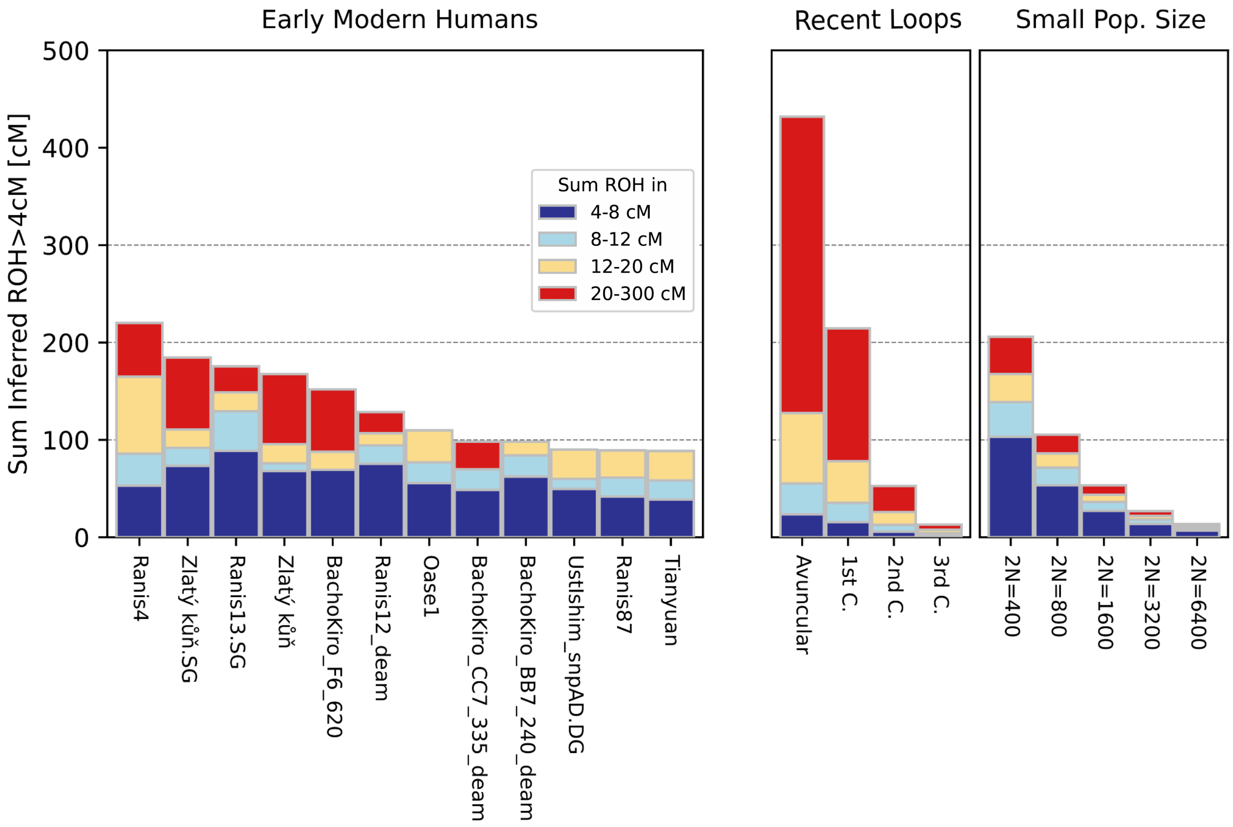

Supplementary Figure 7.6: Detected ROH for each individual, colour coded in four length classes. On the left, the estimates for Early Modern Human genomes are presented including those for Ranis and Zlatý kůň, and on the right are the expected amounts of ROH under certain simulated scenarios of close consanguinity and small population size[40]. SG and DG in the individual IDs indicate shotgun sequenced data, deam stands for limiting sequences to only those with deamination on the terminal three bases, and the absence of any indicates 1240k data is used.

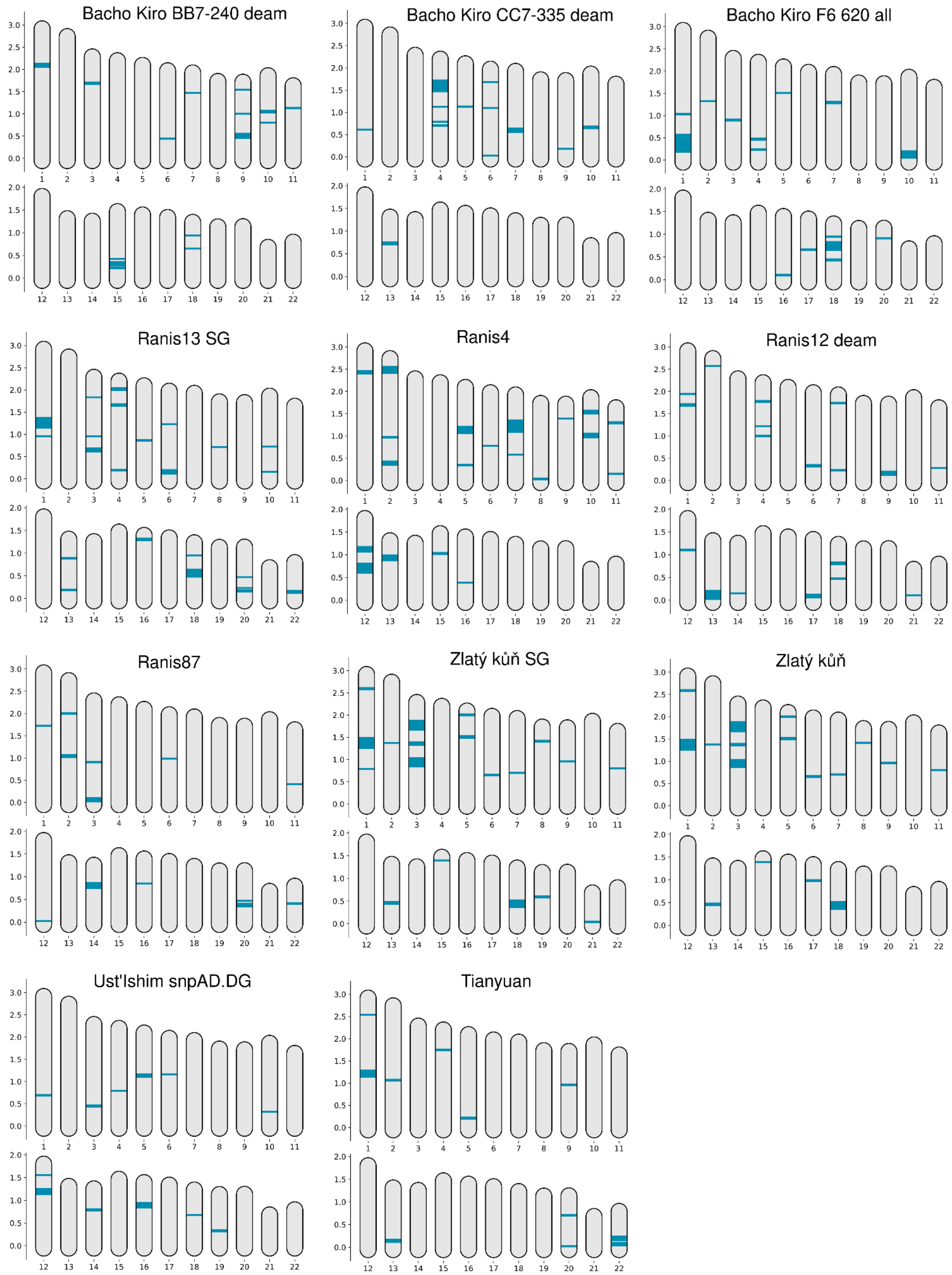

Supplementary Figure 7.7: Locations of the ROH > 4 cM for each Early Modern Human genome included in the hapROH[40] analysis. We included the high coverage Zlatý kůň and Ranis13 genomes subsetted to the 1240k capture array site (indicated as ZlatyKun.SG and RNI013.SG, respectively), along with the lower coverage 1240k capture data from these individuals.

Supplementary Information 8

IBD Analysis

Yieli Huang, Harald Ringbauer

Downsampling experiment on the two high-coverage genomes

To assess imputation performance on samples as old as ~45,000 years, and its implications on IBD calling with ancIBD[42], we conducted a series of downsampling experiments on the two high-coverage genomes of Ranis13 and Zlatý kůň. As previous studies have shown that 1240k data with 650k-700k SNPs covered at least once are comparable to the whole genome sequencing (WGS) data with 0.2x average sequencing depth[42], we downsampled WGS BAM files of Ranis13 and Zlatý kůň to 0.1x, 0.2x, 0.5x, 0.75x, with 25 independent replicates for each coverage and then performed imputation and IBD calling as described in Ringbauer et al., 2023[42].

To determine the "ground truth" IBD segments for this analysis, we used the snpAD[32] called diploid genotypes as described in Supplementary Information 5 and applied IBIS[43] to call IBD > 6 cM shared between Ranis13 and Zlatý kůň. IBIS uses the fact that since IBD state requires the two samples to share one identical haplotype, they cannot have different homozygous alleles ("opposing homozygote") at one site. Since this does not require phasing information, IBIS is particularly well-suited for high-coverage aDNA samples where diploid genotypes can be confidently determined but high-quality phasing is not attainable. As IBIS was originally designed for SNP-array data, it is sensitive to genotyping errors; therefore, we further filter snpAD output to transversion sites to eliminate the effects of aDNA damage. We then apply IBIS using its default settings on these filtered snpAD vcf files.

When comparing the ancIBD-called IBD segments in downsampled replicas to those inferred from the high-coverage data with IBIS, we found that, for the two ~45,000-year-old individuals, ancIBD[42] can reliably identify long IBD (> 12 cM) down to 0.1x WGS coverage (Supplementary Figure 8.1). Lengths greater than 12 cM are particularly informative for identifying relatives. In contrast, IBD segments in the length range 8-12 cM are informative for recent demography, and ancIBD's precision and recall start to decay at 0.2x but remain overall robust. We note that some

of the potential false positives in the 8-12 cM range are the result of a < 8 cM IBD segment in the "ground truth" set being inferred as > 8 cM.

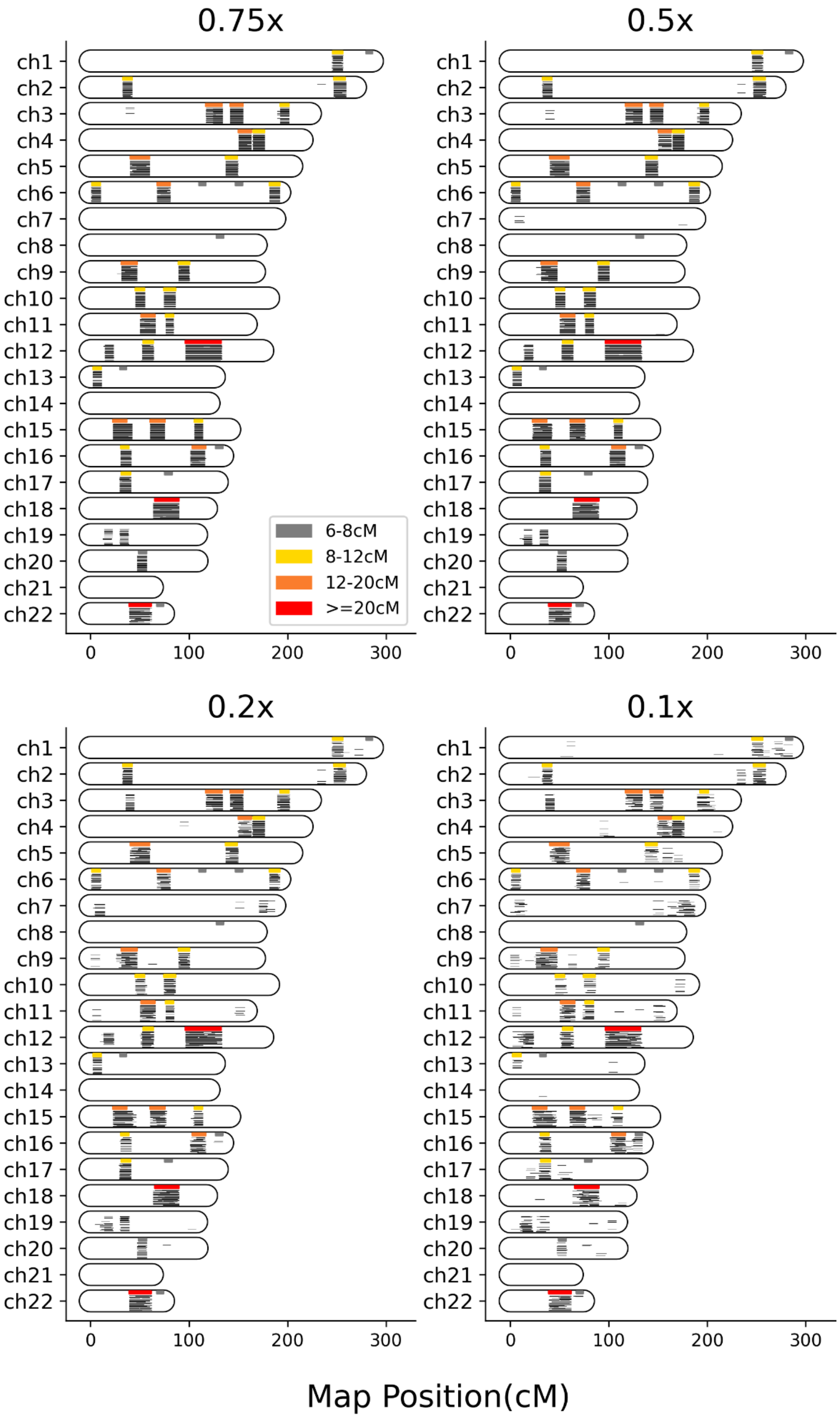

Supplementary Figure 8.1: Performance of ancIBD on downsampled WGS data of Ranis13 and Zlatý kůň. We visualize "ground truth" IBD (plotted at the top of each chromosome, coloured differently for four length bins) against inferred IBD (plotted as black bars, each replicate stacked vertically). For inferred IBD segments from downsampled data, only segments longer than 8 cM are shown.

Applying ancIBD to other samples

Having established that ancIBD works well for 0.2x WGS data, which is comparable to 1240k data with 650k-700k SNPs covered at least once, we applied ancIBD to the 1240k data of additional individuals, which includes three more Ranis individuals (Ranis12, Ranis4, Ranis87) and four individuals from Bacho Kiro Cave, Bulgaria[41] (BK1653, BK_BB7-240, BK_CC7-335, BK_F6-620). We imputed the high-coverage Ranis13 and Zlatý kůň data alongside the low-coverage data to avoid batch effects from imputation. We visualized IBD sharing among these samples in Supplementary Figure 8.2 (Supplementary Table 8.1).

First, we tried to determine the pedigree relationship between Ranis4 and Ranis12. Since the Ranis population has a relatively small effective population size (Ne) and therefore is expected to share substantial background IBD even in unrelated pairs, we first estimated Ne from ROH using all Ranis individuals with at least 300,000 of the 1240k SNPs covered (see Supplementary Information 7 for details). Assuming no consanguinity (but see Supplementary Table 8.2), we estimate Ne from 4-20 cM ROH for the Ranis population to be 299.7 (95% CI: 241.9-374.0), following the approach described in Fernandes et al., 2021[44]. With this Ne, the expected background IBD sharing is $\text{sumIBD}_{>12\text{cM}}$ 183.95 cM and $\text{nIBD}_{>12\text{cm}}$ 8.58. Subtracting this value from Ranis4's and Ranis12's raw IBD sharing ($\text{sumIBD}_{>12\text{cM}}$ = 758.6 cM, $\text{nIBD}_{>12\text{cm}}$ = 28), we found that Ranis4 and Ranis12 are consistent with being third or fourth-degree relatives[3]. In addition, all Ranis pairs share multiple long IBD segments, indicating an overall small effective population size.

We note that Ranis12 has a relatively high contamination rate (~10%); therefore, we also called IBD of Ranis12 with others using BAM files filtered to reads exhibiting aDNA-characteristic damage patterns. With this filtering, Ranis12 shares slightly less IBD with others (Supp Fig 8.3), but called IBD segments remain largely consistent and Ranis12 still shares the IBD characteristic of a third or fourth-degree relative with Ranis4.

We also compared IBD sharing between Ranis and Bacho Kiro (hereafter referred to as BK) individuals. We found that Ranis and BK share no IBD > 12 cM (Supplementary Figure 8.4), and each pair between Ranis and BK only shares a few IBD 8-12 cM with total length sum < 30 cM. As shown in Supplementary Figure 8.4, the Ranis and BK individuals form two distinct clusters on the IBD heatmap constructed from $\text{sumIBD}_{>12\text{cM}}$. We note that Zlatý kůň clusters tightly within the Ranis cluster and appears indistinguishable from the rest of the Ranis individuals. BK1653 shares no IBD with all others. This is expected as BK1653 was dated to ~8,200 years more recent than the other BK samples and was shown to be genetically more similar to later Upper Palaeolithic individuals from Europe than other BK samples, while the other BK samples are more similar to each other than to later populations.

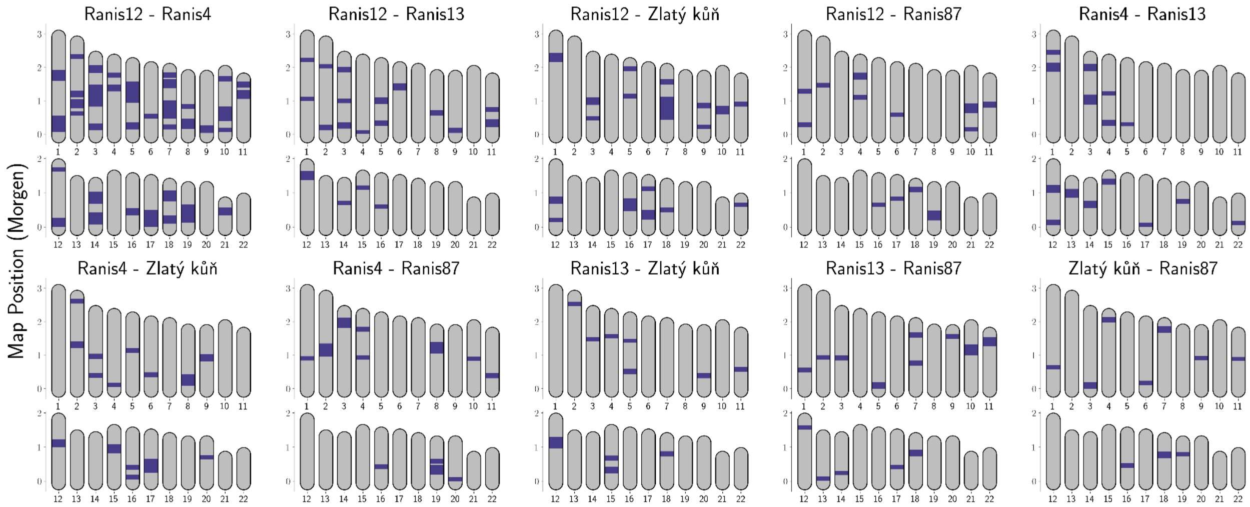

Supplementary Figure 8.2: IBD sharing patterns among Ranis, Zlatý kůň and Bacho Kiro individuals. Only IBD > 12 cM are shown.

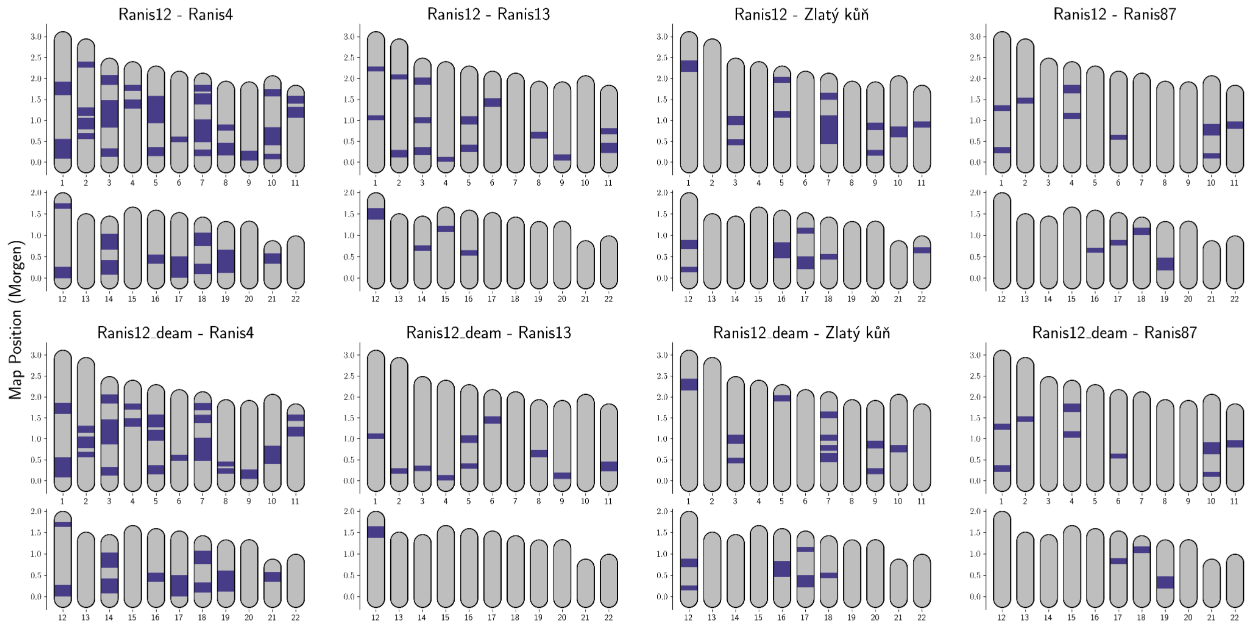

Supplementary Figure 8.3: Comparing IBD of Ranis12 filtered to reads with aDNA characteristic damage patterns to that of all reads. Each column is a pair involving Ranis12, and the first row shows IBD called with data imputed from Ranis12's original BAM file and the second row shows IBD called with data imputed from Ranis12's filtered BAM file. Only segments > 12 cM are shown.

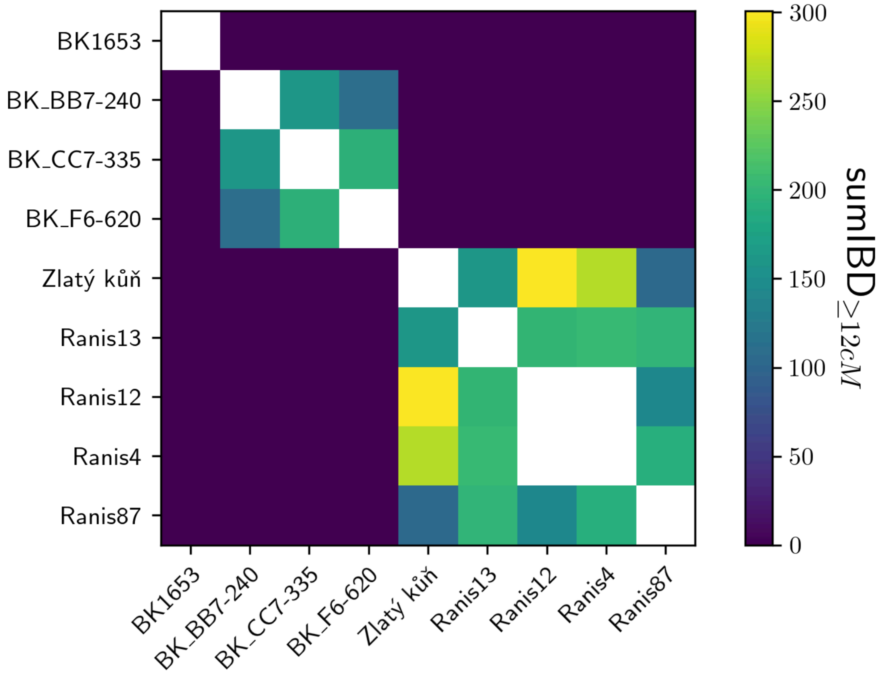

Supplementary Figure 8.4: Heatmap of IBD sharing among Zlatý kůň, Ranis and Bacho Kiro individuals. The similarity matrix is constructed by summing up the length of IBD segments longer than 12 cM. The IBD sharing between Ranis4 and Ranis12 is not shown because they are close relatives.

Supplementary Table 8.1: Number and total sum of the lengths of the shared IBD segments between pairs of individuals.

Pair		IBD > 12.0 cM		IBD > 16.0 cM		IBD > 20.0 cM	
**Individual 1**	**Individual 2**	**Sum (cM)**	**n**	**Sum (cM)**	**n**	**Sum (cM)**	**n**
Ranis4	Ranis12	758.6132914	28	664.2546035	21	607.8018941	18
Ranis4	Zlatý kůň	268.237124	13	167.2704175	6	167.2704175	6
Ranis12	Zlatý kůň	300.7843703	13	216.3156822	7	179.7551826	5
Ranis12	Ranis13	201.3394967	12	124.4645983	6	71.58399522	3
Ranis4	Ranis13	204.8702061	10	166.0786122	7	148.4541118	6
Ranis4	Ranis87	189.7304863	9	120.4339951	4	104.2358935	3
Ranis13	Ranis87	199.3063062	11	99.07030313	4	60.2823019	2
Ranis13	Zlatý kůň	162.6308918	10	67.06140041	3	34.08569694	1
Ranis12	Zlatý kůň	144.9987009	9	64.38469887	3	28.42069864	1
Ranis87	Zlatý kůň	105.2392133	7	40.04029786	2	20.55429816	1

Supplementary Table 8.2: Inferred Ne from ROH with different length ranges. It cannot be unambiguously determined whether consanguinity occurred among the Ranis individuals and to what extent. However, shorter ROH segments are generally less affected by consanguinity and mostly reflect background relatedness as a result of small effective population size. Therefore, we estimated Ne from ROH using progressively shorter segments. We found that although the inferred Ne increases with shorter ROH, there is no change in orders of magnitude, and different ROH length ranges all support a small Ne on the order of ~300.

ROH length range used	Inferred (diploid) Ne (95% CI)
4-20 cM	299.7 (241.9-374.0)
4-16 cM	310.3 (250.4 - 393.0)
4-12 cM	319.0 (253.9 - 406.3)
4-8 cM	319.0 (250.4 - 417.7)

IBD-based dating

Skeletal elements from the Zlatý kůň individual were not successfully radiocarbon dated previously, likely due to collagen contamination from animal glue[3]. Here we utilize the extensive IBD sharing between Zlatý kůň and other radiocarbon-dated Ranis samples to provide a bound for the age of Zlatý kůň. As Zlatý kůň and Ranis13 both have high-coverage WGS data, from

which even shorter IBD can be reliably detected, we use IBD shared between the two as the basis of the genetic dating.

We adapt the model described in Ringbauer, Coop and Barton, 2017[,7], to accommodate the fact that two haplotypes are not sampled at the same time, and we are interested instead in inferring the time difference between the two sampled haplotypes. We assume the simplest case of a panmictic population with constant size (Ne). When two haplotypes are separated by Δt generations, the branch length to the most recent common ancestor t generations ago becomes $2t - \Delta t$. Then Equation 4 in Ringbauer, Coop and Barton, 2017[3,45], becomes:

$$E[K_l^t] = 2(2t - \Delta t)e^{-(2t-\Delta t)l} + (L - l)(2t - \Delta t)^2 e^{-(2t-\Delta t)l}$$

where L denotes the chromosome length and l denotes the IBD segment length. Multiplying this with the standard coalescent rate function (an exponential distribution with mean $2Ne$) and then integrating over t, one obtains:

$$E[N_l] = \int_{\Delta t}^{\infty} E[K_l^t] \frac{1}{2N} e^{-\frac{t-\Delta t}{2N}} dt$$

$$= \frac{e^{-l\Delta t}}{(1 + 4Nl)^3} \left(8N(1 + 4NL) + 2\Delta t(1 + 4NL)(1 + 4Nl) - (L - l)(\Delta t + 4Nl\Delta t)^2\right)$$

This describes the rate of IBD segment of length l along the chromosome of length L for a pair of haplotypes. Summing this over 22 autosomes and multiplying by four (there are four pairs of haplotypes between two diploid individuals), one obtains the IBD sharing rate genome-wide for a pair of diploid individuals.

Following Ringbauer, Coop and Barton, 2017[45], we compute the log-likelihood by assuming a Poisson distribution for the number of IBD segments in a small length bin. We use bin width 0.25 cM for IBD between 6 cM to 30 cM. We evaluate the log-likelihood on a 2D grid of Ne and Δt. Specifically, we evaluate the likelihood for $Ne = np.arange(10, 1000, 10)$ and $\Delta t = np.arange(0, 100, 1)$. The maximum likelihood estimate of Ne and Δt is the combination of Ne and Δt (out of the 2D grid) that yields the highest likelihood. We determine the 95% confidence region using the 14.7% likelihood region approach. Specifically, the 95% confidence region is the set of $(Ne, \Delta t)$ tuples satisfying the following:

$$\{(Ne, \Delta t) | likelihood(Ne, \Delta t) >= \max_{(Ne, \Delta t) \in 2Dgrid} likelihood(Ne, \Delta t) - 1.92\}$$

We first evaluated this approach using simulations. We simulated ancestral recombination graphs (ARG) using msprime and then extracted IBD segments from ARGs using its own API (function ibd_segments from the tskit package). We simulated $Ne = 250$ and $\Delta t = 0, 15, 25$, each with 50 independent replicates. We chose $Ne = 250$ because it is representative of the effective population size of the Ranis population and we anticipate the accuracy of the method depends heavily on the Ne. With small Ne, there will be more IBD segments, so meaningful inference is possible even with a single pair of diploid samples. We found that there is large variability in the estimated Ne and Δt, but they remain unbiased except for the case $\Delta t = 0$. This might be caused by the fact that the model is undefined for $\Delta t < 0$ and this asymmetry causes issues with optimization and calculations of confidence regions. Despite large variances of the estimates, we still find this model useful as the confidence region covers the true value most of the time.

We applied this model to IBD segments >= 6 cM shared between Ranis13 and Zlatý kůň. We used the segments detected by IBIS from snpAD-called transversion sites as IBIS has been benchmarked with shorter segments than ancIBD and we found using shorter segments helps with accuracy in our simulations. The maximum likelihood estimate is $Ne = 160, \Delta t = 3$. The 95% confidence region (100-240) is shown by the dashed black circle in Supplementary Figure 8.5D. Our modeling indicates that the age difference between Ranis13 and Zlatý kůň is at most 15 generations, or 435 years assuming a generation time of 29 years. We note that our model assumes two samples are drawn randomly from the population. If Ranis13 and Zlatý kůň are relatives, then our model would underestimate Ne and Δt. That said, Ranis13 and Zlatý kůň are contemporaneous in both cases. Therefore, our analysis supports the previous dating (~45,000 years BP[3]) by Neandertal introgression segment lengths and suggests that Zlatý kůň is contemporaneous with Ranis13.

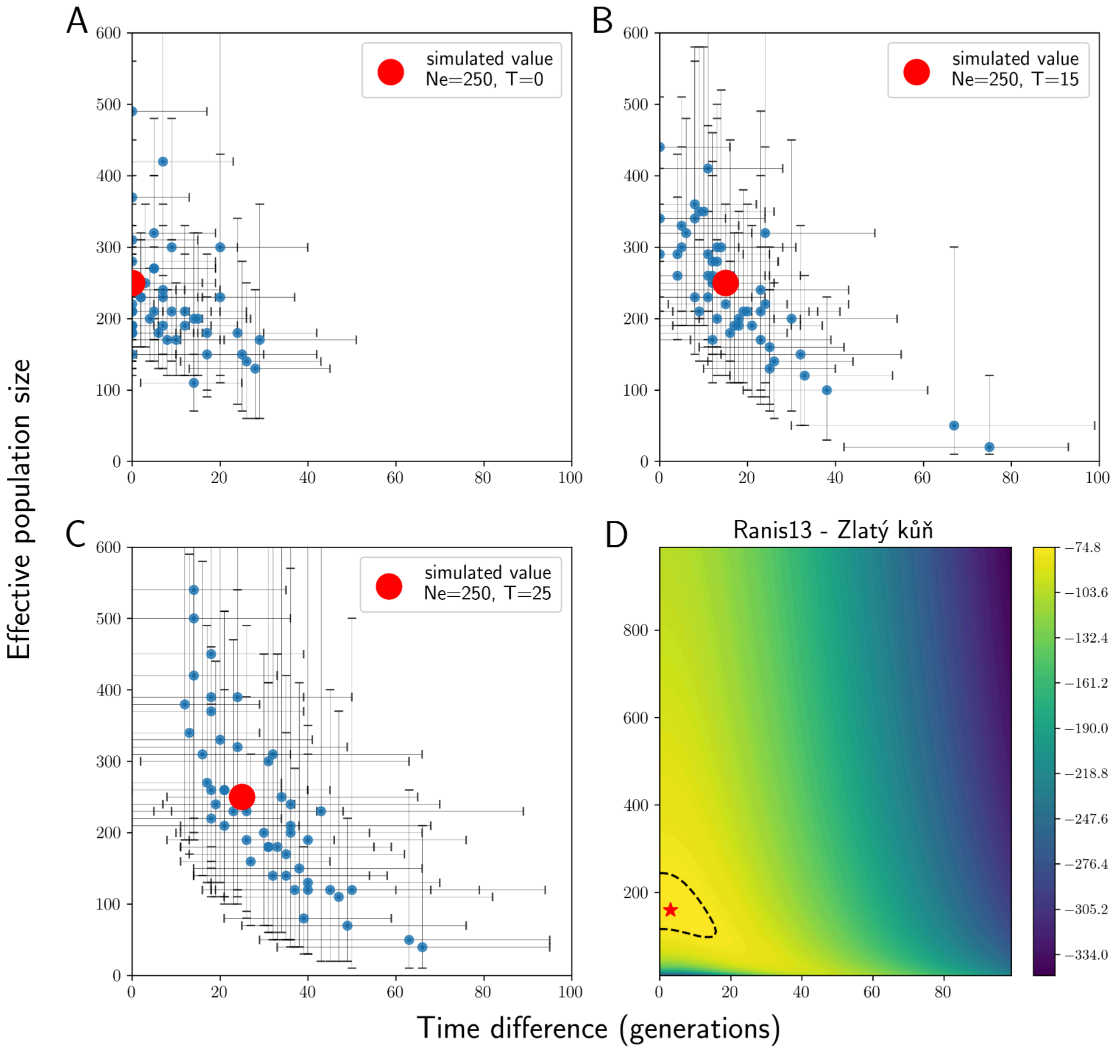

Supplementary Figure 8.5: IBD-based dating. **A.** Inferred effective population size (Ne) and time difference Δt for simulated $Ne = 250, \Delta t = 0$. Each blue dot represents one simulated replicate. Error bars represent 95% confidence intervals. **B.** Same as panel A but with $\Delta t = 15$. **C.** Same as panel A but with $\Delta t = 25$. **D.** Likelihood over the 2D grid for IBD shared between Ranis13 and Zlatý kůň. The red star indicates the maximum likelihood estimate and the black dashed circle indicates the 95% confidence region.

Supplementary Information 9

Population Genomic Analysis

Vanessa Villalba-Mouco*, Arev Pelin Sümer, Mateja Hajdinjak, Kay Prüfer

Analysis using the 1240k capture sites

Since a large number of hunter-gatherer (HG) individuals have been sequenced after enriching for ~1.2 million positions of known human diversity (1240k), we first analyzed Ranis and Zlatý kůň data using these sites. Unless otherwise stated, we use two different sets of individuals for downstream analysis:

1. RNI: a set of individuals with low contamination that is suitable for analysis without further filtering and includes Ranis4, Ranis13 and Ranis87.
2. RNI_deam: includes all the above and, in addition, individuals Ranis10 and Ranis12. Since we estimated modern human contamination over 5% for these individuals, the data from all individuals were filtered for sequences that show C-to-T substitutions at terminal positions to the human reference in alignment. Ranis6 was omitted from all analyses when Ranis is considered as a group, due to the first degree relatedness to the Ranis4 individual (Supplementary Information 6).

Generally, 1240k capture data were used for all individuals. However, for some analyses, shotgun data were analyzed by subsetted the reads to those overlapping 1240k sites (denoted by the suffix ".SG" in the sample name).

For comparative analyses, we incorporated the recently published dataset by Posth et al., 2023[25] and Villalba-Mouco et al., 2023[46], as well as the genomes of Bacho Kiro, Oase[41], and Buran Kaya[47] individuals.

Genetic clustering analysis of the Ranis individuals

To compare Ranis individuals with previously published hunter-gatherer (HG) individuals that date to before 11kya, we ran *f3(HG, HG; Mbuti)* among all possible pairs of individuals/groups with more than ~30,000 SNPs covered in the 1240k panel. We also included the new ~36,000 and ~37,000-year-old Buran-Kaya III individuals from Crimea published by Bennett et al.,

2023[47], despite the low number of covered 1240k sites (17,830 SNPs for BuKa3A and 26,249 for BuKa3C, respectively). The resulting *f3*-matrix (Extended Figure 3) shows that Ranis, Zlatý kůň, Bacho Kiro_IUP individuals, Oase 1, Ust'-Ishim, and Tianyuan form a group. Within this group, the individuals from Ranis and Zlatý kůň share more drift with one another than with any other HG individual. Outside the group of the oldest HGs, we observed the grouping of the remaining HG individuals following previously described genetic clusters, such as Fournol, Věstonice, GoyetQ2, and Villabruna+Oberkassel individuals[25,48,49]. An alternative visualization, the result of a Multi-Dimensional Scale analysis (MDS) on the distance matrix calculated as 1 − *f3(HG, HG; Mbuti)*, is shown in Supplementary Figure 9.1.

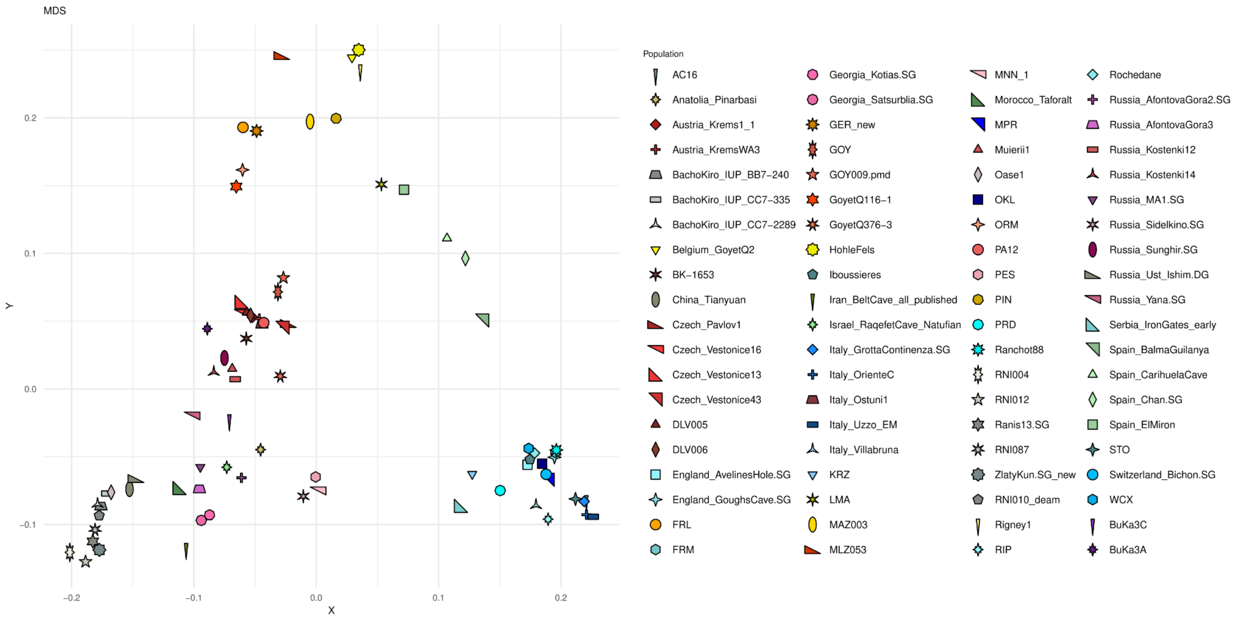

Supplementary Figure 9.1: MDS plot using the pairwise genetic distances calculated via 1 − *f3(HG, HG; Mbuti)* showing the main genetic clusters defined previously[25,48,49] and the new Ranis and Zlatý kůň individuals.

Testing the genetic relationship of Ranis to other hunter-gatherer (HG) individuals

The *f3*-outgroup analysis suggested a close relationship between Ranis and Zlatý kůň individuals. To formally test this relationship, we performed all *f4*-outgroup tests at an individual level using *Ranis13.SG*, *Ranis4*, *Ranis6_deam*, *Ranis10_deam*, *Ranis12_deam*, *Ranis13*, *Ranis87*, the capture version of Zlatý kůň[3] and the new high coverage shotgun genome (SG) of Zlatý kůň generated in this study (*Zlatykun.SG*). In an attempt to gain further power, we also ran

the analysis with Ranis as a group, using either individuals for which all sequences can be used (*RNI = Ranis4 + Ranis13 + Ranis87*), or a version where further individuals are included and filtering for C-to-T at sequence ends are applied (*RNI_deam = Ranis4_deam + Ranis10_deam + Ranis12_deam + Ranis3_deam + Ranis87_deam*).

We first calculated *f4*-outgroup statistics of the form *f4(RNI, test; Zlatý kůň, Mbuti)* and found significantly positive values for most of the tested populations (Z-score > 3). From the remaining comparisons that show positive values but do not reach our significance threshold of Z-score > 3, they involve individuals with low coverage (Table S4.1), Supplementary Figure 9.2A, B and C). These results indicate that Zlatý kůň is more closely related to Ranis individuals than any other HG individual in our comparison.

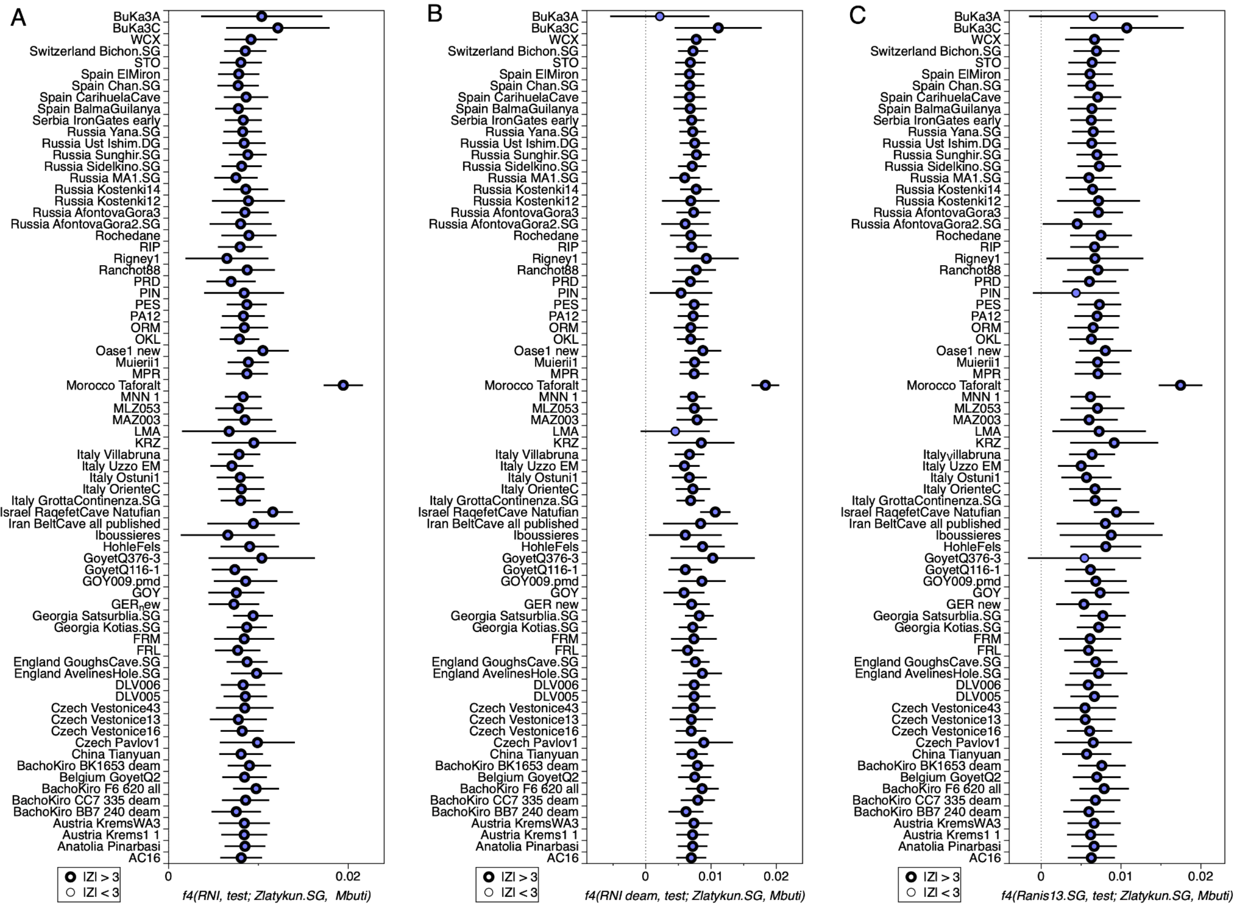

Supplementary Figure 9.2: A. The *f4*-outgroup statistics of the form *f4*(*RNI*, *test*; *Zlatykun.SG*, *Mbuti*) show a significant affinity between *RNI* and Zlatý kůň when comparing any test population (Table S4.1). **B.** Same *f4*-test as in A but using the deaminated version of *RNI* and reporting similar results (Table S4.1).

C. Same f4-test as in A and B but using Ranis13.SG (i.e. shotgun data, 1240k SNP subsetted) and reporting similar results (Table S4.1). Error bars correspond to three standard errors.

To test if *RNI* and Zlatý kůň were part of the same genetic clade, we calculated a *f4*-outgroup test of the form *f4(RNI, Zlatý kůň; test, Mbuti).* The test will result in significant Z-scores if either *RNI* or Zlatý kůň share more ancestry with the test individual. The results were consistent with f4 = 0 (Z-scores < |3|), providing no evidence against a model of a coherent population consisting of *RNI* and Zlatý kůň (Tables S4.1-S4.2, Supplementary Figure 9.3A, B and C).

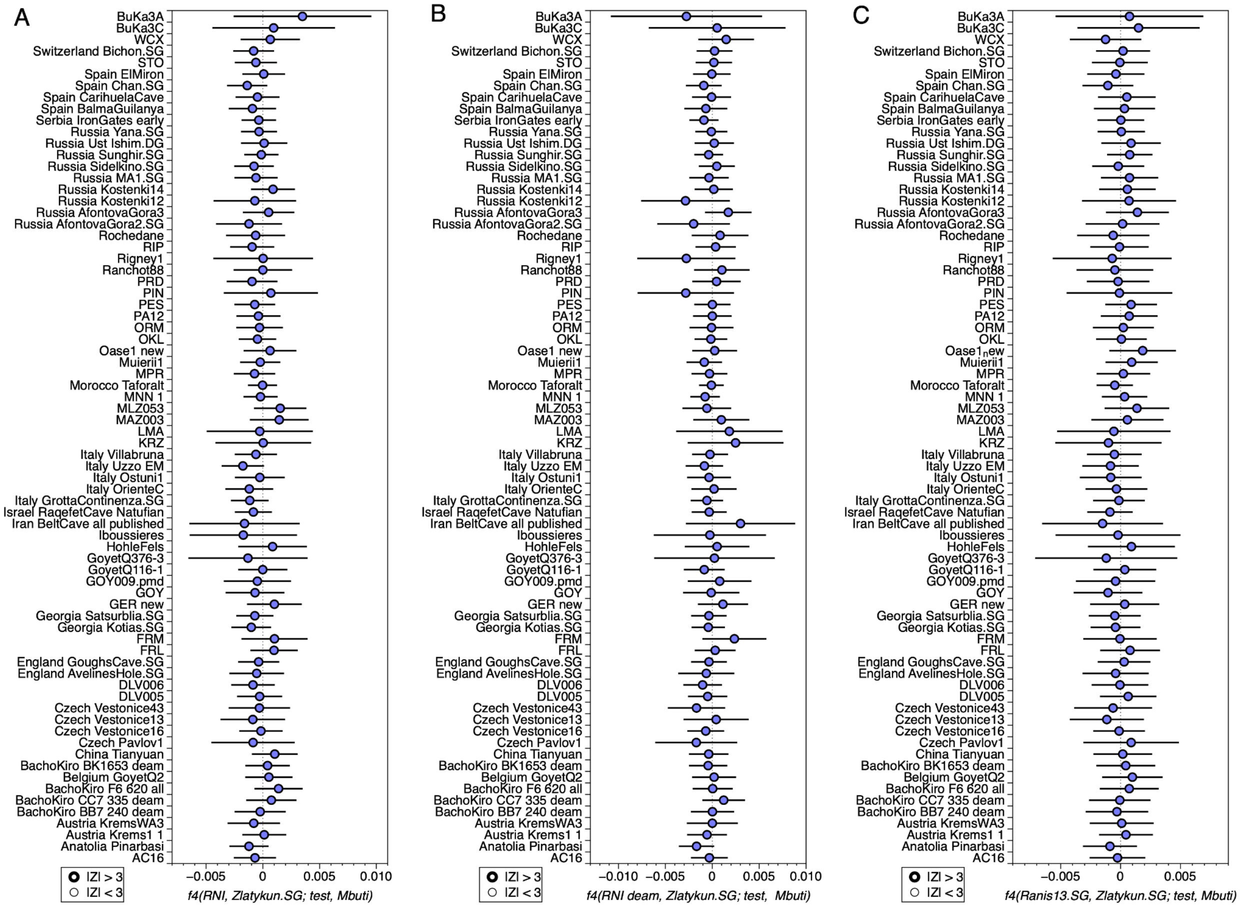

Supplementary Figure 9.3: **A.** The *f4*-outgroup statistics of the form *f4(RNI, Zlatykun.SG; test, Mbuti)* show that *Ranis* and Zlatý kůň are symmetrically related to any test population (Table S4.2). **B.** Same *f4*-outgroup test as in A but using the deaminated version of *Ranis* and reporting similar results. **C.** Same *f4*-outgroup test as in A and B but using *Ranis13.SG* (shotgun data, 1240k SNP subsetted) and reporting similar results. Error bars correspond to three standard errors.

Exploration of the genetic contribution of Ranis/Zlatý kůň to later populations

Previously reported genomic data from Zlatý kůň showed that this individual represented a population that split earlier than Asians and Europeans split from each other, and that the population Zlatý kůň belonged to did not contribute substantially to specific subsequent out-of-Africa populations[3]. However, a recent study from Bennett et al., 2023[47] suggests that Zlatý kůň-like ancestry is found in the two Buran-Kaya III individuals from the Crimea peninsula which were dated to ~37,000 and ~36,000 years BP.

To investigate the potential contribution of Zlatý kůň and Ranis-like ancestry to these and other HG populations, we calculated *f4(HGi, HGj; Ranis13.SG, Mbuti)* where *HGi* and *HGj* represent all possible combinations of HGs older than ~11,000 years BP. Using the highest coverage individual, Ranis13.SG, as the representative of Ranis and Zlatý kůň-like ancestry, we performed a total of 3,003 tests.

We observed that the majority of the results were consistent with zero, i.e. consistent with no difference in contribution between the HG individuals compared. Disregarding the previously demonstrated close relationship amongst Zlatý kůň and Ranis individuals, we only find significant deviations from zero (Z-score > |3|) for comparisons that typically involve one HG population with high levels of African (Taforalt) or Basal Eurasian ancestry (e.g. Kotias, Satsurblia, Natufian, Anatolia_Pinarbasi or Iran Belt Cave) (Table S4.3, Supplementary Figure 9.4A and B). African and Basal Eurasian ancestry are more closely related to the African outgroup than out-of-Africa ancestry is, and can thus lead to significant deviations from zero in this particular test. Eight of the remaining nine comparisons included an individual from the Villabruna/Oberkassel cluster (England_AvelinesHole.SG, STO, Serbia_IronGates_early, WCX), which were all previously shown to have Near Eastern-like ancestry that also contains some level of Basal Eurasian ancestry. The remaining comparison which deviates from zero is with *Sidelkino.SG*, a group that shows increased sharing of alleles with Caucasus HGs who also carry Basal Eurasian ancestry.

BuKa3A and BuKa3C, the two individuals from Buran Kaya III, showed no significant deviations in the direction of increased allele sharing with Ranis. Three *f4*-outgroup test combinations that included BuKa3C showed a Z-score > 2.5: *f4(Czech_Vestonice43, BuKa3C;*

Ranis.13SG, Mbuti), *f4(Italy_OrienteC, BuKa3C; Ranis.13SG, Mbuti)* and *f4(Austria_Krems1_1, BuKa3C; Ranis.13SG, Mbuti)* with a minimum number of SNPs overlapping (2,900, 6,649 and 16,251 SNPs, respectively). This indicated less sharing between BuKa3C and Ranis13, which could be explained by subtle levels of Basal Eurasian ancestry in Buka3C (Table S4.3, Supplementary Figure 9.4A and B).

We investigate the Basal Eurasian ancestry further using the *f4*-outgroup statistics of the form *f4(test, Ust'-Ishim; Zlatý kůň, Mbuti)*. Previous tests (Supplementary Figure 9.4A and B, Table S4.3) have found no evidence for an increased sharing of alleles between Zlatý kůň and Ust'-Ishim, and detectable contribution from a Basal Eurasian lineage has never been suggested for any of the two genomes. This means that negative values in this *f4*-outgroup test are only expected if the test population carries an African-related (e.g. Basal Eurasian) contribution. Increased sharing with Ranis13, on the other hand, would result in positive values. The results of running this *f4*-outgroup test on all the HG individuals/groups older than 11,000 years BP are reported in Table S4.4 and Supplementary Figure 9.4C. Significant negative values (Z-score < 3) are reported for Taforalt, Natufian or Satsurblia individuals. Other populations that showed significant negative values, for the previous *f4(HGi, HGj; Ranis13.SG, Mbuti)* test consistently show negative values although this difference is not significant.

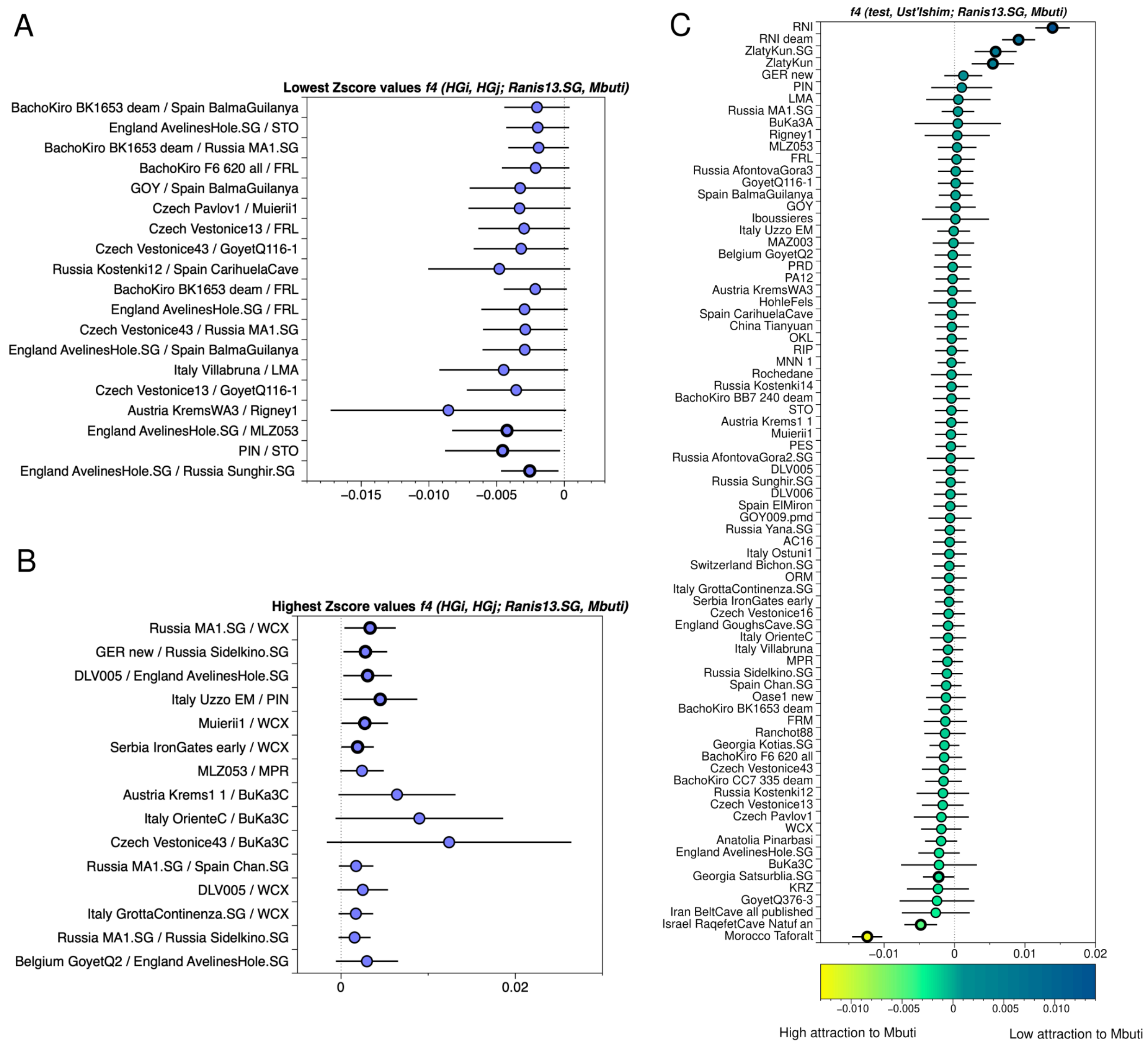

Supplementary Figure 9.4: A. Top-high Z-scores obtained from *f4(HGi, HGj; Ranis13.SG, Mbuti)* (Table S4.3). **B.** Top-low Z-scores obtained from *f4(HGi, HGj; Ranis13.SG, Mbuti)* (Table S4.3). **C.** *f4*-outgroup statistics of the form *f4(test, Ust'-Ishim; Ranis13.SG, Mbuti)* to measure the attraction to the outgroup (Mbuti) (Table S4.4). Error bars correspond to three standard errors.

Ranis, African populations and Basal Eurasian ancestry

Previous studies[3,25] have shown that the Zlatý kůň individual represents the most basal branch of Early Modern Humans (EMH) outside of Africa from whom we currently have genetic data available. However, previous results also indicate that the Basal Eurasian ancestry predates the split of Ranis and Zlatý kůň from a lineage leading to other out-of-Africans. As expected, when testing *f4(RNI/Zlatýkůň, HG; African, Chimpanzee)* with all HG older than 11,000 years without

Basal Eurasian ancestry, we find no significant differences in most cases (Supplementary Figure 9.5, Table S4.5). The only exceptions are Oase 1 and the Bacho Kiro Cave individuals, who showed positive values (some of them with a Z-score > |3|) as a result of an additional Neandertal ancestry[8,41]. Note that we have chosen populations from East (Ethiopia_4500BP)[50], West (Cameroon SMA)[51] and South Africa (SouthAfrica 2000_BP)[52] for Africans as these likely do not harbor European or Middle Eastern ancestry, which has been introduced later.

In contrast, when testing HG with Basal Eurasian ancestry (e.g. CHGs, Natufians, Anatolian HG and Morocco Iberomaurusian[53,54,38,55,56], test: *f4(Ranis/Zlatýkůň, HG with Basal Eurasian ancestry; Africans, Chimpanzee)*), we observed overall positive values (some of them with a Z-score > |3|) indicating that Basal Eurasian ancestry represents an earlier lineage than the one represented by Zlatý kůň/Ranis (Supplementary Figure 9.6).

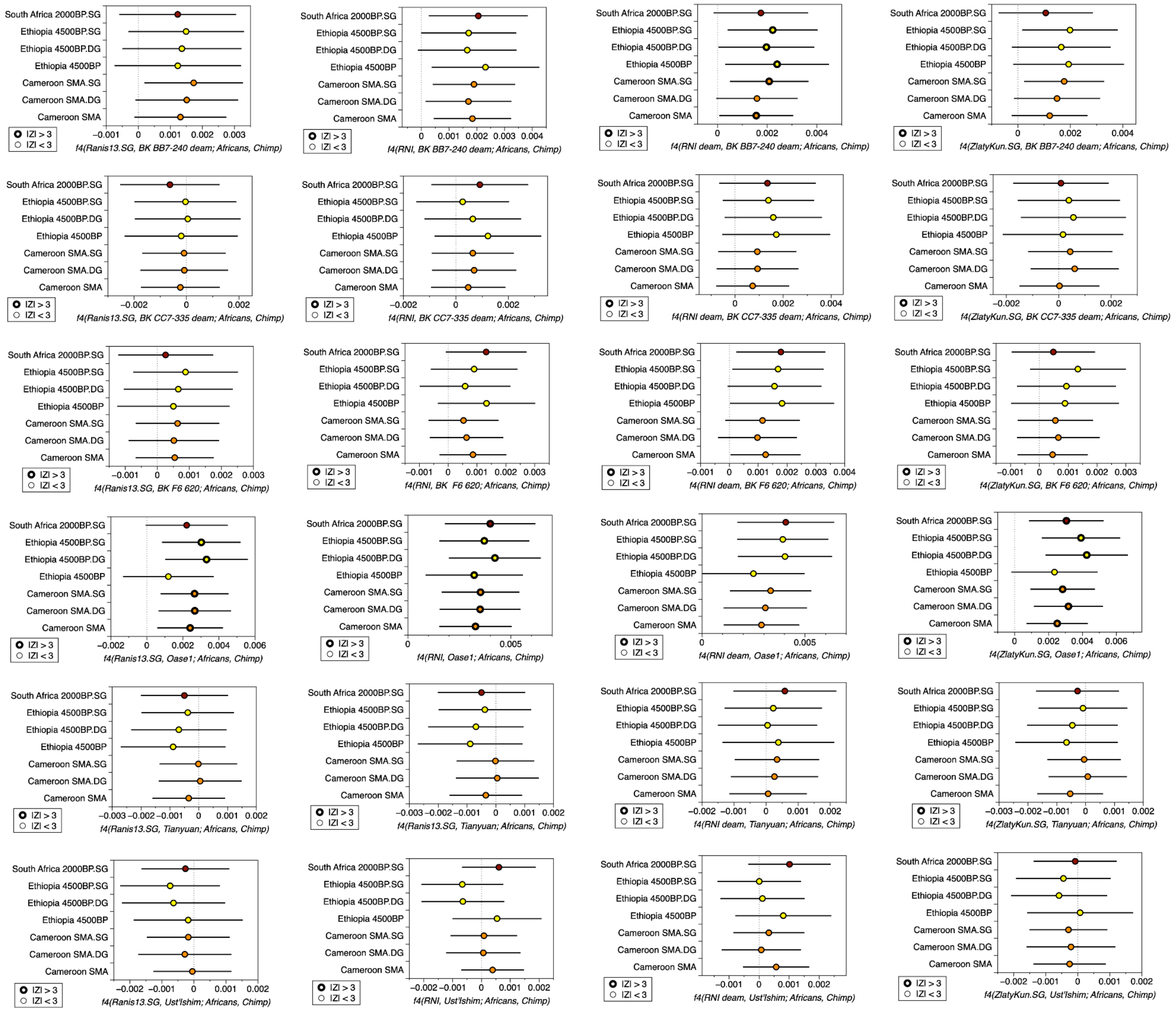

Supplementary Figure 9.5: The *f4*-outgroup statistics of the form *f4(Ranis/Zlatýkůň, test; African, Chimpanzee)* showing different genetic affinity to distant African populations. Each row represents a different EMH (Bacho Kiro IUP individuals, Ust'-Ishim, Tianyuan, and Oase 1) in the second position, and each column compares the Ranis and Zlatý kůň individuals with various data treatments (SG, capture and deaminated capture) (Table S4.5). Error bars correspond to three standard errors.

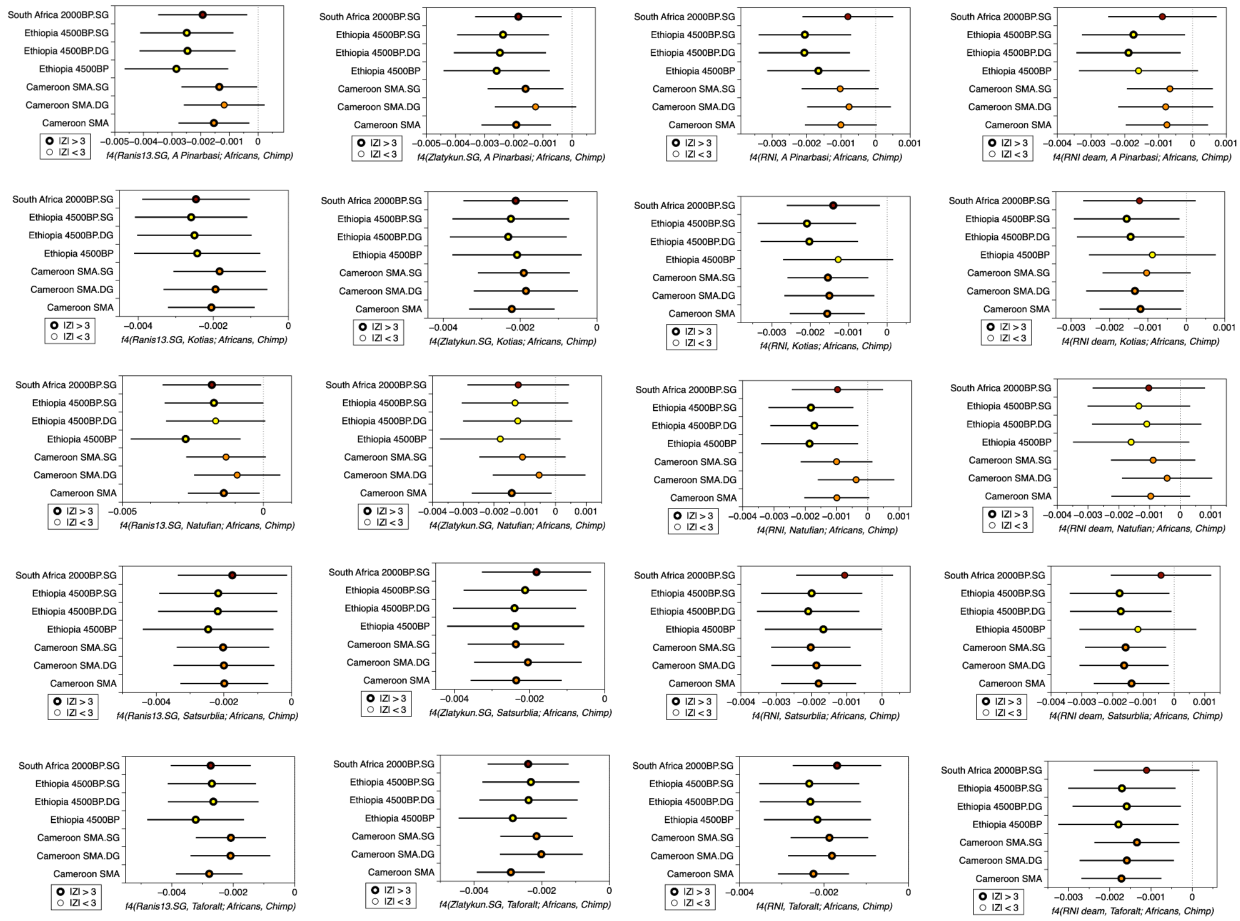

Supplementary Figure 9.6: The *f4*-outgroup statistics of the form *f4(Ranis/Zlatýkůň, Basal Eurasian ancestry; African, Chimp)* showing different genetic affinity to distant African populations. Each row represents a different HG with high levels of Basal Eurasian ancestry (Anatolia Pinarbasi, Kotias, Natufian, Satsurblia, and Taforalt) and each column compares the Ranis and Zlatý kůň individuals with various data treatments (SG, capture and deaminated capture) (Table S4.6). Error bars correspond to three standard errors.

Testing the genetic relationship of Ranis with Late Neandertals and Denisovans

We performed the *f3*-outgroup test of the form *f3(Ranis/Zlatýkůň, Neandertal; Chimpanzee)* to detect the Neandertal individuals with whom Ranis and Zlatý kůň share the most genetic drift. The results reported *Mezmaiskaya2_deam* as the top f3-value regardless of the *Ranis* version

tested, indicating a possible higher degree of shared genetic drift, although this difference is not significantly higher than that of the other Late Neandertals (Supplementary Figure 9.7, Table S4.7). We detected similar results (*Mezmaiskaya2_deam* showing the highest f3-values) when other EMHs were tested (e.g. Tianyuan, Bacho Kiro IUP individuals, Oase 1, and Ust'-Ishim). This consistent result could be indicative of *Mezmaiskaya2_deam* carrying residual levels of human contamination rather than reflecting population structure of Late Neandertals within the admixed Neandertal ancestry found in EMH[57].

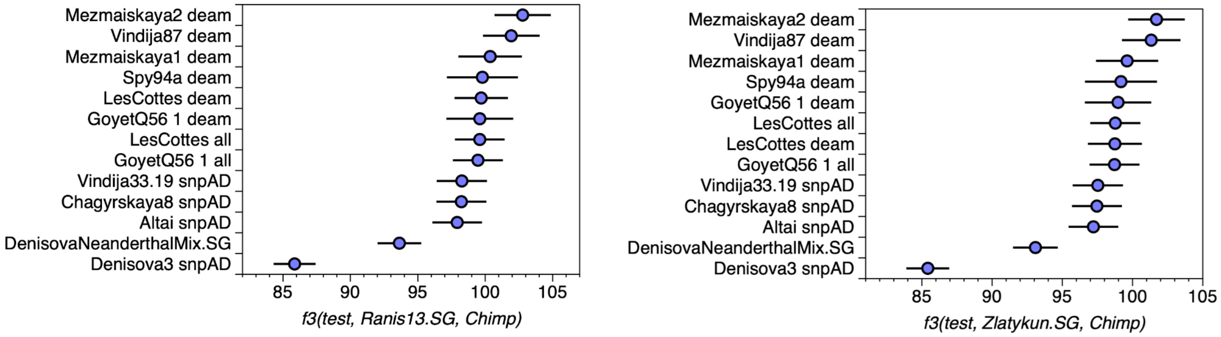

Supplementary Figure 9.7: The *f3*-outgroup statistics of the form *f3(test, Ranis13.SG/Zlatý kůň; Chimp)* showing the shared genetic drift between Neandertals and EMH. The *f3*-outgroup statistics with the rest of EMH can be found in Supplementary Table 9.7. Error bars correspond to three standard errors.

We also addressed the same question by using *f4*-outgroup statistics. In these tests we used the genome of the Altai Neandertal (an early, ~120,000-year-old, Neandertal from the Denisova Cave)[30] as baseline for Neandertal ancestry and measured the additional shared genetic drift with other Late Neandertals using an *f4*-outgroup test of the form *f4(Late Neandertals, Altai Neandertal; EMH, Chimp)*. The results obtained showed a consistent pattern of higher attraction of any EMH population to low coverage Late Neandertals with *Mezmaiskaya2_deam* and *Vindija87_deam* exhibiting the highest f4-values. This observation suggests that these higher values reflect residual levels of contamination in Late Neandertals rather than indicating a specific structure of the Neandertal ancestry in EMHs (Supplementary Figure 9.8, Table S4.8). This observation would be supported by the fact that Vindija87_deam and Vindija33.19 are the same individual, and only in the low-coverage version we see this increased affinity.

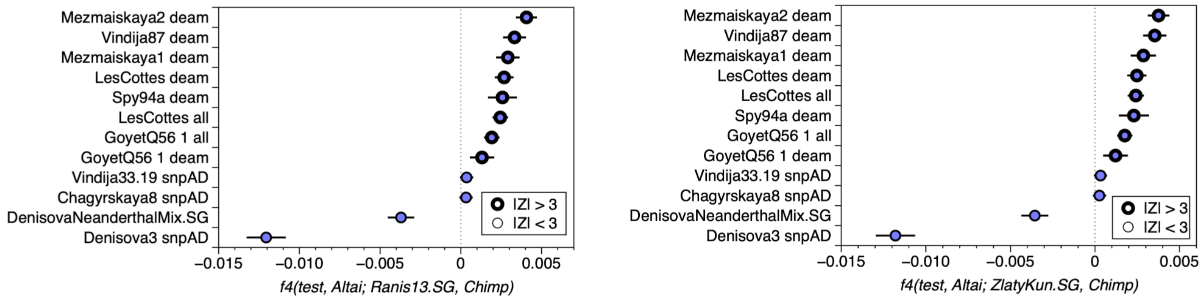

Supplementary Figure 9.8: The *f4*-outgroup statistics of the form *f4(test, Altai Neandertal; Ranis13.SG/Zlatýkůň, Chimp)* showing extra shared drift between Late Neandertals and *Ranis13.SG* and *Zlatykun.SG* when the Altai Neandertal is used as the baseline for Early Neandertal ancestry. The *f4*-outgroup statistics with the rest of EMH can be found in Table S4.8. Error bars correspond to three standard errors.

To test for differences between Ranis and Zlatý kůň in terms of Neandertal and Denisovan ancestry, we calculated *f4(Ranis, Zlatýkůň; Neandertal, Chimpanzee)* for both non-deaminated SG and deaminated SG versions of *RNI*, Ranis13 and Zlatý kůň (Supplementary Figure 9.9, Table S4.9). We observed no significant differences, with all the *f4*-values close to zero, giving no indication for a difference in Neandertal ancestry in Ranis13 and Zlatý kůň. Similarly, no significant differences are observed in comparisons against Denisova unless datasets with different filterings are mixed. In particular, we observe borderline significant tests when comparing the combined Ranis deaminated data (*RNI_deam*) against unfiltered Zlatý kůň shotgun data and *Ranis13_deam* against all data from Zlatý kůň capture. This latter comparison also results in |Z| > 3 when the Denisovan is substituted by Denisova11, the offspring of a Neandertal mother and a Denisovan father (Table S4.9).

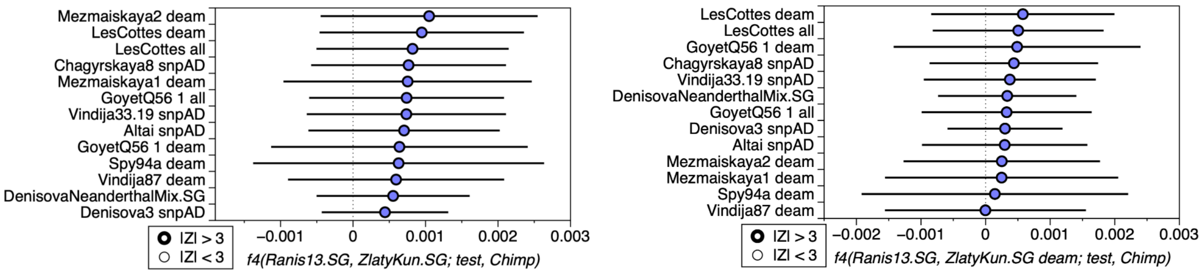

Supplementary Figure 9.9: The *f4*-outgroup statistics of the form *f4(Ranis13.SG, Zlatýkůň; Neandertal/Denisovan, Chimpanzee)* showing no differences between *Ranis13.SG* and *Zlatýkůň* regarding Neandertal and Denisovan ancestries (Table S4.9). Error bars correspond to three standard errors.

Phylogenetic position of Ranis (qpGraph modeling)

We use qpGraph from ADMIXTOOLS (https://github.com/DReichLab) to infer the population relationship of Zlatý kůň and Ranis to other EMH such as Bacho Kiro IUP, Ust'-Ishim, and Tianyuan. We built a scaffold tree with seven populations: Chimpanzee, Vindija Neandertal, Mbuti, Zlatý kůň, Ranis13, Bacho Kiro F6 620, and Sunghir, following the proposed structure by Posth et al., 2023[25]. When modelling Ranis13 and Zlatý kůň as sister branches from the same basal clade the worst Z-score was -1.874 (Supplementary Figure 9.10A). However, if we model Zlatý kůň basal to Ranis13 (Supplementary Figure 9.10B) or Ranis13 basal to Zlatý kůň (Supplementary Figure 9.10C), we obtain a worst Z-score of 8.882 in both cases.

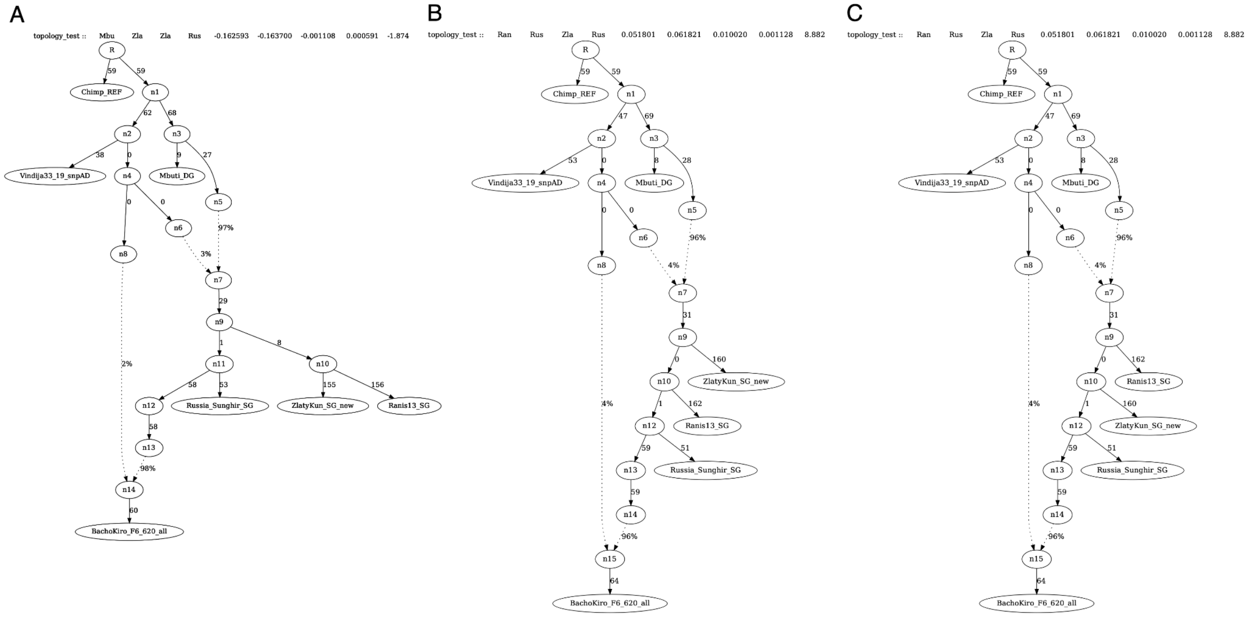

Supplementary Figure 9.10: qpGraph reconstructions including Ranis13 and Zlatý kůň individuals, together with Chimpanzee, Mbuti, Vindija33_19, Sunghir and Bacho Kiro F6 620. **A** qpGraph tree fitting Ranis13 and Zlatý kůň as sister branches from the same early split clade showing the worst Z-score of -1.874. **B** qpGraph tree fitting Zlatý kůň basal to Ranis13 and showing the worst Z-score of 8.882. **C** qpGraph tree fitting Ranis13 basal to Zlatý kůň and showing the worst Z-score of 8.882.

As in Hajdinjak et al., 2021[41], and Posth et al., 2023[25], we added Tianyuan and Ust'-Ishim as later splits. The worst Z-score reported for this tree was -2.470 (Supplementary Figure 9.11).

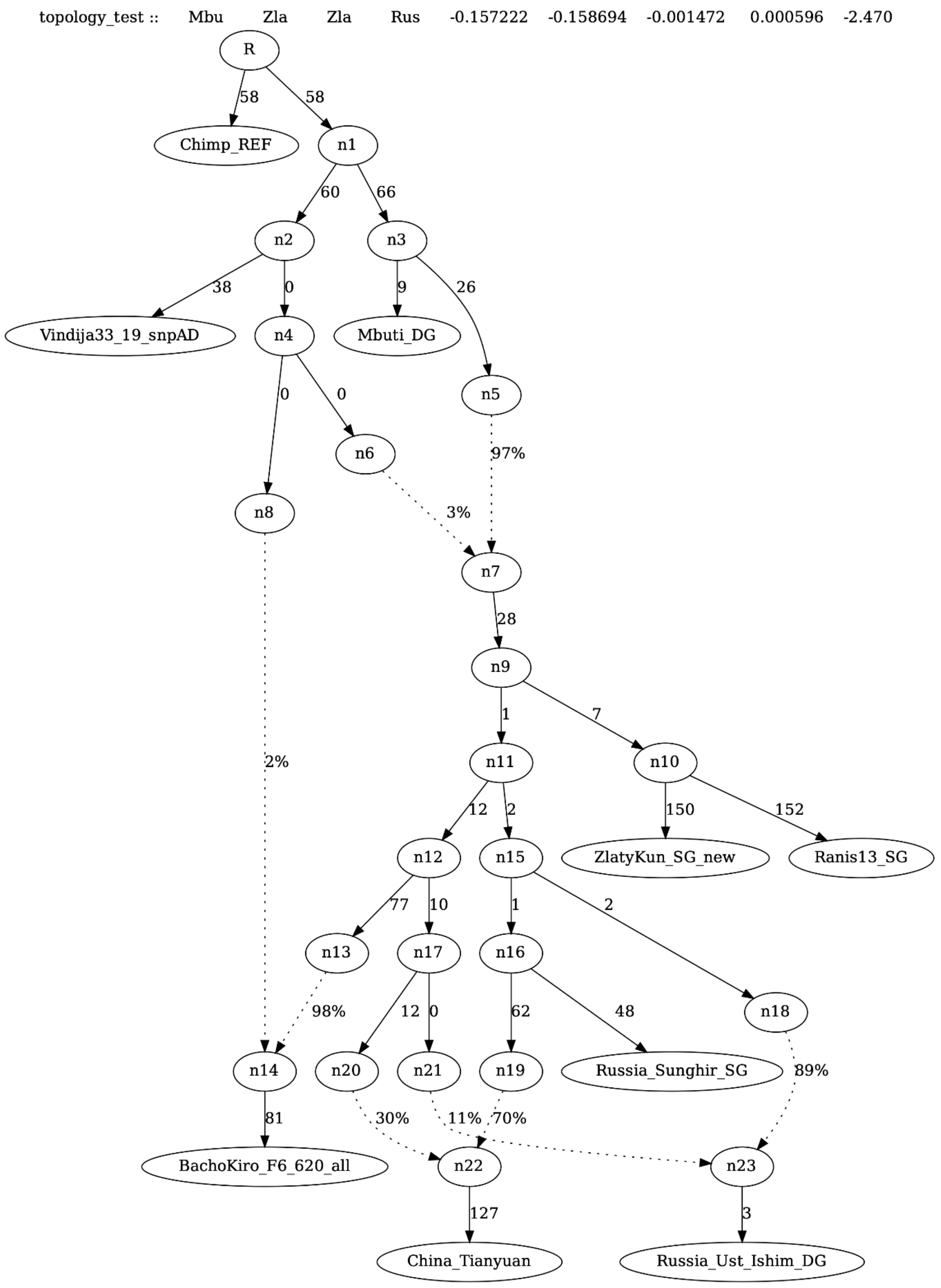

Supplementary Figure 9.11: qpGraph reconstructions including Ranis13 and Zlatý kůň individuals, together with Chimpanzee, Mbuti, Vindija33_19, Sunghir, Bacho Kiro F6 620, Tianyuan and Ust'-Ishim, worst Z-score = -2.470.

Analysis using the high coverage shotgun data

In the previous sections we have calculated statistics on the sharing of alleles based on the 1240k sites. This allowed us to include a large number of hunter-gatherer individuals for which capture datasets are available in the analysis. In this section, we use the high-coverage genomes of Ranis13 and Zlatý kůň to re-examine the previous results and expand on these analyses.

We first combined snpAD (version 0.3.11) genotype calls of the modern humans Ranis13, Zlatý kůň, Ust'-Ishim[37], Loschbour and Stuttgart (LBK)[38] with genotype calls of Vindija[31], Altai[30] and Chagryskaya[34] Neandertals and the Denisovan[21] reference genome (Denisova3). We also include the genotype calls of a present-day French, Han, Papuan, Mbuti, Yoruba and San individual (SS6004468, SS6004469, SS6004472, SS6004471, SS6004473, SS6004475)[30]. A random allele was chosen per individual at heterozygous calls and the analysis was restricted to sites that pass a recommended set of minimal filters (see Supplementary Informations 4 and 5). In addition, the base of a random overlapping sequence was added at all positions from the shotgun data of the Kostenki14[58], Sunghir3[59] and the Kotias[53] individuals. Lastly, the pairwise whole-genome alignment of the genomes of chimpanzee, bonobo, gorilla and orangutan to the human genome[60,61] were queried for the aligning base. Whenever the alignments of all four ape genomes yielded the same base, this base was considered the most likely base of the ape-ancestor. The inferred ape-ancestor was set to "N" in all other cases. The resulting table was filtered for bi-allelic sites disregarding missing bases (N) or gaps (-).

As before, we calculate D-statistics as $\frac{BABA-ABBA}{ABBA+BABA}$ and confidence-intervals based on a weighted block jackknife procedure in 5Mb windows[3,31,62,63]. The three present-day African genomes and the ape reference genomes were used as outgroups in the analysis and all combinations of the remaining individuals were tested, resulting in a total of 10,920 tests (Tables S4.10-S4.15).

We find that results based on the full genomes of Ranis13 and Zlatý kůň recapitulate earlier results based on capture data. In particular:

- We found no significant differences between Ranis13 and Zlatý kůň in their allele sharing with non-Africans.

- Ust'-Ishim shares more alleles with non-Africans than Ranis13 does (although not all comparisons are significant) with the notable exception of Zlatý kůň. Comparisons of Ust'-Ishim to Zlatý kůň yields similar results: Ranis13 is the only individual that shows more sharing with Zlatý kůň while all other comparisons with non-Africans indicate more sharing with Ust'-Ishim.

- The pairs of individuals (Han, French), (French, Papuan) and (Han, Papuan) do not differ significantly in their sharing with Zlatý kůň or Ranis13 when African outgroups are used. Note that ape outgroups yield significant signals in some comparisons of Papuans to either Han or French. These signals can be explained by additional Denisovan ancestry in Papuans which is expected to lead to significant sharing with outgroups, as shown by highly significant signals for *D(Han/French, Denisova, Zlatý kůň/Ranis13, Chimpanzee/ApeAncestor)* ($D < -65\%$; $|Z| > 140$).

We also compared Ranis and Zlatý kůň with three Neandertals that were sequenced to high-coverage. Similar to previous comparisons with out-of-Africans[3,34], we find that the approximately 50,000-year-old Vindija Neandertal from Croatia shares significantly more alleles with Zlatý kůň and Ranis13 than the ≈120,000-year-old Altai Neandertal from Siberia does. Substituting the approximately 80,000-year-old Chagyrskaya Neandertal from Siberia yields similar results (Supplementary Figure 9.12). Comparisons of Chagyrskaya and Vindija Neandertals show no significant differences, but a consistent tendency towards more sharing with the Vindija Neandertal. These results are broadly similar when the differences in sharing of Neandertals are compared to Ust'-Ishim. All results are consistent with published results that indicate a closest relationship of the introgressed Neandertal ancestry in out-of-Africans to late Neandertals[34,57].

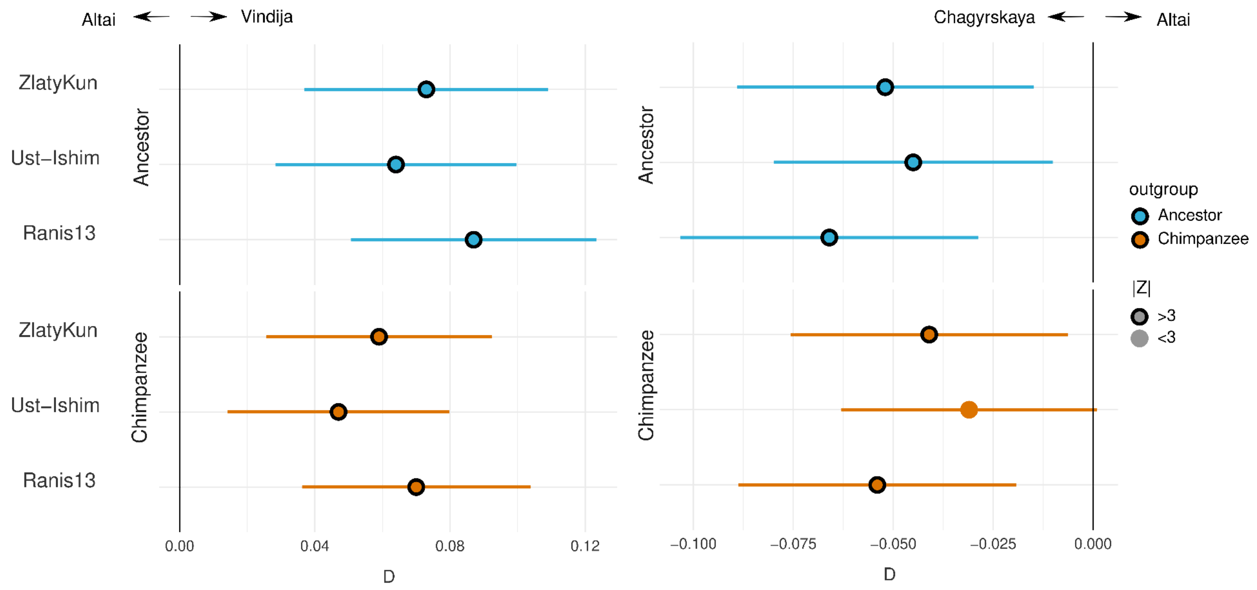

Supplementary Figure 9.12: The *D*-statistics of the form *D(Neandertal1, Neandertal2, EMH, Outgroup)*. Either the Chimpanzee or the ape-ancestor was used as an outgroup. Error bars correspond to three standard errors.

Comparison of high-coverage shotgun data with Buran Kaya III

A recent publication of two individuals from Buran Kaya III found statistical significant signals in site pattern analysis that were interpreted as evidence for a contribution of Zlatý kůň-like ancestry into the population represented by the Buran Kaya III individuals[47]. In an attempt to test this hypothesis and maximize statistical power, we merged the data from both Buran Kaya III individuals and sampled a base from all reads covering any position on human autosomes. The Buran Kaya III calls were then merged with the genotype calls from Ust'-Ishim, Ranis13, and Zlatý kůň in addition to calls from present-day Mbuti, Yoruba and San individuals and the aligned bases from the reference genomes of chimpanzee, bonobo, gorilla and orangutan. Ape ancestor sequence was inferred identical to the analysis carried out above. This combined table was then joined with Kostenki14, again by sampling a random sequence from the Kostenki14 shotgun data at every covered position. We repeated this process individually with other shotgun sequencing data from Sunghir (Sunghir1, Sunghir2, Sunghir3, Sunghir4). We then calculated a statistic of the form *D(Kostenki14/Sunghir1-4, Buran Kaya III, Zlatý kůň/Ranis13/Ust'-Ishim, Outgroup)* that is expected to yield significantly negative statistics if ancestry is shared between Buran Kaya III and the ancient HG individuals in the third position of the test to the exclusion of

Kostenki14/Sunghir1-4. In contrast to this expectation, we find positive values for *D* in 53 out of 75 comparisons. Only three comparisons yield negative values with Z < 3. These occur in comparisons *D(Sunghir1, Buran Kaya III, Zlatý kůň/Ranis13, Chimpanzee)* and *D(Sunghir4, Buran Kaya III, Ust'-Ishim, Chimpanzee)*, are of similar magnitude and are not found with closer outgroups (Supplementary Figure 9.13).

Since ancient DNA damage is a likely contributor to error in these comparisons, we restricted our analysis to transversions. None of the statistics were significantly different from 0 after this filtering was applied (Supplementary Figure 9.14). Further analysis, tables and code are available at https://doi.org/10.17617/3.0VLEOH.

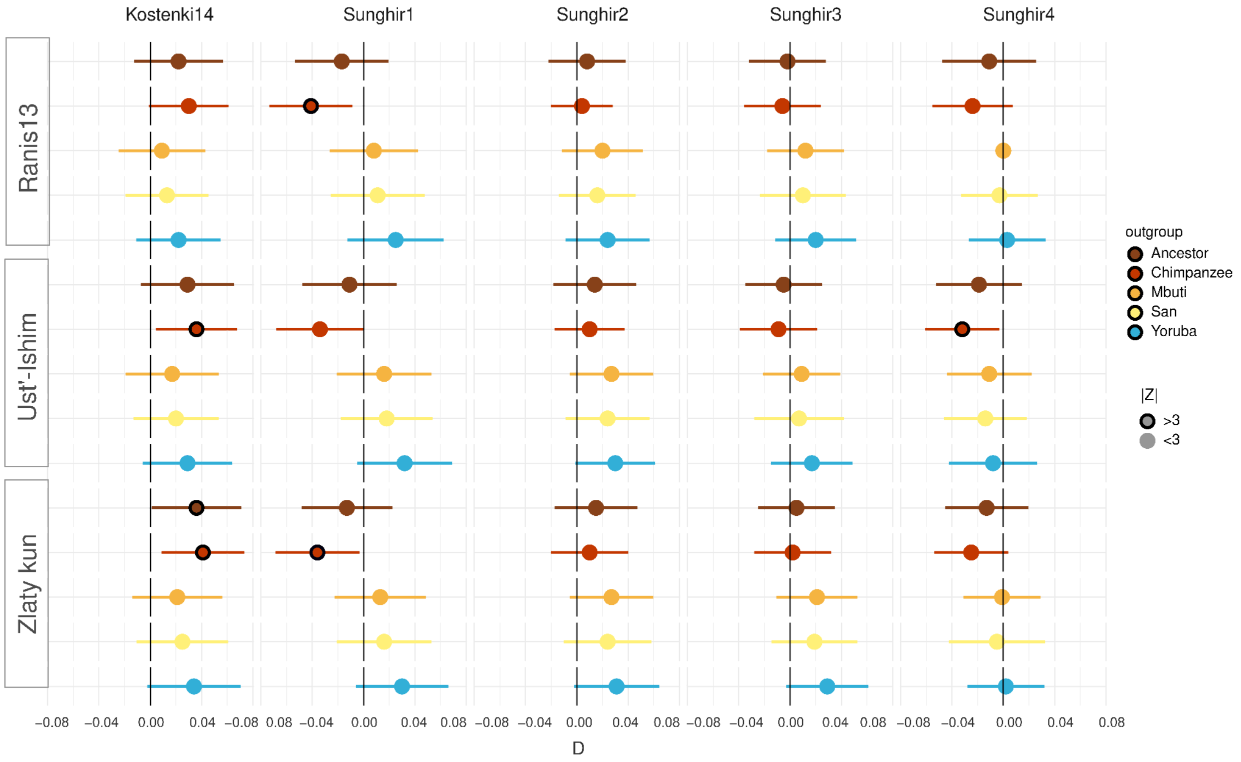

Supplementary Figure 9.13: *D*-statistics in the form of *D(Kostenki14/Sunghir1-4, Buran Kaya III, Zlatý kůň/Ranis13/Ust'-Ishim, Outgroup)* using all sites. Outgroups used for the test are colour coded, and columns and rows stand for the genomes used in each test, along with the Buran Kaya III genome. Error bars correspond to three standard errors.

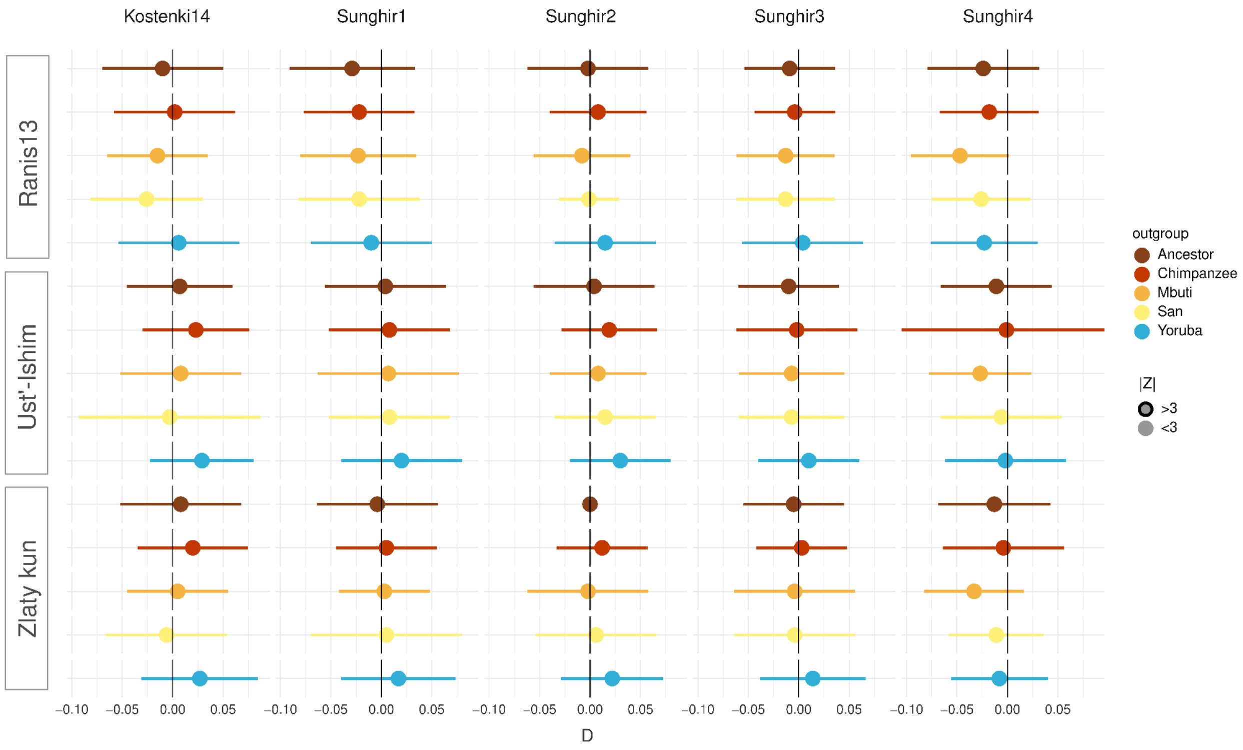

Supplementary Figure 9.14: *D*-statistics in the form of *D(Kostenki14/Sunghir1-4, Buran Kaya III, Zlatý kůň/Ranis13/Ust'-Ishim, Outgroup)* using only transversions. Outgroups used for the test are colour coded, and columns and rows stand for the genomes used in each test, along with the Buran Kaya III genome. Error bars correspond to three standard errors.

We further investigated the relationship between these groups by following a slightly different strategy, which is more similar to the one presented in Bennett et al., 2023[47]. We merged genotype calls of 263 present-day humans from the Simons Genome Diversity Project (SGDP)[39] with the genotype calls from the high-coverage hunter-gatherer genomes (Zlatý kůň, Ranis13, Ust'-Ishim[37], Loschbour[38]) and high-coverage archaic human genomes (Denisova3[21], Denisova5[30], Chagyrskaya8[34] and Vindija33.19[31]) using bcftools (version 1.4)[64]. This merged VCF file was then filtered as described in Supplementary Information 5 for all ancient genomes. We further subsetted this file to the sites that show variation, polarized all sites using the chimpanzee reference genome (*panTro4*) and kept only the biallelic positions for downstream analyses. Using an in-house script, we converted this VCF file to eigenstrat format. Next, we filtered the low-coverage genomes from the Buran Kaya III individuals and Kostenki14 for a minimum length of 35 base pairs and mapping quality of 25, and called pseudo-haploid genotypes using bam-caller (https://github.com/bodkan/bam-caller). In order to perform

D-statistics using ADMIXTOOLS, we added the genotypes from the low coverage genomes to our eigenstrat files. We repeated the calculation of the *D(Kostenki14, Buran Kaya III, Zlatý kůň/Ranis13/Ust'-Ishim, Outgroup)* statistics using ADMIXTOOLS as implemented in admixr (version 0.9.1)[65] and used Mbuti from SGDP (S_Mbuti-1, S_Mbuti-2 and S_Mbuti-3) as the outgroup. The data and code used for this analysis are available on the Max Planck Digital Library through https://doi.org/10.17617/3.EGKV28.

Comparisons between Kostenki14 and Buran Kaya III (individual BK3C) show significant deviations from zero in the opposite direction as observed by Bennett et al., 2023[47], i.e. an increased sharing between Zlatý kůň/Ranis with Kostenki14 or between Buran Kaya and the Mbuti outgroup. This signal could be explained by Basal Eurasian ancestry in the genome of Buran Kaya III (individual BK3C), although we caution that further analysis and more data will likely be necessary to substantiate this hypothesis.

Supplementary Table 9.1: The *D*-statistics in the form of *D(Kostenki14, Buran Kaya III, Zlatý kůň/Ranis13/Ust'-Ishim, Outgroup).*

W	X	Y	Z	D	stderr	Zscore	BABA	ABBA	nsnps
Kostenki14	BK3C	Zlatý kůň	Mbuti	0.0458	0.01181	3.875	3,699	3,376	208,114
Kostenki14	BK3C	Ranis13	Mbuti	0.0375	0.011648	3.217	3,633	3,371	204,964
Kostenki14	BK3C	Ust'-Ishim	Mbuti	0.0302	0.011253	2.683	3,767	3,547	209,895
Kostenki14	BK3A	Zlatý kůň	Mbuti	0.0356	0.019759	1.802	792	738	42,072
Kostenki14	BK3A	Ranis13	Mbuti	0.0368	0.019415	1.896	784	728	41,183
Kostenki14	BK3A	Ust'-Ishim	Mbuti	0.036	0.017869	2.014	830	772	42,427

Supplementary Information 10

Molecular Dating by Branch Shortening

Arev Pelin Sümer

We estimate the age of the high-coverage Ranis13 and Zlatý kůň genomes based on the number of derived variants they carry compared to present-day humans. As in previous studies[30,37], we use genotype calls based on alignments of sequences with a minimum length of 35 base pairs and filter for only transversion substitutions. The ancestral state at each position, was determined by requiring the *hg19*-aligned reference genomes of four apes (chimpanzee, bonobo, gorilla, and orangutan - *panTro4*, *bonobo*, *gorgor3*, *ponabe2*) to show the same base. In each ancient genome, we then counted the number of derived sites, i.e. called genotypes that are different from the ancestral state. We did this by randomly sampling one allele at heterozygous positions. To calibrate our estimates, we also included a present-day human genome, that of a Mbuti individual (*SS6004471*, *HGDP00982*)[30]. To estimate the age of an ancient individual, we subtracted from the count of derived sites in Mbuti the number of derived sites in the ancient individual and divided this number by the count from the Mbuti genome ($(N_{Mbuti} - N_{ancient})/N_{Mbuti}$). We then multiplied this value by the divergence time between the common ancestor of hominins and chimpanzees, which is assumed to be around 13 million years ago[30]. We applied the previously described minimal filters for all genomes (Supplementary Information 4). Only sites passing the filters for all genomes were considered further.

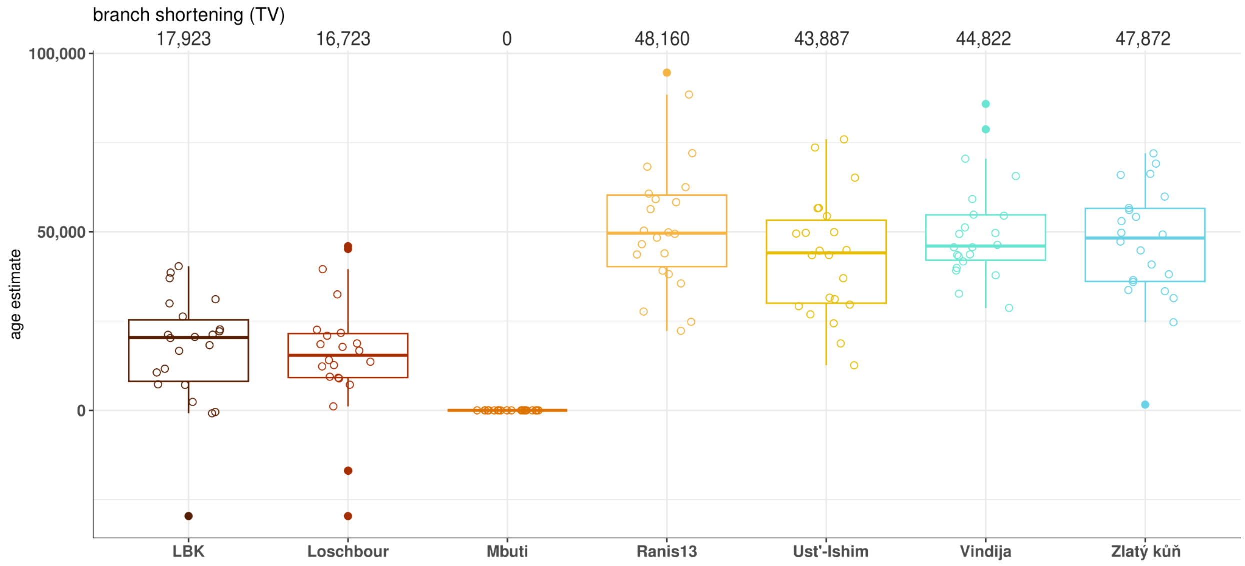

Supplementary Figure 10.1: Distribution of age estimates calculated for each autosome (n=22) using the count of missing mutations in the ancient high coverage genomes compared to a high coverage genome of a present-day Mbuti individual (*SS6004471*, *HGDP00982*)[39]. Outliers are indicated by filled dots which are excluded from estimating the weighted average age, given on the top.

We included several other high coverage genomes from direct radiocarbon dated specimens in our branch shortening analysis as controls. These include the Ust'-Ishim individual directly dated to around 44,000 years BP (44,363 calBP with 95.4% CI of 45,924 BP - 42,905 BP with IntCal20)[37], the Vindija Neandertal (Vindija33.19)[31] that is dated to 44,300 BP (95.4% CI between 45,500 and 43,100 calBP, IntCal20)[66]. LBK (also referred to as Stuttgart) is the 19x genome of an ancient farmer who lived around 7,000 years ago in present day Germany and Loschbour is the around 8,000-year-old 22x genome of a hunter-gatherer individual from the Loschbour rock shelter in Luxembourg[38].

Compared to radiocarbon dates, the branch shortening average estimates are high for the LBK and Loschbour individuals. We observed that our estimates of average age for both are around 9,000 years older than the radiocarbon dates, although the estimates vary substantially between chromosomes. Branch shortening analysis is sensitive to differences in genotype-quality since the analysis depends on small differences in derived sites, especially for the younger individuals. The Ust'-Ishim and Vindija Neandertal individuals are expected to be less sensitive since they are older and we estimate branch shortening ages from their genomes that are very close to the radiocarbon dates obtained from these specimens (Supplementary Figure 10.1)[30,34]. The Vindija

Neandertal is estimated to be older than Ust'-Ishim, as described previously[30]. Our age estimates suggest that Ranis13 and Zlatý kůň are also older than Ust'-Ishim (albeit with overlapping intervals), and contemporaneous to or slightly older than the Vindija Neandertal. These observations align well with the radiocarbon dates for Early Modern Humans and the Neandertal genome we included in this analysis, and confirm Zlatý kůň being older than 45,000 years as previously estimated using the Neandertal segments in her genome[3]. In the next section, we use the demographic curves for these genomes in an effort to estimate independent branch shortening estimates.

Supplementary Table 10.1: Number of sites used to estimate the molecular age per chromosome, and the age estimates for Ranis13 and Zlatý kůň. The values in red correspond to the outliers, that were not included in the calculation of the weighted mean age, and indicated with filled dots in Supplementary Figure 10.1.

	**Ranis13**		**Zlatý kůň**	
**Chromosome**	**Number of sites**	**Age estimate**	**Number of sites**	**Age estimate**
1	193,129	46,548	193,155	44,804
2	236,251	43,982	236,358	38,114
3	199,697	59,165	199,592	65,969
4	196,713	48,393	196,902	35,949
5	179,030	35,556	178,868	47,287
6	155,765	60,724	155,775	59,893
7	142,003	27,680	141,937	33,709
8	161,961	43,678	161,885	49,758
9	77,660	49,860	77,619	56,697
10	122,272	58,320	122,293	56,097
11	125,324	38,164	125,298	40,854
12	118,474	56,375	118,539	49,273
13	98,762	94,606	98,935	72,000
14	80,788	39,145	80,824	33,370
15	75,350	50,355	75,460	31,450
16	77,993	62,537	78,150	36,494
17	54,474	49,450	54,459	53,016
18	78,242	68,260	78,327	54,211

19	33,127	88,475	33,184	66,259
20	56,507	22,278	56,597	1,608
21	34,536	24,796	34,418	69,129
22	26,196	72,052	26,292	24,675

Supplementary Information 11

Population Size, Age and Split Time Estimates Using PSMC

Arev Pelin Sümer*, Alba Bossoms Mesa, Stéphane Peyrégne

We aimed to estimate the age of Ranis13 and Zlatý kůň and reconstruct the demographic history of the populations they belong to, using the Pairwise Sequentially Markovian Coalescent (PSMC) method[67]. Alongside, we also analyzed the previously published high coverage human genomes from Loschbour[38], Ust'-Ishim[37], and a present-day French (*SS6004468*, *HGDP00533*)[30,39] as reference.

Inference of population size changes over time

In order to run PSMC (version 0.6.5), we prepared the input files by converting the high-coverage genotypes (vcf files called using snpAD, filtered as explained in Supplementary Information 4) to the psmcfa format. This involved splitting each genome in windows of 100bp, and determining whether at least one heterozygous site was present (state K), all called sites were homozygous (state T), or more than 10% of the data was missing (state N). The psmcfa files - one for each chromosome - from the same specimen were concatenated and the sex chromosomes were excluded.

The output from PSMC (.psmc file) includes, for each of the iterations, estimates of the model parameters as well as the effective population size for each specified time interval, all scaled relative to the population size at time 0 (N0). We used the 20th iteration out of 25 to prevent overfitting[67]. As previously noticed, PSMC is sensitive to the missing data caused by filtering[31,34]. To correct the resulting biases, we followed the approach in Mafessoni et al., 2020[34], and calibrated our parameters θ (mutation rate) and ρ (recombination rate) using simulations with scrm[68]. The approach uses the following steps:

1. Creating the input from genotype calls and running PSMC (as `psmc -N25 -t15 -r5 -p "4+25*2+4+6" -o ${SAMPLE}_aut.psmc ${SAMPLE}_aut.psmcfa`), where N is the number of iterations, r is the ratio between θ/ρ and p is a string defining time intervals.

2. Creating the scrm simulation code using the output (.psmc) file and two scripts included in the PSMC suite of tools (as `psmc2history.pl ${SAMPLE}_aut.psm | history2ms.pl -L {GENOME_LENGTH} -u 1.45e-08 -g 29 | sed 's/msHOT-lite/scrm/g' | sed 's/-l//g' > ${SAMPLE}_aut_scrm.txt`).

3. Simulating whole genomes of size 2,881,033,286 bp, while varying correction factors for θ and ρ, from 1 to 2 in steps of 0.05.

4. Filtering the simulated genomes with the same filters applied to the original data, obtaining the input files for PSMC, and applying PSMC on the simulated data.

5. Running PSMC on the real data by setting the demographic history to that estimated with PSMC on the simulated data (as `psmc -N1 -i simulated_demography -o output {SAMPLE}.psmcfa`).

6. From the outputs of the previous step, we obtained the likelihood for each correction factor combination (fθ and fρ), and identified the combination that maximizes the likelihood and, therefore, the corrected demographic history that best fits the real data (Supplementary Figure 11.1 and Table 11.1).

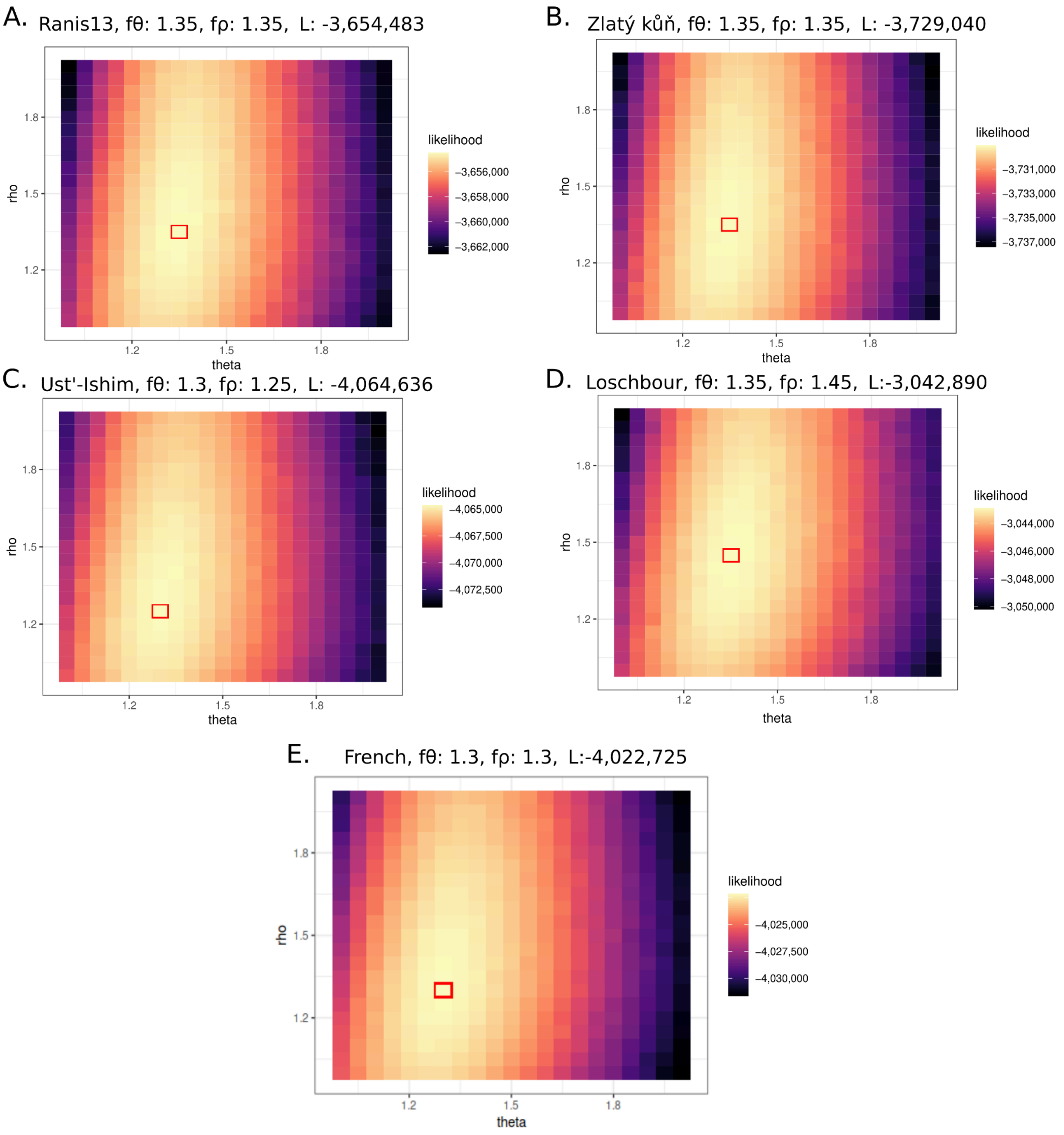

Supplementary Figure 11.1: Heatmaps representing the fit of the corrected demographic histories for Ranis13, Zlatý kůň, Ust'-Ishim, Loschbour, and French (*SS6004468*, *HGDP00533*)[39]. The colours represent the likelihood of the real data given different correction factors of the θ and ρ parameters (fθ and fρ). The squares with red borders represent the best pair of correction factors for each genome, which values are reported on top of the heatmaps together with the corresponding likelihood.

Supplementary Table 11.1: Correction factors of the θ and ρ parameters (fθ and fρ) for the genomes included in the PSMC analysis. Not all corrected demographic histories were plotted in Supplementary Figure 11.2 for simplicity. However, those inferred for San (*SS6004473*, *HGDP01036*)[39], Mbuti

(*SS6004471*, *HGDP00982*)[39], French (*SS6004468*, *HGDP00533*)[39], and Loschbour were used for the branch shortening and/or split time estimate analyses.

Specimen	f_Theta	f_Rho	Likelihood
Ranis13	1.35	1.35	-3,654,483
Zlatý kůň	1.35	1.35	-3,729,040
Ust'-Ishim	1.3	1.25	-4,064,636
Loschbour	1.35	1.45	-3,042,890
Mbuti	1.3	1.2	-5,150,129
French	1.3	1.3	-4,022,725
San	1.3	1.15	-5,212,127

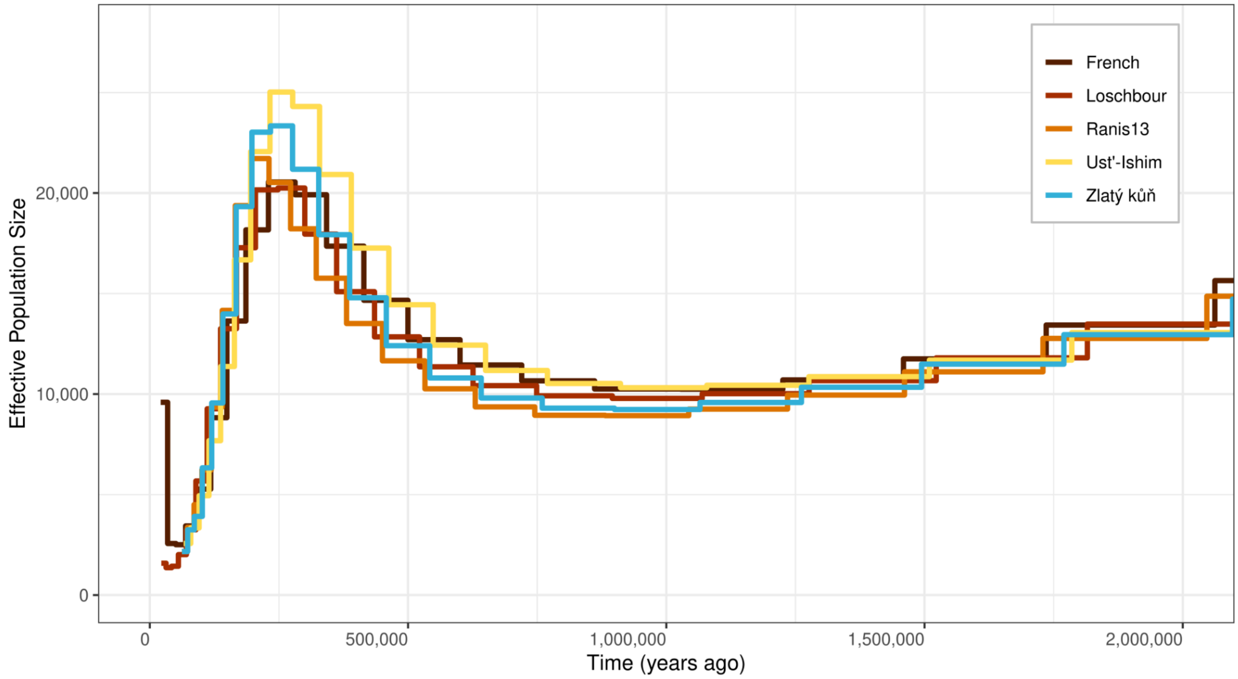

Supplementary Figure 11.2: Corrected demographic histories estimated with PSMC, assuming a mutation rate of $0.5x10^{-9}$ per year per base pair (corresponding to $1.45x10^{-8}$ per generation per base pair, for a generation time of 29 years). The curves are aligned by the age of the specimen, which are taken to be 45,000 years BP for Ranis13, Zlatý kůň, and Ust'-Ishim, and 8,000 years BP for Loschbour.

From the demographic curves we observe that all three Palaeolithic individuals lived during the bottleneck likely caused by the out-of-Africa event, and experienced drastic reduction in population size. The present-day French individual (*SS6004468*, *HGDP00533*)[39], who is included in the analysis for comparison, shows the recent population size increase that occurred

after the Neolithization. Detailed description and pipeline for this analysis are available on github (https://github.com/StephanePeyregne/calibratePSMC).

Supplementary Table 11.2: Corrected scrm codes to produce simulations of the demographic histories plotted in Supplementary Figure 11.2.

Genome	Simulation code
Ranis13	`scrm 2 1 -SC abs -p 10 -t 247520 -r 45104.7 2881033286 -en 0.1417 1 3.5540 -en 0.2325 1 2.7614 -en 0.3399 1 4.3446 -en 0.4668 1 7.4989 -en 0.6169 1 11.8110 -en 0.7944 1 15.7912 -en 1.0042 1 18.0478 -en 1.2523 1 19.0798 -en 1.5456 1 18.2970 -en 1.8925 1 15.8168 -en 2.3026 1 13.1239 -en 2.7875 1 11.0612 -en 3.3609 1 9.5900 -en 4.0388 1 8.5685 -en 4.8404 1 7.9630 -en 5.7882 1 7.7190 -en 6.9089 1 7.7673 -en 8.2340 1 8.1083 -en 9.8007 1 8.8725 -en 11.6532 1 10.2823 -en 13.8436 1 12.4599 -en 16.4335 1 15.1060 -en 19.4958 1 17.2631 -en 23.1166 1 17.9031 -en 27.3978 1 17.0664 -en 32.4599 1 15.5351 -en 45.5223 1 16.6289`
Zlatý kůň	`scrm 2 1 -SC abs -p 10 -t 264437 -r 47170.9 2881033286 -en 0.1400 1 3.2141 -en 0.2296 1 3.0669 -en 0.3352 1 3.9901 -en 0.4599 1 6.6799 -en 0.6072 1 11.7278 -en 0.7810 1 16.8411 -en 0.9861 1 19.4094 -en 1.2283 1 19.3278 -en 1.5142 1 17.8568 -en 1.8517 1 15.6032 -en 2.2501 1 13.0909 -en 2.7203 1 10.9773 -en 3.2755 1 9.4673 -en 3.9308 1 8.4525 -en 4.7044 1 7.8145 -en 5.6176 1 7.4995 -en 6.6956 1 7.4951 -en 7.9681 1 7.8280 -en 9.4703 1 8.5778 -en 11.2436 1 9.8567 -en 13.3369 1 11.6886 -en 15.8079 1 13.8286 -en 18.7249 1 15.6525 -en 22.1682 1 16.4187 -en 26.2330 1 16.0986 -en 31.0313 1 15.5317 -en 43.3819 1 17.7281`
Ust'-Ishim	`scrm 2 1 -SC abs -p 10 -t 389608 -r 61600.1 2881033286 -en 0.1029 1 1.6713 -en 0.1686 1 1.8597 -en 0.2464 1 2.8044 -en 0.3382 1 4.9495 -en 0.4466 1 8.1397 -en 0.5747 1 10.9428 -en 0.7260 1 12.3559 -en 0.9047 1 13.0335 -en 1.1158 1 12.6460 -en 1.3651 1 10.9750 -en 1.6597 1 9.1046 -en 2.0076 1 7.6185 -en 2.4186 1 6.6143 -en 2.9040 1 6.0101 -en 3.4775 1 5.6722 -en 4.1548 1 5.5308 -en 4.9549 1 5.5755 -en 5.9000 1 5.8332 -en 7.0163 1 6.3723 -en 8.3350 1 7.3060 -en 9.8926 1 8.6948 -en 11.7325 1 10.3341 -en 13.9058 1 11.6892 -en 16.4730 1 12.2752 -en 19.5053 1 12.0909 -en 23.0873 1 11.5810 -en 32.3161 1 14.9450`
Loschbour	`scrm 2 1 -SC abs -p 10 -t 1.11598e+06 -r 225929 2881033286 -en 0.0245 1 0.2395 -en 0.0404 1 0.2893 -en 0.0592 1 0.3252 -en 0.0816 1 0.4459 -en 0.1082 1 0.6964 -en 0.1398 1 1.1829 -en 0.1774 1 2.0122 -en 0.2222 1 3.0057 -en 0.2753 1 3.7760 -en 0.3385 1 4.0545 -en 0.4136 1 3.8697 -en 0.5029 1 3.4645 -en 0.6091 1 3.0140 -en 0.7353 1 2.5937 -en 0.8854 1 2.2684 -en 1.0638 1 2.0510 -en 1.2759 1 1.9290 -en 1.5280 1 1.8901 -en 1.8277 1 1.9180 -en 2.1840 1 2.0200 -en 2.6076 1 2.2486 -en 3.1112 1 2.6723 -en 3.7098 1 3.2937 -en 4.4215 1 3.9198 -en 5.2676 1 4.1980 -en 6.2735 1 3.8993 -en 8.8908 1 4.8223`
French (*SS6004468*, *HGDP00533*)	`scrm 2 1 -t 50741556.3204465 -r 8862118.35046735 2881033286   -en 0.0006 1 0.0231 -en 0.0010 1 0.0082 -en 0.0014 1 0.0079 -en 0.0019 1 0.0113 -en 0.0025 1 0.0196 -en 0.0033 1 0.0337 -en 0.0041 1 0.0502 -`

	en 0.0052 1 0.0622 -en 0.0064 1 0.0668 -en 0.0078 1 0.0637 -en 0.0095 1 0.0554 -en 0.0115 1 0.0470 -en 0.0139 1 0.0409 -en 0.0167 1 0.0368 -en 0.0200 1 0.0341 -en 0.0240 1 0.0327 -en 0.0287 1 0.0325 -en 0.0342 1 0.0338 -en 0.0407 1 0.0368 -en 0.0484 1 0.0424 -en 0.0576 1 0.0509 -en 0.0684 1 0.0614 -en 0.0812 1 0.0698 -en 0.0964 1 0.0722 -en 0.1143 1 0.0697 -en 0.1355 1 0.0674 -en 0.1903 1 0.1994
San (*SS6004473*, *HGDP01036*)	scrm 2 1 -SC abs -p 10 -t 3.48943e+06 -r 470238 2881033286 -en 0.0164 1 0.7845 -en 0.0268 1 1.0546 -en 0.0389 1 1.1066 -en 0.0532 1 1.2858 -en 0.0699 1 1.3674 -en 0.0895 1 1.3598 -en 0.1125 1 1.3550 -en 0.1394 1 1.2566 -en 0.1709 1 1.0790 -en 0.2079 1 0.9088 -en 0.2513 1 0.7804 -en 0.3021 1 0.6895 -en 0.3616 1 0.6311 -en 0.4315 1 0.6016 -en 0.5133 1 0.5961 -en 0.6092 1 0.6119 -en 0.7217 1 0.6536 -en 0.8535 1 0.7335 -en 1.0079 1 0.8657 -en 1.1890 1 1.0476 -en 1.4013 1 1.2353 -en 1.6501 1 1.3574 -en 1.9417 1 1.3789 -en 2.2835 1 1.3344 -en 2.6842 1 1.2977 -en 3.1539 1 1.4693 -en 4.3498 1 5.0458
Mbuti (*SS6004471*, *HGDP00982*)	scrm 2 1 -SC abs -p 10 -t 3.26666e+06 -r 474753 2881033286 -en 0.0172 1 0.5575 -en 0.0280 1 0.8056 -en 0.0408 1 0.9256 -en 0.0557 1 1.1389 -en 0.0733 1 1.3670 -en 0.0938 1 1.4694 -en 0.1180 1 1.4534 -en 0.1463 1 1.3397 -en 0.1795 1 1.1608 -en 0.2184 1 0.9714 -en 0.2641 1 0.8267 -en 0.3177 1 0.7323 -en 0.3806 1 0.6739 -en 0.4544 1 0.6430 -en 0.5409 1 0.6375 -en 0.6424 1 0.6570 -en 0.7615 1 0.7043 -en 0.9012 1 0.7875 -en 1.0650 1 0.9179 -en 1.2572 1 1.0974 -en 1.4827 1 1.2915 -en 1.7472 1 1.4320 -en 2.0575 1 1.4771 -en 2.4214 1 1.4476 -en 2.8483 1 1.4089 -en 3.3491 1 1.5570 -en 4.6258 1 5.2190

Inference of population size changes over time corrected for the effect of background selection

Demographic inferences are affected by background selection[69,70]. To correct for the effect of background selection on the shape of the demographic curves, we removed all regions with evidence of more than 10% reduction in genetic variation due to background selection (B-score<900)[71], and repeated the pipeline described above, including the calibration of the θ (mutation rate) and ρ (recombination rate) parameters to avoid biases caused by missing data (Supplementary Table 11.3, Supplementary Figure 11.3).

Supplementary Table 11.3: Correction factors of the θ and ρ parameters (fθ and fρ) for the genomes included in this part investigating the effect of background selection.

Specimen	f_Theta	f_Rho	Likelihood
Ranis13	1.35	1.95	-1,589,645

Zlatý kůň	1.4	1.95	-1,593,884
Ust'-Ishim	1.35	1.8	-1,739,338
Loschbour	1.35	2	-1,323,639

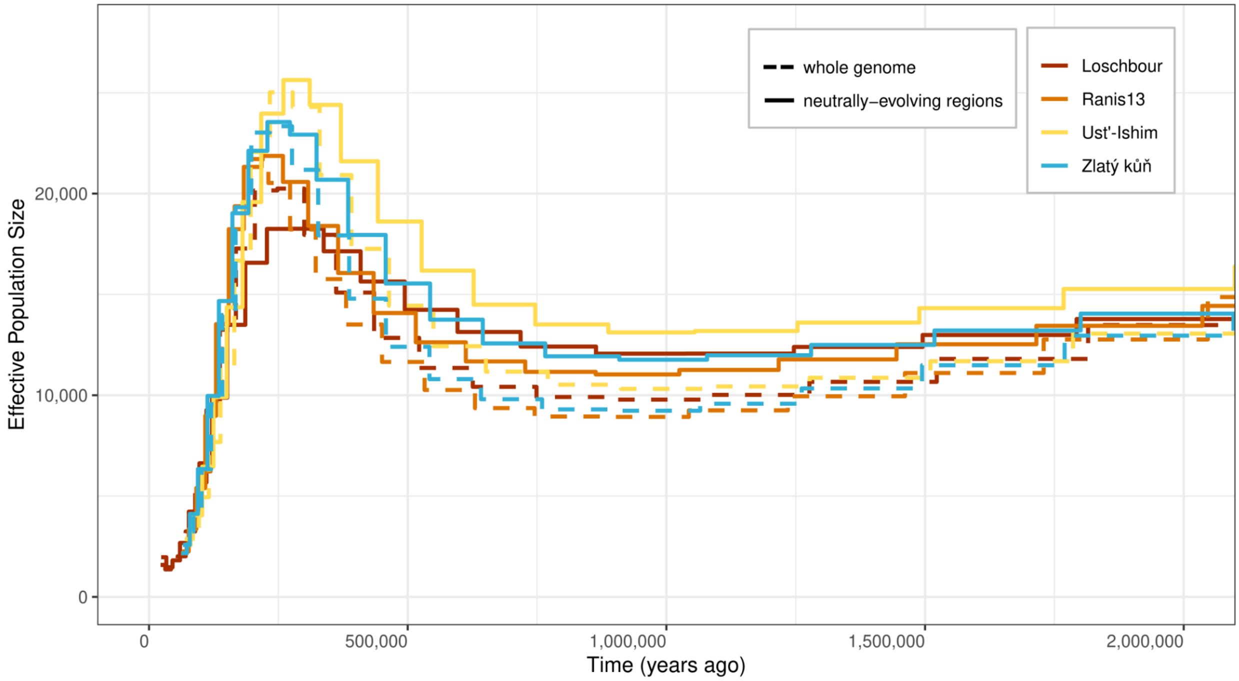

Supplementary Figure 11.3: Corrected demographic histories estimated with PSMC, assuming a mutation rate of 0.5×10^{-9} per year per base pair (corresponding to 1.45×10^{-8} per generation per base pair, for a generation time of 29 years). The curves are also aligned by the age of the specimen, which are taken to be 45,000 years BP for Ranis13, Zlatý kůň, and Ust'-Ishim, and 8,000 years BP for Loschbour. Dashed lines indicate demographic curves obtained using whole genomes, and solid lines only the genomic regions that presumably evolve neutrally.

Age estimates using demographic histories

We used the demographic histories inferred by PSMC to estimate the ages of our Palaeolithic individuals, following the approach in Mafessoni et al., 2020, SI Appendix 6[34]. For this, we compared the demographic history reconstructed from the genome of a present-day French individual (*SS6004468*, *HGDP00533*)[39] or the 8,000-year-old Loschbour individual to the demographic history reconstructed from the genomes of the Palaeolithic individuals to estimate the age difference between these two groups.

We first truncated the corrected demographic histories of the French or Loschbour individuals obtained in the previous step (Supplementary Figure and Table 11.1), by 0 to 162,429 years in steps of 5,075 years, and, each time, simulated ten whole genomes following that truncated demographic history. The simulated genomes were then filtered identically to the genome of the Palaeolithic individual under consideration. We ran PSMC on these filtered genomes, obtained a new demographic history, which takes into account the effects of the filters applied to the genome of the Palaeolithic individual, and evaluated the fit of this demography to the genome of that Palaeolithic individual, by computing the likelihood with the command “psmc -N1 -i {input.demography} -o {output} {input.data}”. Lastly, we identified the truncated demographic history of the French or Loschbour individual that best fits the genome of the Palaeolithic individual (with the highest likelihood), allowing us to estimate the best fitting time difference between the two individuals. We plotted estimates from all simulations in Supplementary Figure 11.4, and the yellow intervals correspond to the top 5% simulations with the highest likelihood.

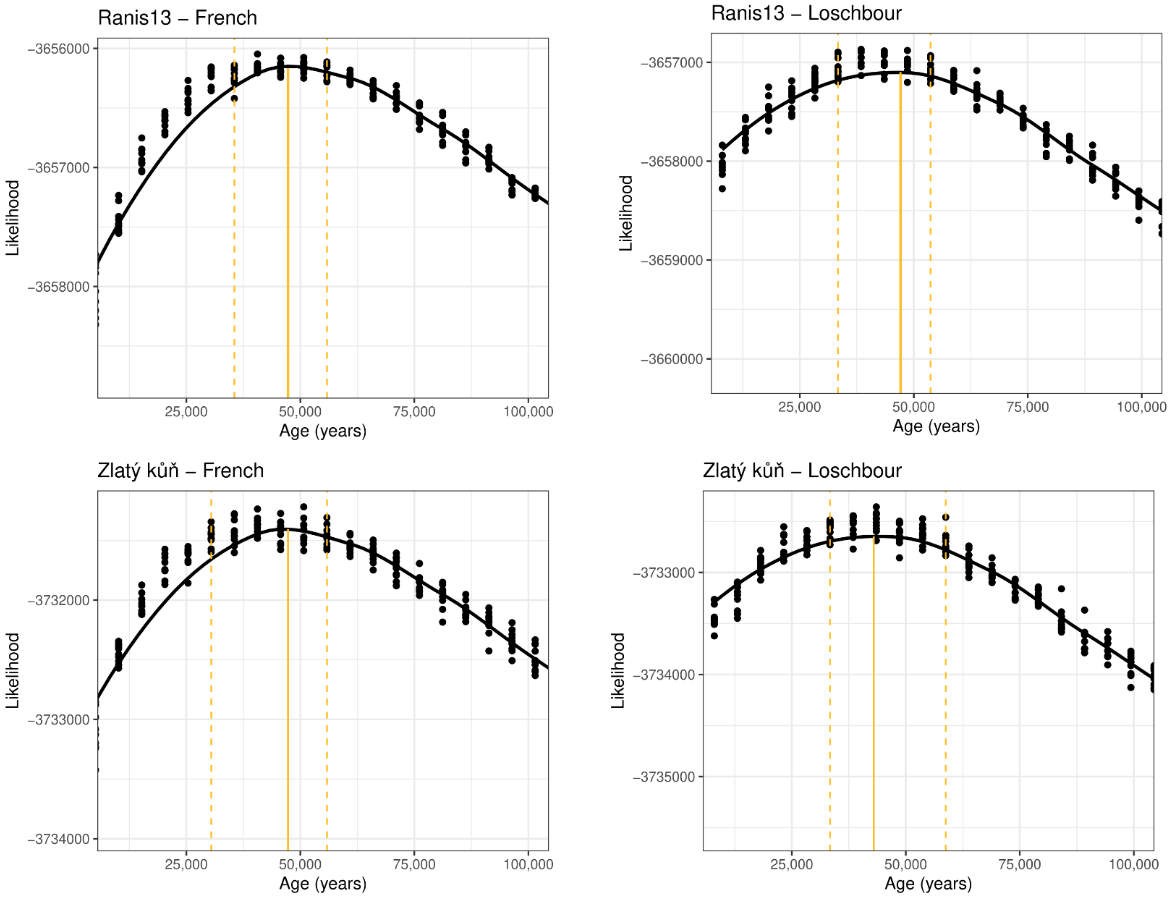

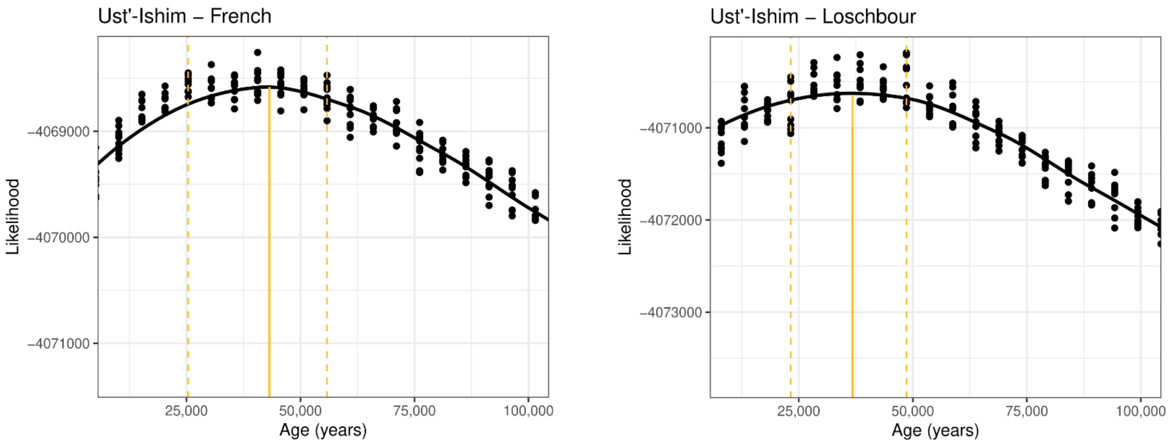

Supplementary Figure 11.4: Age estimates for Ranis13, Zlatý kůň, and Ust'-Ishim, using the demographic curves obtained by PSMC. Estimates are based on assuming an age of 0 for the present-day French individual (*SS6004468*, *HGDP00533*)[39], 8,000 years BP for the Loschbour individual, and 29 years as generation time. Intervals in yellow dashed lines indicate estimates from simulations with the best likelihood (top 5%), and the solid line indicates the peak of the distribution. For each time point, n=10 corresponding to the ten simulations ran to obtain these values.

The age estimates for Ranis13, Zlatý kůň, and Ust'-Ishim agree with our previous estimates from branch shortening. They are also in line with radiocarbon dating estimates for Ranis13 and Ust'-Ishim. While radiocarbon dating for Zlatý kůň failed likely due to treatment of the skull with animal glue[3], our age estimates here are in line with the previous estimates obtained using the length distribution of the Neandertal segments in the low coverage genome of Zlatý kůň, indicating this individual lived likely before 45,000 years BP. We note, however, that age estimates based on inferred PSMC demography have large uncertainty (Supplementary Table 11.4).

Supplementary Table 11.4: Branch shortening estimates at the peak of the distribution plotted in Supplementary Figure 11.4, and corresponding likelihoods. In the figure, minimum and maximum values, corresponding to the estimates from simulations with the highest likelihood (top 5%), are those indicated by the dashed lines.

**Genomes used**		**Estimated age for the focus (G*old*) genome**			
**G*old***	**G**	**Peak of the distribution**	**Likelihood**	**Minimum**	**Maximum**
Ranis13	French	47,281	-3656151	35,525	55,825
Ranis13	Loschbour	47,058	-3,657,102	33,375	53,675
Zlatý kůň	French	47,281	-3,731,407	30,450	55,825
Zlatý kůň	Loschbour	42,947	-3,732,647	33,375	58,750
Ust'-Ishim	French	43,170	-4,068,582	25,375	55,825

Genomes used		Estimated age for the focus (G*old*) genome			
G*old*	G	Peak of the distribution	Likelihood	Minimum	Maximum
Ranis13	French	47,281	-3656151	35,525	55,825
Ranis13	Loschbour	47,058	-3,657,102	33,375	53,675
Ust'-Ishim	Loschbour	36,780	-4,070,626	23,225	48,600

Split time estimates

We used the F(A|B) statistic to estimate the split times between the three high-coverage early Modern Human genomes and the present-day French (*SS6004468*, *HGDP00533*)[39], San (*SS6004473*, *HGDP01036*)[39], and Mbuti (*SS6004471*, *HGDP00982*)[39] genomes. This statistic corresponds to the proportion of derived alleles in a pseudo-haploid genome from population A, where a genome from population B is heterozygous. F(A|B) measures time in the history of population B since the split time from population A, as new mutations that occur since the split time in B decrease the value of F(A|B). The value of F(A|B) is highest if the two genomes belong to individuals from the same population, and decreases with increasing divergence if they belong to different populations. Following previous analyses, we used only transversions and defined the ancestral states as those shared by four ape reference genomes (*panTro4*, *bonobo*, *gorGor3*, *ponAbe2*). To estimate the value of the F(A|B) statistic that is expected for a given population split time, we used coalescent simulations as listed in Supplementary Table 11.2, corresponding to the corrected demographic histories obtained from the PSMC analysis. In order to scale time in thousands of years (ka), we used a mutation rate of 1.45×10^{-8} per base pair per generation and a generation time of 29 years. We sampled genomes from these simulations from various time points after the population split, from 0 to 200,000 years with steps of 1,000 years, and from 200,000 years to 800,000 years with steps of 5,000 years. We calculated F(A|B) statistics for each simulation, and estimated an expected value with 10 replicates for each time point. We then used these values to obtain a calibration curve and fitted the observed value of F(A|B) to this curve, in order to estimate the split times.

For each of the high coverage Early Modern Human genomes, we calculated the split times with present-day San (*SS6004473*, *HGDP01036*)[39], Mbuti (*SS6004471*, *HGDP00982*)[39] and French (*SS6004468*, *HGDP00533*)[39] individuals (Supplementary Figures 11.5, 11.6 and 11.7). We

alternated the genomes in position A and B in the F(A|B) statistics, hence obtained two estimates for each split time. Although the two estimates were similar, the confidence intervals did not always overlap (Supplementary Table 11.5). This suggests unaccounted uncertainties in the demographic histories or F(A|B) values, which may be sensitive to errors in the heterozygous positions of genome B. Our assessments of split times yielded values similar to and in the range of those previously reported[37,72,73]. We were not able to estimate the split time between the three high coverage Early Modern Human genomes with this method due to the high uncertainty of PSMC curves in the most recent times (i.e. demographic histories close to the individuals' lifetime)[67,74].

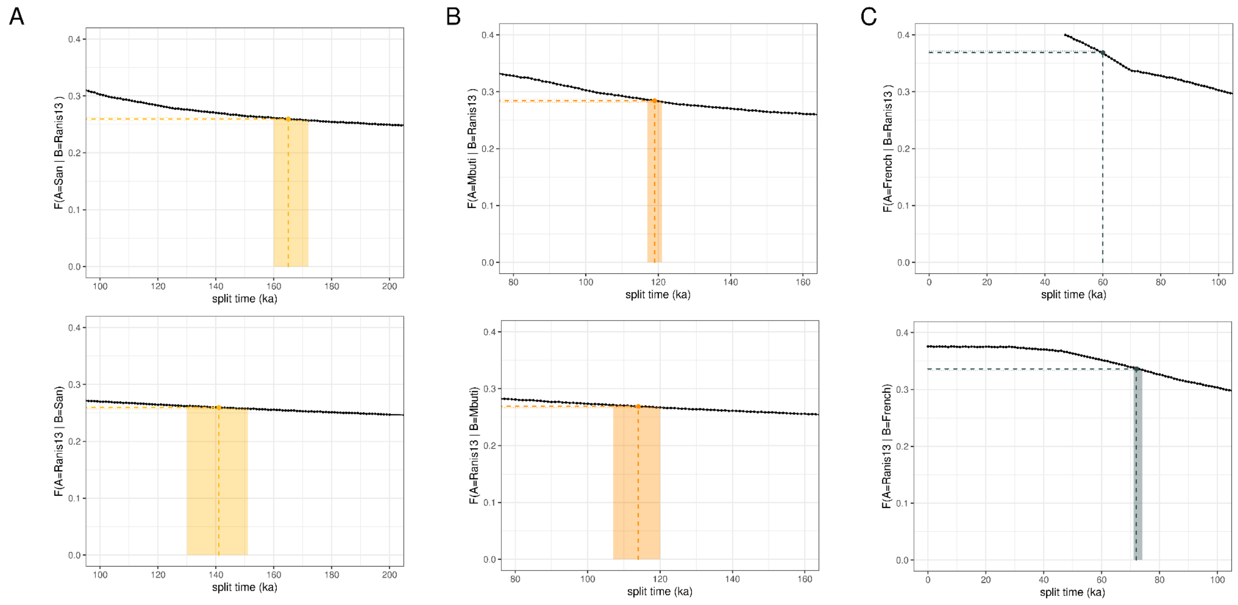

Supplementary Figure 11.5: Split time estimates for Ranis13 and the present-day San (*SS6004473*, *HGDP01036*)[39], Mbuti (*SS6004471*, *HGDP00982*)[39] and French (*SS6004468*, *HGDP00533*)[39] individuals. Top row shows Ranis13 as individual B in the F(A|B) calculations, and bottom row is Ranis13 as individual A. Standard errors (SE) are estimated using block jackknife using a window size of 5Mb, and CI is two SE of the mean.

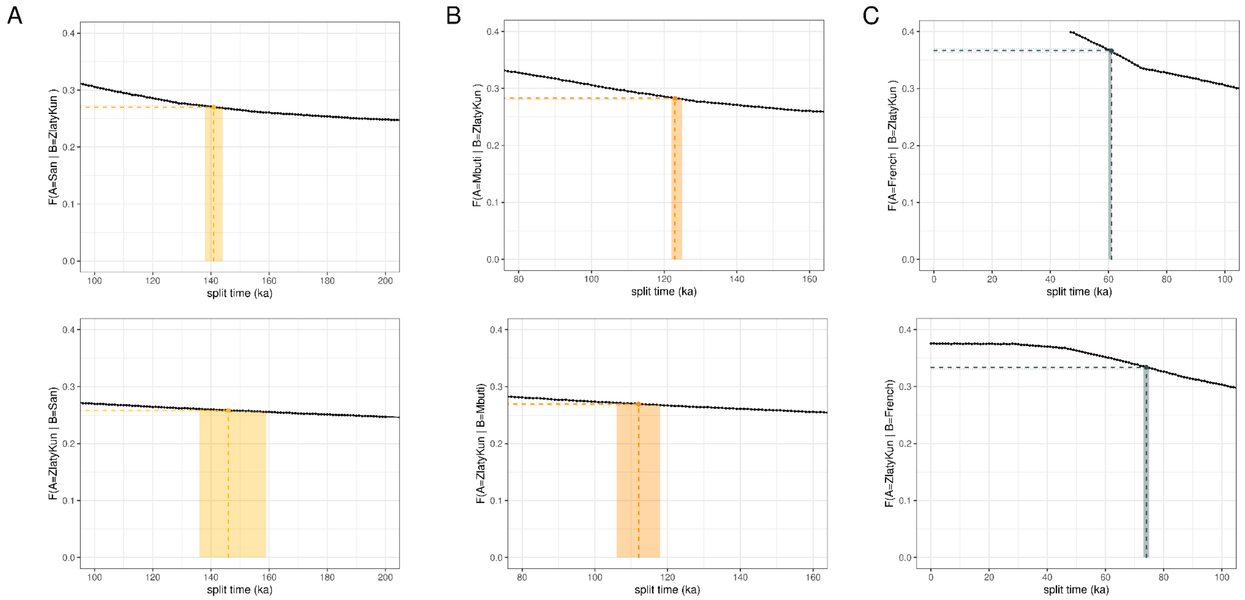

Supplementary Figure 11.6: Split time estimates for Zlatý kůň and the present-day San (*SS6004473*, *HGDP01036*)[39], Mbuti (*SS6004471*, *HGDP00982*)[39] and French (*SS6004468*, *HGDP00533*)[39] individuals. Top row shows Zlatý kůň as individual B in the F(A|B) calculations, and bottom row is Zlatý kůň as individual A. Standard errors (SE) are estimated using block jackknife using a window size of 5Mb, and CI is two SE of the mean.

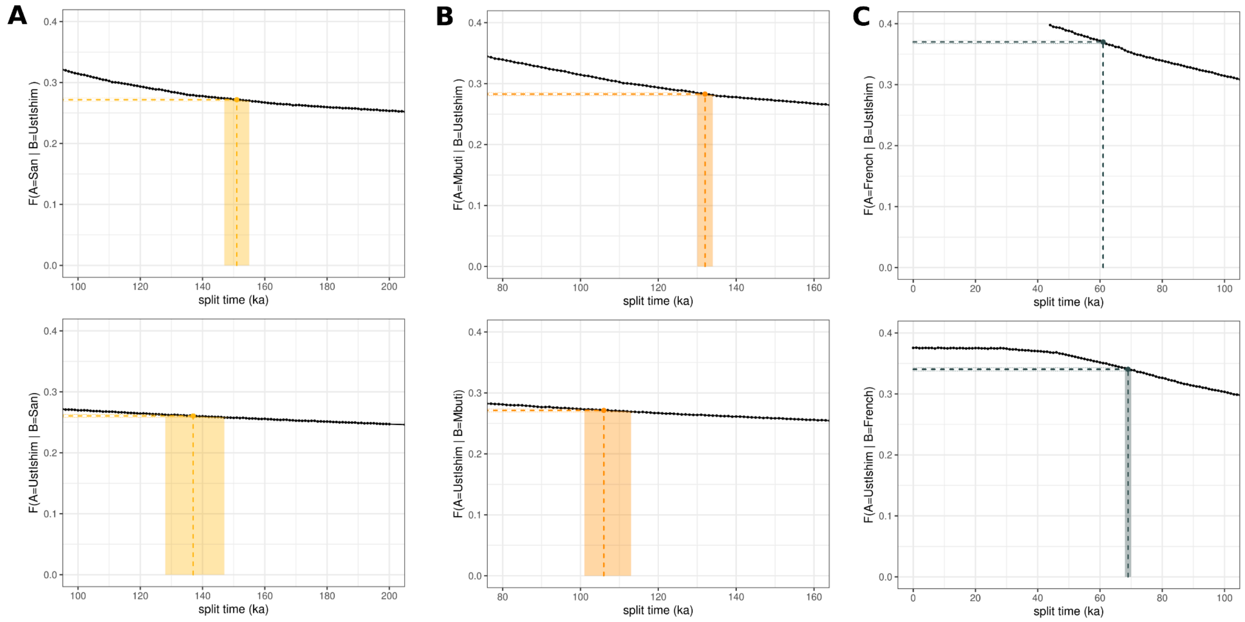

Supplementary Figure 11.7: Split time estimates for Ust'-Ishim and the present-day San (*SS6004473*, *HGDP01036*)[39], Mbuti (*SS6004471*, *HGDP00982*)[39] and French (*SS6004468*, *HGDP00533*)[39] individuals. Top row shows Ust'-Ishim as individual B in the F(A|B) calculations, and the bottom row is Ust'-Ishim as individual A. Standard errors (SE) are estimated using block jackknife using a window size of 5Mb, and CI is two SE of the mean.

Supplementary Table 11.5: Results of the F(A|B) statistics plotted above for different pairs of genomes along with the number of positions used for calculating the estimates.

Genomes used		F(A|B)		Number of positions		Split time estimates	
**A**	**B**	**stats**	**se**	**ancestral**	**derived**	**min.**	**max.**
Mbuti	Ranis13	2.84E-01	1.27E-03	215,756	85,673	116,000	122,000
Ranis13	Mbuti	2.70E-01	1.24E-03	299,563	110,737	106,000	121,000
Mbuti	Zlatý kůň	2.83E-01	1.21E-03	218,556	86,148	121,000	126,000
Zlatý kůň	Mbuti	2.70E-01	1.22E-03	307,681	113,522	105,000	119,000
Mbuti	Ust'-Ishim	2.83E-01	1.20E-03	234,811	92,601	129,000	135,000
Ust'-Ishim	Mbuti	2.71E-01	1.19E-03	322,021	119,863	100,000	114,000
San	Ranis13	2.71E-01	1.31E-03	220,863	82,079	159,000	173,000
Ranis13	San	2.60E-01	1.23E-03	309,277	108,393	132,000	150,000
San	Zlatý kůň	2.70E-01	1.29E-03	223,335	82,684	135,000	160,000
Zlatý kůň	San	2.58E-01	1.23E-03	318,105	110,736	137,000	145,000
San	Ust'-Ishim	2.72E-01	1.31E-03	239,344	89,352	146,000	156,000
Ust'-Ishim	San	2.61E-01	1.20E-03	332,019	116,986	127,000	148,000
French	Ranis13	3.69E-01	1.44E-03	191,665	111,982	58,000	60,000
Ranis13	French	3.36E-01	1.37E-03	205,644	104,233	70,000	75,000
French	Zlatý kůň	3.67E-01	1.50E-03	194,134	112,327	59,000	62,000
Zlatý kůň	French	3.34E-01	1.40E-03	211,598	106,126	72,000	76,000
French	Ust'-Ishim	3.70E-01	1.35E-03	207,322	121,750	60,000	62,000
Ust'-Ishim	French	3.40E-01	1.35E-03	220,560	113,843	67,000	71,000

Supplementary Information 12

Demographic History Inference

Fabrizio Mafessoni

To investigate the relationship of Ranis13 and Zlatý kůň with other out-of-Africa populations, we jointly modeled their demographic history and relationship with other populations with momi2[75], which optimizes the composite-likelihood of observing a given site frequency spectrum for a set of populations. To this goal we used the genomes of present-day populations from the SGDP dataset (Tuscani, Yorubas and Han Chinese, as representatives of European, African, and Asian populations, respectively, and Papuans), ancient modern humans (Ranis13, LBK[38], Loschbour[38], Zlatý kůň, Ust'-Ishim[37]), and archaic hominins (a Neandertal (Vindija33.19[31]) and a Denisovan (Denisova3)[21]). We parsed the genotype calls from these genomes with the script momi.extract_sfs from the momi2 suite to compute the site frequency spectrum, retaining only regions which passed quality filters for all genomes included in each of the models described below.

To investigate whether the genomes of Ranis13 and Zlatý kůň represented an early out-of-Africa event preceding that of Ust'-Ishim, we used a model (core model) including Yorubas, Tuscani, Han Chinese, Neandertals, Ust'-Ishim, Ranis13, and Zlatý kůň, but excluding Papuans and Denisovans. Neandertal introgression into the out-of-Africa population was modeled by adding a ghost (unsampled) introgressing Neandertal population, whose split time from Vindija33.19 as well as the proportion of introgression were estimated in the model. We optimized this model using a stochastic optimization procedure, implemented in the function stochastic_optimize, and ran 100 bootstraps to obtain 5% confidence intervals, using the same procedure as in Mafessoni et al., 2020[34]. Tip dates were fixed in the model for ancient modern humans (i.e. 45,000 years ago for Ranis13, Ust'-Ishim, and Zlatý kůň), but were allowed to vary between 45,000 and 55,000 years ago for Vindija33.19. In this model (Supplementary Figure 12.1) we observe estimates generally complying with literature, including a 2.3% (2.0-2.6%) admixture from Neandertals to out-of-Africa populations, estimated to have occurred 49,400 years before present (BP) (47,900-52,100 years BP) from a Neandertal population separating 58,900 years BP (53,100-69,500 years BP) from Vindija33.19 (Supplementary Table 12.1). We estimate a small

recent effective population size for Ranis13 (Ne = 184, 50-716). We estimate that the separation of Ranis13 and Zlatý kůň from the ancestor of all out-of-Africa populations occurred 48,000 years BP (46,900-51,300 years BP) which overlaps the point estimate of the time of separation of Ust'-Ishim 47,200 years BP. Note however that despite the small difference in time, the time of separation of Ranis13 and Zlatý kůň is significantly older than that of Ust'-Ishim, (p-value = 0.03, i.e. in 97 out of 100 bootstraps Ust'-Ishim appeared to separate later than Ranis13, owing to the strong correlation between the estimates of the two split times; Supplementary Figure 12.2).

We then implemented a second model to assess: i) the robustness of our previous estimates to different models and the inclusion of different genomes, ii) the relationships between Ranis13 and other ancient modern humans as well as Papuans, which have been claimed to derive parts of their ancestry from an earlier out-of-Africa event[3]. To do this, we added Papuans as well as Denisovans to the set of populations analyzed, and we replaced present-day Tuscani with the genome of a ~8,000-year-old ancient hunter-gatherer (Loschbour)[38] and a ~7,000-year-old ancient farmer (LBK Stuttgart)[38] (Supplementary Figure 12.3). Tip dates were fixed to 8,000 and 7,000 years ago, respectively. In this second model we confirmed the earlier split of the clade Ranis13/Zlatý kůň compared to Ust'-Ishim (p-value = 0.04) (Supplementary Figure 12.4), with a similar small estimated difference in split times (Supplementary Figure 12.3, Supplementary Table 12.2). Regarding Papuans, we find support for an earlier split of Ranis13 and Zlatý kůň compared to Papuans (p-value = 0.03) of about 6,000 years. Note however that this model might not be able to capture all the complex events that lead to the origin of Papuans. In particular, very low proportions of ancestry from an early out-of-Africa population could be missed due to the limited number of individuals that we can use to build the site frequency spectrum with momi2. Such ancestry has been claimed to account for ~1% of the genome of Papuans[76]. Thus, we implemented a more complex model imposing an early split of Papuans, followed by a substantial gene flow from populations participating in a later out-of-Africa event. Under this model, we estimate that the majority (76.6%) of the current genomic composition of Papuans come from an out-of-Africa event which originated from a population separating about 30,000 years ago, about 20,000 years after the separation of Ranis13 and Zlatý kůň from other out-of-Africa populations (Supplementary Figure 12.5, Supplementary Figure 12.6). While considerable uncertainty exists in interpreting the complex demography that led to the present

genomic composition of Papuans, our model suggests that Ranis13 and Zlatý kůň represent the earliest genomes of an out-of-Africa modern human, though we cannot exclude conclusively that a small proportion of the genome of Papuans carry additional components.

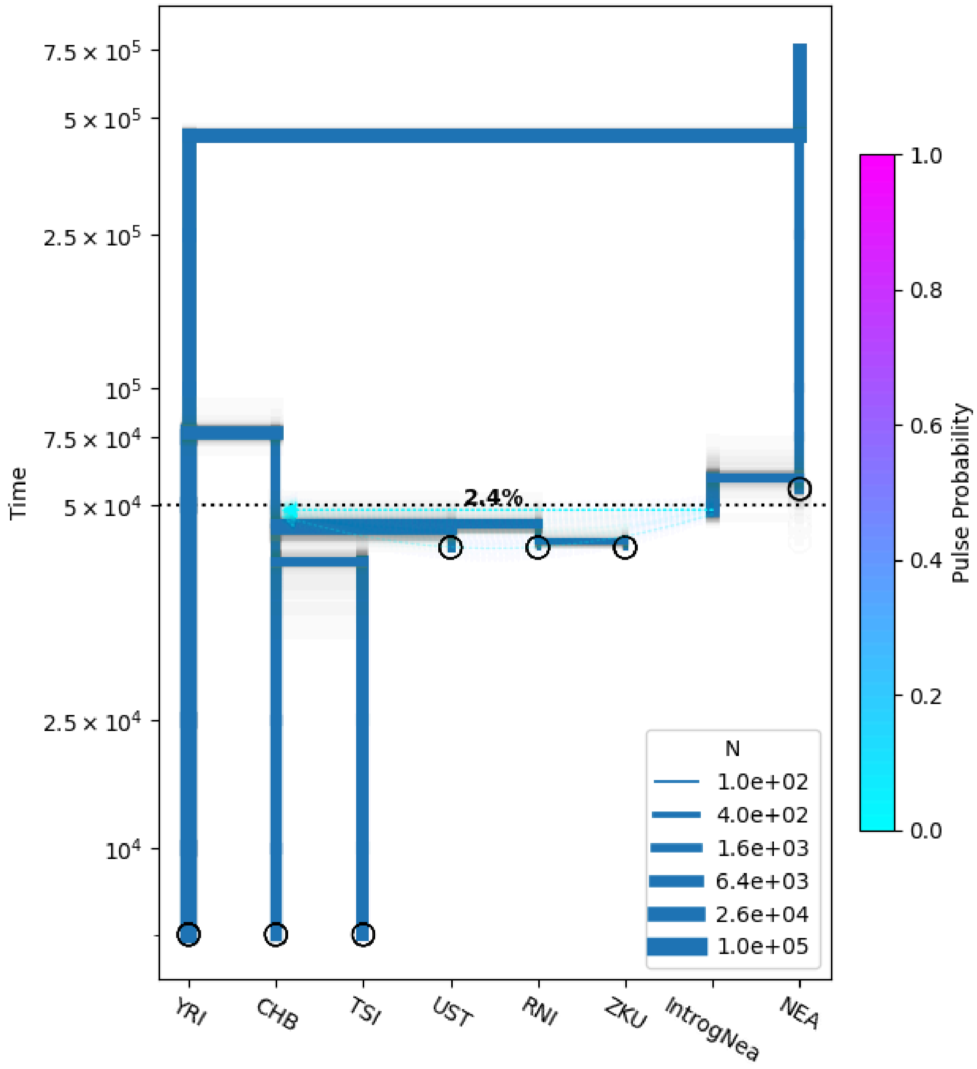

Supplementary Figure 12.1: Model estimates from momi2 for the core model in which Yorubas (YRI), Han Chinese (CHB), Tuscani (TSI), Ust'-Ishim (UST), Ranis13 (RNI), Zlatý kůň (ZKU), Vindija33.19 (NEA), and an introgressing Neandertal population (IntrogNea) are included. Shadows indicate estimates from individual bootstrap (resamplings of the site frequency spectrum) iterations. The thickness of blue lines indicates the estimated effective population sizes as described in the legend. The colour of the arrow indicates the proportion of admixture as indicated in the legend. The y-axis indicates time in years.

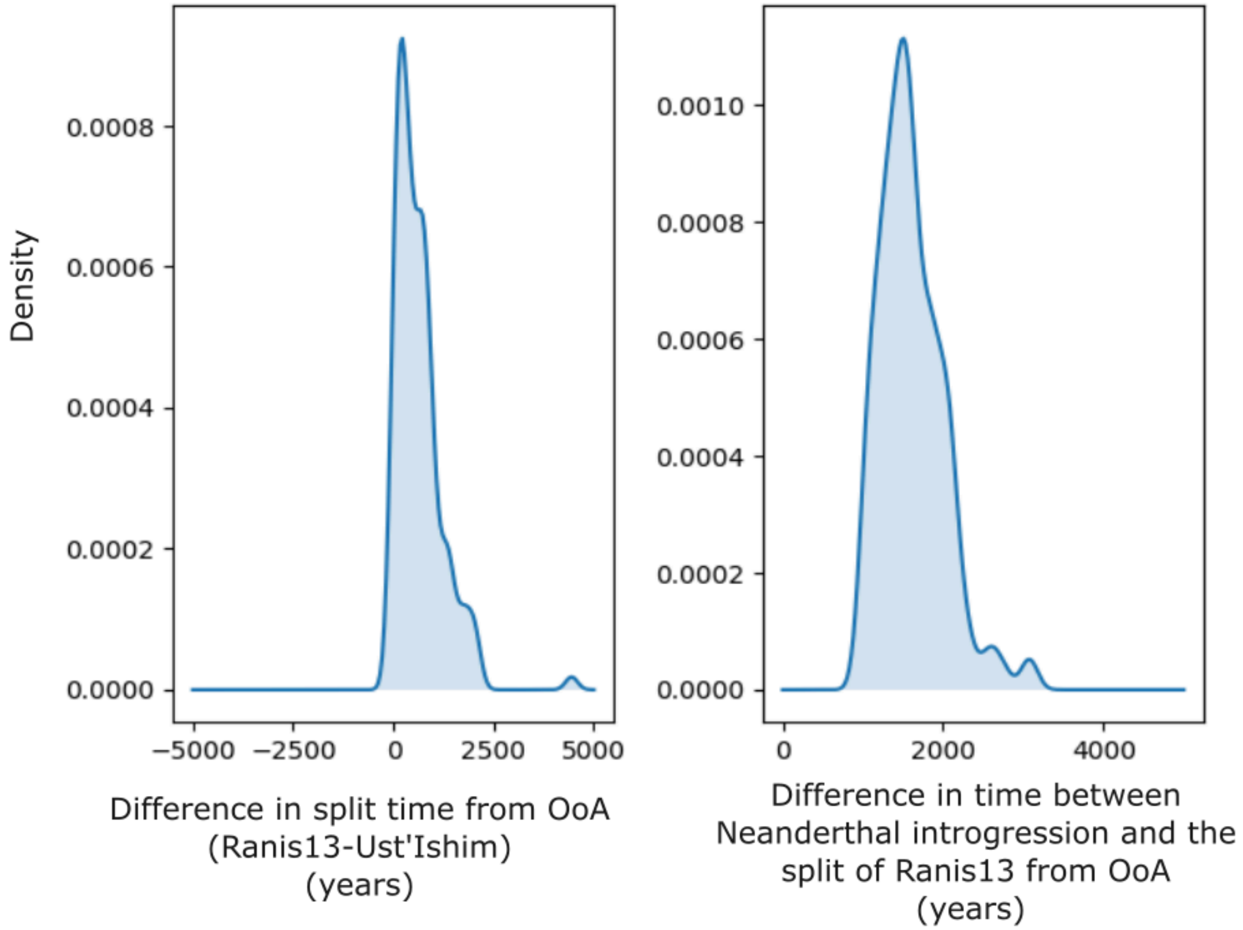

Supplementary Figure 12.2: Distribution of the differences in estimated split times for Ranis13 and Ust'-Ishim from other out-of-Africa populations (left) and of the time occurring between Neandertal introgression and the split of Ranis13/Zlatý kůň (right), both for the core model in which Yorubas (YRI), Han Chinese (CHB), Tuscani (TSI), Ust'-Ishim (UST), Ranis13 (RNI), Zlatý kůň (ZKU), Vindija33.19 (NEA), and an introgressing Neandertal population (IntrogNea) are included.

Supplementary Table 12.1: Parameter estimates for the core model without Papuans with 5%-confidence intervals estimated as the 2.5% and 97% quantiles of the estimates from bootstrapped data. Parameters are named using the following naming convention: *t* indicates dates from branch shortening estimates (when only one population is present) or split times between two populations (when two populations are present); *n* indicates effective population sizes, e.g. n_chb indicates the effective population size of CHB and n_RNI_HUM the effective population size of RNI and other out-of-Africa populations; *p* indicates the proportion of introgression for admixture events, also indicated as pulse and the name of the introgressing population, e.g. p_pulseNea is the introgression proportion of Neandertal admixture into modern humans, t_pulseNea is the time of introgression and, t_introgNea indicates the time of separation of the introgressing Neandertal populations from the sequenced Neandertals.

Parameter	Mean	2.50%	97.50%
n_chb	5075.85	4277.00	5471.70
n_yri	46419.13	40767.36	54146.51
n_chb_yri	14079.30	13691.50	14539.17
t_chb_yri	76855.16	71121.43	81942.79
n_tsi	8728.90	7399.58	9559.59
n_tsi_chb	1844.71	988.36	6040.54
t_tsi_chb	43338.91	36159.04	46366.73
n_nea	2228.06	2172.00	2286.10
n_nea_yri	14925.83	14749.98	15143.82
t_nea_yri	451876.84	450194.15	462288.41
t_nea	54863.03	48931.82	54988.27
p_pulseNea	0.02	0.02	0.03
t_introgNea	58941.18	53146.74	69469.11
t_pulseNea	49430.44	47898.74	52144.74
n_UST	901.51	291.20	2016.22
n_UST_ OoA	3026.30	946.38	17741.51
t_UST_ OoA	47263.47	45631.77	50159.12
n_RNI	183.56	50.04	716.30
n_RNI_OoA	2213.91	1780.47	3902.50
t_RNI_ OoA	47999.57	46927.14	51260.37
t_RNI_ZKU	45664.01	45186.15	47766.72
n_ZKU	225.56	61.27	1118.87
n_RNI_ZKU	514.27	500	1261.30

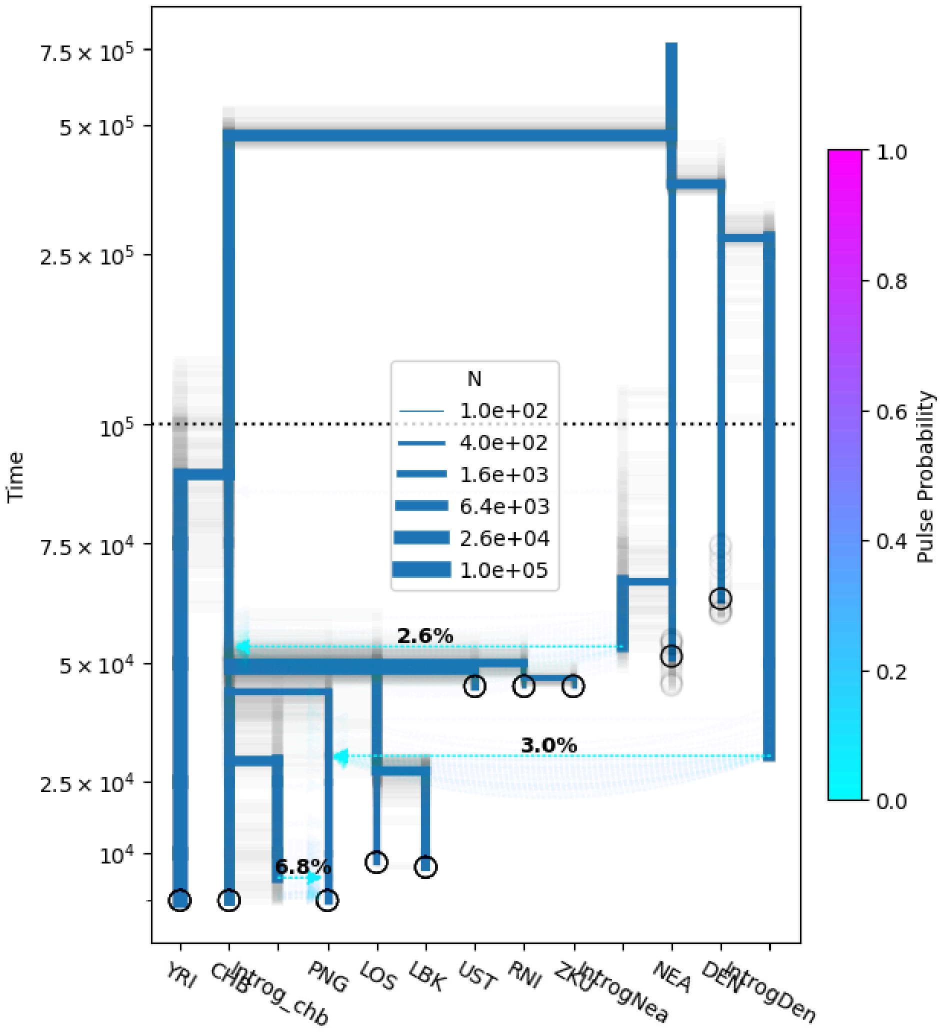

Supplementary Figure 12.3: Model estimates from momi2 for the simpler model (PNG model) in which Yorubas (YRI), Han Chinese (CHB), an introgressing Asian population into Papuans (Introg_chb), Papuans (PNG), Loschbour (LOS), Stuttgart LBK (LBK), Ust'-Ishim (UST), Ranis13 (RNI), Zlatý kůň (ZKU), Vindija33.19 (NEA), and an introgressing Neandertal population (IntrogNea) are included, as well as Denisova3 (DEN) and the introgressing Denisovan into Papuans. Shadows indicate estimates from individual bootstrap (resamplings of the site frequency spectrum) iterations. The thickness of blue lines indicates the estimated effective population sizes as described in the legend. The colour of the arrow indicates the proportion of admixture as indicated in the legend. The y-axis indicates time in years.

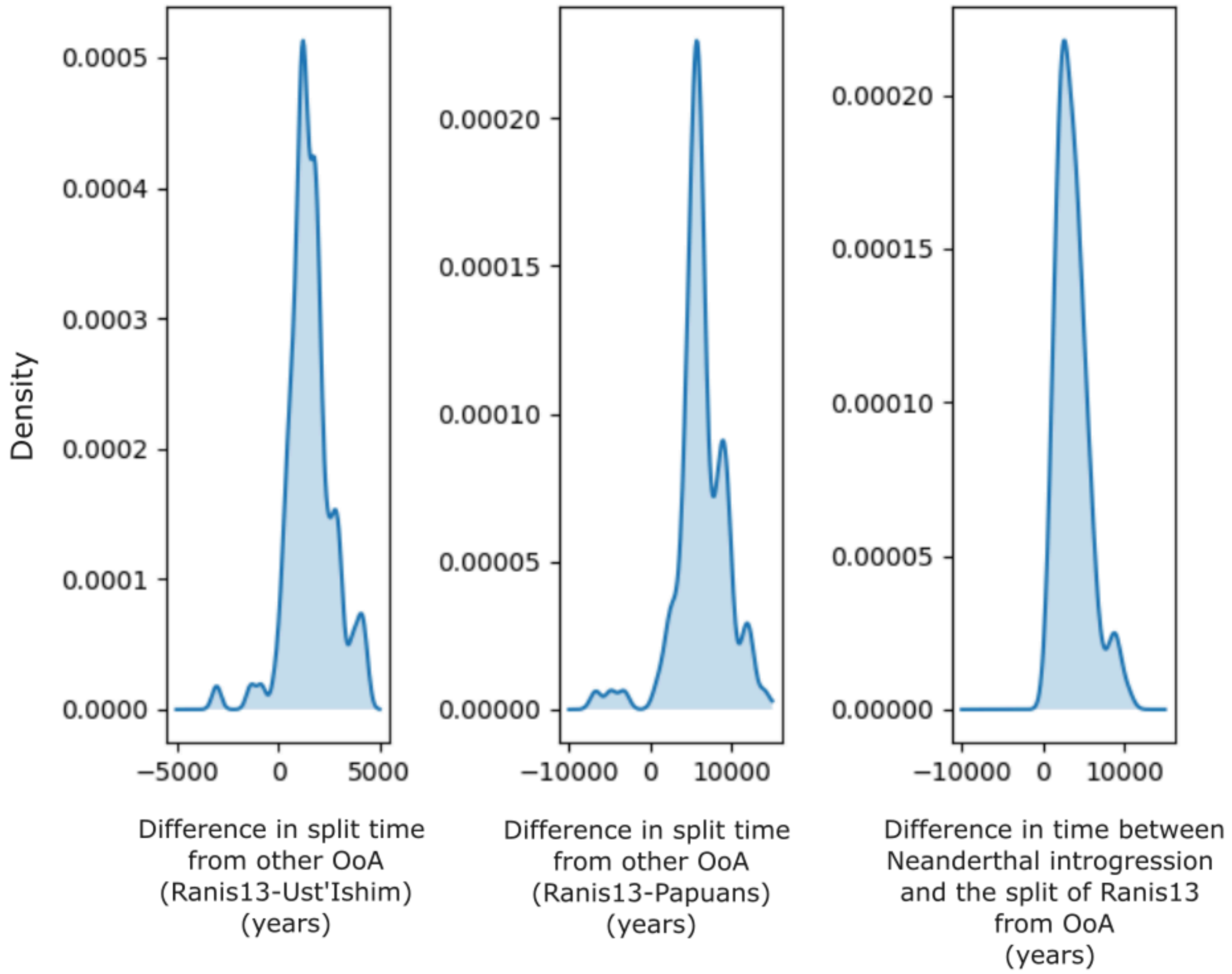

Supplementary Figure 12.4: Distribution of the differences in estimated split times of Ranis13 and Ust'-Ishim (left), or Ranis13 and Papuans (middle), from other out-of-Africa populations, for the PNG model. The time occurring between Neandertal introgression and the split of Ranis13/Zlatý kůň (right) is shown on the right for the same model.

Supplementary Table 12.2: Parameter estimates for the Papuan model, with 5%-confidence intervals estimated as the 2.5% and 97% quantiles of the estimates from bootstrapped data. Parameters are named using the same naming convention as described in Supplementary Table 12.1. In addition to Neandertal introgression into modern humans, Denisovan introgression into Papuans is estimated with p_pulseDen as the proportion of introgression, t_pulseDen as the time of introgression, and t_introgDen as the time of separation of the introgressing Denisovan from Denisova3. Note how – consistent with previous findings – the split time of the introgressing Denisovans is much deeper in time than that of the introgressing Neandertal and Vindija33.19. Introgression from CHB to Papuans was represented instead with the parameters p_pulsechbpng, t_introg_chbpng, and t_pulsechbpng, following the same naming convention as above.

Parameter	Mean	2.50%	97.50%
n_chb	6849.209	3282.528	7721.99
n_yri	47007.42	29602.53	75843.67
n_chb_yri	13800.48	11296.6	15156.81
t_chb_yri	89374.2	64685.86	131059.5
n_los	1084.651	718.104	4493.747
n_los_chb	3566.57	974.1418	50000
t_los_chb	49985.2	29536.04	56446.62
n_LBK	4075.993	254.6994	5213.532
n_LBK_ OoA	5480.051	500.579	8198.902
t_LBK_ OoA	27362.69	7385.07	29929.22
n_png	2195.042	1490.833	2708.287
n_png_chb	1303.965	1000	7339.349
t_png_chb	43774.15	38408.34	52131.58
n_nea	2068.948	1964.744	2396.864
n_nea_yri	15089.14	14271.31	16866.76
t_nea_yri	472615.2	451306.2	530347.9
t_nea	51275.21	45145.38	54900.27
n_den	2107.263	2001.431	2408.299
n_den_nea	5280.919	1049.032	8133.461
t_den_nea	364635.6	351137.4	431649.8
t_den	63349.85	60175.12	74769.45
p_pulseNea	0.025515	0.020919	0.036394
t_introgNea	67241.81	55010.56	114800.7
t_pulse	53334.63	49768.26	60169.24
p_pulseDen	0.029697	0.02	0.038231
t_introgDen	273796.3	132666.4	308450.9
t_pulseDen	30393.38	30051.23	38509.87
p_pulsechbpng	0.068076	0.006655	0.281119
t_introg_chb	29517.45	2049.648	44618.18
t_pulsechbpng	4828.456	206.6442	43344.06
n_UST	2036.314	449.2241	9771.13
n_UST_OoA	3074.303	912.7232	26371.05
t_UST_ OoA	48196.65	45844.83	53206.53
n_RNI	388.2701	100.4713	1271.356
n_RNI_ OoA	3281.482	1457.561	26062.43
t_RNI_ OoA	50002.56	47336.81	53975.69
t_RNI_ZKU	46583.96	45365.91	49565.23

n_ZKU	519.3964	127.8631	1599.821
n_RNI_ZKU	884.464	500	1975.511

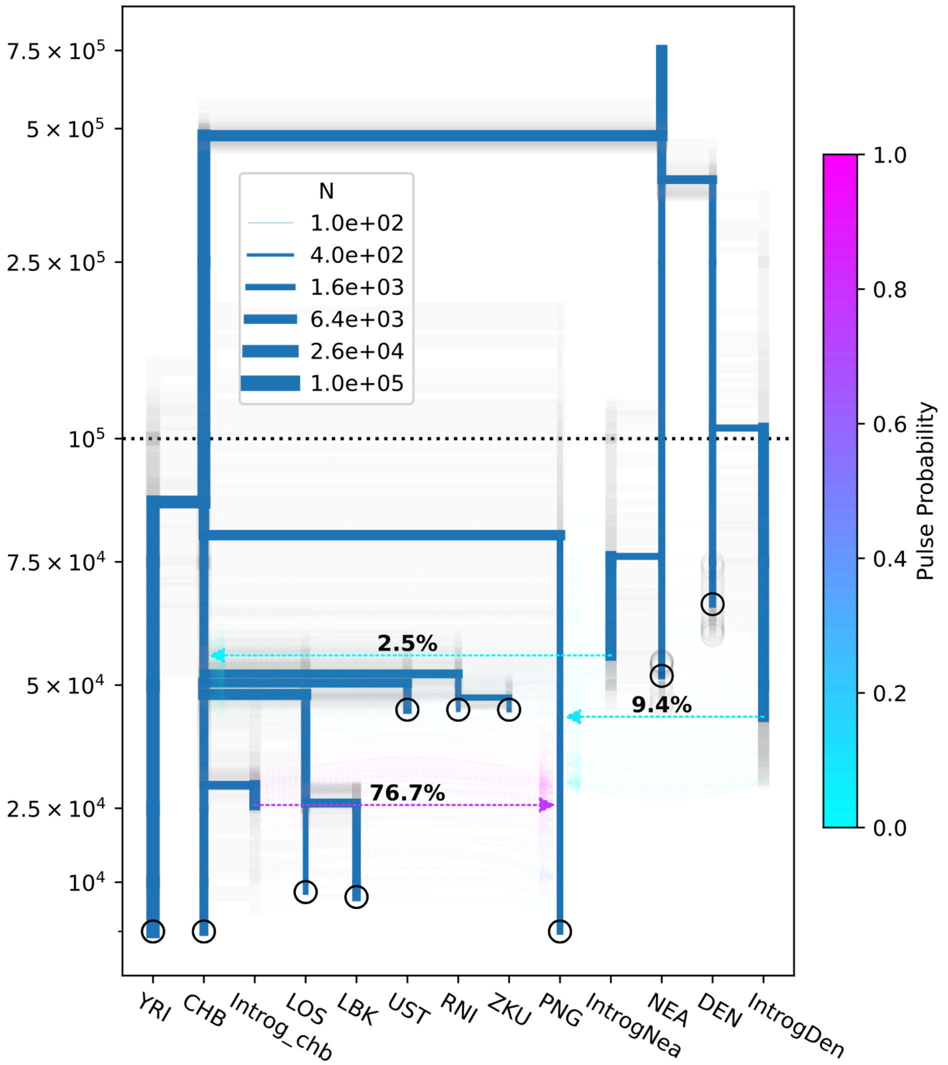

Supplementary Figure 12.5: Model estimates from momi2 for the model including the same populations as the PNG model, but including two out-of-Africa events – the earliest of which contributed to the ancestry of modern Papuans. Shadows indicate estimates from individual bootstrap (resamplings of the site frequency spectrum) iterations. The thickness of blue lines indicates the estimated effective population sizes as described in the legend. The colour of the arrow indicates the proportion of admixture as indicated in the legend. The y-axis indicates time in years.

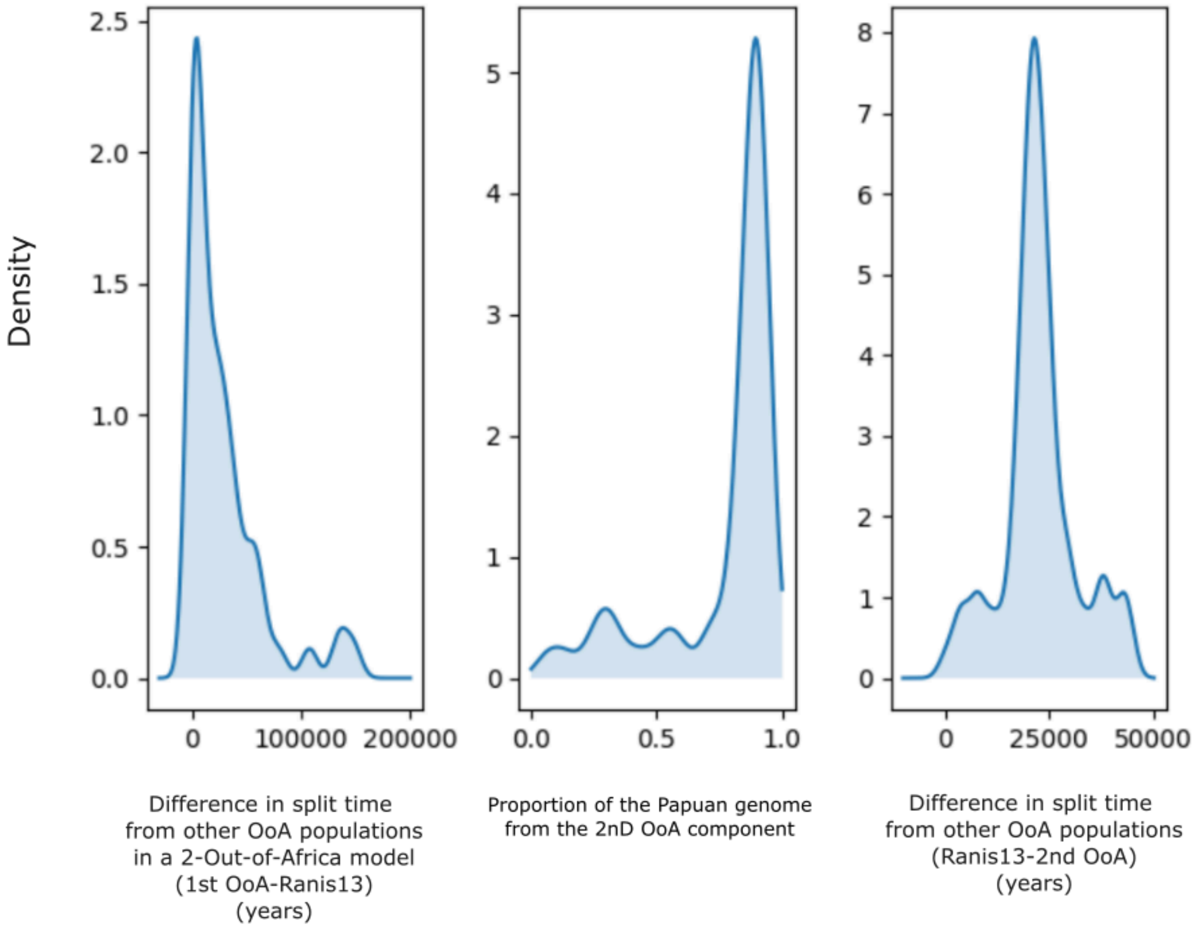

Supplementary Figure 12.6: Distribution of the differences in estimated split times from other out-of-Africa populations for the first early out-of-Africa population contributing to Papuan ancestry and Ranis13 (left), and of Ranis13 with the later out-of-Africa event contributing most of Papuan ancestry (right). In the middle, the proportion of the Papuan genome, which came from the later second out-of-Africa component, is shown.

Supplementary Table 12.3: Parameter estimates for the complex Papuan model, with 5%-confidence intervals estimated as the 2.5% and 97% quantiles of the estimates from bootstrapped data. Naming convention as in Supplementary Tables 1 and 2.

Parameter	Mean	2.50%	97.50%
n_chb	4573.17	3013.21	6940.08
n_yri	35043.81	11805.18	61412.22
n_chb_yri	30312.32	6766.93	200421.46
t_chb_yri	87442.1	54712	130261.09
n_los	998	534.31	1550.50
n_los_chb	12731.35	890.27	50000
t_los_chb	48904.2	24371.34	59043.02

n_LBK	4111.32	1179.69	6431.63
n_LBK_OoA	5109.32	943.86	8688.44
t_LBK_OoA	24011.02	16332.81	31724.13
n_png	1532.11	1035.7	26438.22
n_png_chb	10001.11	1142.8	29539.77
t_png_chb	80317.11	44961.1	198621.87
n_nea	2199.99	1433.05	2580.52
n_nea_yri	15299.00	13920.67	17214.32
t_nea_yri	481102.28	4678474.26	539491.76
t_nea	51986.12	45430.14	55151.33
n_den	2530.01	1805.13	2519.68
n_den_nea	4890.21	1452.01	7982.37
t_den_nea	379642.33	358597.17	452391.18
t_den	65412.2	60141.54	75147.56
n_UST	3516.18	243.71	12161.85
n_UST_OaA	5451.42	589.51	30000
t_UST_ OaA	50411.22	45041.85	58243.14
n_RNI	690.22	65.11	1962.72
n_RNI_ OaA	4890.75	590.97	27222.56
t_RNI_ OaA	52314.38	47234.61	59591.24
t_RNI_ZKU	48134.49	45244.48	51437.6
n_ZKU	921.23	121.24	2704.88
n_RNI_ZKU	1518.45	500.4	3548.21
p_pulseNea	0.02	0.01	0.04
t_introgNea	76166.82	51653.34	118046.37
t_pulseNea	56980.27	45431.05	81087.02
p_pulseDen	0.09	0.02	0.12
t_introgDen	104664.3	62513	296677.09
t_pulseDen	43540.39	30260.16	75395.18
p_pulse_chbpng	0.75	0.14	0.97
t_introg_chbpng	29673.33	11800.52	49543.08
t_pulse_chbpng	25577.01	8756.65	41709.14

Supplementary Information 13

Neandertal Ancestry in the High Coverage Genomes

Arev Pelin Sümer*, Leonardo Iasi, Stéphane Peyrégne, Kay Prüfer

Calling of the Neandertal segments

For the inference of Neandertal ancestry, we used admixfrog version 0.7.1[77] on the high coverage Zlatý kůň and Ranis13 genomes. We built our reference panels from allele information of the high coverage genomes of Denisova 3[21], Vindija 33.19[31], Denisova 5 (or the Altai Neandertal)[30], Chagyrskaya 8[34], the chimpanzee reference genome (*panTro6*), and all the female Mende, Mandenka, Yoruba, Esan, and Luhya individuals from the 1000 Genomes at the sites ascertained by the Archaic Admixture array (Fu et al., 2015, Panel 4)[8] and, for the X chromosome sites, from the Extended Archaic ascertainment[78]. We used the allele frequencies estimated from the Vindija 33.19, Altai Neandertal, and Chagyrskaya 8 genomes as the reference for Neandertals, Denisova 3 for Denisovans, and the 1000 Genomes Africans as representatives of African ancestry. The Chimpanzee reference genome was used to infer the ancestral state for each variant. If the ancestral state could not be determined, admixfrog uses a uniform prior for both alleles. The genetic distances were either assigned using the African American recombination map[79] or the deCODE map[80].

The exact command to obtain the input and run admixfrog for the analysis was:

```
admixfrog-bam --ref $ref --bamfile ranis13.bam --force-bam --deam-cutoff 3 --out
ranis13.in.xz --length-bin-size 15

admixfrog --infile  ranis13.in.xz --ref $ref -o ranis13 \
--states AFR NEA DEN  --cont-id AFR  --ll-tol 1e-2 --bin-size 5000 \
--est-F --est-tau --freq-F 3  --freq-contamination 3 --e0 1e-2  --est-error \
--ancestral PAN  --run-penalty 0.1 --max-iter  250 --n-post-replicates 200 \
--filter-pos 50 --filter-map 0.000  --init-guess AFR
```

We ran the program with the same parameters for the Zlatý kůň genome, as well as for the high coverage genome of the Ust'-Ishim[37] individual, for comparison. The default of admixfrog is to use the African American recombination map[79]. We repeated the analysis with the deCODE

recombination map[80] using the additional `--map-id deCODE` parameter, in order to check if the results agree between different recombination maps.

We did not detect any reliable Denisovan ancestry (i.e. all Denisovan segments were shorter than 0.025 cM) in the high coverage genomes of Ranis13, Zlatý kůň and Ust'-Ishim. Using the segments longer than 0.2 cM, we estimated the percentage of Neandertal ancestry in the autosomes of Ranis13 and Zlatý kůň to be 2.86% (2.80% - 2.94%) and 2.95% (2.89% - 3.02%), respectively. We estimated the Neandertal ancestry in the high coverage Ust'-Ishim genome to be 2.42% (2.36% - 2.47%) using the same approach. We identified in total 190.33 cM of Neandertal segments in the high coverage genome of Ranis13, with the longest fragment on chromosome 9 (chr9:694,566-696,681) being 10.58 cM long. For Zlatý kůň, we estimated the total length of Neandertal segments to be 196.03 cM, with the largest segment located on chromosome 16 (chr16:246,177-247,809) having the length of 8.16 cM.

In addition, we estimated the Neandertal ancestry on the X chromosomes of Ranis13 and Ust'-Ishim, both male, to be 0.08% (0.069% - 0.098%) and 0.026% (0.017% - 0.038%), respectively. We find that the Neandertal ancestry on the X chromosome of Ranis13 consists of two segments, one of 4.06 cM and 1.145 cM in length.

Testing the number of admixture pulses and dating Neandertal introgression

We used the Moorjani et al., 2016[81], dating method on the genotype calls of the high coverage Ranis13, Zlatý kůň, and Ust'-Ishim genomes to estimate the number of generations that passed since the Neandertal introgression. Following previous approaches[81,3], the method was run in bins of 0.001 cM and uses informative sites provided alongside the software package ("ascertainment0"). Genotype calls overlapping these informative sites were either downsampled to a random allele, resulting in states "2" or "0" for the input file, or taken completely into consideration, resulting in states that also include "1" for heterozygous calls. A model of one pulse or two pulses was fit to the range of 0.02-10 cM. Supplementary Table 13.1 summarizes the results. Neither Ranis13 nor Zlatý kůň yield plausible or significantly better fits to a two pulse model. This contrasts with Ust'-Ishim who shows evidence for two pulses, consistent with earlier estimates[81].

Supplementary Table 13.1: Estimated number of generations since Neandertal admixture for Zlatý kůň, Ranis13, and Ust'-Ishim using the Moorjani et al., 2016[81], method.

	Single Admixture		Two Pulses				LRT
Sample	Generations	SE	Generations1	SE	Generations2	SE	
Ranis13 (random allele)	57.0	0.71	649.6	172.80	53.4	1.01	8.91E-13
Zlatý kůň (random allele)	60.3	0.62	142.0	37.46	102.3	25.99	1
Ust'-Ishim (random allele)	86.8	1.36	22.8	2.71	203.3	10.03	4.97E-153
Ranis13 (full genotype)	60.0	0.65	1293.0	573.50	58.66	0.79	3.80E-05
Zlatý kůň (full genotype)	63.0	0.54	68.9	2706.00	72.72	909.20	1
Ust'-Ishim (full genotype)	91.7	1.19	21.8	2.23	206	7.81	2.44E-236

In addition to the Moorjani et al., 2016[81] dating method, we used another method where we calculated the likelihood of the observed Neandertal segment lengths assuming a single exponential distribution, or a mixture of two exponential distributions, which correspond to representing a single event of admixture or two events of admixture with different ages, respectively. As we used a length cutoff of 0.2 cM to avoid segments shared due to incomplete lineage sorting, we also used left-truncated distributions to model the decay of the Neandertal segment length. The maximum likelihood estimate of the rate of the single left-truncated exponential distribution is $\hat{\lambda} = \frac{N}{\sum_{i}^{N} Li - 0.002}$ and the corresponding likelihood is $\prod_{i}^{N} \hat{\lambda} e^{-\hat{\lambda}(Li - 0.002)}$, where N is the number of segments longer than 0.2 cM, and L_i is the length of segment i in Morgans. We computed the maximum likelihood estimates of the rates, λ_1 and λ_2, and the mixture proportion, p, using an Expectation-Maximization (EM) algorithm for the mixture of two left-truncated exponential distributions. In the expectation step, the probability that segment i was inherited from the first $(p1)$ or second $(p2)$ admixture event is:

$p1 = \frac{p\lambda_1 e^{-\lambda1 (Li - 0.002)}}{p\lambda1 e^{-\lambda1(Li-0.002)} + (1-p)\lambda2 e^{-\lambda2(Li-0.002)}}$ or

$p2 = \frac{(1-p)\lambda_2 e^{-\lambda2 (Li - 0.002)}}{p\lambda1 e^{-\lambda1(Li-0.002)} + (1-p)\lambda2 e^{-\lambda2(Li-0.002)}}$. In the maximization step, the parameters were updated as

$\hat{\lambda}1 = \frac{\sum_i^N p1}{\sum_i^N (Li-0.002)p1}$, $\hat{\lambda}2 = \frac{\sum_i^N p2}{\sum_i^N (Li-0.002)p2}$, and $p = \frac{\sum_i^N p1}{N}$,

and the likelihood is $\prod_i^N (p\hat{\lambda}_1 1 e^{-\hat{\lambda}1(Li-0.002)} + (1-p)\hat{\lambda}_2 e^{-\hat{\lambda}2(Li-0.002)})$.

The algorithm is assumed to have converged when the change in the log-likelihood was smaller than 10^{-8}, or after 1,000 iterations. In order to test if two exponentials or one fit the data better, we perform a likelihood ratio test with two degrees of freedom.

We investigated if the segment length distributions support a single or double introgression event in the high coverage Early Modern Human genomes of Ranis13, Zlatý kůň, and Ust'-Ishim by using two different segment calling methods, one using admixfrog as explained in the beginning of the chapter, and the other using the genotype calls, as explained in the beginning of this subsection. We also used two recombination maps, the African American map and the deCODE map. While calling the segments with admixfrog, we used two different run penalties, 0.1 and 0.25, which controls how likely nearby segments are joined or broken up. We used two different run parameters to measure the impact of this parameter, as the lengths of the detected segments will vary. While a run penalty of 0.1 expects the likelihood of the next bin to be more than 90% to continue an archaic segment, a run penalty of 0.25 expects this probability to be at least 75%. Thus, the inferred segments are broken more often when using a run penalty of 0.1, which results in shorter inferred segment lengths, and hence larger generation time estimates. Using this method, we did not find any evidence for more than one introgression event to Ranis13 and Zlatý kůň population, confirming the observation using the Moorjani et al., 2016[81], dating method (Supplementary Table 13.2, Supplementary Figures 13.1 and 13.2).

Supplementary Table 13.2: Log likelihood corresponding the fit to one or two exponentials using the segments detected by admixfrog or from the genotypes. Analysis repeated for two different recombination maps, two different admixfrog run penalties, and three high coverage Upper Palaeolithic human genomes.

Individual	Recombination map	Penalty	Log L. single	Lambda for single	Log L. two	Mixture of two	L. ratio stat	*p*-value	Method
Ranis13	AA	0.1	502.62	91.36	505.11	78.18493 (λ_1) 443.4739 (λ_2) 0.8249323 (proportion)	4.98	0.08	admixfrog
Ranis13	deCode	0.1	402.13	84.52	403.59	51.25661 (λ_1) 120.1244 (λ_2) 0.3135004 (proportion)	2.92	0.23	admixfrog
Ranis13	AA	0.25	441.14	78.85	444.68	67.05986 (λ_1) 531.9773 (λ_2) 0.8289457 (proportion)	7.07	0.03	admixfrog
Ranis13	deCode	0.25	393.45	80.78	393.79	79.79401 (λ_1) 8928.253 (λ_2) 0.9876501 (proportion)	0.69	0.71	admixfrog
Ranis13	AA	N/A	350.76	69.95	351.37	65.77559 (λ_1) 598.5838 (λ_2) 0.9329688 (proportion)	1.22	0.54	Segments from the genotypes
Ranis13	deCode	N/A	346.68	76.21	347.52	71.79602 (λ_1) 934.6139 (λ_2) 0.9373347 (proportion)	1.69	0.43	Segments from the genotypes
Zlatý kůň	AA	0.1	487.57	86.31	488.4	78.26881 (λ_1) 359.2733 (λ_2) 0.880922 (proportion)	1.67	0.43	admixfrog
Zlatý kůň	deCode	0.1	419.57	82.37	419.64	77.08604 (λ_1) 137.4297 (λ_2) 0.8540291 (proportion)	0.14	0.93	admixfrog
Zlatý kůň	AA	0.25	468.73	81.17	469.64	74.82501 (λ_1) 487.687 (λ_2) 0.9076411 (proportion)	1.83	0.40	admixfrog
Zlatý kůň	deCode	0.25	401.67	77.26	401.67	76.59185 (λ_1) 159.6985 (λ_2) 0.9833242 (proportion)	0.01	0.99	admixfrog
Zlatý kůň	AA	N/A	426.52	82.44	428.76	70.63016 (λ_1) 419.1386 (λ_2) 0.8276636 (proportion)	4.48	0.11	Segments from the genotypes

Zlatý kůň	deCode	N/A	394.79	84.18	396.55	77.72315 (λ_1) 1125.6 (λ_2) 0.9175922 (proportion)	3.52	0.17	Segments from the genotypes
Ust'-Ishim	AA	0.1	525.47	115.97	525.47	115.9259 (λ_1) 151.6909 (λ_2) 0.998355 (proportion)	0.001	0.99	admixfrog
Ust'-Ishim	deCode	0.1	486.79	118.34	487.16	116.5174 (λ_1) 7458.33 (λ_2) 0.9843733 (proportion)	0.74	0.69	admixfrog
Ust'-Ishim	AA	0.25	517.81	109.80	518.27	108.3129 (λ_1) 10590.58 (λ_2) 0.9863585 (proportion)	0.93	0.63	admixfrog
Ust'-Ishim	deCode	0.25	459.78	107.58	459.79	106.9647 (λ_1) 1615.552 (λ_2) 0.9938966 (proportion)	0.03	0.99	admixfrog
Ust'-Ishim	AA	N/A	436.97	103.69	437.72	96.15613 (λ_1) 673.7535 (λ_2) 0.9152473 (proportion)	1.51	0.47	Segments from the genotypes
Ust'-Ishim	deCode	N/A	404.98	104.42	405.79	99.17874 (λ_1) 1533.794 (λ_2) 0.9463107 (proportion)	1.63	0.44	Segments from the genotypes

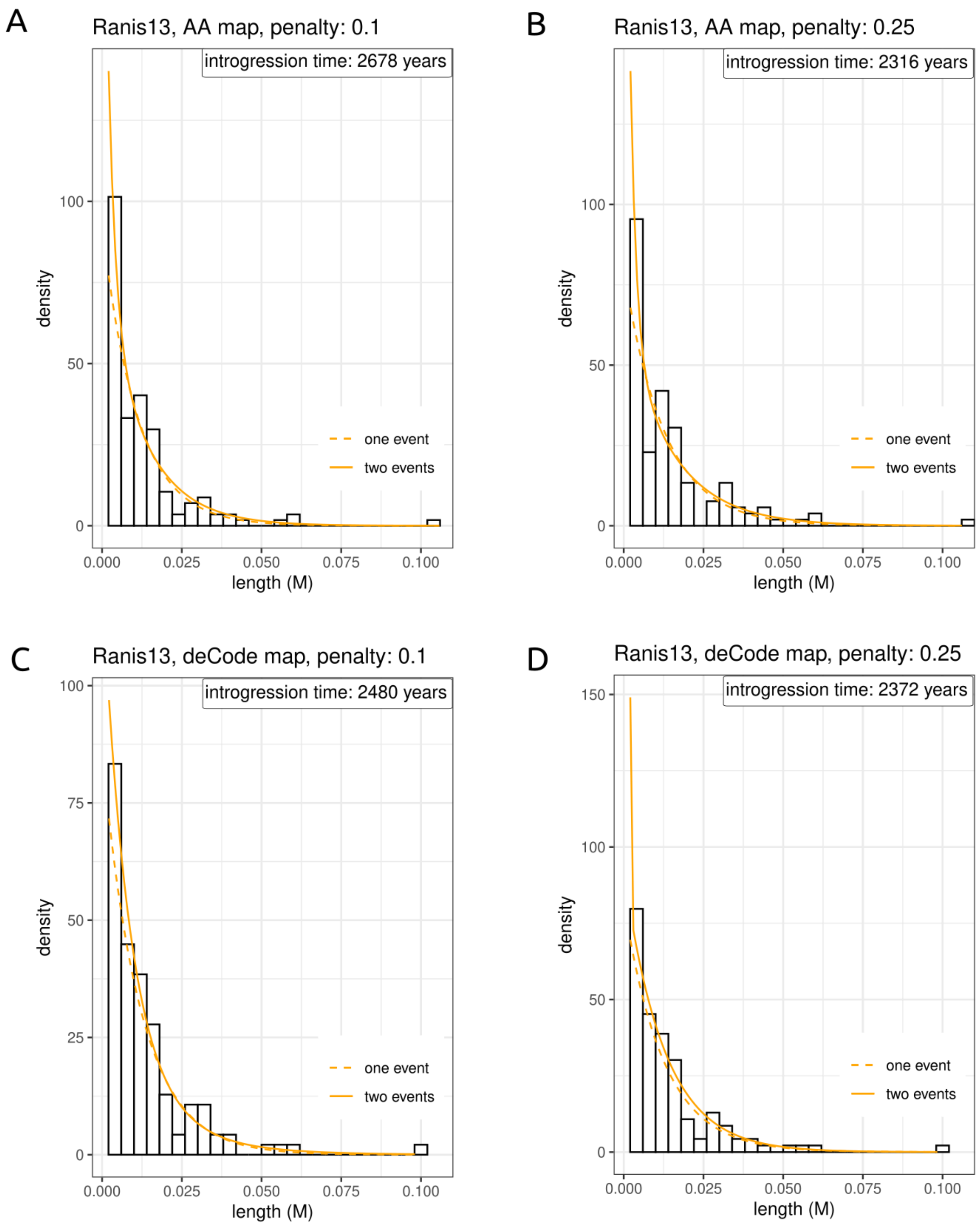

Supplementary Figure 13.1: Binned lengths of Neandertal segments in the high coverage Ranis13 genome, along with the fitted distributions corresponding to one or two introgression events. Panels **A** and **B** present the results when using the African American recombination map[79] with two different run penalties as explained in the text, while **C** and **D** use the deCode recombination map[80].

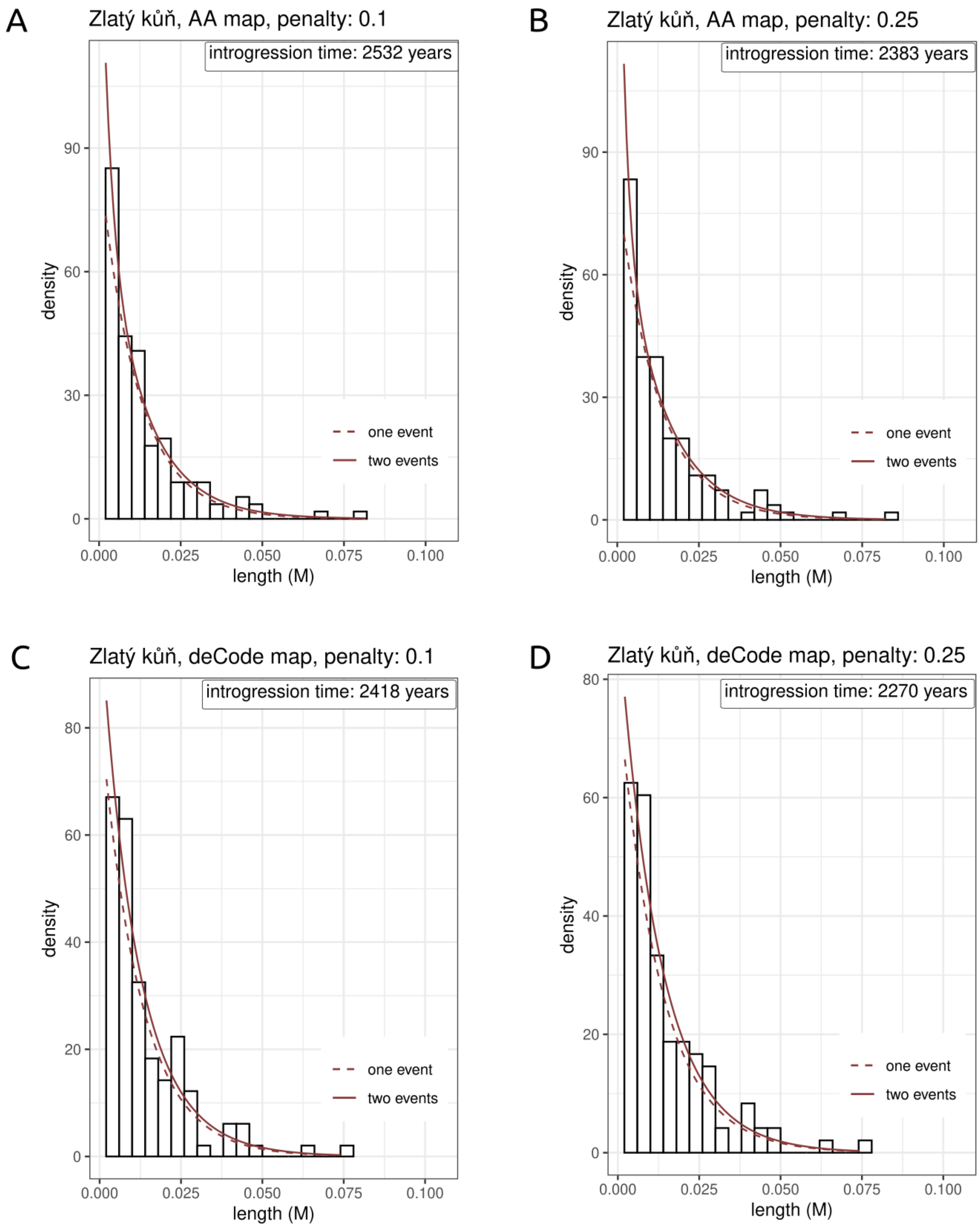

Supplementary Figure 13.2: Binned lengths of Neandertal segments in the high coverage Zlatý kůň genome, along with the two exponential distributions corresponding to one or two introgression events. Panels **A** and **B** present the results when using the African American recombination map[79] with two different run penalties as explained in the text, while **C** and **D** use the deCode recombination map[80]. Text

on the right top corner indicates the best fit for the introgression time before the life of the individual, assuming a generation time of 29 years.

After we had two independent methods pointing to a single introgression event into Ranis13 and Zlatý kůň, we also used the exponential decay method[3], to estimate the generations since a hypothetical single pulse admixture event. We used the 100 longest segments detected by admixfrog, using two different run penalties again, 0.1 and 0.25 (autosomes only).

Supplementary Table 13.3: Estimated number of generations since Neandertal admixture for Zlatý kůň and Ranis13 using the 100 largest segments as described in Prüfer et al., 2021[3]. Segments used here were detected by admixfrog[77] using two different penalty parameters, 0.1 and 0.25.

**Estimate**	**Zlatý kůň**		**Ranis13**	
	**Mean**	**CI (95%)**	**Mean**	**CI (95%)**
AA recombination map, p = 0.1	80.2	(64.5 - 95.5)	82.2	(66.0 - 97.8)
Decode map recombination map, p = 0.1	80.8	(64.9 - 96.2)	81.9	(65.9 - 97.6)
AA recombination map, p = 0.25	74.3	(59.6 - 88.3)	70.6	(56.7 - 83.9)
Decode map recombination map, p = 0.25	76.8	(61.7 - 91.4)	79.3	(63.7 - 94.3)

In all four comparisons, we find that generation time estimates for Zlatý kůň and Ranis13 overlap and are between 55 and 96 generations, including their 95% CI. Interestingly, we observe that in all but one of the four cases, Zlatý kůň is one to three generations older than Ranis13, even though the logarithmic length of the segments indicate Ranis13 has longer segments and should therefore be older (Supplementary Figure 13.3). It should be noted that we observed Ranis13 to be older than Zlatý kůň using the Moorjani et al., 2016[81], dating method, but the opposite pattern with the fit to one or two exponentials method, thus these two individuals were most likely contemporaneous.

We date the timing of this introgression event using the direct radiocarbon date on Ranis13 and assuming a generation time of 29 years, following previous studies[31,34], to 45,024-49,422 years BP. A recent study by Wang et al., 2023[82] reported generation time estimates of humans in time bins across the past 250,000 years. The average generation time estimate they report for humans who lived between ~43,000 and ~50,000 years fluctuate between 29.15 and 29.70 years (in their supplementary data table named gentime_estimatesAll.txt, between bin_age 1484.35 and 1712.83). Recalculating the timing of the introgression event with these generation times yields a

date from 45,032 to 49,491 years BP, very close to our estimates using a generation time of 29 years.

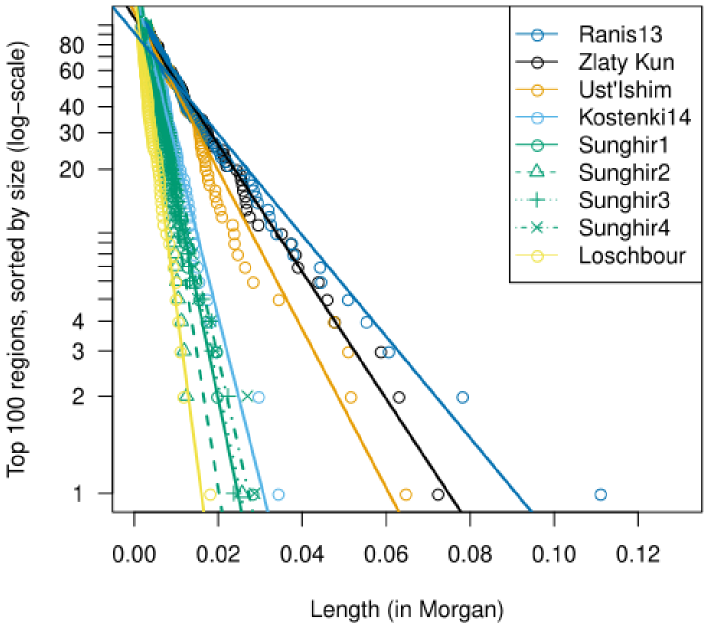

Supplementary Figure 13.3: Logarithmic length of the 100 longest Neandertal fragments in the Ranis13 and Zlatý kůň genomes, and linear fit to these lengths. Other Upper Palaeolithic and Mesolithic Eurasian hunter-gatherers are plotted for comparison.

Extended pulse model

In addition to a simple pulse, we also tried to fit an extended pulse as described in Iasi et al., 2021[83]. This method assumes that migration rates follow a gamma distribution over a duration described by the shape parameter k with the mean time of the admixture at t_m instead of a single generation long admixture event. The simple pulse model is an edge case of the extended pulse model, which is recovered when k approaches infinity. If k goes to 2, it approaches a model of constant migration. We fitted the inferred segments of Zlatý kůň and Ranis13 to the probability density distribution of an extended pulse using the *optim* function in R. We only considered segments longer than 0.2 cM. We used empirical significance cutoffs from Kozubowski et al., 2008[84], to distinguish between a simple and extended pulse, since standard likelihood theory cannot be applied. A log-likelihood difference equal or bigger than 2.5 is significant for $\alpha = 0.05$. Since these two individuals are from the same population and close in time shown by their IBD sharing (Supplementary Figure 7.5D), we also jointly modeled the mean admixture time and k parameter using the data from both individuals at once (Supplementary Table 13.4). Supplementary Figure 13.4 shows the migration rate density over time predicted by the extended pulse model, where 0 is the sample time.

Supplementary Table 13.4: Estimated mean admixture time t_m (in generations before the live time of the individual), duration parameter k, duration in which 95.5 % of gene flow occurs (in generations before the live time of the individual) and LRT between the simple pulse and extended pulse model for Zlatý kůň, Ranis13 and the joint fit using both individuals together using segments inferred with admixfrog and a penalty of 0.25.

Individual	map	t_m (95.5 % CI)	k	gene flow start and end (95.5 % percentile)	LRT
Ranis13	AA Map	77 (67 - 87)	2	9 - 214	9.6
Ranis13	deCODE	86 (73 - 99)	2	10 - 240	11.2
Zlatý kůň	AA Map	77 (67 - 87)	2.4	12 - 201	5.1
Zlatý kůň	deCODE	74 (63 - 86)	4.1	21 - 161	1.8
Ranis13 & Zlatý kůň	AA Map	79 (72 - 86)	2.4	13 - 205	12.8
Ranis13 & Zlatý kůň	deCODE	81 (73 - 90)	2.9	16 - 200	9.3

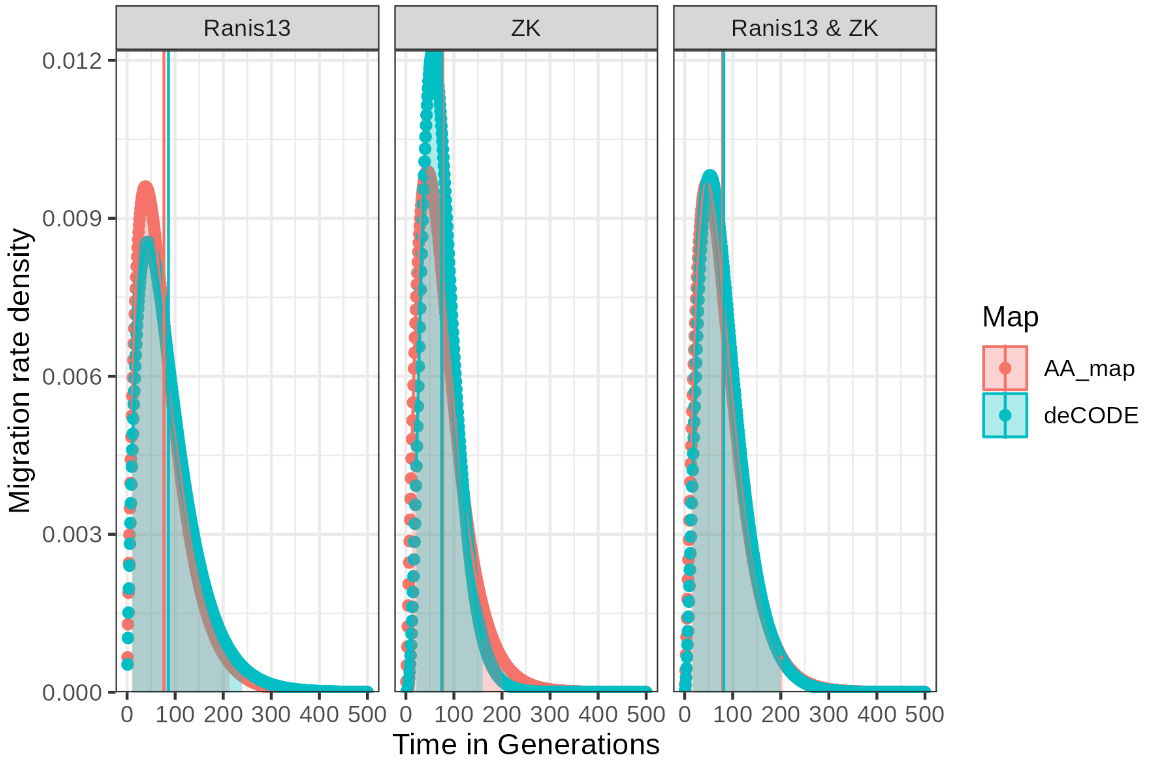

Supplementary Figure 13.4: Migration rate density over time for the extended pulse model using the highest likelihood parameters for Ranis13, Zlatý kůň and the joint fit to Ranis13 and Zlatý kůň using two different recombination maps. Vertical lines indicate mean admixture time (t_m). Shaded area the 95.5 percentile interval of the distribution. Zero generation corresponds to the sampling time of the individual.

Supplementary Information 14

Neandertal Ancestry in the Low Coverage Genomes

Arev Pelin Sümer*, Vanessa Villalba-Mouco

We used the Archaic Admixture array designed by and published in Fu et al., 2015, specifically Panel 4[8], as it allows for a better detection of introgressed Neandertal segments in the genomes of the Ranis individuals compared to the 1240k array. For this, we enriched 17 libraries for ~1.7 million SNPs at which one or more Neandertals and one Denisovan carry a different allele from the one carried by the Yoruba individuals from 1000 Genomes, and filtered the sequences as explained in Supplementary Information 3. We merged the libraries from the same specimen together, in the cases where we captured multiple libraries from a single specimen. We estimated the contamination in each library, specimen, and eventually individual, using AuthentiCT[22], and report these along with the average depth in all sequences and the sequences with terminal C to T substitutions (Table S1.4, Supplementary Table 14.1). The average depths for Ranis10, Ranis84, and Ranis86 were too low, and present-day human contamination was relatively high, however we still ran admixfrog on these data in the hope of detecting some Neandertal segments, albeit unsuccessfully (Supplementary Figure 14.1). The average depth for Ranis6 was also low, however admixfrog was able to detect Neandertal segments. However, for this individual, results from the analysis using the detected segments should be considered with caution, as data quality was on the edge of what is required for these analyses.

Supplementary Table 14.1: Average depth and present-day contamination estimates for individuals for which we obtained low coverage genomes using the Archaic Admixture capture array. CI (confidence intervals) for the contamination estimates are calculated by adding and subtracting two standard errors.

Individual ID	Specimen ID(s)	Coverage (all)	Coverage (deam)	Contamination % (AuthentiCT)
Ranis4	RNI004	1.08	0.26	8.4 (7.6 - 9.1)
Ranis6	RNI006	0.18	0.053	19.2 (18.6 - 19.8)
Ranis10	RNI010	0.14	0.04	13.3 (12.8-13.9)
Ranis12	RNI080, RNI081, RNI085	2.56	0.7	10.9 (10.1 - 11.7)
Ranis84	RNI084	0.04	0.01	23.4 (22.3 - 24.5)
Ranis86	RNI086	0.03	0.005	31.2 (30.0 - 32.5)
Ranis87	RNI087	1.38	0.48	0.7 (0.5 - 1.5)

Detecting archaic segments from the Archaic Admixture capture data

To detect the segments introgressed from Archaic Humans (Neandertals and Denisovans), we used admixfrog version 0.7.1[77] on the low coverage genomes obtained by Archaic Admixture capture. The command used for obtaining the input file from a bam file, and the one to run admixfrog on this, is reported with the exact parameters. We used the same reference panel used for the admixfrog analysis of the high coverage genomes (see Supplementary Information 13), even though the Archaic Admixture array does not target any positions on the sex chromosomes.

```
admixfrog-bam --ref ${ref} --bamfile ${RNIX.filtered}.bam --force-bam --deam-cutoff 3
--out {RNIX}.in.xz --length-bin-size 15
```

```
admixfrog --infile {RNIX}.in.xz --ref $ref -o RNIX \
--states AFR NEA DEN  --cont-id AFR  --ll-tol 1e-2 --bin-size 5000 \
--est-F --est-tau --freq-F 3  --freq-contamination 3 --e0 1e-2  --est-error \
--ancestral PAN  --run-penalty 0.1 --max-iter  250 --n-post-replicates 200 \
--filter-pos 50 --filter-map 0.000  --init-guess AFR
```

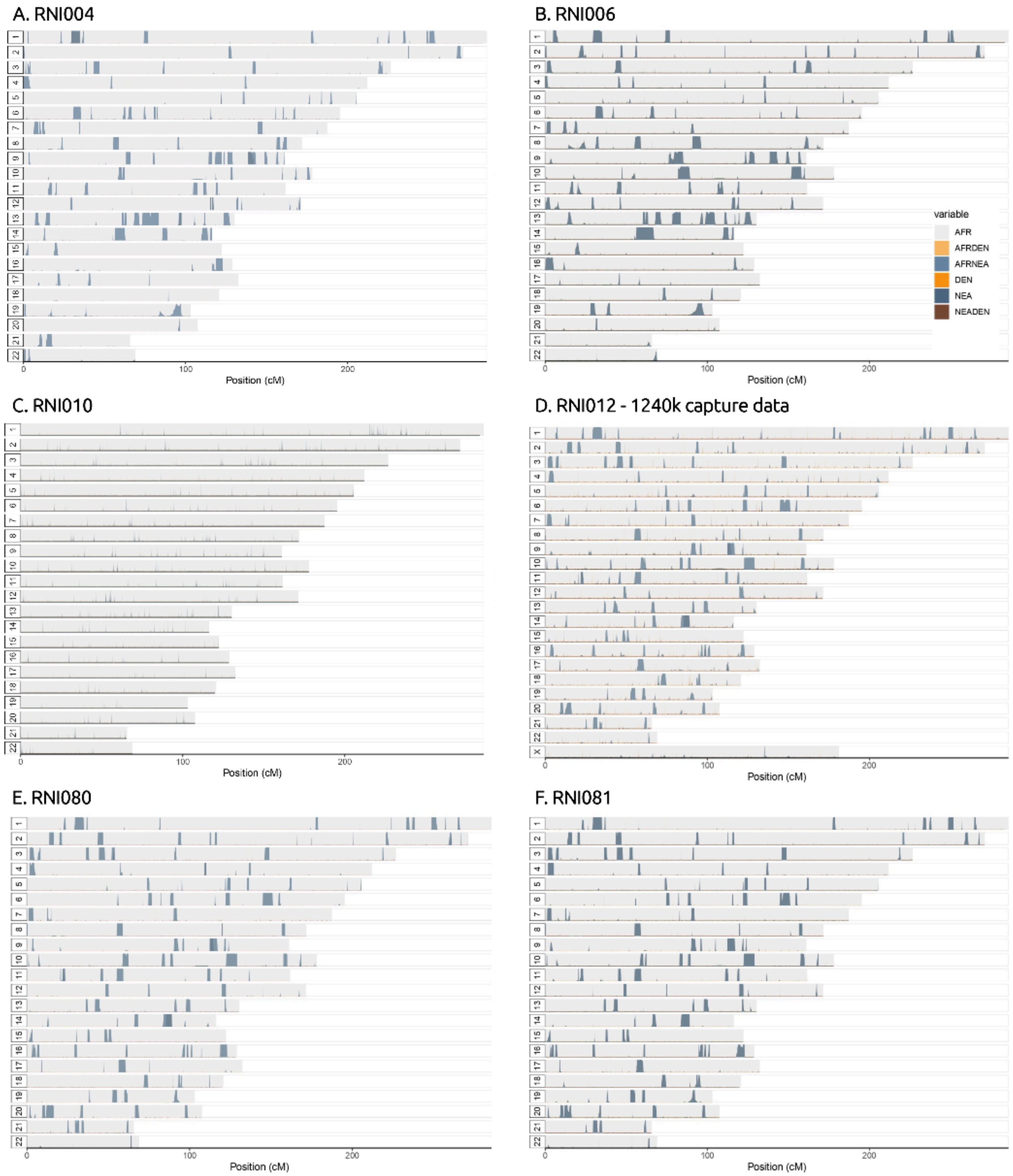
A. RNI004
B. RNI006
C. RNI010
D. RNI012 - 1240k capture data
E. RNI080
F. RNI081
Position (cM)
variable
AFR
AFRDEN
AFRNEA
DEN
NEA
NEADEN

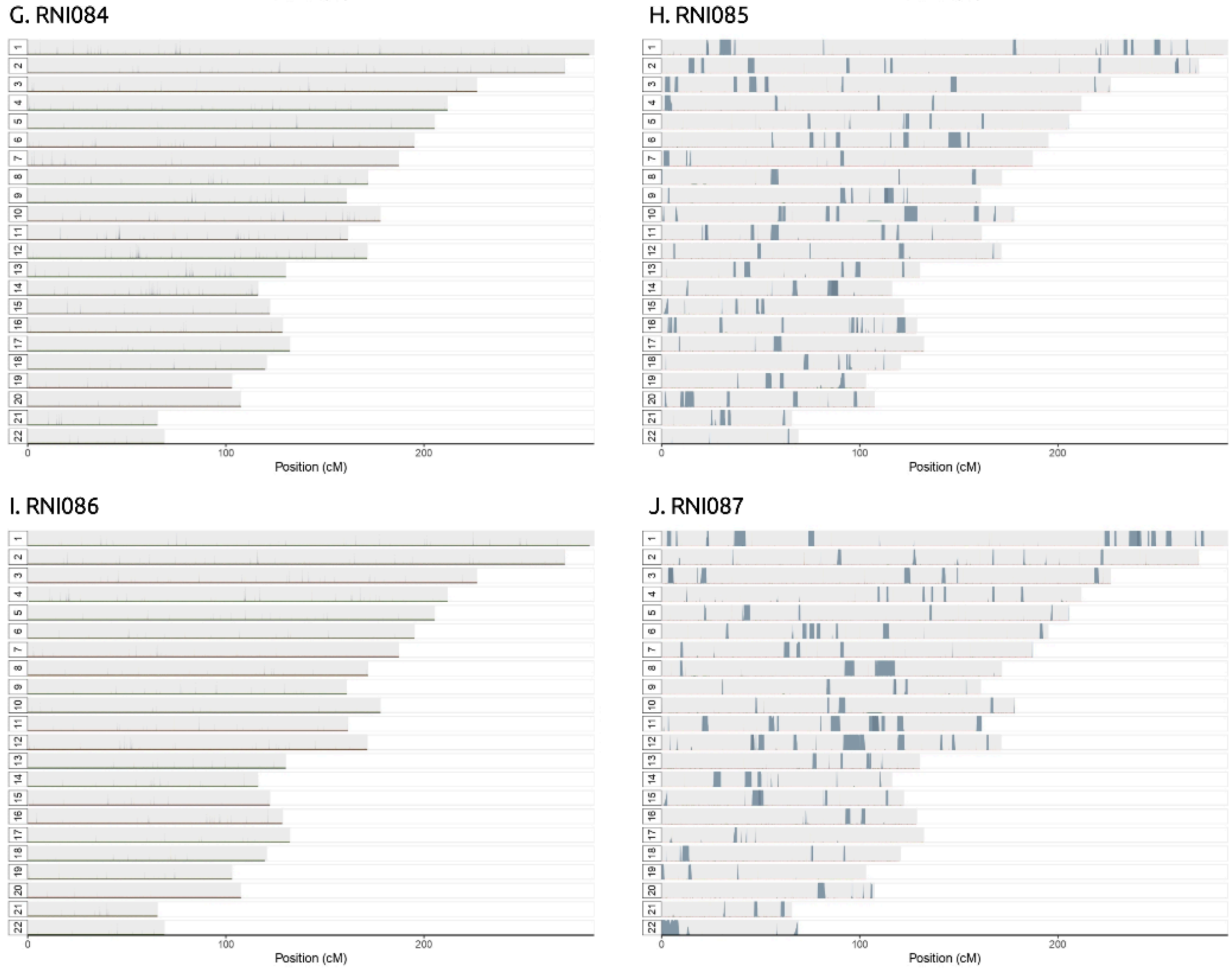

Supplementary Figure 14.1: Archaic segments detected in the low-coverage-captured genomes from Ranis. The data for all except RNI012 were obtained by Archaic Admixture capture. Segments detected in the 1240k data from RNI012 are plotted for comparison with RNI080, RNI081, and RNI085. There was not enough data for RNI010, RNI084, and RNI086 to detect any introgressed fragments.

We did not detect any Denisovan segments in the low coverage Ranis genomes, in the same way as for the high coverage ones. The Neandertal segments we detected were long and comparable in length with those of Ranis13. We noticed the segments detected in the genomes of RNI012, RNI080, RNI081, and RNI085 were the same and overlapped perfectly, as would be expected for specimens coming from the same individual, supporting the results of our kinship analysis (as reported in Supplementary Information 6).

All Neandertal segments in Ranis6 were called as homozygous, which is a clear indication of unreliable segment calling due to low data quality. As discussed earlier in the chapter, the coverage of Ranis6 genome is on the edge of what is required for calling segments with

admixfrog. Furthermore, we know that Ranis4 is the mother of Ranis6, and in her genome we observe only four homozygous segments with a total length of 10.46 cM, while 104 heterozygous segments with lengths summing to 136.79 cM. Meanwhile for Ranis6, we observe 83 homozygous segments with a total length of 138.765 cM, and no heterozygous segments, due to low data quality.

Testing the number of admixture pulses and dating Neandertal introgression

We used the method explained in the previous section to test if the length distribution of the Neandertal segments in the low coverage genomes from the Ranis individuals are best explained by a single introgression event or a mixture of two exponential distributions, corresponding to two admixture events. For this, we used the segments detected by admixfrog using a run penalty of 0.1 and the African American recombination map. We used a minimum length cutoff of 0.2 cM, as we did for the high coverage genomes. We did not find any evidence for more than one introgression event for any of the four low coverage genomes (Supplementary Table 14.2 and Supplementary Figure 14.2).

Supplementary Table 14.2: Log likelihood corresponding the fit to one or two exponentials using the segments detected by admixfrog.

Individual	Log L. single	λ for single	Log L. two	Mix of two	L. ratio stat	*p*-value
Ranis4	328.28	77.46	328.91	53.37 (λ_1)105.73 (λ_2) 0.372 (proportion)	1.27	0.53
Ranis6	237.58	61.93	237.67	61.63 (λ_1) 19779.45 (λ_2) 0.995 (proportion)	0.18	0.91
Ranis12	428.61	81.59	428.69	80.34 (λ_1) 996.30 (λ_2) 0.983 (proportion)	0.16	0.93
Ranis87	364.29	68.29	364.37	68 (λ_1) 14501.94 (λ_2) 0.995 (proportion)	0.16	0.92

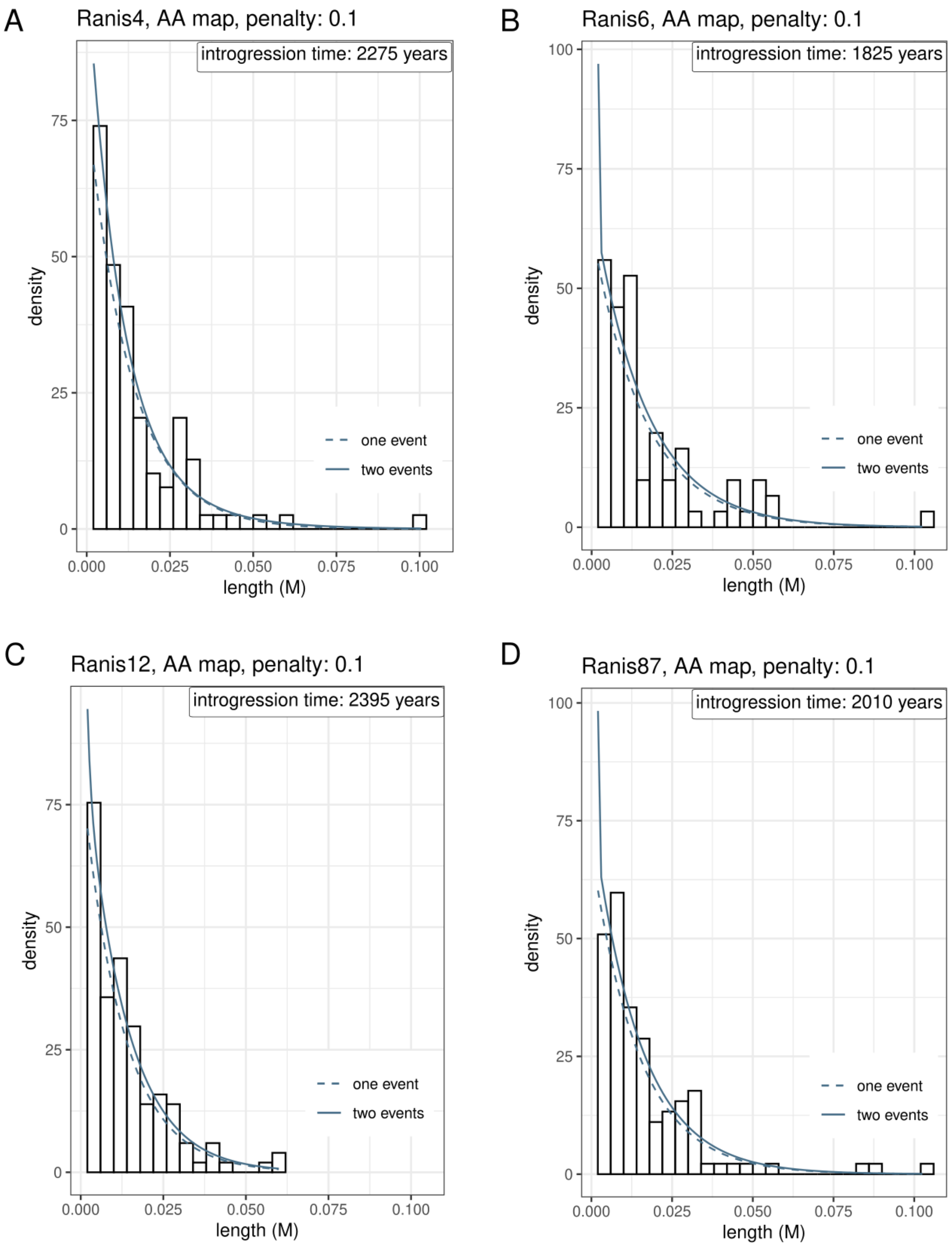

Supplementary Figure 14.2: Binned lengths of Neandertal segments in the low coverage genomes of the Ranis individuals, along with the two exponential distributions corresponding to one or two introgression events. Introgression time estimate reported for the best fitting λ, calculated assuming a generation time of 29 years.

Estimates of generations since the introgression event

Using the length distribution of the Neandertal segments detected by admixfrog, we estimated for each individual how many generations passed since the Neandertal introgression event. We excluded Ranis10, Ranis84, and Ranis86 from this analysis as the genome coverage for these individuals was too low to detect any Neandertal introgressed segments. As we did this analysis on an individual basis, we merged the data from the Archaic Admixture captures of specimens RNI080, RNI081, and RNI085 and refer to this individual as Ranis12, after the name of the specimen with the lowest number. Using the 100 longest segments and following the method from Prüfer et al., 2021[3], we estimated the number of generations, and the chi-square 95% confidence interval around the point estimates. This was done using 0.1 run penalty and the African American recombination map[79] and is comparable to the generation time estimates of 80.2 (64.5 - 95.5) and 82.2 (66.0 - 97.8) for Zlatý kůň and Ranis13, respectively, with overlapping confidence intervals, indicating that these individuals were most likely contemporaneous (Supplementary Table 14.3).

Supplementary Table 14.3: Number of generations between the life of the individual and the Neandertal introgression event. Point estimates are reported along with the 95% confidence interval obtained from the chi-square distribution, using the 100 longest segments detected by admixfrog.

Individual ID	Generation estimate	Minimum generation estimate	Maximum generation estimate
Ranis4	75.2	60.9	90.2
Ranis6	74.5	59.8	88.6
Ranis12	78.7	63.2	93.6
Ranis87	70.4	56.5	83.7

Estimates of Neandertal ancestry proportion using the segments

In addition to detecting the Neandertal fragments and using their length distributions to estimate the number of generations since the introgression event, we also included those used to estimate the proportion of Neandertal ancestry in the low coverage data obtained from Archaic Admixture captures. For this, we used the new algorithm in admixfrog introduced in Salem et al., Nature (in review), and filtered out the segments shorter than 0.2 cM (approximately 200 kb) to remove segments caused by incomplete lineage sorting (ILS), and restrict the analysis only to the confidently called segments with Neandertal ancestry. As all the Neandertal segments in Ranis6

were called as homozygous due to low data quality, Neandertal ancestry percentage is double of what is observed in the other Ranis individuals.

Supplementary Table 14.4: Neandertal ancestry estimates using segments longer than 0.2 cM, as explained in Salem et al., Nature (in review). Estimates for Ranis6 are in red as they are likely biased and should be considered with caution.

Individual ID	Neandertal ancestry (%)	Minimum Neandertal ancestry (%)	Maximum Neandertal ancestry (%)
Ranis4	2.85	2.74	3.0
Ranis6	6.28	5.96	6.76
Ranis12	3.00	2.91	3.06
Ranis87	3.20	3.12	3.31

Calculation of Neandertal ancestry using *f4*-ratio on 1240k capture data

We estimated the proportion of Neandertal ancestry (α) in the autosomes of the Ranis individuals using the "direct *f4*-ratio test" [30,31,85], using ADMIXTOOLS (https://github.com/DReichLab) of the form $\alpha = \frac{f_4(Altai, Chimpanzee, X, Dinka)}{f_4(Altai, Chimpanzee, Vindija, Dinka)}$. We also replicated the results using Chagyrskaya 8, a Neandertal around 65,000 years old, instead of the Altai Neandertal, who is around 120,000 years old, which resulted in similar estimates. The results obtained using the 1240k capture data point to similar estimates calculated using the Archaic Admixture panel (Supplementary Table 14.4). We confirm the higher amount of Neandertal ancestry reported by the authors in Bacho Kiro IUP[41] and Oase 1[8] when compared to the Ranis individuals. We also confirm a highly variable estimate in the low coverage genomes in line with the results reported by Posth et al., 2023[25] (Supplementary Figure 14.3, Supplementary Table 14.5).

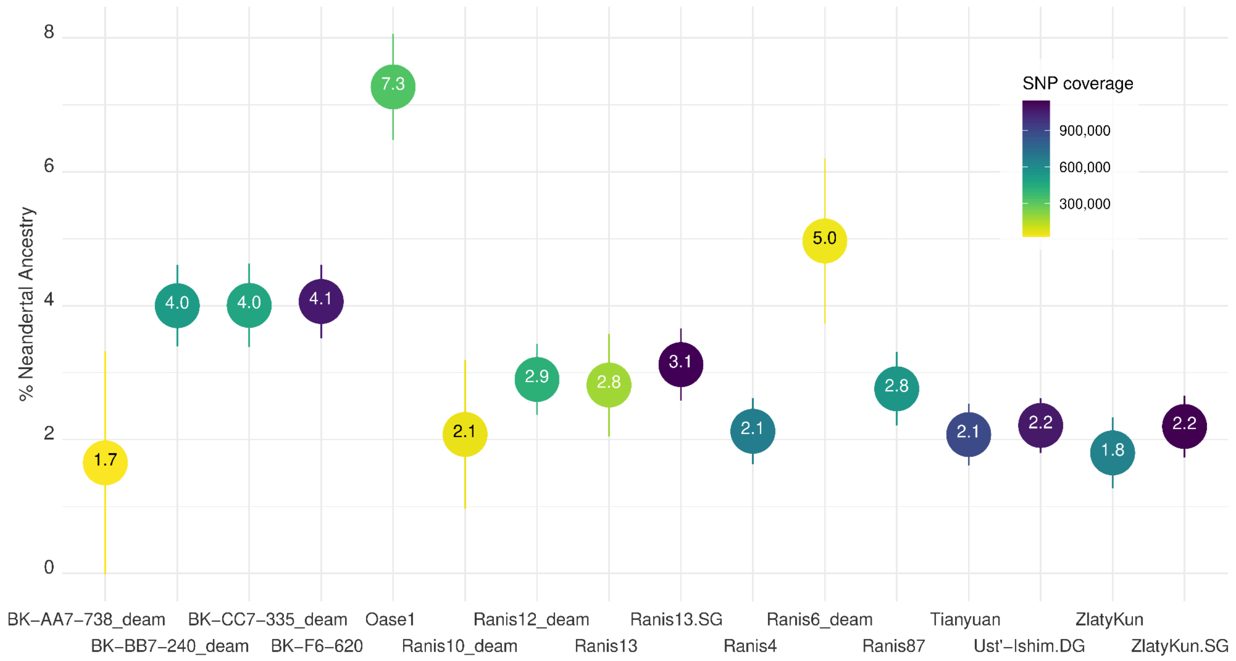

Supplementary Figure 14.3: Neandertal ancestry percentage estimated using the *f4*-ratio test and SNP coverage per individual. Error bars correspond to three standard errors.

Supplementary Table 14.5: Neandertal ancestry percentage values estimated using *f4*-ratio using Altai and Chagyrskaya 8 Neandertals.

Individual	Value of the *f4*-ratio test (Altai)	Three standard errors (Altai)	Zscore (Altai)	Value of the f4-ratio test (Chagyrskaya8)	Three standard errors (Chagyrskaya8)	Zscore (Chagyrskaya8)	SNP coverage of targeted individual
Ranis4	2.1232	14.811	4.3	2.3145	14.667	4.735	660264
Ranis13	2.8146	22.998	3.672	2.8767	2.229	3.872	181469
Ranis13.SG	3.1191	16.152	5.793	3.2066	16.071	5.986	1140912
Ranis87	2.7581	16.476	5.022	2.8812	16.062	5.381	544676
Ranis6_deam	4.9671	36.981	4.029	5.3804	36.603	4.41	46823
Ranis10_deam	2.0767	33.384	1.866	2.3259	32.055	2.177	61987
Ranis12_deam	2.8974	15.924	5.459	2.8984	15.492	5.613	420816
Zlatykun.SG	2.195	13.815	4.766	2.2475	13.464	5.008	1142037
Zlatý kůň	1.8008	159	3.398	1.9766	15.567	3.809	653677
BachoKiro_F6-620	4.0623	16.545	7.366	4.1457	1.608	7.735	1059660
BachoKiro_AA7-738_deam	1.6521	49.998	991	1.095	48.795	673	28538
BachoKiro_BB7-240_deam	3.9994	18.294	6.559	4.0877	17.889	6.855	519492
BachoKiro_CC7-335_deam	4.1942	18.702	6.424	4.0444	18.276	6.638	467678
Ust'-Ishim.DG	2.2104	12.291	5.395	2.2503	12.366	5.459	1062044
Oase1	7.2692	23.835	9.15	7.2955	23.496	9.315	325309
Tianyuan	2.0738	13.779	4.515	2.222	13.428	4.965	885079

Supplementary Information 15

Neandertal Ancestry Correlation

Leonardo Iasi

Calling of Neandertal segments

We inferred segments of Neandertal ancestry as described in Supplementary Information 13. We supplemented our data set of the two high coverage genomes of Zlatý kůň and Ranis13 with the dataset used in Iasi et al., 2024[78], which contains 274 individuals from the SGDP data set[39] as representatives of present-day humans, and 57 ancient modern humans which span from around 45,000 years BP to 2,000 years BP. The ancestry in all samples was called using admixfrog[77] with the archaic admixture + X ascertainment described in Iasi et al., 2024[78]. We used the African-American map to assign genetic length[79]. For the SGDP data, admixfrog was run using the genotype mode. We obtained the posterior probability for each bin and each ancestry state (i.e. Neandertal-Neandertal, Denisovan-Denisovan, African-African and Neandertal-African, Neandertal-Denisovan, Denisovan-African). We used a bin size of 0.005 cM and a penalty for classification of archaic ancestry of 0.25.

Calculating the correlation of Neandertal segments

For this analysis we only consider segments and bins on segments for autosomal segments with lengths of at least 0.2 cM in ancient individuals and 0.05 cM in present-day individuals. For each bin, we inferred the posterior probability for each ancestry state (i.e. Neandertal, Denisovan or African) by summing up the probability of having heterozygous and homozygous ancestry related to that state. We only consider Neandertal ancestry by putting all bins on a called Neandertal segment to 1 and the rest to 0. For present-day individuals, we followed the same procedure as Massilani et al., 2020[86]. We only considered the union of Neandertal segments of an SGDP population by merging all individuals of a SGDP population into one genome. We calculated the correlation of Neandertal bins for each pair of genomes using the *cor* function in R.

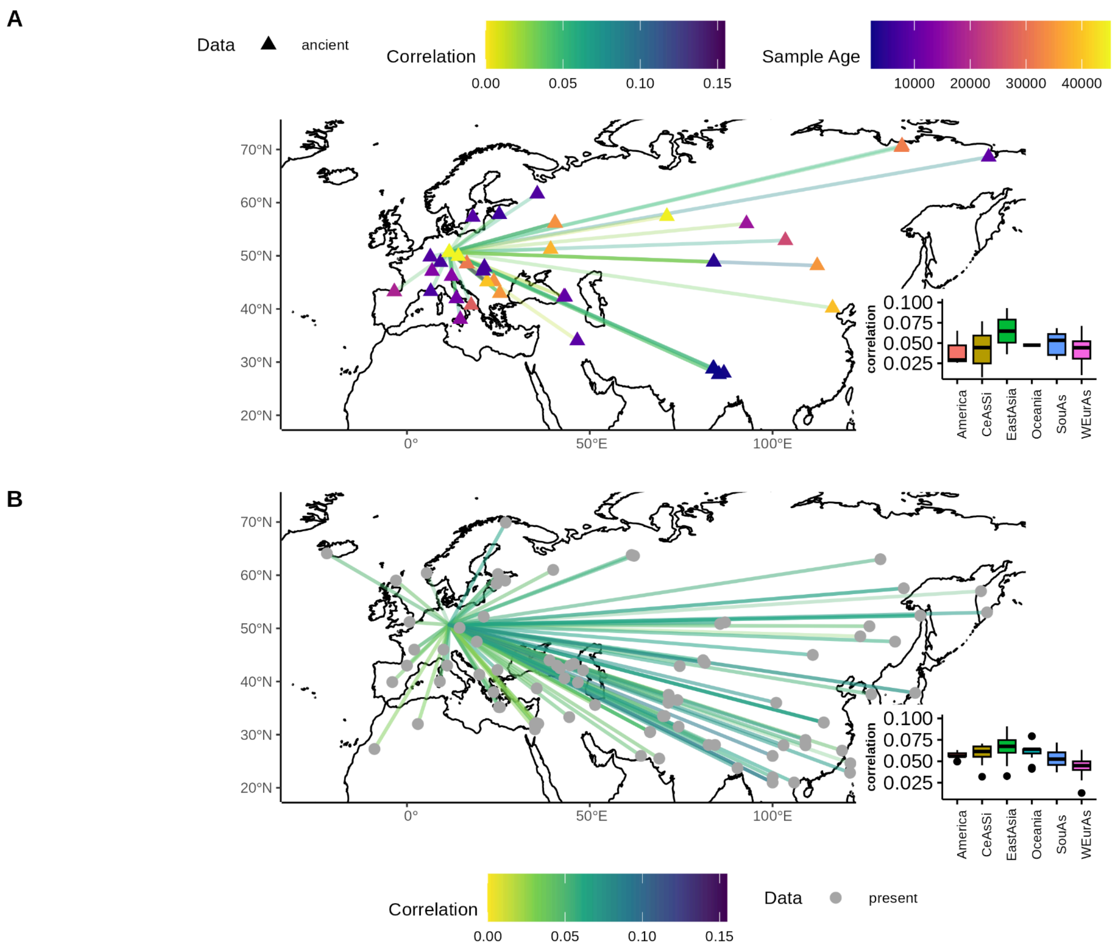

Supplementary Figure 15.1: Correlation of Neandertal segments in Ranis13 with **A.** ancient individuals, and **B.** present-day individuals. Coloured triangles give the sample location of ancient individuals with the colour indicating the sample age. Line colour indicates the strength of correlation. The boxplots summarize the correlation for all individuals in a SGDP superpopulation (CeAsSi = CentralAsiaSiberia, WEurAs = WestEurasia).

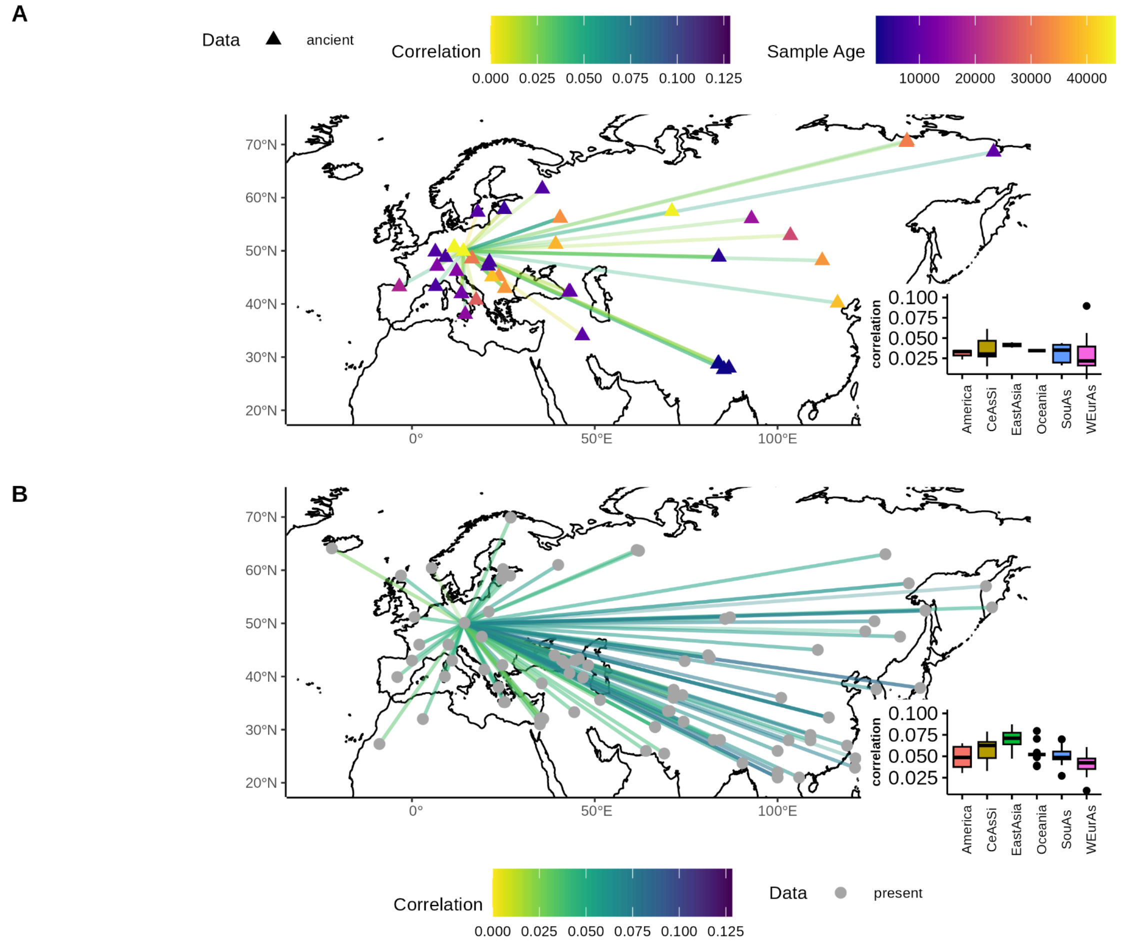

Supplementary Figure 15.2: Correlation of Neandertal segments in Zlatý kůň with **A.** ancient individuals, and **B.** present-day individuals. Coloured triangles give the sample location of ancient individuals with the colour indicating the sample age. Line colour indicates the strength of correlation. The boxplots summarize the correlation for all individuals in a SGDP superpopulation (CeAsSi = CentralAsiaSiberia, WEurAs = WestEurasia).

We investigated the correlation to Ust'-Ishim[37], Oase 1[8], and Bacho Kiro F6-620, CC7-335, and BB7-240[41], which are close in time to Zlatý kůň and Ranis13, in more detail. Since these individuals have very long Neandertal segments, we establish significance thresholds by randomly shuffling the location of Neandertal segments throughout the genome. Furthermore, we jackknife the segments by always leaving one out and calculating the correlation. We report the mean and standard deviation from 100 replicates of the shuffling and Jackknife runs (Supplementary Figure 15.3).

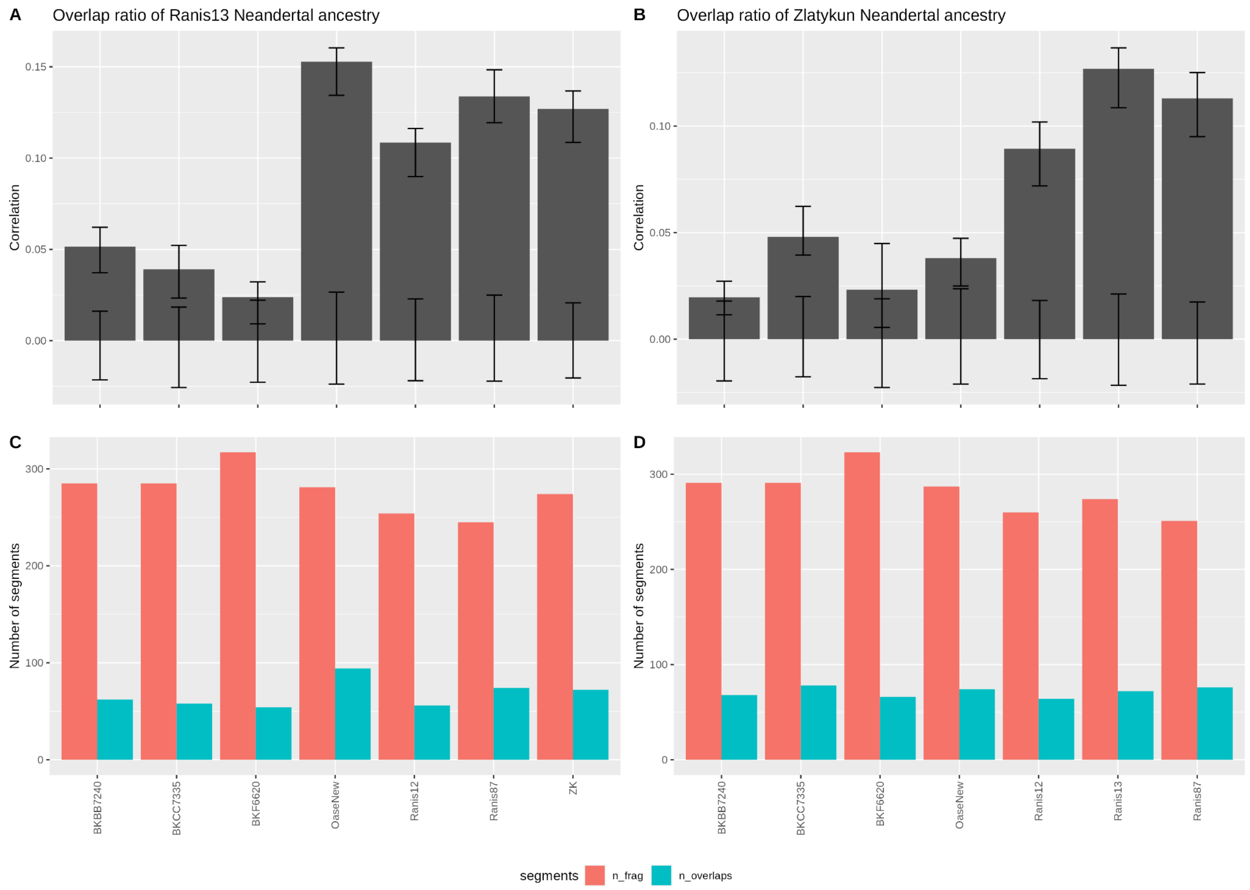

Supplementary Figure 15.3: Correlation analysis between early out-of-Africa individuals. **A.** Correlation of Ranis13 segments to all others. **B.** Zlatý kůň to all others. **C.-D.** Total number of segments and number of overlapping segments. The lower error bars indicate the mean and standard deviation from random correlation by shuffling the segments. The upper error bars indicate standard deviation from jackknifing the segments.

Supplementary Information 16

Sharing of Breakpoints in Neandertal Segments

Kay Prüfer

We locate breakpoints of Neandertal ancestry in the high coverage genomes of Ranis13, Zlatý kůň, Ust'-Ishim, and other ancient Modern Humans and compare these breakpoints to those in over 2000 present-day Modern Human genomes. Breakpoints in present-day humans are shared with Ranis13 and Zlatý kůň, indicating that Ranis13 and Zlatý kůň share an initial Neandertal admixture event with present-day out-of-Africans.

Segments of Neandertal ancestry

We use a set of sites that are informative for Neandertal ancestry to infer Neandertal segments in Modern Human genomes. At these sites, the 1000 Genomes Phase III African genomes (YRI, MSL, LWK, GWD, ESN)[87] carry an allele of at least 99.9% allele frequency that differs from the allele observed in the high coverage Vindija Neandertal genome[31]. Heterozygous calls in the Vindija Neandertal genome are excluded. The Modern Human reference *hg19* allele is considered as African fixed allele when a site is not included as variable in the 1000 Genomes dataset.

We further filter for a derived allele in the Vindija Neandertal and an ancestral allele for the (near-)fixed African allele. To define ancestral and derived states, we consult the base in the chimpanzee, bonobo, gorilla, orangutan, and rhesus macaque genomes[88–92] according to their *hg19* whole genome alignments[93,94]. All five outgroup bases are required to be identical to define the ancestral state. The alignments are freely available[95].

The set of informative sites is joined with a curated, combined set of genotype calls[96] of data from the HGDP[97] and 1000 Genomes[98] sequencing projects. After applying the HGDP accessibility mask (https://ngs.sanger.ac.uk/production/hgdp/hgdp_wgs.20190516/accessibility-mask/, lifted over to *hg19*) and removing sites where individuals had an unresolved genotype, the combined data consists of around 300,000 autosomal sites with diploid genotype calls from 2,591 non-African

individuals that are mono- or bi-allelelic and match the African and/or Neandertal states. Sites without a call were again considered fixed for the *hg19* allele.

To call Neandertal segments, the diploid calls along the genome were converted into a sequence of “0” and “1”, where “1” represents a genotype where either one or both alleles matched the Vindija Neandertal state and “0” a genotype where both alleles match the African state. A Hidden Markov Model approach was applied using the R[99] package HMM[100]. Separately for each individual, the posterior probabilities for hidden states “Modern-Human-Ancestry” and “Neandertal-Ancestry” were inferred from the sequence of “0” and “1” after applying the Baum-Welch algorithm to estimate transition and emission probabilities.

The distribution of posterior probabilities is strongly bimodal, with the majority of sites showing a probability close to 0 for Neandertal ancestry and a smaller fraction of sites showing a high probability of Neandertal ancestry (Supplementary Figure 16.1). To test whether the distribution of these probabilities reflects previously inferred rates of Neandertal ancestries in world-wide populations, we calculated the fraction of sites with a posterior probability greater than 0.75 for all individuals (Supplementary Figure 16.2). We also include in this test African individuals from the HGDP dataset. Note that the genomes of these African individuals were not part of the set that was used to define Neandertal informative sites. At most 0.3% of sites in these African genomes were inferred to originate from Neandertals. Out-of-African genomes typically show 1-2.5% Neandertal ancestry. The misclassification of Denisovan ancestry leads to a higher proportion of inferred sites of Neandertal ancestry in Oceanians.

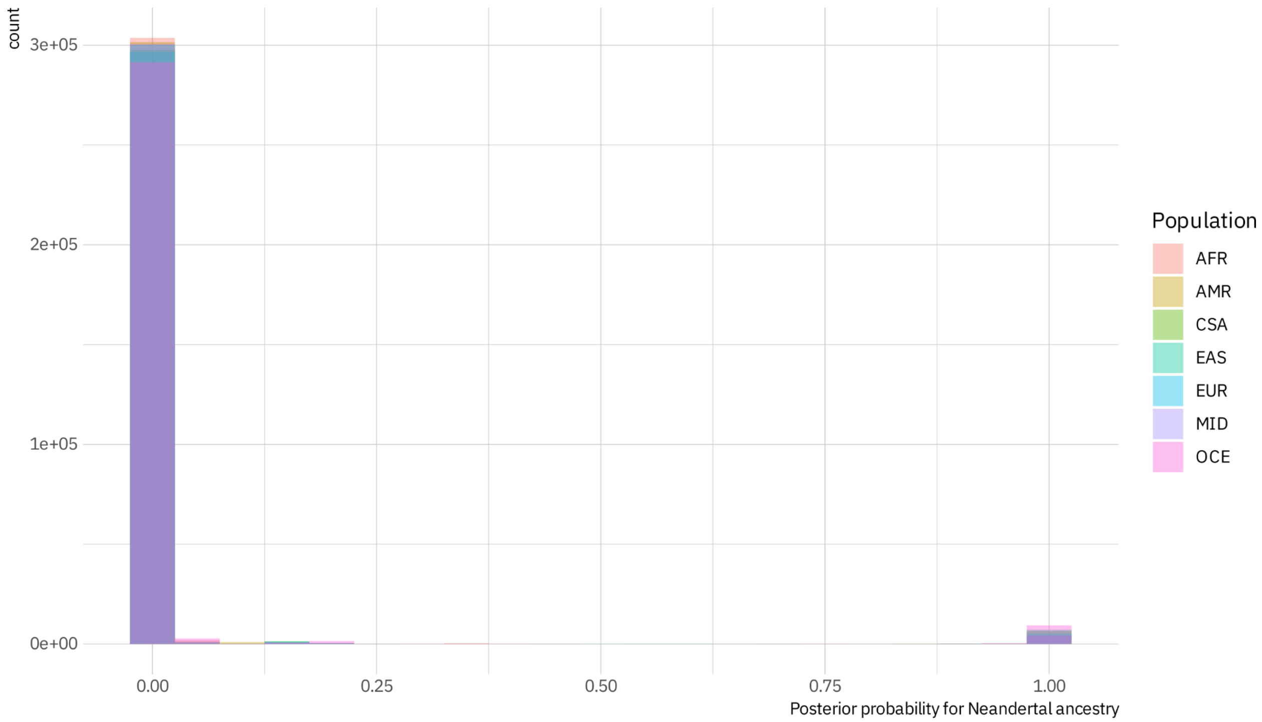

Supplementary Figure 16.1: Histogram of posterior probabilities for one random individual from each population represented in the HGDP/1000 Genomes data.

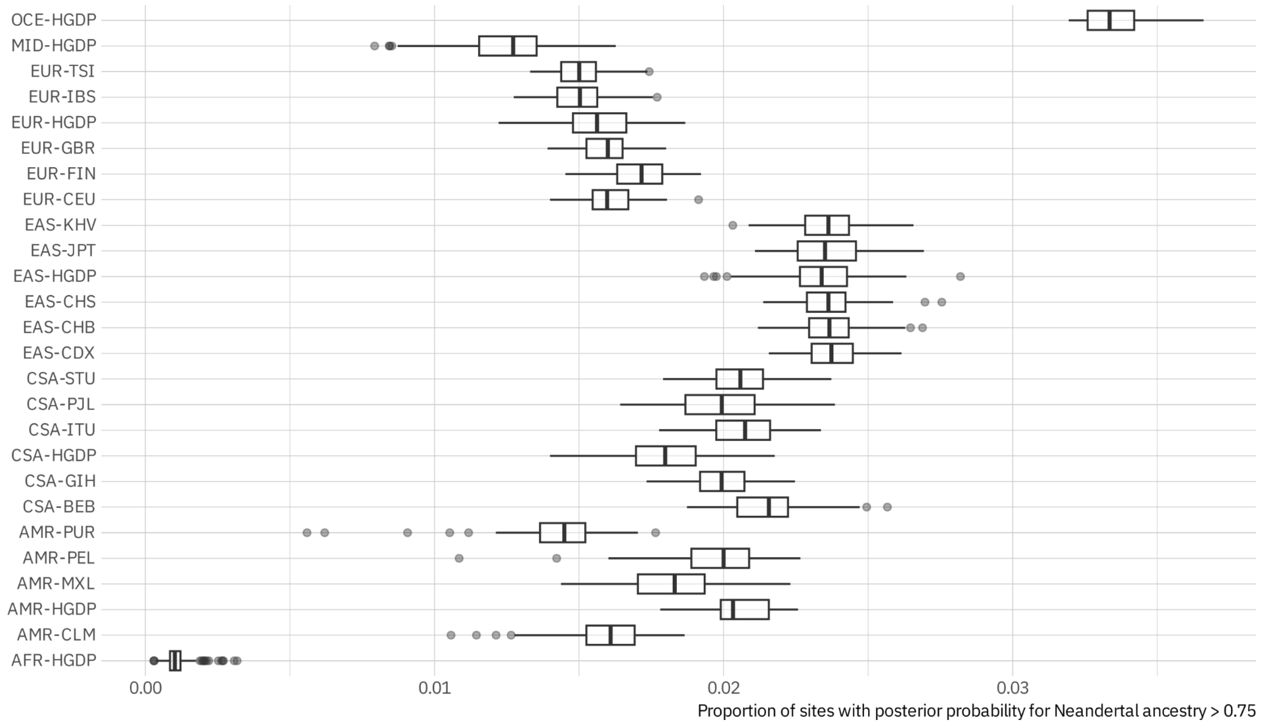

Supplementary Figure 16.2: Proportion of sites with a high probability (> 0.75) of Neandertal ancestry in individuals from the 1000 Genomes and HGDP datasets. Each point represents an individual and the

number of individuals in each population is as the following: AMR-CLM: 94, AMR-HGDP: 49, AMR-MXL: 63, AMR-PEL: 85, AMR-PUR: 104, CSA-BEB: 99, CSA-GIH: 100, CSA-HGDP: 153, CSA-ITU: 102, CSA-PJL: 97, CSA-STU: 98, EAS-CDX: 88, EAS-CHB: 103, EAS-CHS: 103, EAS-HGDP: 217, EAS-JPT: 102, EAS-KHV: 101, EUR-CEU: 121, EUR-FIN: 98, EUR-GBR: 88, EUR-HGDP: 151, EUR-IBS: 104, EUR-TSI: 103, MID-HGDP: 140, OCE-HGDP: 28.

Neandertal fragments and breakpoints

We define two types of breakpoints of Neandertal ancestry: *Start breakpoints* denote the 5' end of Neandertal segments on the positive strand of the human genome and *End breakpoints* the 3' end of such segments. A breakpoint is called when five SNPs of low Neandertal ancestry posterior probability are followed by five SNPs with high probability or vice versa (Supplementary Figure 16.3). Note that this approach may lead to only one of the two breakpoints of a segment to be called.

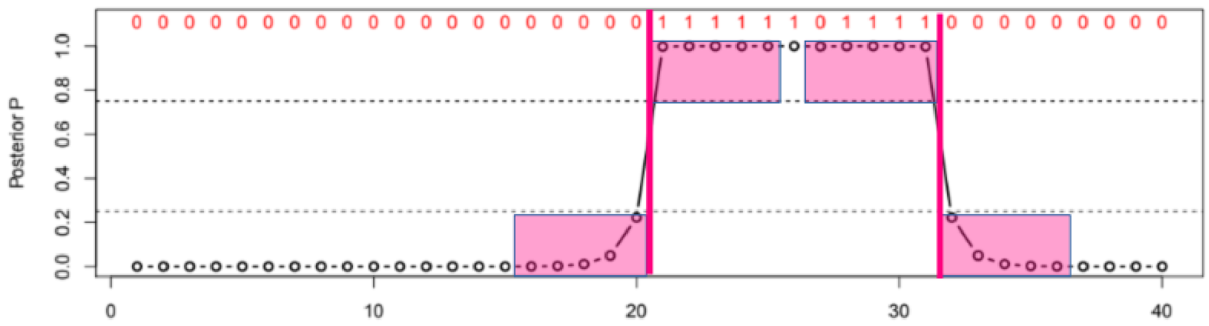

Supplementary Figure 16.3: Example of breakpoint calling. An original sequence of Neandertal informative SNPs is shown at the top as red numbers (1 = Neandertal-shared-derived, 0 = ancestral). Posterior probabilities are shown as a black line with circles. A confident Start breakpoint of a Neandertal segment is called when five SNPs with low probability for Neandertal ancestry (< 0.25) are followed by five SNPs with high probability (> 0.75) (shown as red boxes). The inferred breakpoint lies in between these two sets of SNPs (left bold red line). A confident End breakpoint is inferred when five high-confident SNPs of Neandertal ancestry are followed by five low-confident SNPs.

A breakpoint does not have a single coordinate, but lies in between two SNPs. To minimize the fraction of chance overlaps in our later comparison of breakpoints, we require that the genetic distance between these two SNPs is less than 0.01 cM according to two different recombination maps[79,101].

Ancient and present-day individuals typically yield around 100-300 breakpoints (Supplementary Figure 16.4). More breakpoints per individual are observed for Oceanians, who have additional Denisovan ancestry. African individuals from the HGDP panel that was not used to define

informative SNPs show little to no breakpoints (81/99 individuals have no breakpoints, maximum number of breakpoints in any African individual is 12).

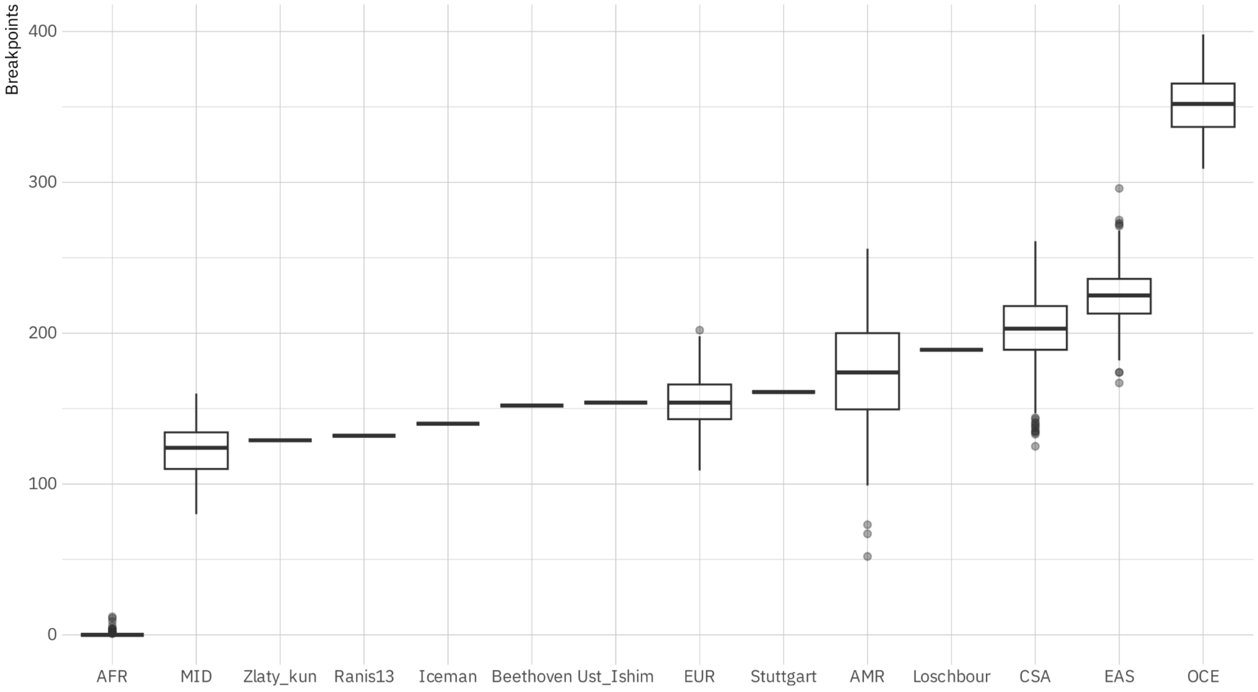

Supplementary Figure 16.4: Number of breakpoints (sum of Start and End breakpoints) identified in present-day populations and ancient individuals. Abbreviations for the populations and the number of individuals in each are the same as in Extended Figure 8C-D.

Sharing of breakpoints

We quantify the degree of sharing of breakpoints with an ancient individual between present-day human populations by calculating the percentage of breakpoints in each present-day individual that is shared with an ancient comparison genome. The type of breakpoint ("Start breakpoint" or "End breakpoint") is required to be identical between present-day and ancient genomes to be counted as a match in this comparison (but see below for a test with mismatching types of breakpoints).

Supplementary Figures 16.5 and 16.6 show the population distribution of the sharing with ancient genomes. As expected, the distribution of breakpoint-sharing with Loschbour, Stuttgart (LBK), and the Tyrolean Iceman are highest for Europeans, followed by Middle Eastern populations. All three ancient individuals share ancestry with later Europeans[38,102] and

comparisons to other populations are statistically significant in a Wilcoxon rank test. (Note, however, that this test may be influenced by error as discussed later in this section.). The smallest difference between Europeans and Middle Easterners is observed for Stuttgart (LBK), an individual with Anatolian Farmer ancestry[38]. The breakpoint-sharing of present-day individuals with the Ranis13, Zlatý kůň, and Ust'-Ishim genomes is lower than for the Tyrolean Iceman, Loschbour, and Stuttgart (LBK) genomes. This is compatible with an older age and an earlier split-time of the former compared with the latter group of genomes. Differences in the distributions of breakpoint-sharing between present-day populations are also less pronounced for the older group although individual comparisons are still found to be significant using a Wilcoxon rank test.

We also calculated the fraction of breakpoints shared in pairwise comparisons of ancient genomes (Supplementary Figure 16.7). As expected from their close genetic relationship, the highest fraction of shared breakpoints is observed in the comparison of Ranis13 and Zlatý kůň. The approximately 10% of Western Hunter Gatherer ancestry in the Tyrolean Iceman[102] offers an explanation for the increased sharing of breakpoints between Tyrolean Iceman and Loschbour. Higher sharing is also observed between the Stuttgart (LBK), Tyrolean Iceman, and Beethoven[103] genomes (included here as a control) who share Anatolian Farmer ancestry.

Recombination events will generate two gametes that carry opposing parental segments along the chromosomes. However, these gametes are not expected to be inherited by the next generation. On the father's side, a vanishingly small chance exists where the exact two gametes form the paternally inherited part of the genomes of dizygotic twins. Viable twins from two maternal gametes with opposing parental segments have never been observed[104–106]. This means that any observed overlap between opposing types of Neandertal segment breakpoints (i.e. End breakpoints matching Start breakpoints or vice versa) are due to error or chance overlaps caused by independent recombination events. Based on this insight, we can further quantify what fraction of shared breakpoints are expected by error or independent recombination by limiting the analysis to matches of different types of breakpoints. In all comparisons, the number of matching opposing types of breakpoints is substantially smaller than that observed for matching types, suggesting that a large proportion (97%, 88%, and 97% in Ranis13, Zlatý kůň, and Ust'-Ishim, respectively) are reflecting true shared ancestry that preserves a common

recombination event (Supplementary Figure 16.8). It is noteworthy that these random overlaps again show significant differences between the population distributions, indicating that these differences are in part driven by unknown sources of error rather than reflecting true differences in the sharing with present-day populations for Ranis13, Zlatý kůň, and Ust'-Ishim.

We conclude that Ranis13, Zlatý kůň, and Ust'-Ishim share Neandertal ancestry breakpoints with present-day populations and therefore share a common Neandertal admixture event with all present-day Out-of-African populations. Since differences in the sharing with different populations are small and inconsistent between Zlatý kůň and Ranis13, and also persist when analyzing sharing between non-matching types of breakpoints, we consider these signals as likely caused by unknown sources of error or bias.

Detailed tables and code for this analysis are available at https://doi.org/10.17617/3.TG4TO4.

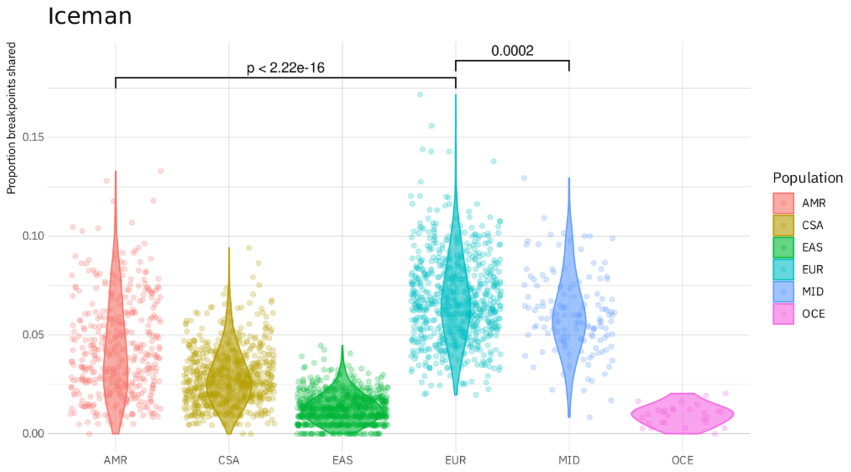

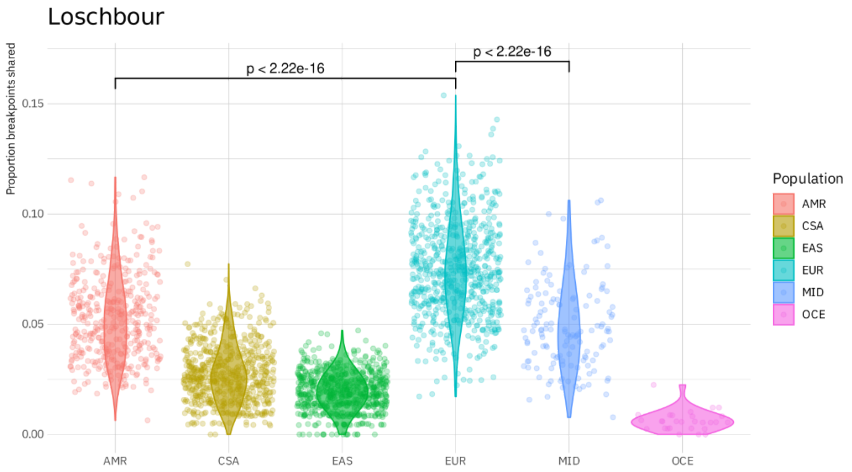

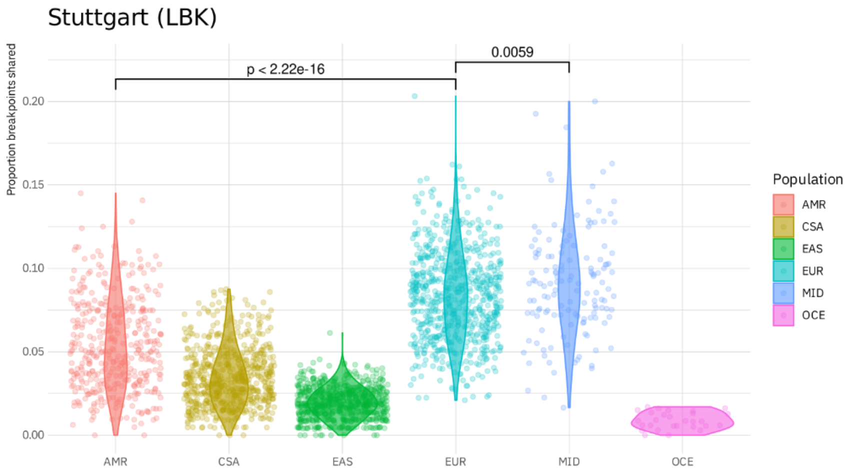

Supplementary Figure 16.5: Fraction of breakpoints in modern genomes shared by ancient genomes. Brackets show the *p-value* of a Wilcoxon rank test on the two population distributions of fractions shared. Abbreviations for the populations and the number of individuals in each are the same as in Extended Figure 8C-D.

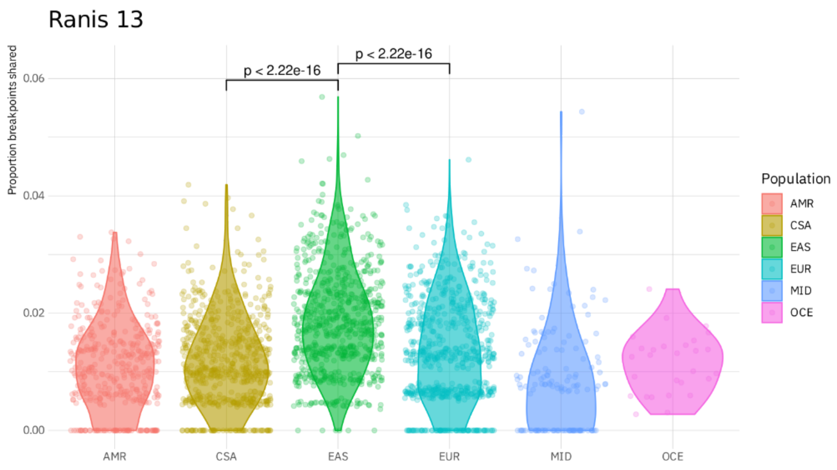

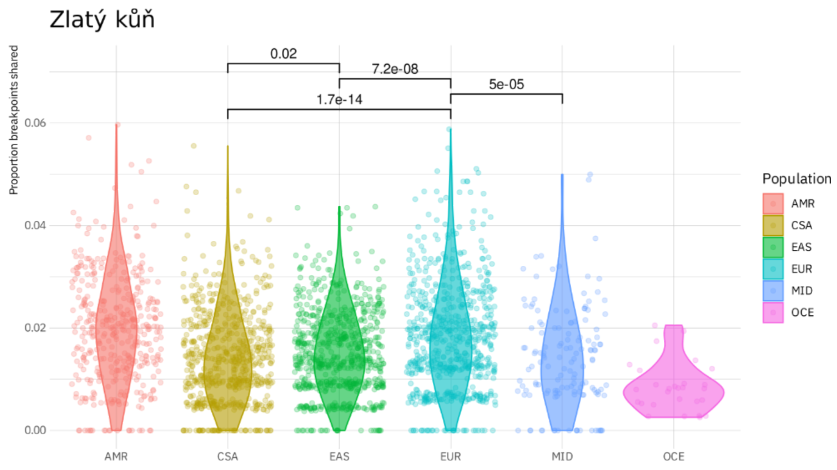

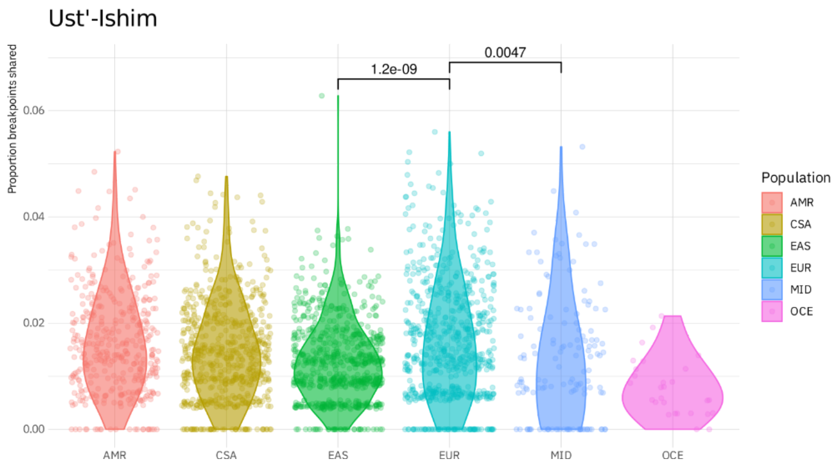

Supplementary Figure 16.6: Fraction of breakpoints in modern genomes shared by ancient genomes. Brackets show the *p-value* of a Wilcoxon rank test on the two population distributions of fractions shared. Abbreviations for the populations and the number of individuals in each are the same as in Extended Figure 8C-D.

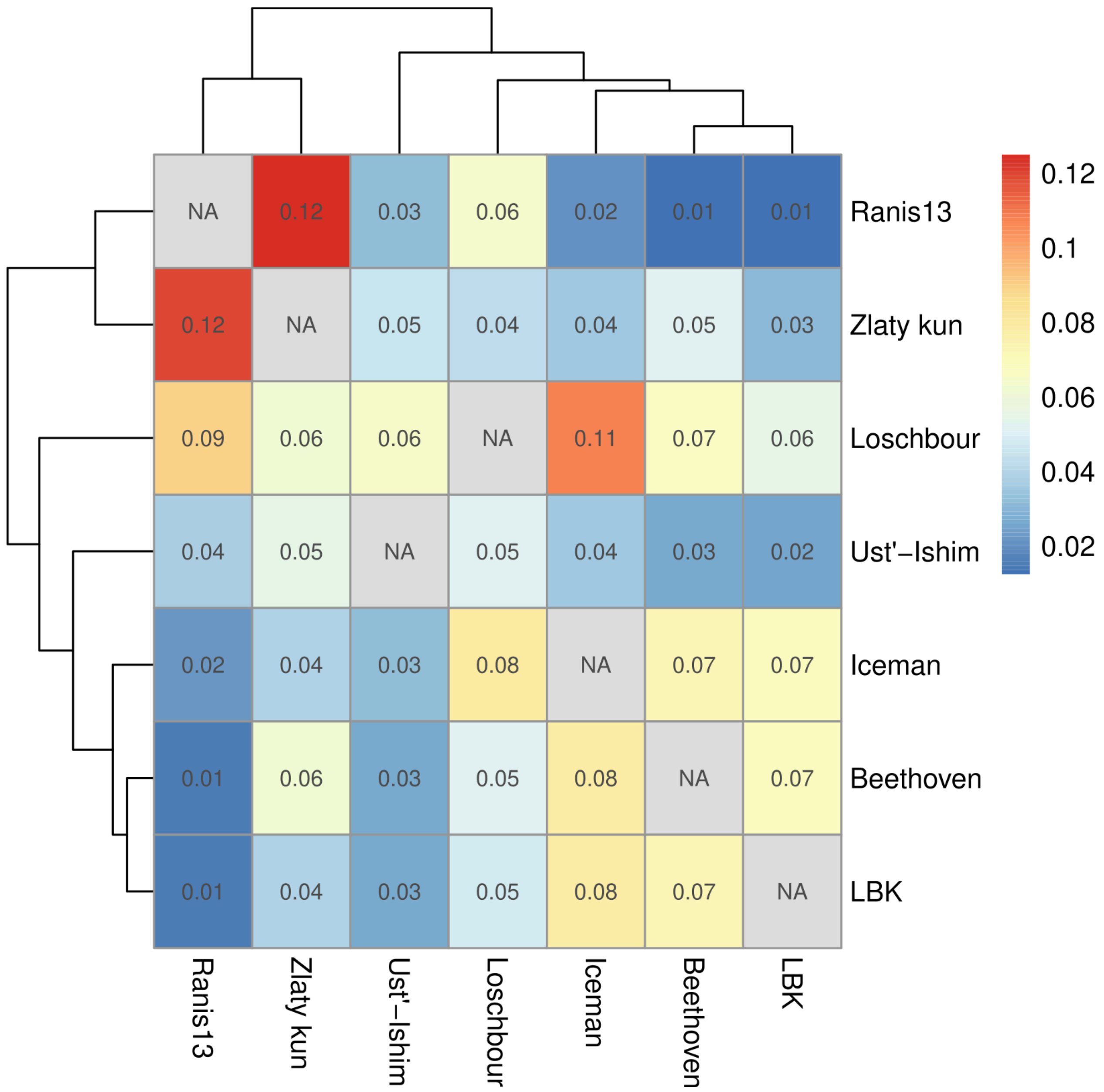

Supplementary Figure 16.7: Pairwise sharing of breakpoints between ancient genomes. The heatmap shows what fraction of breakpoints of the individual shown in the column is shared by the individual in the row.

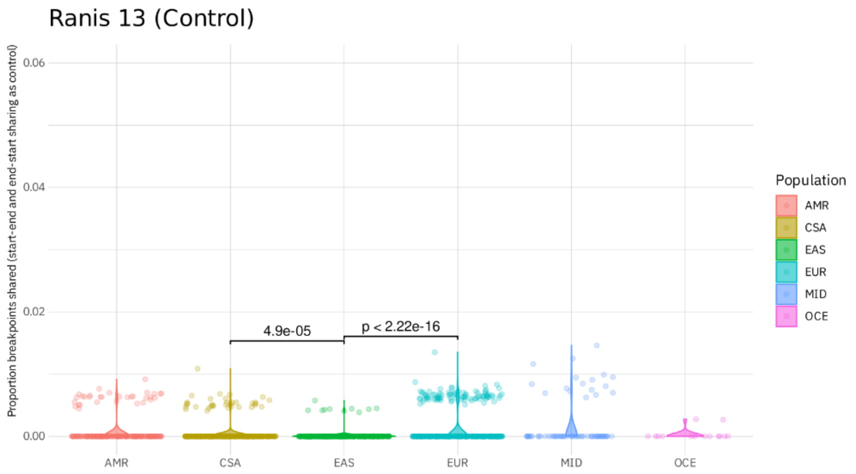

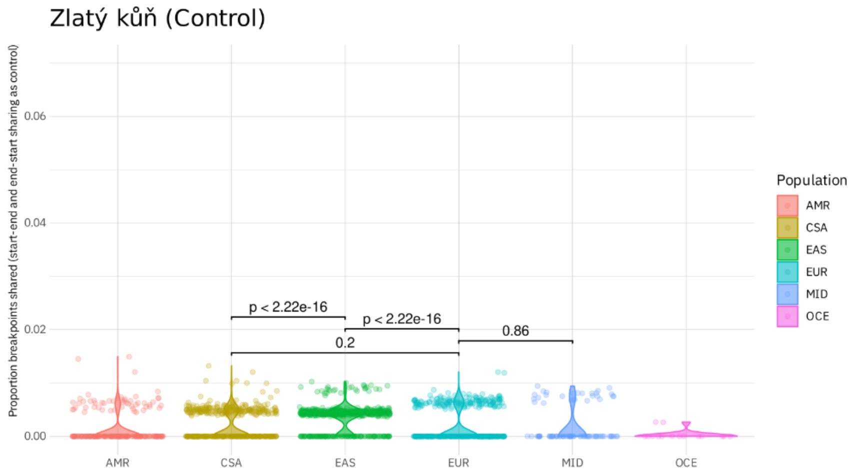

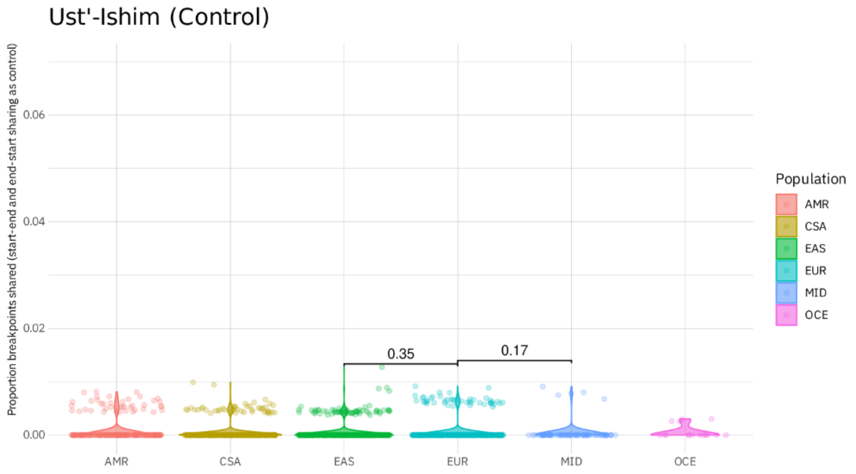

Supplementary Figure 16.8: Fraction of sharing of breakpoints of different types (End breakpoints with Start breakpoints and Start breakpoints with End breakpoints). This sharing is not expected to be generated by true shared recombination events and are used as a measure of false positive breakpoints. See Supplementary Figure 16.6 for comparison. Abbreviations for the populations and the number of individuals in each are the same as in Extended Figure 8C-D.

Supplementary Information 17

Neandertal Deserts

Arev Pelin Sümer

Researchers have previously identified five long regions in the human genome where present-day humans show little to no evidence of introgression from Neandertals or Denisovans[107,108]. These "Neandertal deserts" and "Denisovan deserts" have been suggested to be the result of selection that acted against introgressed archaic alleles[109]. The Initial Upper Palaeolithic individuals from Bacho Kiro Cave and the Oase 1 individual from Romania[8-41] do not carry Neandertal DNA in these desert regions either, even though some of the individuals have a Neandertal ancestor within around five generations[41]. However, previous analysis of the low coverage Zlatý kůň genome showed that this individual carries Neandertal ancestry (chr1:112723662-112855462) within one of the previously reported Neandertal desert regions (chr1:102200000-114900000), indicating that the Neandertal ancestry may not have been completely removed yet in the desert regions at the time Zlatý kůň lived[3]. Here we test whether the high coverage Ranis13, Zlatý kůň, and Ust'-Ishim genomes and the low coverage Ranis87 and Ranis12 genomic data show evidence of introgression in the archaic deserts.

We used the segments of Neandertal ancestry identified by admixfrog version 0.7.1[77], using two different run penalties (0.1 and 0.25), which controls how likely nearby segments are joined or broken up, and can affect the length of the called segment, as explained in Supplementary Information 13. We employed these two run parameters to assess the influence of this parameter, as the detected segment lengths will differ. Segments called with run parameter 0.1 are comparably shorter than the ones called with run parameter 0.25. We then compared the inferred Neandertal segments with a set of five archaic desert regions obtained by merging previously reported deserts of both Neandertal and Denisovan ancestry[107,108] (Supplementary Table 17.4).

Supplementary Table 17.1: Coordinates of the large regions depleted of both Neandertal and Denisovan ancestry (deserts). Deserts are included in the table if the region was called by either Vernot et al., 2016[108] or Sankararaman et al., 2016[107]. Start position and end position give the smallest and largest coordinates of the reported regions from these two publications, respectively (the union).

Chromosome	Start Position	End Position
1	99,000,000	114,900,000
3	76,500,000	90,700,000
7	108,000,000	128,000,000
8	54,500,000	65,400,000
13	49,000,000	61,000,000

We were not able to identify any fragments with Neandertal ancestry in the high coverage Ranis13 genome in any of the previously reported deserts (Supplementary Table 17.1). A Neandertal segment on chromosome 7 was detected when using a run penalty of 0.1, between chr7:125119508-125123764 and of 0.01 cM in length. The length of this segment is shorter than the cutoff we use to avoid ILS (0.2 cM) and hence we did not consider it as a reliable signal. We also analyzed archaic admixture capture data from Ranis87 and Ranis12, and found no evidence of Neandertal ancestry in the desert regions.

We found that Zlatý kůň shares the deserts on chromosomes 3, 7, 8, and 13 with Ranis13, supporting the previous observation[3]. Furthermore, we again identified the previously reported Neandertal segment in the desert of chromosome 1[3] in the high coverage Zlatý kůň genome. Using a run penalty of 0.25, this fragment is 0.24 cM long (chr1:112694993-112857040), and thirteen homozygous sites that match the Neandertal genome support the presence of a Neandertal haplotype (Supplementary Figure 17.1). We observe this segment also using the more conservative run penalty of 0.1, with the 0.225 cM long Neandertal fragment falling at coordinates chr1:112696030-112855127.

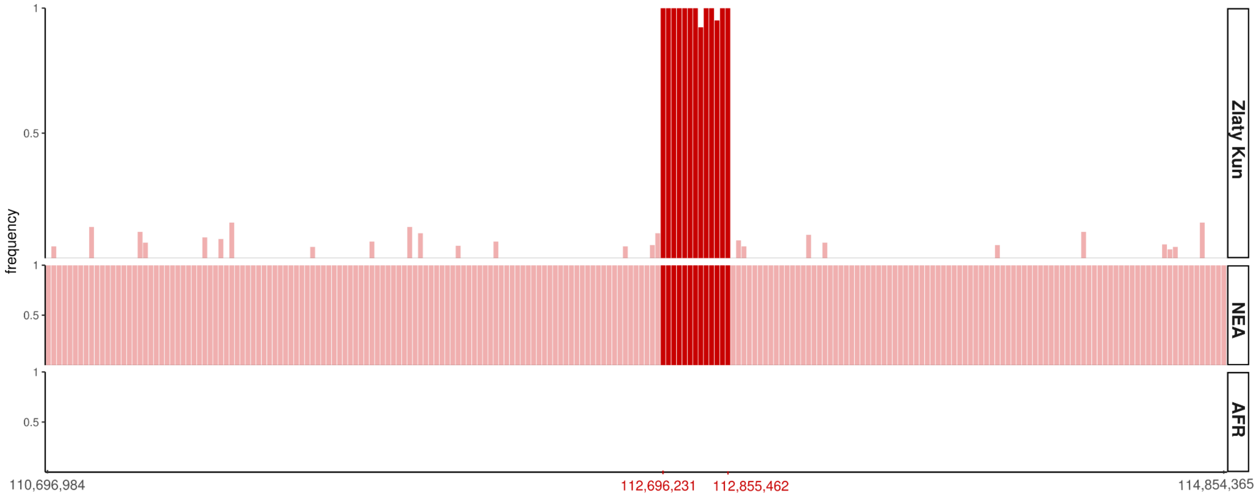

Supplementary Figure 17.1: Neandertal segment in the high coverage Zlatý kůň genome that falls within the desert region of chromosome 1. Light and dark reds represent heterozygous and homozygous Neandertal calls, respectively. The plot was generated for admixfrog segments found using the run penalty of 0.25.

Supplementary Information 18

Y Chromosome Analysis

Adam Benjamin Rohrlach

We identified four of the Ranis specimens to be originating from genetically male individuals, including the one from Ranis13, from which we obtained a high coverage genome. Libraries from one of the individuals, Ranis86, contained low amounts of endogenous DNA and was highly contaminated with present-day human DNA (Table S1.5), hence we excluded this individual from further analysis. We used two capture assays to enrich for Y chromosomal sequences from the remaining two individuals, Ranis10 and Ranis87, in order to analyze them along with the Y chromosome of Ranis13, which we obtained from the high coverage shotgun genome.

The two capture assays we used are the Y chromosome capture design published in Petr et al., 2020[9], which targets 6.9 Mb of the Y chromosome with high alignability, and the YMCA (Y-mappable capture assay) designed and published in Rohrlach et al., 2021[10], which targets ~10.4 Mb in the regions of the non-recombining portion of the Y chromosome that are "mappable" for short reads characteristic of aDNA studies. Three libraries were captured with the former assay targeting 6.9 Mb, and five using YMCA, and filtered as described in Supplementary Information 3, Table S1.5).

All in-house bam files were filtered for reads mapping to the Y chromosome, then sequence data from the same libraries were merged, and deduplication was repeated on the merged bam files. Finally, bam files from different libraries of the same individuals were merged. We then created pileups of the merged bam files for sites found on the Y-SNP list from the ISOGG[110] (International Society of Genetic Genealogy) collection (v. 15.73) included in the 1240k and Y-capture probes. Pileups were further filtered by restricting the analysis of C, T variants to reads aligning in reverse orientation and G, A variants to those aligning in forward orientation to avoid damage-associated errors. We then manually called the most resolved Y haplogroup for which we have derived calls, and minimal ancestral calls. We also manually inspected the position of a

called nucleotide on the individual reads and the associated sequencing quality for uncertain cases.

Haplogroup detection

We found that Ranis10 fell within haplogroup F but did not have any hits for F-M89, unlike Bacho Kiro F6-620. We detected no hits on GHIJK, however we observed ancestral calls for both branches G and HIJK, indicating that Ranis10 is basal to all haplogroups included in GHIJK. Haplogroup F has a T_{MRCA} of ~48,800 years before present[111].

Ranis13 and Ranis87 were assigned to haplogroup NO [K2a], matching the haplogroup assignment of Ust'-Ishim. However, when comparing the topology of the Y chromosome tree at NO [K2a] in Yfull[111], we found that NO [K2a] can be separated into two subgroups, denoted K-M2308 and K-M2335, where K-M2308 is the parent branch of K-M2335. We found that Ranis13 and Ranis87 yielded derived calls for both haplogroups, whereas Ust'-Ishim yielded only derived calls for the more basal K-M2308. Haplogroup K-M2335 has a T_{MRCA} of between 45,600 and 37,400 years before present. Interestingly, Ranis13 and Ranis87 date to between 46,580 to 43,400 years BP and 49,540 to 44,850 years BP, respectively, meaning that these individuals lived either before, or close to the most recent common ancestor of K-M2335, and hence this estimate could be updated to include these individuals. We also observed that Ranis13 and Ranis87 were ancestral for all downstream branches, and are thus basal for K-M2335 (Supplementary Figure 18.1).

To answer the question of whether Ranis13 and Ranis87 formed an extinct but continuous paternal lineage, we looked for all sites which were covered by reads for Ust’-Ishim, Ranis13 and Ranis87, and compared these to an individual from haplogroup A-L963. We then counted the number of unique mutations shared by Ranis13 and Ranis87 that were not shared by Ust’-Ishim. We then compared this to the number of mutations shared by Ranis13 and Ust’-Ishim (but not Ranis87), and the number shared by Ranis87 and Ust’-Ishim (but not Ranis13). To account for the effect of DNA deamination, we omitted any reads with C->T or G->A transversions on the forward and reverse strands, respectively.

We observed that Ranis13 and Ranis87 shared five mutations that Ust'-Ishim did not, encouragingly, three of which were known SNPs belonging to NO-M2335. We note that Ranis87 and Ust'-Ishim share no such mutations, but that Ranis13 and Ust'-Ishim shared one. Hence, we find no strong evidence to suggest that Ranis13 and Ranis87 belong to the same paternal lineage.

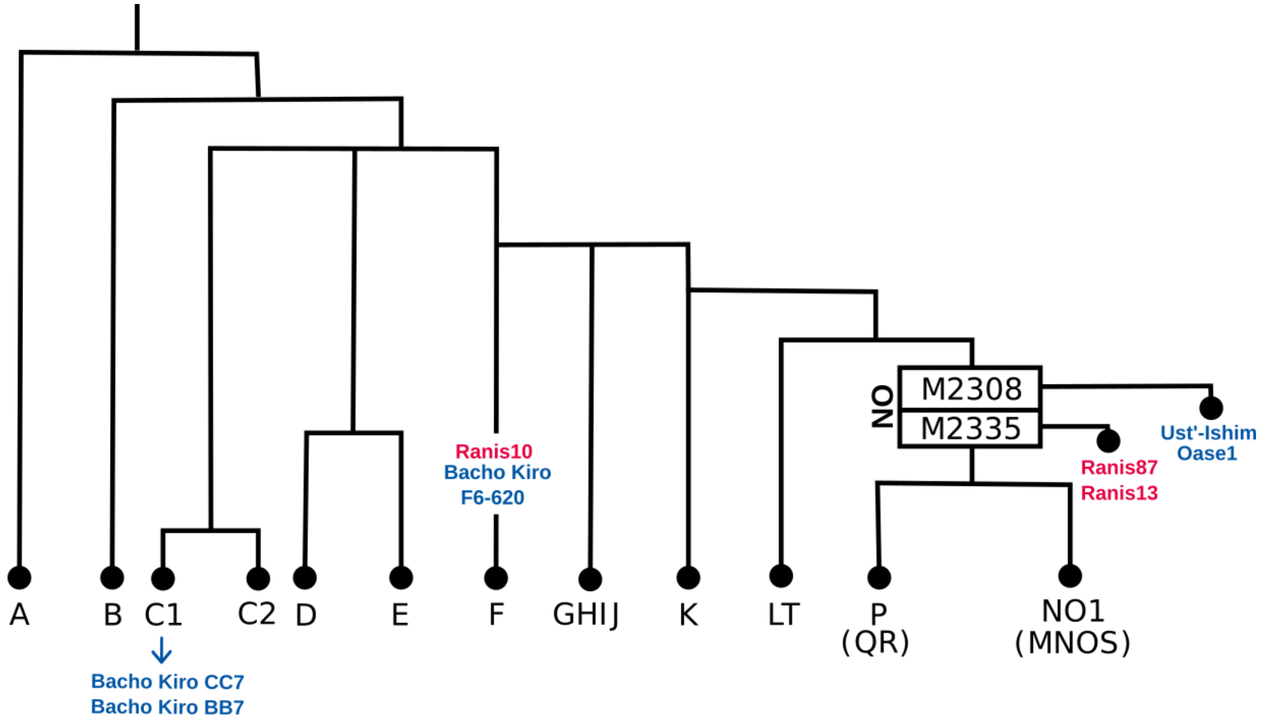

Supplementary Figure 18.1: Phylogenetic tree for the Y chromosome with the Ranis samples placed at likely positions. Note that we represent haplogroup NO (ISOGG) in the YFull representation by splitting this haplogroup into two separate but contiguous branches.

Supplementary Information 19

The Human Leukocyte Antigen (HLA) Analysis

Federica Pierini

HLA haplotypes of Ranis13 and Zlatý kůň

We inferred HLA haplotypes at the five most polymorphic HLA loci (HLA-A, -B, -C, -DRB1, and –DQB1) for Zlatý kůň and Ranis13 from sequence data obtained from all available libraries. Pre-mapping steps differed between Zlatý kůň and Ranis13:

- **Zlatý kůň**. One selected library (ZKU002.A0101) was hybridized in-solution to a molecular bait panel designed to enrich exome sequences of 488 human immunity genes, including allelic variants of the highly polymorphic HLA region[112]. After capture, the enriched library was paired-end sequenced to a depth of ~20 million reads on the HiSeq 4000 sequencer at the former Max Planck Institute for the Science of Human History in Jena, Germany. Raw sequencing data resulting from capture enrichment and shotgun sequencing of all available libraries (ZKU002.A0101, ZKU002.A0102, and ZKU002.A0103) were combined after adaptor removal and merging of forward and reverse reads as described in Supplementary Information 4.

- **Ranis13**. Raw data resulting from deep sequencing of the four pooled libraries (RNI013.A0101, RNI013.A0201, RNI013.A0301, and RNI013.A0401) were combined after adaptor removal and merging of forward and reverse reads as described in Supplementary Information 4.

The presence of paralogous and highly polymorphic sequences along the HLA region can cause mapping bias when genotyping HLA genes with standard genotyping pipeline[113]. Therefore, the specificity of HLA genetic diversity was here taken into account to prevent alignment bias. Merged fastq files were filtered to remove reads shorter than 30 base pairs. Following the method described in Pierini et al. 2020[114], sequence data were aligned to an HLA reference file using Bowtie2 (v. 2.2.6)[115] in local alignment mode. The HLA reference file (accessible at https://targt-pipeline.sourceforge.io/) includes all known nucleotide sequence variants of the

exons coding for the peptide-binding groove of the classical class I (exons 2 and 3; HLA-A, HLA-B, HLA-C) and class II HLA genes (exon 2; HLA-DRB1, HLA-DQA1, HLA-DQB1, HLA-DPA, HLA-DPB1) as well as sequence variants of non-classical HLA genes. Nucleotide coding sequences included in the reference file were downloaded from the IPD-IMGT/HLA database[116] (Release 3.21, 2015-07) and processed as described in the original publication[114].

During the mapping phase, each read could map multiple haplotypes at one or more HLA loci in the reference (-a). This critical step was introduced to prevent mismapping and misclassification of reads caused by the high level of sequence similarity across HLA genes. We applied a maximum mismatch threshold of 1% (–score-min L,0,0.99), and a local alignment matching bonus of 1 (–ma 1) while the maximum (MX) and minimum (MN) mismatch penalties were set equal to 0 (–mp 0,0). As the HLA reference file includes N nucleotides in the flanking region of the exons, the penalty for ambiguous characters such as N was fixed to 0 (--np 0). BAM files were further processed through an automated read sorting step, in which all reads mapping to a specific HLA gene were saved into an individual fasta file, following the mapping position along each exon. Alignment files can be accessed through https://doi.org/10.17617/3.GHAALO. Outputs of the sorting phase were manually inspected to reconstruct, at each locus, the consensus sequences of the two haplotypes while considering only reads that map uniquely to the locus of interest. Consensus sequences were finally compared to known 4-digit HLA alleles to find the best-matching sequences and thus define allele calls. The average read depth at each locus was quantified as the sum of read lengths divided by the total length of the region under investigation (exon2+exon3 for class I loci and exon2 for class II). To account for the large number of sequences mapping to multiple HLA loci, read lengths were weighted by the number of alignments to different loci. Average HLA coverage was quantified as the mean percentage of covered sites at each locus. High-resolution typing results are commonly reached through good coverage along the exons encoding the antigen binding site of HLA molecules (exons 2 and 3 for class I and exon 2 for class II). This usually allows the definition of nonsynonymous changes within the regions of interest and, thus the reconstruction of unique amino acid sequences to which an individual two-field allele name can be assigned. Following the specific HLA nomenclature, established by the IPD-IMGT/HLA database[116], two-field resolution allele calls were reported throughout our work.

HLA allele calls at the five most polymorphic HLA loci (HLA-A, -B, -C, -DRB1, and -DQB1) for the two ancient individuals can be found in Table S5.1, together with the average read depth, average coverage, and the number of sites not covered at each locus.

Homozygosity at HLA loci in worldwide human populations

HLA genotype information for worldwide human populations with available data across the five investigated HLA genes were downloaded from the allele frequency net database (AFND; http://www.allelefrequencies.net/BrowseGenotype.aspx)[117]. We next quantified the proportion of individuals in each population with at least three HLA loci showing homozygosity (out of five: -A, -B, -C, -DRB1, and -DQB1) (Table S5.2). Fisher's exact test was applied to test the probability of observing, in a given modern population, the same states as Ranis13 and Zlatý kůň (i.e. one genome with at least three homozygote haplotypes and one genome with less than three homozygote haplotypes) (Table S5.2).

HLA allele frequency in worldwide human populations

We used the Bayesian framework implemented in the HLAfreq Python package[118] to estimate the combined HLA allele frequency from multiple datasets that belong to the same geographical region (Sub-Saharan Africa, North Africa, Europe, Western Asia, South Asia, North-East Asia, South-East Asia, Oceania, Australia, North America, South and Central America). To quantify both point estimates of allele frequency and credible intervals, we applied the compound model, which allows us to take into account the variance between datasets (Table S5.3). The default 's' option was used to filter study quality and include all datasets classified as silver in the AFND database (i.e. in which allele frequencies sum to 1 and without restrictions in terms of sample size). To apply a more stringent filter, only studies reaching the two-field resolution level were included; if higher, the resolution was reduced to the two-field level to maintain consistency across datasets.

HLA allele frequency in ancient European populations

To explore the prevalence of the observed haplotypes in other prehistoric and historic populations, we collected available data at five HLA genes (HLA-A, -B, -C, -DRB1, and -DQB1) from published ancient datasets. Allele frequencies were quantified for two medieval

datasets, the Ellwangen mass grave in Germany (ELW)[112] and the St. Jørgen leprosarium in Denmark (DEN)[114], as well as for the Early Neolithic site of Derenburg in Germany (DER)[119]. Frequencies were obtained by direct counting, focusing only on two field allele calls and removing NA calls. HLA allele frequencies of early and late Neolithic populations of farmers, as reported in da Silva et al., 2023[120], were also included in our comparison (EF and LF respectively) (Table S5.4).

Results

Haplotypes at five HLA genes (HLA-A, -B, -C, -DRB1, and –DQB1) were investigated in the genomes of Ranis13 and Zlatý kůň. At all HLA loci explored, the average depth of coverage was above 7x, whereas the percentage of sites covered along the exons of interest was above 92%. Thanks to the high number of retrieved sequences covering the HLA region, allele typing at the 2nd field level of resolution was possible in all cases (Table S5.1).

One homozygous genotype was found in Zlatý kůň at the HLA–B locus, while three homozygous genotypes were observed in Ranis13 at the HLA-A, -DRB1, and -DQB1 loci. HLA genotyping might be affected by low coverage, resulting in inaccurate estimate of homozygosity due to allelic dropout. The lowest coverage in our analysis was observed at the locus HLA-DQB1 in the Zlatý kůň genome (6.7). Nevertheless, at this gene, a heterozygote call was defined, suggesting that coverage as low as the one obtained is still reliable for assigning zygosity levels. Yet, the cutoff of reliability might be locus-specific; we, therefore, performed a downsampling experiment to test whether the zygosity we observe at the remaining two homozygote loci in Ranis13 (HLA-A and HLA-DRB1) is affected by low coverage. To do so, the coverage of Zlatý kůň was reduced by 80%. Following the downsampling process, read depths of 7.1 and 5.3 were reached at the HLA-A and HLA-DRB1 genes, respectively. After genotyping, a reduction in the typing resolution was observed, with multiple alleles sharing the first field being equally valid when calling both haplotypes (Table S5.1). Notably, even with low genotyping resolution (i.e. first field allele calls), the definition of heterozygote genotypes at both loci was still possible, suggesting that the coverage reduction did not affect the zygosity definition. Thus, the observed homozygous genotypes in Ranis13 are unlikely the result of allelic dropout.

We found that the proportion of individuals having three or more homozygous genotypes (out of five) is generally very low in modern populations, with increased values found only in a few groups (Supplementary Figure 19.1 and Table S5.2). In line with this evidence, Fisher's exact test suggests that the probability of observing the same level of homozygosity we observe in the ancient samples is not a likely event in modern-day populations. Notably, high levels of homozygosity like those we observe, are more common in modern groups for which a history of isolation has been described (Wiwa and Chimila Amerindian populations from the NorthColombia coast, as well as Lacandon and Otomi populations from Mexico)[121–126] (Supplementary Figure 19.1 and Table S5.2). Despite the high level of homozygosity, HLA segments did not fall within runs of homozygosity (ROH) regions detected in both Ranis13 and Zlatý kůň (Tables S3.2-S3.3). Overall, these results suggest that the observed level of homozygosity at HLA is probably not the outcome of close and recent family relationships (i.e. consanguinity), but is more likely due to isolation events and a small population size.

We obtained fourteen different haplotypes in total, of which only two were shared by the two genomes (DRB1*14:01 and DQB1*05:03). When exploring HLA allele frequency in modern human populations worldwide, we found that five alleles observed in the ancient genomes have a frequency lower than 5% in all geographical regions worldwide (B*18:01, B*39:01, B*56:01, C*07:04, and C*12:03); thus, they can be considered rare nowadays (Supplementary Figures 19.2 and Supplementary Table 19.3). An overall worldwide low frequency was also observed for three more alleles (A*31:01, DRB1*12:01, and DRB1*14:01), slightly exceeding the 5% frequency cutoff in only one region each (Supplementary Figures 19.2 and Supplementary Table 19.3). Only two alleles, A*02:01 and DQB1*03:01, are currently shared by many human populations, showing frequencies higher than 5% across all geographical regions (except for A*02:01, which has a frequency lower than 5% in North Africa). The remaining alleles (A*24:02, C*01:02, C*07:02, and DQB1*05:03) were found at higher frequencies in modern non-African populations than in African populations. For all alleles, we observed an overall tendency to be more frequent in one or more geographical regions outside Africa (Supplementary Figure 19.2 and Table S5.3).

In recent years, a growing number of studies have focused their attention on the analysis of HLA genes in prehistoric and historic populations. The totality of such investigations concentrates on

remains collected from European archaeological sites, offering a resource for comparative analysis of HLA alleles in past European populations[112,119,120,127–129]. All the HLA alleles we detected in Ranis13 and Zlatý kůň have been previously identified in ancient European samples (Supplementary Figure 19.3 and Table S5.4). Thus, they were most likely present in other Early Modern Human groups and found at varying frequencies in modern humans in Europe over time. Three alleles observed in Ranis13 and Zlatý kůň (A*02:01, A*24:02, and DQB1*03:01) were described in all previous studies with frequencies higher than 5%, suggesting their presence in Europe as common variants over prolonged periods. Notably, they also show a gradual decrease in frequency starting from the Early Neolithic period until modern times, which might be the result of admixture and/or selective events, as previously suggested for other HLA variants[112,120,127,128]. On the other hand, five of the alleles described in our work (B*18:01, B*56:01, C*07:04, DQB1*05:03, and DRB1*14:01) have been reported at low frequencies in past European populations, likely indicating that they were maintained as rare variants over millennia (Supplementary Figure 19.3 and Table S5.4). HLA frequency changes between different epochs have been described in previous work[120,128]. Nevertheless, the precise dynamics of allele frequency fluctuation at the HLA genes through time remain largely unexplored. Larger sample sizes from each epoch and additional information from other relevant time periods and geographical regions of the world will allow for in-depth interpretations of HLA allele frequency trajectories.

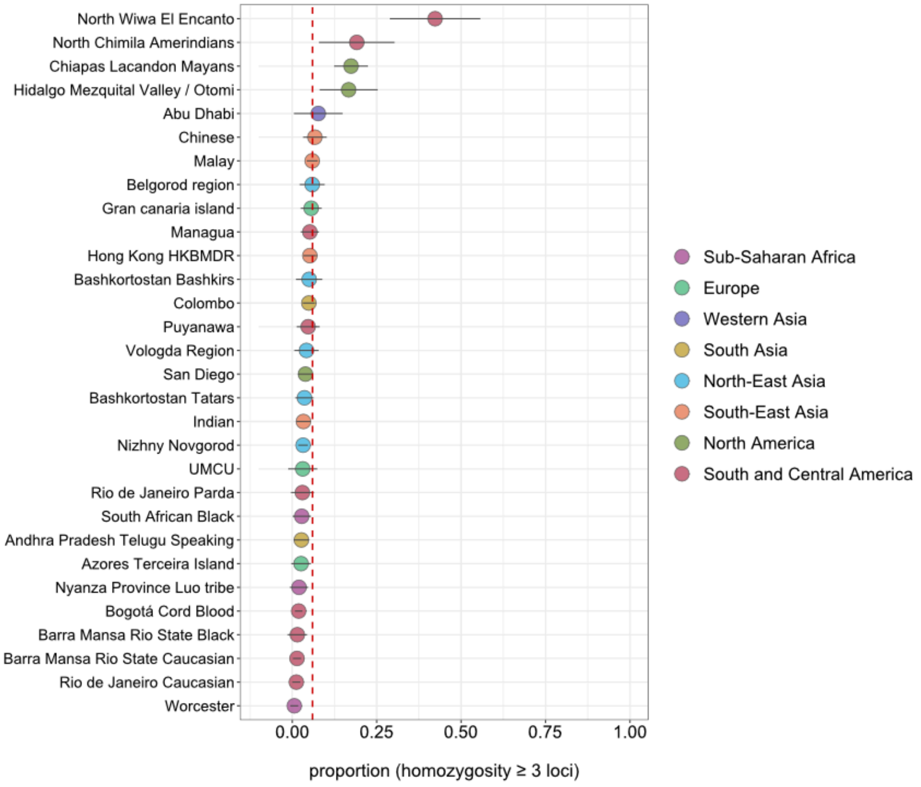

Supplementary Figure 19.1: Proportion of individuals showing at least three HLA homozygote genotypes (-A, -B, -C, -DRB1, and -DQB1) reported for modern human populations along with 95% confidence intervals calculated applying a normal approximation to the Binomial distribution. The dashed red line denotes the mean proportion across populations.

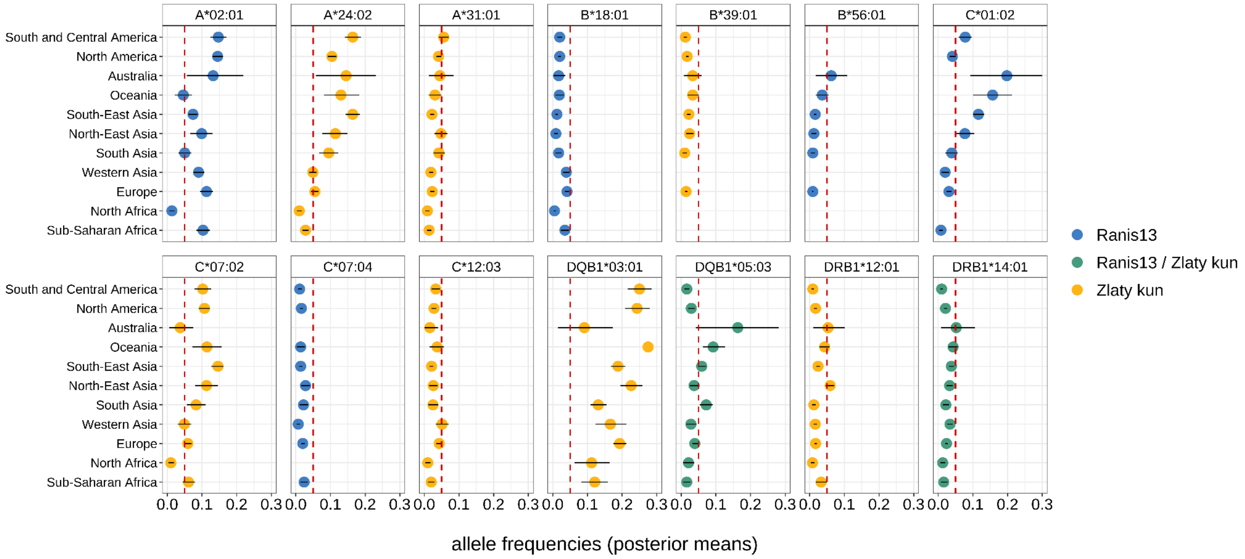

Supplementary Figure 19.2: Point estimates of allele frequency (coloured points) and 95% high density credible intervals (horizontal black lines) quantified using a Bayesian framework implemented in the HLAfreq Python package, and reported for different modern human populations grouped according to

their geographical regions. Credible intervals reflect the variability observed among the combined datasets and provide an indication of the uncertainty associated with the estimated frequency. Only frequencies higher than 1% are reported. Colour codes define haplotypes observed in ancient samples: blue, Ranis13; yellow, Zlatý kůň; green, shared by Ranis13 and Zlatý kůň. The dashed red line denotes 5% frequency.

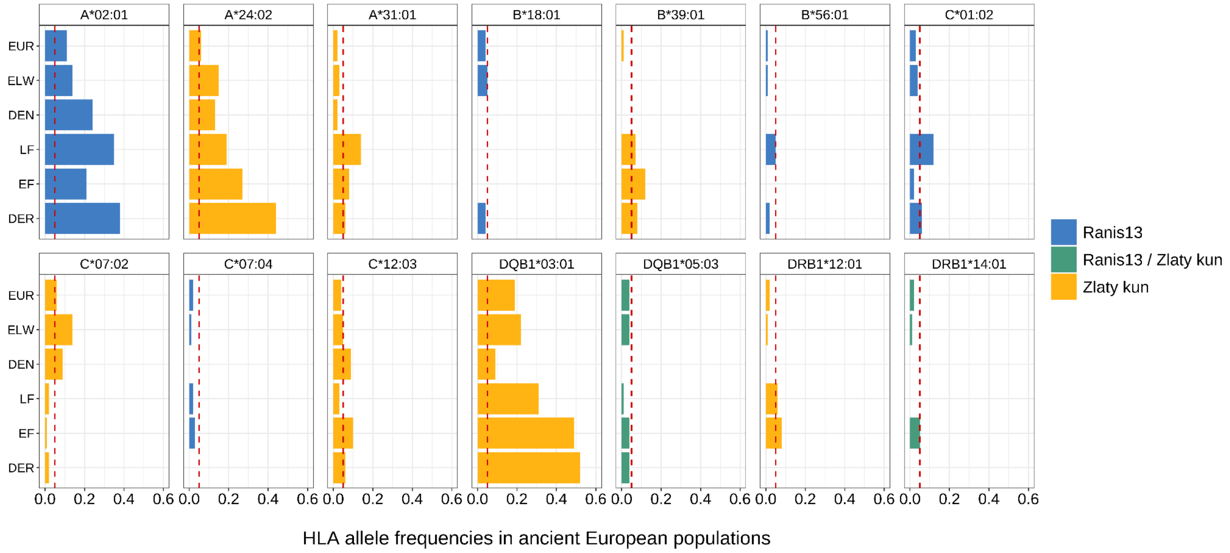

Supplementary Figure 19.3: Allele frequency (coloured bars) quantified in ancient and modern European populations (EUR, modern Europeans; ELW, medieval from Ellwangen in Germany; DEN, medieval from St. Jørgen leprosarium in Denmark; LF, late farmers from Altendorf, Warburg, Rimbeck, and Niedertiefenbach sites in Germany; EF, early farmers from Niederpöring, Fellbach-Öffingen, and Trebur sites in Germany; DER, early farmers from Derenburg in Germany). Colour codes define haplotypes observed in the ancient specimens analyzed in this study: blue, Ranis13; yellow, Zlatý kůň; green, shared by Ranis13 and Zlatý kůň. The dashed red line denotes 5% frequency.

Supplementary Information 20

Phenotypic Inference

Arev Pelin Sümer

We investigated 43 variants that are associated with phenotypic variation (Supplementary Table 20.1). After filtering the sequences from our high-coverage genomes as detailed previously, we counted the number of sequences carrying the effect allele versus the non-effect allele for each variant (Supplementary Table 20.2). In addition, we investigated the same set of variants in the low coverage genomes (≥ 1x coverage on the 1240k sites) of Ranis4, Ranis12, and Ranis87. Due to relatively high contamination in all sequences of Ranis12, we additionally analyzed only deaminated sequences for this individual (Table S6.4).

Supplementary Table 20.1: List of phenotypic variants with genes and their corresponding genomic positions.

Gene	Reference	Chromosome	Position	rs
EDAR	SNPedia[130]	2	109,513,601	rs3827760
MCM6,LCT	Mathieson et al., 2015[7]	2	136,608,646	rs4988235
TLR1,TLR6,TLR10	Mathieson et al., 2015[7]	4	38,815,502	rs4833103
SLC45A2	Mathieson et al., 2015[7], Hirisplex[131]	5	33,951,693	rs16891982
SLC45A2	HIrisPlex[131]	5	33,958,959	rs28777
SLC22A4	Mathieson et al., 2015[7]	5	131,675,864	rs272872
IRF4	HIrisPlex[131]	6	396,321	rs12203592
EXOC2	Branicki et al., 2011[132]	6	457,748	rs4959270
ZKSCAN3,ZSCAN31	Mathieson et al., 2015[7]	6	28,322,296	rs6903823
MHC (EGFL8,PPT2-EGFL8)	Mathieson et al., 2015[7]	6	32,132,233	rs2269424
TYRP1	HIrisPlex[131]	9	12,709,305	rs683
FADS1	Mathieson et al., 2015[7]	11	61,569,830	rs174546
FADS1	Ameur et al., 2012[133]	11	61,570,783	rs174547
FADS1	Ameur et al., 2012[133]	11	61,571,348	rs174548
FADS1	Ameur et al., 2012[133]	11	61,571,382	rs174549
FADS1	Ameur et al., 2012[133]	11	61,571,478	rs174550
FADS1	Ameur et al., 2012[133]	11	61,580,635	rs174556

FADS2	Fumagalli et al., 2015[134]	11	61,597,212	rs174570
FADS2	Ameur et al., 2012[133]	11	61,597,972	rs1535
FADS2	Ameur et al., 2012[133]	11	61,600,342	rs174574
FADS2	Ameur et al., 2012[133]	11	61,603,510	rs174576
FADS3	Fumagalli et al., 2015[134]	11	61,641,717	rs7115739
DHCR7, NADSYN1	Mathieson et al., 2015[7]	11	71,165,625	rs7944926
GRM5	Mathieson et al., 2015[7]	11	88,515,022	rs7119749
TYR	HIrisPlex[131]	11	88,911,696	rs1042602
TYR	Sulem et al., 2007[135]	11	89,011,046	rs1393350
KITLG	HIrisPlex[131]	12	89,328,335	rs12821256
ATXN2,SH2B3	Mathieson et al., 2015[7]	12	112,007,756	rs653178
SLC24A4	HIrisPlex[131]	14	92,773,663	rs12896399
SLC24A4	HIrisPlex[131]	14	92,801,203	rs2402130
OCA2	HIrisPlex[131]	15	28,230,318	rs1800407
HERC2	HIrisPlex[131]	15	28,365,618	rs12913832
MC1R	HIrisPlex[131]	16	89,985,844	rs1805005
MC1R	HIrisPlex[131]	16	89,985,918	rs1805006
MC1R	HIrisPlex[131]	16	89,985,940	rs2228479
MC1R	HIrisPlex[131]	16	89,986,091	rs11547464
MC1R	HIrisPlex[131]	16	89,986,117	rs1805007
MC1R	HIrisPlex[131]	16	89,986,130	rs1110400
MC1R	HIrisPlex[131]	16	89,986,144	rs1805008
MC1R	HIrisPlex[131]	16	89,986,154	rs885479
MC1R	HIrisPlex[131]	16	89,986,546	rs1805009
ASIP/PIGU	HIrisPlex[131]	20	33,218,090	rs2378249
PNPLA3	SNPedia[130]	22	44,324,727	rs738409

The derived variant of rs16891982 on SLC45A2 is associated with light skin pigmentation. In line with the results from the low coverage Zlatý kůň data[3], we find that sequences covering this position support the ancestral and non-effect allele. There was only one read carrying the derived allele G, likely due to sequencing error, which was not reflected in the genotype call at this position (Supplementary Table 20.2). For Ranis13, all reads overlapping this variant carried the ancestral, non-effective allele. Another variant, rs28777, in the same gene is associated with darker hair/skin. Both Ranis13 and Zlatý kůň carry the effect allele C in a homozygous state, supported by 26 and 21 reads, respectively. In both high coverage genomes, sequences

overlapping SLC24A4 (rs12896399) and HERC2 (rs12913832) support a dark-eyed phenotype. TYR (rs1393350) was filtered out in both high coverage genomes and MC1R (rs1805005) was filtered out in Ranis13 during genotype calling, as these positions did not fall within the 2.5% extremes of the GC-corrected coverage distribution.

The ancestral allele G of rs4988235 in MCM6, LCT is associated with lactose intolerance; both Ranis13 and Zlatý kůň are homozygous for this allele. For the low coverage genomes of Ranis4, Ranis12, and Ranis87, we found similar patterns, supporting darker hair/skin and lactose intolerance (Tables S6.3-S6.5).

Supplementary Table 20.2: Read counts of the effective and non-effective alleles for the given phenotypic effect in the high coverage genomes of Ranis13 and Zlatý kůň, excluding the FADS genes. The full table can be viewed at Tables S6.1-S6.2.

Gene	Chr.	Position	rs	Non-effect allele	Effect allele	Ranis13			Zlatý kůň			Phenotypic effect
						Non-effect	Effect	Genotype call	Non-effect	Effect	Genotype call	
EDAR	2	109,513,601	rs3827760	A	G	24	0	0/0	27	0	0/0	Thicker straighter hair, increased chance of shovel-shaped incisors
MCM6, LCT	2	136,608,646	rs4988235	A	G	1	28	0/0	0	16	0/0	Lactose intolerance
TLR1,TLR6, TLR10	4	38,815,502	rs4833103	A	C	0	22	1/1	0	21	1/1	Immunity (lower risk of Non-Hodgkin's lymphoma)
SLC45A2	5	33,951,693	rs16891982	C	G	25	0	0/0	23	1	0/0	Light skin; Possibly an increased risk of melanoma
SLC45A2	5	33,958,959	rs28777	A	C	0	26	0/0	0	21	0/0	Darker hair/skin
SLC22A4	5	13,167,5864	rs272872	G	A	19	0	1/1	13	11	0/1	Ergothionine transport?
IRF4	6	396,321	rs12203592	C	T	25	0	0/0	26	0	0/0	Presence of freckles, brown hair and high sensitivity of skin to sun exposure
EXOC2	6	457,748	rs4959270	C	A	0	30	1/1	0	19	1/1	Black hair colour[102,132]
ZKSCAN3, ZSCAN31	6	28,322,296	rs6903823	A	G	21	0	0/0	18	0	0/0	Linked to pulmonary function, autophagy, lung cancer
TYRP1	9	12,709,305	rs683	A	C	0	22	0/0	1	18	0/0	Darker skin/hair
DHCR7,NADSYN1	11	71,165,625	rs7944926	A	G	20	0	0/0	31	0	0/0	Vitamin D
GRM5	11	88,515,022	rs7119749	A	G	9	19	0/1	10	16	0/1	Pigmentation
TYR	11	88,911,696	rs1042602	C	A	19	0	0/0	28	0	0/0	Absence of freckles

TYR	11	89,011,046	rs1393350	G	A	26	0	NA	28	2	NA	Blond with blue eyes rather than brown with green eyes[135]
KITLG	12	89,328,335	rs12821256	T	C	25	0	0/0	16	0	0/0	Lighter hair
ATXN2,SH2B3	12	112,007,756	rs653178	T	C	38	0	1/1	26	0	1/1	Celiac disease link
SLC24A4	14	92,773,663	rs12896399	T	G	0	16	0/0	0	22	0/0	Darker eyes
SLC24A4	14	92,801,203	rs2402130	A	G	14	9	0/1	3	24	0/0	Darker hair/skin
OCA2	15	28,230,318	rs1800407	C	T	17	1	0/0	21	3	0/0	Green/hazel/brown eyes, not blue/gray
HERC2	15	28,365,618	rs12913832	G	A	0	35	0/0	0	23	0/0	Brown eyes/not blue
MC1R	16	89,985,844	rs1805005	G	T	14	0	NA	22	0	0/0	Light blond hair
MC1R	16	89,985,918	rs1805006	C	A	18	0	0/0	29	0	0/0	Red hair; Increased risk of melanoma
MC1R	16	89,985,940	rs2228479	G	A	18	0	0/0	21	0	0/0	Red/blond hair
MC1R	16	89,986,091	rs11547464	G	A	14	0	0/0	27	0	0/0	Red hair
MC1R	16	89,986,117	rs1805007	C	T	20	1	0/0	34	2	0/0	Red hair; Increased risk of melanoma; Higher anaesthetic response in homozygous individuals
MC1R	16	89,986,130	rs1110400	T	C	23	0	0/0	32	0	0/0	Lighter pigmentation
MC1R	16	89,986,144	rs1805008	C	T	16	0	0/0	33	0	0/0	Red hair; Increased risk of melanoma
MC1R	16	89,986,154	rs885479	G	A	18	0	0/0	33	0	0/0	Lighter skin
MC1R	16	89,986,546	rs1805009	G	C	25	0	0/0	23	0	0/0	Red hair; Increased risk of melanoma
ASIP/PIGU	20	33,218,090	rs2378249	A	G	15	0	1/1	22	0	1/1	Brown/reddish hair

PNPLA3	22	44,324,727	rs738409	G	C	7	10	0/1	31	0	1/1	Fatty liver disease; Higher risk of damage to liver from alcohol

References

1. Diedrich, C. G. & Žák, K. Prey deposits and den sites of the Upper Pleistocene hyena *Crocuta crocuta spelaea* (Goldfuss, 1823) in horizontal and vertical caves of the Bohemian Karst (Czech Republic). *Bull. Geosci.* **81**, 237–276 (2006).
2. Slon, V. *et al.* Mammalian mitochondrial capture, a tool for rapid screening of DNA preservation in faunal and undiagnostic remains, and its application to Middle Pleistocene specimens from Qesem Cave (Israel). *Quat. Int.* **398**, 210–218 (2016).
3. Prüfer, K. *et al.* A genome sequence from a modern human skull over 45,000 years old from Zlatý kůň in Czechia. *Nat. Ecol. Evol.* **5**, 820–825 (2021).
4. Slon, V. *et al.* Neandertal and Denisovan DNA from Pleistocene sediments. *Science* **356**, 605–608 (2017).
5. Huson, D. H., Auch, A. F., Qi, J. & Schuster, S. C. MEGAN analysis of metagenomic data. *Genome Res.* **17**, 377–386 (2007).
6. Mylopotamitaki, D. *et al. Homo sapiens* reached the higher latitudes of Europe by 45,000 years ago. *Nature* **626**, 341–346 (2024).
7. Mathieson, I. *et al.* Genome-wide patterns of selection in 230 ancient Eurasians. *Nature* **528**, 499–503 (2015).
8. Fu, Q. *et al.* An early modern human from Romania with a recent Neanderthal ancestor. *Nature* **524**, 216–219 (2015).
9. Petr, M. *et al.* The evolutionary history of Neanderthal and Denisovan Y chromosomes. *Science* **369**, 1653–1656 (2020).
10. Rohrlach, A. B. *et al.* Using Y-chromosome capture enrichment to resolve haplogroup H2 shows new evidence for a two-path Neolithic expansion to Western Europe. *Sci. Rep.* **11**, 15005 (2021).
11. Svoboda, J. A., van der Plicht, J. & Kuželka, V. Upper Palaeolithic and Mesolithic human fossils from Moravia and Bohemia (Czech Republic): some new 14C dates. *Antiquity* **76**, 957–962 (2002).
12. Sponheimer, M. *et al.* Saving old bones: a non-destructive method for bone collagen prescreening. *Sci. Rep.* **9**, 1–7 (2019).
13. Fewlass, H. *et al.* Pretreatment and gaseous radiocarbon dating of 40–100 mg archaeological bone. *Sci. Rep.* **9**, 1–11 (2019).
14. Wacker, L., Němec, M. & Bourquin, J. A revolutionary graphitisation system: Fully automated, compact and simple. *Nucl. Instrum. Methods Phys. Res. B* **268**, 931–934 (2010).
15. Synal, H.-A., Stocker, M. & Suter, M. MICADAS: A new compact radiocarbon AMS system. *Nucl.*

Instrum. Methods Phys. Res. B **259**, 7–13 (2007).

16. Wacker, L. *et al.* MICADAS: Routine and high-precision radiocarbon dating. *Radiocarbon* **52**, 252–262 (2010).
17. Reimer, P. J. *et al.* The IntCal20 Northern hemisphere radiocarbon age calibration curve (0–55 cal kBP). *Radiocarbon* **62**, 725–757 (2020).
18. Bronk Ramsey, C. Bayesian analysis of radiocarbon dates. *Radiocarbon* **51**, 337–360 (2009).
19. Rohland, N., Glocke, I., Aximu-Petri, A. & Meyer, M. Extraction of highly degraded DNA from ancient bones, teeth and sediments for high-throughput sequencing. *Nat. Protoc.* **13**, 2447–2461 (2018).
20. Gansauge, M.-T. *et al.* Single-stranded DNA library preparation from highly degraded DNA using T4 DNA ligase. *Nucleic Acids Res.* **45**, e79 (2017).
21. Meyer, M. *et al.* A high-coverage genome sequence from an archaic Denisovan individual. *Science* **338**, 222–226 (2012).
22. Peyrégne, S. & Peter, B. M. AuthentiCT: a model of ancient DNA damage to estimate the proportion of present-day DNA contamination. *Genome Biol.* **21**, 246 (2020).
23. Zavala, E. I. *et al.* Quantifying and reducing cross-contamination in single- and multiplex hybridization capture of ancient DNA. *Mol. Ecol. Resour.* **22**, 2196–2207 (2022).
24. Huang, Y. & Ringbauer, H. hapCon: estimating contamination of ancient genomes by copying from reference haplotypes. *Bioinformatics* **38**, 3768–3777 (2022).
25. Posth, C. *et al.* Palaeogenomics of Upper Palaeolithic to Neolithic European hunter-gatherers. *Nature* **615**, 117–126 (2023).
26. Renaud, G., Stenzel, U. & Kelso, J. leeHom: adaptor trimming and merging for Illumina sequencing reads. *Nucleic Acids Res.* **42**, e141 (2014).
27. Li, H. & Durbin, R. Fast and accurate short read alignment with Burrows-Wheeler transform. *Bioinformatics* **25**, 1754–1760 (2009).
28. de Filippo, C., Meyer, M. & Prüfer, K. Quantifying and reducing spurious alignments for the analysis of ultra-short ancient DNA sequences. *BMC Biol.* **16**, 121 (2018).
29. Li, H. *et al.* The Sequence Alignment/Map format and SAMtools. *Bioinformatics* **25**, 2078–2079 (2009).
30. Prüfer, K. *et al.* The complete genome sequence of a Neanderthal from the Altai Mountains. *Nature* **505**, 43–49 (2014).
31. Prüfer, K. *et al.* A high-coverage Neandertal genome from Vindija Cave in Croatia. *Science* **358**, 655–658 (2017).
32. Prüfer, K. snpAD: an ancient DNA genotype caller. *Bioinformatics* **34**, 4165–4171 (2018).

33. McKenna, A. *et al.* The Genome Analysis Toolkit: a MapReduce framework for analyzing next-generation DNA sequencing data. *Genome Res.* **20**, 1297–1303 (2010).
34. Mafessoni, F. *et al.* A high-coverage Neandertal genome from Chagyrskaya Cave. *Proc. Natl. Acad. Sci. U. S. A.* **117**, 15132–15136 (2020).
35. Benson, G. Tandem repeats finder: a program to analyze DNA sequences. *Nucleic Acids Res.* **27**, 573–580 (1999).
36. Popli, D., Peyrégne, S. & Peter, B. M. KIN: a method to infer relatedness from low-coverage ancient DNA. *Genome Biol.* **24**, 1–22 (2023).
37. Fu, Q. *et al.* Genome sequence of a 45,000-year-old modern human from western Siberia. *Nature* **514**, 445–449 (2014).
38. Lazaridis, I. *et al.* Ancient human genomes suggest three ancestral populations for present-day Europeans. *Nature* **513**, 409–413 (2014).
39. Mallick, S. *et al.* The Simons Genome Diversity Project: 300 genomes from 142 diverse populations. *Nature* **538**, 201–206 (2016).
40. Ringbauer, H., Novembre, J. & Steinrücken, M. Parental relatedness through time revealed by runs of homozygosity in ancient DNA. *Nat. Commun.* **12**, 5425 (2021).
41. Hajdinjak, M. *et al.* Initial Upper Palaeolithic humans in Europe had recent Neanderthal ancestry. *Nature* **592**, 253–257 (2021).
42. Ringbauer, H. *et al.* ancIBD - Screening for identity by descent segments in human ancient DNA. *bioRxiv* (2023) doi:10.1101/2023.03.08.531671.
43. Seidman, D. N. *et al.* Rapid, phase-free detection of long identity-by-descent segments enables effective relationship classification. *Am. J. Hum. Genet.* **106**, 453–466 (2020).
44. Fernandes, D. M. *et al.* A genetic history of the pre-contact Caribbean. *Nature* **590**, 103–110 (2021).
45. Ringbauer, H., Coop, G. & Barton, N. H. Inferring recent demography from isolation by distance of long shared sequence blocks. *Genetics* **205**, 1335–1351 (2017).
46. Villalba-Mouco, V. *et al.* A 23,000-year-old southern Iberian individual links human groups that lived in Western Europe before and after the Last Glacial Maximum. *Nat. Ecol. Evol.* **7**, 597–609 (2023).
47. Bennett, E. A. *et al.* Genome sequences of 36,000- to 37,000-year-old modern humans at Buran-Kaya III in Crimea. *Nat. Ecol. Evol.* **7**, 2160–2172 (2023).
48. Fu, Q. *et al.* The genetic history of Ice Age Europe. *Nature* **534**, 200–205 (2016).
49. Villalba-Mouco, V. *et al.* Survival of Late Pleistocene Hunter-Gatherer Ancestry in the Iberian Peninsula. *Curr. Biol.* **29**, 1169–1177.e7 (2019).
50. Gallego Llorente, M. *et al.* Ancient Ethiopian genome reveals extensive Eurasian admixture

throughout the African continent. *Science* **350**, 820–822 (2015).

51. Lipson, M. *et al.* Ancient DNA and deep population structure in sub-Saharan African foragers. *Nature* **603**, 290–296 (2022).
52. Schlebusch, C. M. *et al.* Southern African ancient genomes estimate modern human divergence to 350,000 to 260,000 years ago. *Science* **358**, 652–655 (2017).
53. Jones, E. R. *et al.* Upper Palaeolithic genomes reveal deep roots of modern Eurasians. *Nat. Commun.* **6**, 8912 (2015).
54. Feldman, M. *et al.* Late Pleistocene human genome suggests a local origin for the first farmers of central Anatolia. *Nat. Commun.* **10**, 1218 (2019).
55. Lazaridis, I. *et al.* Genomic insights into the origin of farming in the ancient Near East. *Nature* **536**, 419–424 (2016).
56. van de Loosdrecht, M. *et al.* Pleistocene North African genomes link Near Eastern and sub-Saharan African human populations. *Science* **360**, 548–552 (2018).
57. Hajdinjak, M. *et al.* Reconstructing the genetic history of late Neanderthals. *Nature* **555**, 652–656 (2018).
58. Seguin-Orlando, A. *et al.* Genomic structure in Europeans dating back at least 36,200 years. *Science* **346**, 1113–1118 (2014).
59. Sikora, M. *et al.* Ancient genomes show social and reproductive behavior of early Upper Paleolithic foragers. *Science* **358**, 659–662 (2017).
60. Raney, B. J. *et al.* The UCSC Genome Browser database: 2024 update. *Nucleic Acids Res.* **52**, D1082–D1088 (2024).
61. UCSC Genome Browser Home. https://genome.ucsc.edu/.
62. Patterson, N. *et al.* Ancient admixture in human history. *Genetics* **192**, 1065–1093 (2012).
63. Busing, F. M. T. A., Meijer, E. & Van Der Leeden, R. Delete-m jackknife for unequal m. *Stat. Comput.* **9**, 3–8 (1999).
64. Danecek, P. *et al.* Twelve years of SAMtools and BCFtools. *GigaScience* **10**, giab008 (2021).
65. Petr, M., Vernot, B. & Kelso, J. admixr—R package for reproducible analyses using ADMIXTOOLS. *Bioinformatics* **35**, 3194–3195 (2019).
66. Devièse, T. *et al.* Direct dating of Neanderthal remains from the site of Vindija Cave and implications for the Middle to Upper Paleolithic transition. *Proc. Natl. Acad. Sci. U. S. A.* **114**, 10606–10611 (2017).
67. Li, H. & Durbin, R. Inference of human population history from individual whole-genome sequences. *Nature* **475**, 493–496 (2011).
68. Staab, P. R., Zhu, S., Metzler, D. & Lunter, G. scrm: efficiently simulating long sequences using the

approximated coalescent with recombination. *Bioinformatics* **31**, 1680–1682 (2015).

69. Pouyet, F., Aeschbacher, S., Thiéry, A. & Excoffier, L. Background selection and biased gene conversion affect more than 95% of the human genome and bias demographic inferences. *elife* **7**, e36317 (2018).
70. Johri, P. *et al.* The impact of purifying and background selection on the inference of population history: problems and prospects. *Mol. Biol. Evol.* **38**, 2986–3003 (2021).
71. McVicker, G., Gordon, D., Davis, C. & Green, P. Widespread genomic signatures of natural selection in hominid evolution. *PLoS Genet.* **5**, e1000471 (2009).
72. Gronau, I., Hubisz, M. J., Gulko, B., Danko, C. G. & Siepel, A. Bayesian inference of ancient human demography from individual genome sequences. *Nat. Genet.* **43**, 1031–1034 (2011).
73. Wang, K., Mathieson, I., O'Connell, J. & Schiffels, S. Tracking human population structure through time from whole genome sequences. *PLoS Genet.* **16**, e1008552 (2020).
74. Schiffels, S. & Durbin, R. Inferring human population size and separation history from multiple genome sequences. *Nat. Genet.* **46**, 919–925 (2014).
75. Kamm, J., Terhorst, J., Durbin, R. & Song, Y. S. Efficiently inferring the demographic history of many populations with allele count data. *J. Am. Stat. Assoc.* **115**, 1472–1487 (2020).
76. Pagani, L. *et al.* Genomic analyses inform on migration events during the peopling of Eurasia. *Nature* **538**, 238–242 (2016).
77. Peter, B. M. 100,000 years of gene flow between Neandertals and Denisovans in the Altai mountains. *bioRxiv* (2020) doi:10.1101/2020.03.13.990523.
78. Iasi, L. N. M. *et al.* Neandertal ancestry through time: Insights from genomes of ancient and present-day humans. *bioRxiv* (2024) doi:10.1101/2024.05.13.593955.
79. Hinch, A. G. *et al.* The landscape of recombination in African Americans. *Nature* **476**, 170–175 (2011).
80. Halldorsson, B. V. *et al.* Characterizing mutagenic effects of recombination through a sequence-level genetic map. *Science* **363**, eaau1043 (2019).
81. Moorjani, P. *et al.* A genetic method for dating ancient genomes provides a direct estimate of human generation interval in the last 45,000 years. *Proc. Natl. Acad. Sci. U. S. A.* **113**, 5652–5657 (2016).
82. Wang, R. J., Al-Saffar, S. I., Rogers, J. & Hahn, M. W. Human generation times across the past 250,000 years. *Sci. Adv.* **9**, eabm7047 (2023).
83. Iasi, L. N. M., Ringbauer, H. & Peter, B. M. An extended admixture pulse model reveals the limitations to human-Neandertal introgression dating. *Mol. Biol. Evol.* **38**, 5156–5174 (2021).
84. Kozubowski, T. J., Panorska, A. K., Qeadan, F., Gershunov, A. & Rominger, D. Testing exponentiality versus Pareto distribution via likelihood ratio. *Commun. Stat. Simul. Comput.* **38**,

118–139 (2008).

85. Petr, M., Pääbo, S., Kelso, J. & Vernot, B. Limits of long-term selection against Neandertal introgression. *Proc. Natl. Acad. Sci. U. S. A.* **116**, 1639–1644 (2019).
86. Massilani, D. *et al.* Denisovan ancestry and population history of early East Asians. *Science* **370**, 579–583 (2020).
87. The 1000 Genomes Project Consortium. A global reference for human genetic variation. *Nature* **526**, 68–74 (2015).
88. The Chimpanzee Sequencing and Analysis Consortium. Initial sequence of the chimpanzee genome and comparison with the human genome. *Nature* **437**, 69–87 (2005).
89. Prüfer, K. *et al.* The bonobo genome compared with the chimpanzee and human genomes. *Nature* **486**, 527–531 (2012).
90. Scally, A. *et al.* Insights into hominid evolution from the gorilla genome sequence. *Nature* **483**, 169–175 (2012).
91. Locke, D. P. *et al.* Comparative and demographic analysis of orangutan genomes. *Nature* **469**, 529–533 (2011).
92. Gibbs, R. A. *et al.* Evolutionary and biomedical insights from the rhesus macaque genome. *Science* **316**, 222–234 (2007).
93. Nassar, L. R. *et al.* The UCSC Genome Browser database: 2023 update. *Nucleic Acids Res.* **51**, D1188–D1195 (2023).
94. Harris, R. S. *Improved Pairwise Alignment of Genomic DNA.* (Pennsylvania State University, 2007).
95. Prüfer, K. *Alignments of Modern and Archaic Human Genomes with Primate Outgroup Genomes in Tab-Separated-Values Format.* (Edmond, 2021) doi:10.17617/3.5h.
96. Koenig, Z. *et al.* A harmonized public resource of deeply sequenced diverse human genomes. *bioRxiv* (2023) doi:10.1101/2023.01.23.525248.
97. Bergström, A. *et al.* Insights into human genetic variation and population history from 929 diverse genomes. *Science* **367**, eaay5012 (2020).
98. Byrska-Bishop, M. *et al.* High-coverage whole-genome sequencing of the expanded 1000 Genomes Project cohort including 602 trios. *Cell* **185**, 3426–3440.e19 (2022).
99. R Core Team. *R: A Language And Environment for Statistical Computing.* (R Foundation for Statistical Computing, Vienna, Austria, 2020).
100. Himmelmann, L. HMM: Hidden Markov Models. *R package version* **1**, (2010). doi: 10.32614/CRAN.package.HMM
101. Kong, A. *et al.* Fine-scale recombination rate differences between sexes, populations and individuals. *Nature* **467**, 1099–1103 (2010).

102. Wang, K. *et al.* High-coverage genome of the Tyrolean Iceman reveals unusually high Anatolian farmer ancestry. *Cell Genomics* **3**, 100377 (2023).

103. Begg, T. J. A. *et al.* Genomic analyses of hair from Ludwig van Beethoven. *Curr. Biol.* **33**, 1431–1447.e22 (2023).

104. Bieber, F. R. *et al.* Genetic studies of an acardiac monster: evidence of polar body twinning in Man. *Science* **213**, 775–777 (1981).

105. Fisk, N. M., Ware, M., Stanier, P., Moore, G. & Bennett, P. Molecular genetic etiology of twin reversed arterial perfusion sequence. *Am. J. Obstet. Gynecol.* **174**, 891–894 (1996).

106. McNamara, H. C., Kane, S. C., Craig, J. M., Short, R. V. & Umstad, M. P. A review of the mechanisms and evidence for typical and atypical twinning. *Am. J. Obstet. Gynecol.* **214**, 172–191 (2016).

107. Sankararaman, S., Mallick, S., Patterson, N. & Reich, D. The combined landscape of Denisovan and Neanderthal ancestry in present-day Humans. *Curr. Biol.* **26**, 1241–1247 (2016).

108. Vernot, B. *et al.* Excavating Neandertal and Denisovan DNA from the genomes of Melanesian individuals. *Science* **352**, 235–239 (2016).

109. Sankararaman, S. *et al.* The genomic landscape of Neanderthal ancestry in present-day humans. *Nature* **507**, 354–357 (2014).

110. International Society of Genetic Genealogy. *Y-DNA Haplogroup Tree 2019, Version: 15.73*, Date: 11 July 2020. https://isogg.org/tree/.

111. YFull. https://www.yfull.com/.

112. Immel, A. *et al.* Analysis of genomic DNA from medieval plague victims suggests long-term effect of *Yersinia pestis* on human immunity genes. *Mol. Biol. Evol.* **38**, 4059–4076 (2021).

113. Brandt, D. Y. C. *et al.* Mapping bias overestimates reference allele frequencies at the HLA genes in the 1000 Genomes Project phase I data. *G3 (Bethesda)* **5**, 931–941 (2015).

114. Pierini, F. *et al.* Targeted analysis of polymorphic loci from low-coverage shotgun sequence data allows accurate genotyping of HLA genes in historical human populations. *Sci. Rep.* **10**, 7339 (2020).

115. Langmead, B. & Salzberg, S. L. Fast gapped-read alignment with Bowtie 2. *Nat. Methods* **9**, 357–359 (2012).

116. Robinson, J. *et al.* The IPD and IMGT/HLA database: allele variant databases. *Nucleic Acids Res.* **43**, D423–D431 (2015).

117. González-Galarza, F. F. *et al.* Allele frequency net 2015 update: new features for HLA epitopes, KIR and disease and HLA adverse drug reaction associations. *Nucleic Acids Res.* **43**, D784–D788 (2015).

118. Wells, D. A. & McAuley, M. HLAfreq: Download and combine HLA allele frequency data. *bioRxiv*

(2023) doi:10.1101/2023.09.15.557761.

119. Childebayeva, A. *et al.* Population genetics and signatures of selection in Early Neolithic European farmers. *Mol. Biol. Evol.* **39**, msac108 (2022).

120. da Silva, N. A. *et al.* Admixture as a source for HLA variation in Neolithic European farming communities. *bioRxiv* (2023) doi:10.1101/2023.08.23.554285.

121. Nigh, R. B. The evolutionary potential of Lacandon Maya sustained-yield tropical forest agriculture. *J. Anthropol. Res.* **36**, 1–30 (1980).

122. Juárez-Martín, A. I., González-Sobrino, B. Z., Olvera, Á. E. C. & Falfán-Valencia, R. HLA class II alleles in the Otomi population of the Mezquital Valley: a genetic approach to the history of interethnic migrations in the Mexican Central Plateau. *Hum. Biol.* **86**, 167–184 (2014).

123. Arnaiz-Villena, A. *et al.* HLA genes in Chimila Amerindians (Colombia), the peopling of America and medical implications. *Int. J. Mod. Anthrop.* **1**, 91–116 (2016).

124. Arnaiz-Villena, A. *et al.* Study of Colombia North Wiwa El Encanto Amerindians HLA- genes: Pacific Islanders relatedness. *Hum. Immunol.* **79**, 530–531 (2018).

125. Fernandez Fernandez, T. L. *Vitality of Damana, the Language of the Wiwa Indigenous Community.* (Western University, 2020).

126. Barquera, R. *et al.* Diversity of HLA Class I and Class II blocks and conserved extended haplotypes in Lacandon Mayans. *Sci. Rep.* **10**, 3248 (2020).

127. Krause-Kyora, B. *et al.* Ancient DNA study reveals HLA susceptibility locus for leprosy in medieval Europeans. *Nat. Commun.* **9**, 1–11 (2018).

128. Immel, A. *et al.* Genome-wide study of a Neolithic Wartberg grave community reveals distinct HLA variation and hunter-gatherer ancestry. *Commun. Biol.* **4**, 113 (2021).

129. Rivollat, M. *et al.* Extensive pedigrees reveal the social organization of a Neolithic community. *Nature* **620**, 600–606 (2023).

130. SNPedia. https://www.snpedia.com/.

131. HIrisPlex-S Eye, Hair and Skin Colour DNA Phenotyping Webtool. https://hirisplex.erasmusmc.nl/.

132. Branicki, W. *et al.* Model-based prediction of human hair color using DNA variants. *Hum. Genet.* **129**, 443–454 (2011).

133. Ameur, A. *et al.* Genetic adaptation of fatty-acid metabolism: a human-specific haplotype increasing the biosynthesis of long-chain omega-3 and omega-6 fatty acids. *Am. J. Hum. Genet.* **90**, 809–820 (2012).

134. Fumagalli, M. *et al.* Greenlandic Inuit show genetic signatures of diet and climate adaptation. *Science* **349**, 1343–1347 (2015).

135. Sulem, P. *et al.* Genetic determinants of hair, eye and skin pigmentation in Europeans. *Nat. Genet.*

39, 1443–1452 (2007).
