# Supplementary material for: Earliest modern human genomes constrain timing of Neanderthal admixture
Source: Nature. 2024 Dec 12;638(8051):711–7. doi: 10.1038/s41586-024-08420-x (PMC11839475; doi:10.1038/s41586-024-08420-x)
Supplement: Supplementary file 2 — Reporting Summary [file 41586_2024_8420_MOESM2_ESM.pdf]

nature portfolio

Corresponding author(s): Arev Pelin Sümer
Kay Prüfer
Johannes Krause

Last updated by author(s): Arev Pelin Sümer

Reporting Summary

Nature Portfolio wishes to improve the reproducibility of the work that we publish. This form provides structure for consistency and transparency in reporting. For further information on Nature Portfolio policies, see our Editorial Policies and the Editorial Policy Checklist.

Statistics

For all statistical analyses, confirm that the following items are present in the figure legend, table legend, main text, or Methods section.

n/a	Confirmed	
☐	☒	The exact sample size (n) for each experimental group/condition, given as a discrete number and unit of measurement
☐	☒	A statement on whether measurements were taken from distinct samples or whether the same sample was measured repeatedly
☐	☒	The statistical test(s) used AND whether they are one- or two-sided<br>*Only common tests should be described solely by name; describe more complex techniques in the Methods section.*
☒	☐	A description of all covariates tested
☐	☒	A description of any assumptions or corrections, such as tests of normality and adjustment for multiple comparisons
☐	☒	A full description of the statistical parameters including central tendency (e.g. means) or other basic estimates (e.g. regression coefficient) AND variation (e.g. standard deviation) or associated estimates of uncertainty (e.g. confidence intervals)
☐	☒	For null hypothesis testing, the test statistic (e.g. F, t, r) with confidence intervals, effect sizes, degrees of freedom and P value noted<br>*Give P values as exact values whenever suitable.*
☐	☒	For Bayesian analysis, information on the choice of priors and Markov chain Monte Carlo settings
☒	☐	For hierarchical and complex designs, identification of the appropriate level for tests and full reporting of outcomes
☒	☐	Estimates of effect sizes (e.g. Cohen's d, Pearson's r), indicating how they were calculated

Our web collection on statistics for biologists contains articles on many of the points above.

Software and code

Policy information about availability of computer code

Data collection	no software was used
Data analysis	BWA (v. 0.5.10-evan.9-1-g44db244, https://github.com/mpieva/network-aware-bwa) , leeHom (v1.1.5), bam-rmdup (v0.2), SAMtools (v. 1.3.1), MapL (v0.1), AuthentiCT, hapCon_ROH, snpAD (v. 0.3.11), GATK (v. 1.3-14), SpAl (v. 2) , KIN, PSMC (v. 0.6.5), scrm (v. 1.7.3), qp3Pop (v.435; ADMIXTOOLS v.5.1), qpDstat (v.755; ADMIXTOOLS v.5.1), qpAdm (v.810), admixfrog (v.0.7.1), admixtools (v.2.0.0), BEDTools (v.2.25.0), Bowtie (v. 2.2.6), OptiType (v. 1.3.3). Data visualization was performed in RStudio (v.2022.12.0+353). The following R packages were used for visualization: cowplot (v.1.1.2), ggplot (v.3.4.2), tidyr (v.1.3.0), dplyr (v.1.1.4), magrittr (v.2.0.3), scales (v.1.3.0), MetBrewer (v.0.2.0).

For manuscripts utilizing custom algorithms or software that are central to the research but not yet described in published literature, software must be made available to editors and reviewers. We strongly encourage code deposition in a community repository (e.g. GitHub). See the Nature Portfolio guidelines for submitting code & software for further information.

Data

Policy information about availability of data

All manuscripts must include a data availability statement. This statement should provide the following information, where applicable:

- Accession codes, unique identifiers, or web links for publicly available datasets
- A description of any restrictions on data availability
- For clinical datasets or third party data, please ensure that the statement adheres to our policy

All newly reported ancient nuclear DNA data is archived in the European Nucleotide Archive (accession no. PRJEB78725). Additionally, the Alignment files used for the HLA analyses can be accessed through https://doi.org/10.17617/3.GHAALO.

Publicly available data was obtained from the following sources:
- Publicly available data in the European Nucleotide Archive: PRJEB39134 (Hajdinjak et al., 2021), PRJEB64496 (Bennett et al. 2023), PRJEB51862 (Posth et al., 2023), PRJEB58642 (Villalba-Mouco et al., 2023), PRJEB21157 (Prüfer et al., 2017), ERP002097 (Prüfer et al., 2014),
- Publicly available data in http://ftp.eva.mpg.de/genomes/ for high-coverage Loschbour, Stuttgart (LBK), Ust'-Ishim, Iceman and Beethoven genomes. Similarly, the high-coverage Neandertal genome from Chagyrskaya8 is publicly available in http://ftp.eva.mpg.de/neandertal/Chagyrskaya.
- High-coverage genome from Denisova3 is available in Short Read Archive: SRA047577(Meyer et al. 2012).
- SGDP data is available in the European Nucleotide Archive: PRJEB9586 and ERP010710 (Mallick et al., 2016). HGDP data is available in European Nucleotide Archive: PRJEB6463 and PRJEB14173 (Bergström et al., 2020).
- The HGDP and 1000 genomes curated data used for the Neandertal breakpoint sharing analyses is available through https://gnomad.broadinstitute.org/downloads#v3-hgdp-1kg
- hg19 reference genome is available through https://www.ncbi.nlm.nih.gov/datasets/genome/GCF_000001405.13/, and 1000 genome data can be obtained at https://www.internationalgenome.org/data/.

Research involving human participants, their data, or biological material

Policy information about studies with human participants or human data. See also policy information about sex, gender (identity/presentation), and sexual orientation and race, ethnicity and racism.

Reporting on sex and gender	We use biological data (genomes) from individuals that lived ~45,000 years ago, and infer their biological sex using the genomic data. We do not infer gender.
Reporting on race, ethnicity, or other socially relevant groupings	n/a
Population characteristics	n/a
Recruitment	n/a
Ethics oversight	n/a

Note that full information on the approval of the study protocol must also be provided in the manuscript.

Field-specific reporting

Please select the one below that is the best fit for your research. If you are not sure, read the appropriate sections before making your selection.

☒ Life sciences ☐ Behavioural & social sciences ☐ Ecological, evolutionary & environmental sciences

For a reference copy of the document with all sections, see nature.com/documents/nr-reporting-summary-flat.pdf

Life sciences study design

All studies must disclose on these points even when the disclosure is negative.

Sample size	Our sample consists of thirteen early modern human specimens from Ilsenhöhle in Ranis, Germany, described in Mylopotamitaki et al., 2024 in Nature. In addition, we generate additional data from the Zlaty kun individual from Czechia, introduced in Prüfer et al., 2021 in Nat. Ecol. Evol., and another specimen from the same site.
Data exclusions	Data from five specimens were excluded (RNI082, RNI083, RNI084, RNI086 and ZKU001) due to low endogenous DNA content in the librries obtained from these specimens, and/or high levels of present-day human DNA contamination. In addition, all sequences shorter than 30 basepairs and with a mapping quality of less than 25 were excluded.
Replication	Re sampled multiple individuals from the same site and because they belong to the same population, could replicate the popultion genetics results. Other than this, replication is not applicable.
Randomization	Randomization is not relevant and not carried out for this study. This is because we start the study with the specimens in hand and analyze

Randomization	the data from these specimens. We focus on the sequences per individual, independently and as a population. Randomization is not applicable in this context.
Blinding	Blinding is not applicable for ancient DNA/population genetics studies. We do not compare different treatments on different groups.

Behavioural & social sciences study design

All studies must disclose on these points even when the disclosure is negative.

Study description	
Research sample	
Sampling strategy	
Data collection	
Timing	
Data exclusions	
Non-participation	
Randomization	

Ecological, evolutionary & environmental sciences study design

All studies must disclose on these points even when the disclosure is negative.

Study description	
Research sample	
Sampling strategy	
Data collection	
Timing and spatial scale	
Data exclusions	
Reproducibility	
Randomization	
Blinding	

Did the study involve field work? ☐ Yes ☒ No

Field work, collection and transport

Field conditions	No field work was done for this study.
Location	
Access & import/export	
Disturbance	

Reporting for specific materials, systems and methods

We require information from authors about some types of materials, experimental systems and methods used in many studies. Here, indicate whether each material, system or method listed is relevant to your study. If you are not sure if a list item applies to your research, read the appropriate section before selecting a response.

Materials & experimental systems

n/a	Involved in the study
☒	☐ Antibodies
☒	☐ Eukaryotic cell lines
☐	☒ Palaeontology and archaeology
☒	☐ Animals and other organisms
☒	☐ Clinical data
☒	☐ Dual use research of concern
☒	☐ Plants

Methods

n/a	Involved in the study
☒	☐ ChIP-seq
☒	☐ Flow cytometry
☒	☐ MRI-based neuroimaging

Antibodies

Antibodies used

Validation

Eukaryotic cell lines

Policy information about cell lines and Sex and Gender in Research

Cell line source(s)

Authentication

Mycoplasma contamination

Commonly misidentified lines
(See ICLAC register)

Palaeontology and Archaeology

Specimen provenance: Specimens were collected from Ilsenhöhle in Ranis, Germany and Zlaty kun in Czechia. Permits were provided in Mylopotamitaki et al., 2024 in Nature and Prüfer et al., 2021 in Nat. Ecol. Evol..

Specimen deposition: Specimens were either returned to their corresponding collections (National Museum, Prague, Czechia, Landesamt für Denkmalpflege und Archäologie Sachsen-Anhalt-Landesmuseum für Vorgeschichte, Halle, Germany, Thuringian State Office for the Preservation of Historical Monuments and Archaeology, Weimar, Germany) or kept for further sampling in the cleanroom facilities of the Max Planck Institute for Evolutionary Anthropology in Leipzig.

Dating methods: Collagen was extracted following the acid-base-acid plus ultrafiltration protocol for small samples outlined in Fewlass et al., 201913 in the Ancient Genomics Lab at the Francis Crick Institute, UK. The extracted collagen was graphitised on an AGE 3 system14 and dated on a MICADAS accelerator mass spectrometer (AMS)15,16 at the Laboratory for Ion Beam Physics at ETH Zurich, Switzerland. Dates were calibrated using the IntCal20 calibration curve in OxCal 4.4.

☒ Tick this box to confirm that the raw and calibrated dates are available in the paper or in Supplementary Information.

Ethics oversight: No ethical guidance was required. Sampling was carried out under the guidance and with the permission of archaeologists/curators responsible for the material.

Note that full information on the approval of the study protocol must also be provided in the manuscript.

Animals and other research organisms

Policy information about studies involving animals; ARRIVE guidelines recommended for reporting animal research, and Sex and Gender in Research

Laboratory animals

Wild animals

Reporting on sex

Field-collected samples

Ethics oversight

Note that full information on the approval of the study protocol must also be provided in the manuscript.

Clinical data

Policy information about clinical studies

All manuscripts should comply with the ICMJE guidelines for publication of clinical research and a completed CONSORT checklist must be included with all submissions.

Clinical trial registration

Study protocol

Data collection

Outcomes

Dual use research of concern

Policy information about dual use research of concern

Hazards

Could the accidental, deliberate or reckless misuse of agents or technologies generated in the work, or the application of information presented in the manuscript, pose a threat to:

No	Yes	
☐	☐	Public health
☐	☐	National security
☐	☐	Crops and/or livestock
☐	☐	Ecosystems
☐	☐	Any other significant area

Experiments of concern

Does the work involve any of these experiments of concern:

No	Yes	
☐	☐	Demonstrate how to render a vaccine ineffective
☐	☐	Confer resistance to therapeutically useful antibiotics or antiviral agents
☐	☐	Enhance the virulence of a pathogen or render a nonpathogen virulent
☐	☐	Increase transmissibility of a pathogen
☐	☐	Alter the host range of a pathogen
☐	☐	Enable evasion of diagnostic/detection modalities
☐	☐	Enable the weaponization of a biological agent or toxin
☐	☐	Any other potentially harmful combination of experiments and agents

Plants

Seed stocks

Novel plant genotypes

Authentication

ChIP-seq

Data deposition

- [ ] Confirm that both raw and final processed data have been deposited in a public database such as GEO.
- [ ] Confirm that you have deposited or provided access to graph files (e.g. BED files) for the called peaks.

Data access links<br>*May remain private before publication.*	
Files in database submission	
Genome browser session<br>(e.g. UCSC)	

Methodology

Replicates	
Sequencing depth	
Antibodies	
Peak calling parameters	
Data quality	
Software	

Flow Cytometry

Plots

Confirm that:

- [ ] The axis labels state the marker and fluorochrome used (e.g. CD4-FITC).
- [ ] The axis scales are clearly visible. Include numbers along axes only for bottom left plot of group (a 'group' is an analysis of identical markers).
- [ ] All plots are contour plots with outliers or pseudocolor plots.
- [ ] A numerical value for number of cells or percentage (with statistics) is provided.

Methodology

Sample preparation	
Instrument	
Software	
Cell population abundance	
Gating strategy	

- [ ] Tick this box to confirm that a figure exemplifying the gating strategy is provided in the Supplementary Information.

Magnetic resonance imaging

Experimental design

Design type	
Design specifications	
Behavioral performance measures	

Acquisition

Imaging type(s)

Field strength

Sequence & imaging parameters

Area of acquisition

Diffusion MRI ☐ Used ☐ Not used

Preprocessing

Preprocessing software

Normalization

Normalization template

Noise and artifact removal

Volume censoring

Statistical modeling & inference

Model type and settings

Effect(s) tested

Specify type of analysis: ☐ Whole brain ☐ ROI-based ☐ Both

Statistic type for inference
(See Eklund et al. 2016)

Correction

Models & analysis

n/a	Involved in the study
☐	☐ Functional and/or effective connectivity
☐	☐ Graph analysis
☐	☐ Multivariate modeling or predictive analysis

Functional and/or effective connectivity

Graph analysis

Multivariate modeling and predictive analysis
